# A MAGIBU-based model for pediatric and juvenile CNS tumors: an in-house epigenetic decision-support framework compared with online DNA methylation classifiers

**DOI:** 10.17879/freeneuropathology-2026-9648

**Published:** 2026-08-24

**Authors:** Gianluca Mattei, Laura Giunti, Mirko Scagnet, Rina Agushi, Federico Mussa, Chiara Caporalini, Iacopo Sardi, Vincenzo Yuto Civale, Alberto Magi, Lorenzo Genitori, Anna Maria Buccoliero

**Affiliations:** 1 UOR4 Cellular and Morphofunctional Neurobiology, Meyer Children’s Hospital IRCCS, Florence, Italy; 2 Neuro-Oncology Unit, Meyer Children’s Hospital IRCCS, Florence, Italy; 3 Neurosurgery Unit, Meyer Children’s Hospital IRCCS, Florence, Italy; 4 Pathology Unit, Meyer Children’s Hospital IRCCS, Florence, Italy; 5 Department of Information Engineering, University of Florence, Florence, Italy

**Keywords:** DNA methylation, Central nervous system, Tumor, Children, Epigenetic, Brain, MAGIBU

## Abstract

**Background:** DNA methylation profiling is a tool that provides key support for central nervous system (CNS) tumor classification. However, diagnostically ambiguous pediatric cases may result in discordant outputs across classifiers. We developed MAGIBU, a cross-platform, projection-based framework that embeds individual methylomes into a fixed CNS reference landscape, ranking diagnostic entities by local epigenetic proximity to support clinician-led integrative diagnosis.

**Methods:** As a proof-of-concept, we evaluated MAGIBU in eight morphologically challenging pediatric/juvenile CNS tumors with unresolved diagnoses after institutional and central pathology review. To establish a benchmark in the absence of a definitive histopathological ground truth, a consensus epigenetic reference was defined a priori for cases showing concordant results between the Heidelberg CNS Tumor Methylation Classifier and Methylscape Analysis. Comparisons were also performed with Epigenomic Digital Pathology (EpiDiP). To validate MAGIBU beyond this discovery cohort, performance was assessed at the family level across the CNS methylation spectrum (n = 678, 28 methylation families), on non-array platforms (whole-genome bisulfite sequencing and Oxford Nanopore), and in a focused analysis of the low-grade glioma and diffuse midline glioma compartment across four independent cohorts (n = 670).

**Results:** In the discovery cohort, MAGIBU achieved high concordance with the consensus reference (Cohen’s κ = 0.855), outperforming EpiDiP (κ = 0.278), which frequently placed low-grade tumors in proximity to higher-grade reference regions.

**Conclusions:** MAGIBU provides a stable, quantitative differential diagnosis framework that mitigates the limitations of rigid categorical assignments. By leveraging a distance-based proximity metric, it offers a transparent decision-support tool that integrates effectively with clinical, radiological, and molecular data. While performance is inherently dependent on reference atlas composition, MAGIBU represents a robust complementary approach for the diagnostic workup of ambiguous CNS tumors.

## Introduction

DNA methylation is a fundamental epigenetic mechanism regulating gene expression [1]. In central nervous system (CNS) tumors, methylation profiling has substantially refined tumor classification and is now integrated into the latest World Health Organization (WHO) diagnostic guidelines [2]. Indeed, publicly available machine-learning-based classifiers for DNA methylation data significantly improve tumor diagnosis and are often decisive in cases with ambiguous histology [1]. Nevertheless, challenges persist in pediatric and juvenile CNS tumors, in tumors arising in the context of genetic syndromes or following radiotherapy, and in cases characterized by a significant intratumoral inflammatory or non-neoplastic component [3,4,5,6,7].

The present study focuses on a previously published cohort of eight pediatric and juvenile CNS tumors with histologically unresolved diagnoses, which had previously been analyzed using publicly available DNA methylation classifiers (Heidelberg CNS Tumor Methylation Classifier v12.8.1 and Methylscape Analysis v2.0) [8]. Building on this cohort, we reanalyzed the tumors using EpiDiP (Epigenomic Digital Pathology) and our newly developed framework, MAGIBU. Unlike the Random Forest-based Heidelberg CNS Tumor Methylation Classifier, which outputs a probabilistic class score, Methylscape Analysis performs classification with a Support Vector Machine and uses dimensionality reduction (UMAP) only to visualize tumor profiles in its interactive reports, whereas EpiDiP relies on dimensionality reduction (t-SNE or UMAP) itself to position tumor profiles relative to a large reference epigenomic dataset, aiding diagnostic interpretation through pattern recognition. In contrast, MAGIBU generates cross-platform epigenetic models that complement existing DNA methylation classifiers. It embeds individual cases, regardless of acquisition technology, into a well-annotated reference landscape and applies a distance-based framework to quantify their similarity to established tumor classes in a low-dimensional space, providing an additional layer of interpretative support for challenging cases. Rather than delivering a single categorical assignment, this proximity-based approach generates a ranked differential diagnosis, explicitly acknowledging diagnostic uncertainty and fostering an integrated, pathologist- centered interpretation that combines epigenetic, histological, and molecular information.

Our aim was to evaluate MAGIBU’s performance in diagnostically challenging cases and to assess its potential as a complementary tool for CNS tumor diagnosis. More generally, MAGIBU is not a single fixed classifier, but a framework that builds methylation classification models from any annotated reference, so that its scope is defined by the chosen reference rather than by the tool itself. In this work the framework was used to generate a CNS model: it was evaluated across the broader CNS methylation spectrum to assess its general applicability, and specifically in low-grade gliomas (LGG) and diffuse midline gliomas (DMG), for which the validation cohort was deliberately restricted by excluding samples outside this dichotomy.

## Patients and methods

### Patients

This study included eight pediatric and juvenile patients who underwent CNS tumor surgery at Meyer Children’s Hospital IRCCS (Florence, Italy). Eligible consecutive cases had inconclusive institutional and central review diagnoses, surgery performed at least five years prior to analysis, and available fresh-frozen tissue, ensuring optimal conditions for all experiments.

Clinical and pathological features are summarized in **Table 1**. All patients were male, aged four months to 23 years, with tumors located in the brainstem (four cases), cerebral hemispheres (two cases), cerebellum (one case), and spinal cord (one case). Proposed diagnoses (institutional and centralized) included LGG not otherwise specified (NOS) in three cases (patients 5, 6, 8), low-grade glioneuronal tumor NOS in one case (patient 3), and PNET-like tumor in one case (patient 4). The remaining three cases lacked diagnostic concordance. Patient 2 was classified as LGG NOS with possible high-grade area by the institution versus glioma NOS centrally. Patient 7 was interpreted as possible glioma NOS institutionally and neurocytoma-like tumor centrally. The last of these three cases, patient 1, was a lesion considered as a possible glioma NOS by the institutional pathologist and as a possible mixed glioneuronal tumor NOS at the centralized review. The tumor was resected a year later to remove residual tissue, with the institutional diagnosis revised to rosette-forming glioneuronal tumor (the second specimen was not submitted for centralized review).

**Table 1:** 

**ID**	**Age**	**Sex**	**Location**	**Histological diagnosis**		**Heidelberg CNS Tumor Classification (score)**	**Methylscape Analysis (score)**	**EpiDiP**	**MAGIBU**	**Relevant CNVs Heidelberg CNS Tumor Classification, Methylscape Analysis and EpiDiP**	***MGMT*** ** promoter methylation Heidelberg CNS Tumor Classification and Methylscape Analysis**	**Notes**
				**Institutional**	**Central review**	**FAMILY, CLASS**	**FAMILY, CLASS**					
1	5 m	M	Cerebellar vermis	LGG NOS	LGGN	Low Grade Glial/glioneuronal/neuroepithelial Tumours (0,95); Mc Pilocytic Astrocytoma Infratentorial, Subclass Fgfr1 Altered (0,93)	Low Grade Glial/glioneuronal/ Tumours (0,962), Pilocytic Astrocytoma Infratentorial, Subclass Fgfr1 Altered (0,676)	LGG_PA_MID (67%); LGG_PA_PF (33%)	LGG, PA PF (0,05); LGG, PA MID (0,30); LGG, PA/GG ST (1,19)	Flat	Unmethylated	New surgery one year later. Institutional diagnosis was RFGNT
2	23 y	M	Midbrain (tectal plate)	LGG with areas of anaplastic progression	LGG NOS	Low Grade Glial/glioneuronal/neuroepithelial Tumours (0,97); Pilocytic Astrocytoma (0,95)	Low Grade Glial/glioneuronal/neuroepithelial Tumours (0,992), Pilocytic Astrocytoma Infratentorial (0,988)	ANA_PA (47%); DLGNT (13%); LGG_PA_PF (13%)	LGG, PA/GG ST (0,023); LGG, PA PF (0,29); CONTR, REACT (0,52)	Flat	Methylated	Lesion under follow-up for several years increased in size and changed in features.
3	13 y	M	Parietal lobe right	LGGN NOS	LGGN NOS	Low Grade Glial/glioneuronal/neuroepithelial Tumours (0,89); Mc Ganglioglioma (0,88)	Low Grade Glial/glioneuronal Tumours (0,771), Ganglioglioma (0,998)	HPAP (87%); LGG_PA_PF (7%); LGG_PA_GG_ST (7%)	LGG, GG (0,07); IHG (0,38); ANA PA (0,52)	9p* (CDKN2A/B), *9q* (PTCH1) and 19q *deletions	Unmethylated	/
4	8 y	M	Pons-midbrain	PNET like	PNET like	Pediatric Type Diffuse High Grade Gliomas (0,95); Mc Diffuse Midline Glioma, H3 K27 Altered, Subtype H3 K27 Mutant or Ezhip Expressing (0,92)	Diffuse Midline Glioma H3 K27 Altered, SubtypeH3 K27 Altered (0,983), Diffuse Midline Glioma H3 K27 Altered (1)	DMG_K27 (100%)	DMG, K27 (0,02); GBM, MYCN (1,19); GBM, G34 (2,31)	1p deletion, 1q (*MDM4*) duplication, 3p deletion (*MLH1, VHL, PDCD1*), *PDGFRA *amplification (4p), 4q deletion, 13q deletion (*RB1*), 16p deletion	Unmethylated	CSF dissemination and spinal metastases at the disease onset; 3 cycles of neoadjuvant chemotherapy (HART protocol)
5	9 y	M	Spinal cord (D9–D10)	LGG NOS	LGG NOS	Low Grade Glial/glioneuronal/neuroepithelial Tumours (0,86); Mc Diffuse Leptomeningeal Glioneuronal Tumour Subtype 1 (0.86)	Intermediate grade IDH wildtype gliomas (0.991), Diffuse Leptomeningeal Glioneuronal Tumour Subtype 1 (0.996)	ANA_PA (60); DLGNT (20%); EPN_SPINE (7%)	ANA PA (0,10); LGG, PA/GG ST (0,23); PXA (0,44)	1p and 19q deletions	Unmethylated	/
6	4 y	M	Medulla oblongata	LGG NOS	LGG NOS	Low Grade Glial/glioneuronal/neuroepithelial Tumours (0,99); Mc Pilocytic Astrocytoma Infratentorial (0,94)	Low Grade Glial/glioneuronal Tumours (0,992), Pilocytic Astrocytoma (0,993)	ANA_PA (40%); LGG_PA_GG_ST (13%); LGG_PA_PF (13%)	LGG, PA PF (0,12); LGG, PA MID (0,28); CONTR, REACT (0,30)	Flat	Unmethylated	Adjuvant chemotherapy (modified version of SIOP LGG 2004 protocol)
7	5y	M	Midbrain (tectal plate)	LGG NOS unpredictable prognosis	Neurocytoma-like unpredictable prognosis	Low Grade Glial/glioneuronal/neuroepithelial Tumours (0,99); Mc Pilocytic Astrocytoma Infratentorial (0,98)	Low Grade Glial/glioneuronal Tumours (0,991), Pilocytic Astrocytoma (0,996)	ANA_PA (47%); LGG (20%); SUBEPN_ST (7%)	LGG, PA PF (0,10); LGG, PA MID (0,15); CONTR, REACT (0,54)	5p (*TERT*) and 5q duplication, 6p and 6q (*MYB*) duplication, 7p (*EGFR*) and 7q (*CDK6*,* MET* and* KIAA1549/BRAF*) duplications, 19p and 19q (*C19MC, BRD4*) deletions	Unmethylated	/
8	5y	M	Temporal lobe right	LGG NOS	LGG NOS	No match Control tissue (0.48); Mc Control tissue, Cerebral Hemisphere (0,47)	Low Grade Glial/glioneuronal Tumours (0,867), Ganglioglioma (0,919)	HPAP (33%); CONTR_HEMI (13%); GBM_LOW (13%)	CONTR, HYPTHAL (0,09); CONTR, PONS (0,10); CONTR, WM (0,16)	Flat	Unmethylated	/

**Table 1.** Clinical, histological, and molecular data. In the EpiDiP column, values indicate the frequencies of the nearest diagnostic classes. In the MAGIBU column the first three nearest tumoral classes are shown. Values in parentheses indicate the average Euclidean distance for each projected sample to samples belonging to the corresponding category; lower values indicate higher similarity of the sample to that category. y: years; m: months; LGG: low grade glioma; NOS: not otherwise specified; PNET: primitive neuroectodermal tumor; ANA_PA: anaplastic pilocytic astrocytoma; DLGNT: diffuse leptomeningeal glioneuronal tumor; LGG_PA_PF: low grade pilocytic astrocytoma posterior fossa; EPN_SPINE: ependymomas spinal; HPAP: high-grade glioma with pleomorphic and pseudopapillary features; LGG_PA_GG_ST: low grade glioma subclass hemispheric pilocytic astrocytoma and ganglioglioma; LGG_PA_MID: low grade glioma subclass midline pilocytic astrocytomas; DMG_K27: diffuse midline glioma H3K27M mutant; SUBEPN_ST: subependymoma supratentorial; IHG: infantile hemispheric glioma; GBM_LOW: Glioblastoma, IDHWT NOS, low tumor cell content; GBM, MYCN: glioblastoma, IDH wildtype, subclass MYCN; GBM, G34: glioblastoma, IDH wildtype, H3.3 G34 mutant; PXA: (anaplastic) pleomorphic xanthoastrocytoma; LGG, GG: low grade glioma, ganglioglioma; CONTROL, REACT: control tissue, reactive tumor microenvironment; CONTROL_HEMI: control tissue, hemispheric cortex; CONTR, HYPTHAL: control hypothalamus; CONTR, PONS: control pons; CONTR, WM: control white matter; RFGNT: rosette forming glioneuronal tumor.

This cohort had previously been published and characterized using DNA methylation profiling with publicly available online classifiers (Heidelberg CNS Tumor Methylation Classifier v12.8.1 and the Methylscape Analysis v2.0) [8].

Preoperative informed consent for the conservation of frozen tumor specimens and any subsequent molecular analyses was obtained by the neurosurgical team. The study was approved by the Institutional Ethical Committee.

We selected this small cohort as a proof-of-concept study, focusing on diagnostically challenging cases with unresolved histopathology to evaluate the MAGIBU framework in a real-world, difficult-to-classify setting.

### Methods

DNA methylation profiling was performed using the Infinium MethylationEPIC BeadChip on the Illumina platform, following the manufacturer’s instructions. Raw data were processed and normalized, and methylation patterns were classified using the following publicly available DNA methylation-based classifiers: the Heidelberg CNS Tumor Methylation Classifier v12.8.1 (https://epignostix.com/our-technology/our-technology-cns-tumor-classifier/) and the Methylscape Analysis v2.0 (https://methylscape.ccr.cancer.gov/) in the previously published study [8], and the Epigenomic Digital Pathology (EpiDiP) (https://epidip.usb.ch/ submission January 15th 2026) in the present analysis. Reports from the Heidelberg CNS Tumor Methylation Classifier and Methylscape Analysis include the most likely match between a calibrated score and a chromosomal copy number variation (CNV) plot, as well as the methylation status of the O6-methylguanine-DNA-methyl-transferase (MGMT) promoter. The EpiDiP classifier, run by selecting the 50,000 most variable CpG sites, matching the feature set size used for MAGIBU, and projecting the samples against the reference epigenomic landscape provided by the EpiDiP portal, displays a methylation UMAP plot showing the percentage of closest pathological or non-pathological samples (methylation UMAP score), alongside a copy number plot of CNVs. The CNVs refer to diagnostically relevant regions, which in part overlap for Heidelberg CNS Tumor Methylation Classifier and Methylscape Analysis, but differ for the EpiDiP classifier.

The MAGIBU framework was used to develop a specific distance-based classification model. This specific implementation utilized a reference cohort of 3,905 CNS tumor samples (GSE109381) profiled on the Illumina Infinium HumanMethylation 450BeadChip array (450K) [9], with established histological and molecular diagnoses.

The training dataset is composed of 90 CNS DNA methylation classes, which define the complete label space among which the classifier can discriminate. To ensure consistency across platforms, raw methylation data for test samples were normalized according to the array type: EPICv2 samples were processed using the preprocessRaw function, while 450k reference samples were normalized using the preprocessIllumina function, which includes background correction and control-probe normalization. Cross-platform integration between the 450K reference dataset and our EPICv2 test samples was achieved by retaining only CpG sites mapping to identical genomic coordinates using the packages IlluminaHuman-Methylation450kanno.ilmn12.hg19 and Illumina-HumanMethylationEPICv2anno.20a1.hg38 respectively. Since the 450K probes were originally mapped to hg19, their coordinates were lifted over to hg38. Missing values were imputed using the mean beta value of each CpG position calculated from the reference dataset. Furthermore, the tool extends its functionality to EPICv1 and Oxford Nanopore (ONT) data. EPICv1 samples are processed using the same background-corrected normalization protocol as the 450k arrays. For Nanopore data, the tool employs an optimized stream-reading approach to extract methylation frequencies, with β-values rounded to four decimal places. To maintain compatibility with array-based references, Nanopore methylation sites are assigned a unique Probe ID based on their chromosome:position coordinates. For dimensionality reduction, the 50,000 CpG sites with the highest variance across the reference samples were selected. Principal component analysis (PCA) was computed on the filtered reference matrix, retaining the first 50 principal components. Then the package uwot R package (v0.1.14) was used (parameters: n_neighbors = 30, min_dist = 0.1), to compute the UMAP and score the Euclidean distance metric. Crucially, MAGIBU serializes both the PCA rotation matrix and the learned UMAP manifold structure, creating a frozen reference landscape that allows for the instantaneous projection of prospective samples without the need for model retraining, reducing both the variability and the time needed for predictions. The choice of 50,000 CpG sites was supported by a feature set sensitivity analysis in which the number of selected features was varied from 500 to 100,000, evaluated on two validation settings: an array reference cohort spanning the full CNS spectrum (GSE289137) and sequencing data, whole genome bisulfite sequencing and Oxford Nanopore, covering only a limited set of families (GSE289246) [10]. On the array cohort, family level accuracy increased steeply up to approximately 10,000 sites and then plateaued (**Supplementary Figure 1**). On the sequencing data, by contrast, accuracy kept improving and was maximal at approximately 50,000 sites, where the pooled nanopore samples reached 100 % and per sample whole genome bisulfite sequencing reached 95.5 % family level accuracy, compared with 86.4 % and 25 to 60 % at 10,000 sites (**Supplementary Figure 2**). The 50,000 site configuration was therefore selected as the best trade off across platforms. For the inference phase, the MAGIBU projection module incorporates a dynamic feature alignment layer specifically engineered to integrate heterogeneous data sources, including standard DNA methylation arrays (450k/EPIC) and low-coverage Nanopore sequencing. To address genomic coordinate discrepancies between 0-based (BED) and 1-based (Illumina) formats, the framework utilizes a probe mapping algorithm that identifies reference CpG sites via exact positional matches as well as single-base offsets (±1 bp). Probes present in the reference model but missing in the test samples are handled via a neutral imputation strategy (beta value = 0.5) to preserve the input matrix structure required by the pre-trained model. Test samples are subsequently transformed using the stored PCA loadings and projected into the existing UMAP space using the umap-transform function.

**Figure 1:**
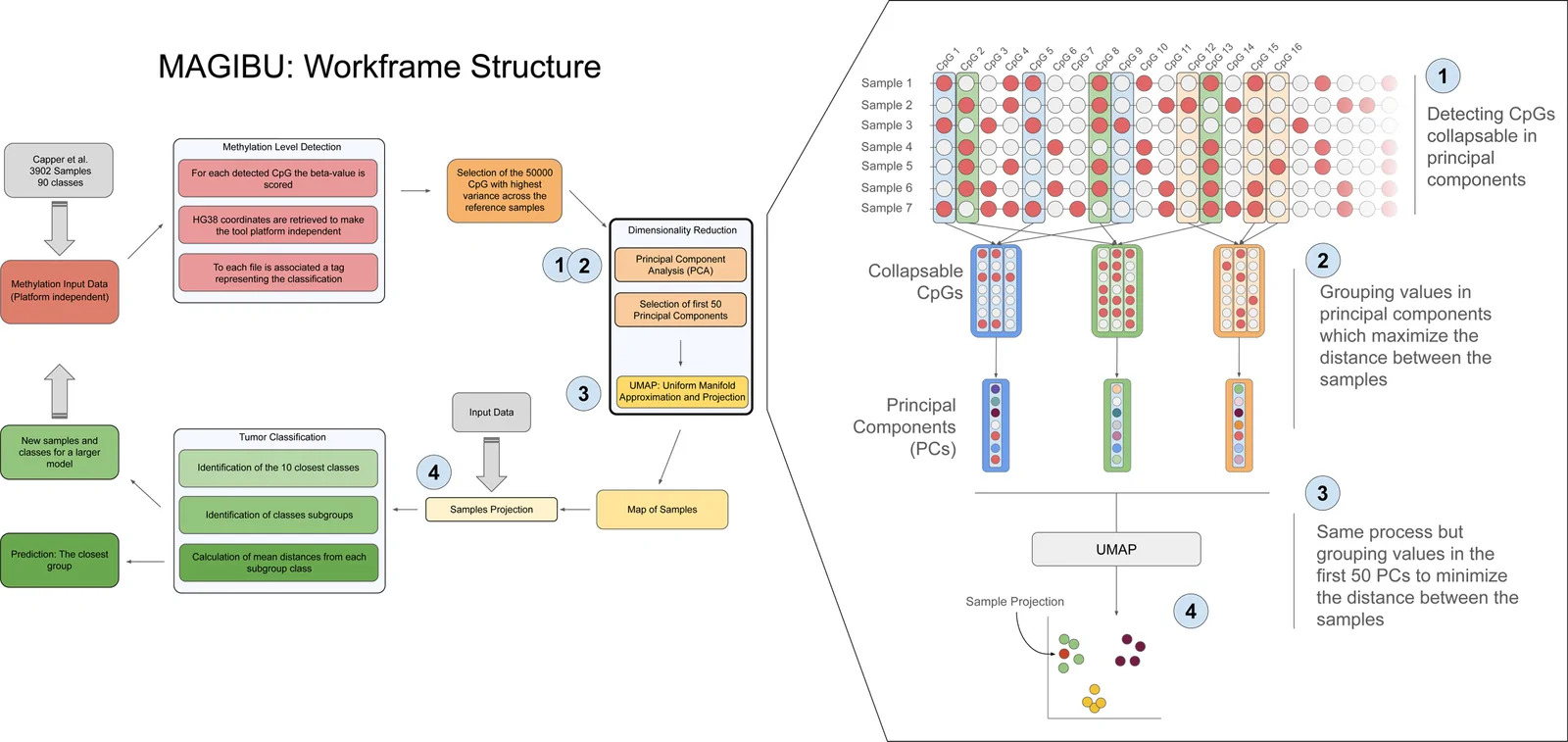
**Figure 1.** **Workflow and Dimensionality Reduction Strategy.** **Left image:** The schematic illustrates the MAGIBU pipeline. The workflow, for CNS tumor classification begins with platform-independent input data (standardized via hg38 coordinates) integrated with a reference training set according to Capper et al. [9]. Feature selection isolates the 50,000 most variable CpGs. Dimensionality reduction and projection, followed by tumor classification. Crucially, the framework is designed to be iterative: validated predictions from new samples can be incorporated back into the dataset, allowing the reference model to progressively expand and refine its diagnostic resolution. **Right image:** A conceptual visualization of the dimensionality reduction and classification process. High-dimensional methylation data (the selected 50,000 CpG probes) are "collapsed" into the first 50 Principal Components (PCA) to extract essential biological variance. These components are then projected into a 2D UMAP embedding, preserving local and global data structures. Final classification is performed by identifying the closest reference subgroups in this topological space and calculating the mean distances to the target sample.

Classification within the MAGIBU framework is driven by a class-specific local neighborhood topology designed to resolve intra-class heterogeneity. Rather than relying on global centroids or simple majority, the algorithm computes a proximity metric based on the mean Euclidean distance to the nearest class representatives. For each methylation class defined in the reference atlas, MAGIBU identifies the k nearest neighbors (default k = 5) relative to the projected target sample. The mean distance to this local subset is calculated, effectively isolating the most biologically similar sub-population of each tumor entity while ignoring distant outliers and classes. The system ranks all reference classes based on this proximity score, assigning the primary diagnosis to the entity with the lowest mean distance. The top 10 ranked classes are visualized in a bar plot alongside the UMAP projection, providing pathologists with a quantitative differential diagnosis that explicitly models the local density and biological similarity of the target sample within the reference landscape (**Figure 1**).

Reference diagnoses were defined for seven of the eight cases that showed concordant results between the Heidelberg CNS Tumor Methylation Classifier and Methylscape Analysis, thereby serving as an operational consensus reference. The eighth case, which yielded discordant results between the two primary classifiers, was evaluated independently to assess MAGIBU’s behavior in settings of high diagnostic uncertainty.

To evaluate the degree of agreement between the epigenetic classifiers (MAGIBU and EpiDiP) and the established diagnostic standard, the Cohen’s Kappa coefficient was calculated. The consensus reference was defined based on cases showing concordant results between the Heidelberg CNS Tumor Methylation Classifier and Methylscape Analysis. For the MAGIBU framework, a correct identification of the primary tumor class was considered an agreement, regardless of whether the specific molecular subtype was identified.

For a focused analysis of the LGG and DMG compartment, MAGIBU accuracy was validated on four independent cohorts: sturm_panCNS (GEO GSE215240, n = 524) [11], CBTN (Children's Brain Tumor Network, n = 349) [12], sturm_pnet (GEO GSE73801, n = 73) [13], and an in-house cohort (n = 30). Ground-truth labels were harmonized into a controlled vocabulary and collapsed into two macro-classes: LGG (any low-grade glioma family entity, including PA_PF, PA_MID, GG, DNT, PXA, MYB, SEGA, DLGNT, PLNTY, RGNT, ANA PA, IHG, PA/GG ST, DIG/DIA, and coarse "LGG") and DMG (DMG, K27-altered). For the in-house cohort, ground-truth labels were assigned by 2-of-3 consensus among Heidelberg CNS Tumor Methylation Classifier, Methylscape Analysis Bethesda v2.0, and MAGIBU, with ties resolved by Methylscape Analysis. Samples whose ground truth or MAGIBU prediction fell outside the LGG/DMG dichotomy (HGG without DMG subclass, controls, pituitary, other CNS entities) were excluded from the analysis on both sides. Per cohort and class strata with n < 3 were excluded from the radar visualization to avoid single-sample artifacts. Confusion matrices were computed globally and per cohort and row normalized; class recall was computed as the proportion of correctly predicted samples per stratum. The sample selection and exclusion flow for this validation is summarized in **Supplementary Figure 3**.

MAGIBU was evaluated across the full CNS methylation spectrum on an independent CNS array cohort from the crossNN [10] reference set (GSE289137, EPICv1 and EPICv2), spanning 28 methylation families (n = 678). Predicted and ground truth labels were harmonized to a common family vocabulary (**Supplementary Table 1**), and concordance was scored at the family level as Top 1 agreement (the highest ranked prediction matches the reference family) and Top 3 agreement (the reference family appears among the three highest ranked predictions) (**Supplementary Table 2**). Resolution within the LGG and DMG compartment was additionally examined at the subclass level. To probe applicability across acquisition platforms, MAGIBU was further applied, as a feasibility analysis, to whole genome bisulfite sequencing and Oxford Nanopore data from the crossNN dataset; sample handling, coordinate harmonization and the sample pooling procedure used for low coverage Nanopore profiles are described in the **Supplementary Methods**.

Computational performance was benchmarked on a single workstation (Intel Xeon Gold 6230 CPU at 2.10 GHz, 360 logical CPUs, 1.5 TB RAM, Red Hat Enterprise Linux 8.6, no GPU). Peak memory and wall clock time for building a reference model were measured as a function of the number of selected CpG sites (500 to 100,000) and of the number of allocated CPU cores (12, 16, 32 and 64) (**Supplementary Figure 4**); scaling with the size of the reference atlas was assessed on random subsets of 500, 1,000, 2,000 and 3,905 reference cases at 50,000 CpG sites and 32 cores, sampled with a fixed class composition (**Supplementary Figure 5**).

## Results

MAGIBU was first evaluated across the full CNS spectrum on an independent array cohort from the crossNN [10] reference set (GSE289137) spanning 28 methylation families (n = 678). The tumor family was recovered as the top ranked call for the majority of samples, and Top 3 concordance further increased recovery in underrepresented families (**Supplementary Figure 6**; per family counts in **Supplementary Table 2**). Well represented families were classified with high family level accuracy, including astrocytoma IDH mutant (96.6 % Top 1), ependymoma (97.8 %), meningioma (100 %), medulloblastoma (100 %) and glioblastoma (94.3 %), whereas families represented by very few reference samples contributed most of the misclassifications. Overall, the correct family was recovered as the top ranked call in 87.2 % of samples (591/678) and within the three closest calls in 93.2 % (632/678).

We then examined in greater depth the LGG and DMG compartment, the clinical focus of this study, on four independent cohorts (sturm_panCNS, GSE215240; CBTN, Children's Brain Tumor Network; sturm_pnet, GSE73801; and an additional in-house series). After restricting the comparison to samples whose ground truth and MAGIBU prediction were both either LGG or DMG (n = 670), MAGIBU correctly classified 99.8 % of LGG samples (593/594) and 96.1 % of DMG samples (73/76) at the class level (**Supplementary Figure 7a**). Performance was consistent across the four cohorts (**Supplementary Figure 7b**). In sturm_panCNS, LGG recall reached 100 % (347/347) and DMG recall was 90.9 % (30/33); in CBTN, LGG recall was 99.5 % (215/216) and DMG recall was 100 % (24/24); in sturm_pnet, both classes were recalled at 100 % (10/10 and 19/19, respectively); in the in-house cohort, LGG recall was 100 % (21/21), with no DMG case passing the inclusion filter. The macro mean recall across the seven cohort by class strata satisfying the n3 criterion was 98.6 % (**Supplementary Figure 7c**). Within this compartment, subclass level concordance is reported in **Supplementary Figure 8**.

To extend the head-to-head comparison with EpiDiP beyond the initial eight cases, both classifiers were additionally evaluated on a subset of 31 crossNN [10] samples (GSE289137) spanning 13 CNS methylation families that cover the principal diagnostic categories of the WHO classification [2]. Because EpiDiP predictions must be obtained one by one through its web portal, the comparison was restricted to a representative subset rather than the full cohort, selecting the first two samples from each class. The complete sample list is given in **Supplementary Table 3**. On this subset MAGIBU reached a family level Top 1 accuracy of 90.3 % (28/31), comparable to its accuracy on the full crossNN cohort (87.2 %, 591/678), indicating that the subset is representative and not enriched for easily classified cases, while EpiDiP reached 80.6 % (25/31), corresponding to a Cohen's Kappa against the reference family of 0.89 and 0.79, respectively (**Supplementary Table 3**). The individual EpiDiP classification reports for these samples are provided in **Supplementary Data 1**.

As a proof of feasibility for cross platform use, MAGIBU was applied to whole genome bisulfite sequencing and Oxford Nanopore profiles of CNS tumors from the crossNN [10] nanopore dataset (GSE289246). Because these datasets did not span the full family spectrum, this analysis was framed as a feasibility assessment of platform applicability rather than as a complete validation. For the represented entities, family level predictions were recovered on both platforms: at 50,000 selected sites the per sample whole genome bisulfite sequencing analysis reached a family level Top 1 accuracy of 95.5 % and Top 3 of 100 %, and the pooled Nanopore pseudo samples reached a family level Top 1 accuracy of 100 %, obtained after aggregation of low coverage Nanopore samples sharing the same tumor family (**Supplementary Figure 2**).

We next examined the eight diagnostically challenging cases. The DNA methylation profile was successfully obtained using both classifiers at the time of the initial analysis in all cases except one (case 8). In this case, the Heidelberg CNS Tumor Methylation Classifier failed to assign a class (highest score 0.48 for control tissue), whereas Methylscape Analysis indicated a glial-glioneuronal tumor (score 0.86), favoring the ganglioglioma class (score 0.91). **Table 1** reports the DNA methylation profiling results for the entire cohort.

To quantify diagnostic performance, we calculated the Cohen’s Kappa coefficient against the consensus reference. MAGIBU demonstrated concordance in 6 out of 7 cases (85.7 %), considering the identification of the primary tumor class as a match. This resulted in a Cohen’s Kappa of 0.855, indicating almost perfect agreement. In contrast, EpiDiP yielded 2 concordant results out of 7 (28.6 %), with a Kappa value of 0.278, corresponding to fair agreement.

**Figure 2:**
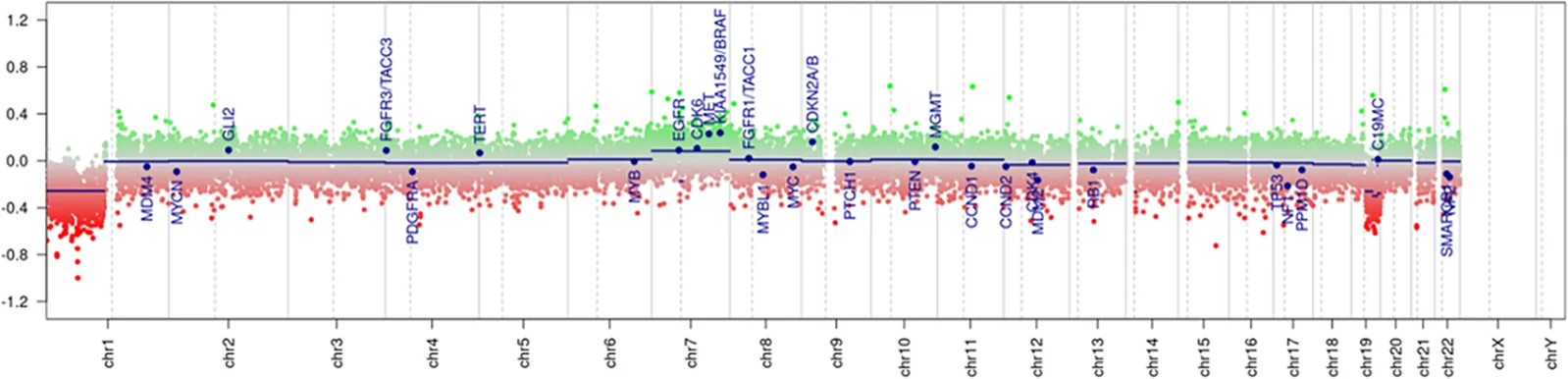
**Figure 2.** **Case 5.** **Methylscape Analysis.** Copy number variation (CNV) analysis identified the characteristic 1p-19q codeletion, supporting the diagnosis of DLGNT. Chromosomes 1–22 are depicted with the p-arm (left) and the q-arm (right), separated by a dotted line. Gains/ amplifications are represented as positive deviations from the baseline whereas losses are represented as negative deviations from the baseline. Relevant deviations from the baseline should be assessed using the horizontal dark blue line, which represents the average of several data points, rather than using the individual colored dots.

**Figure 3:**
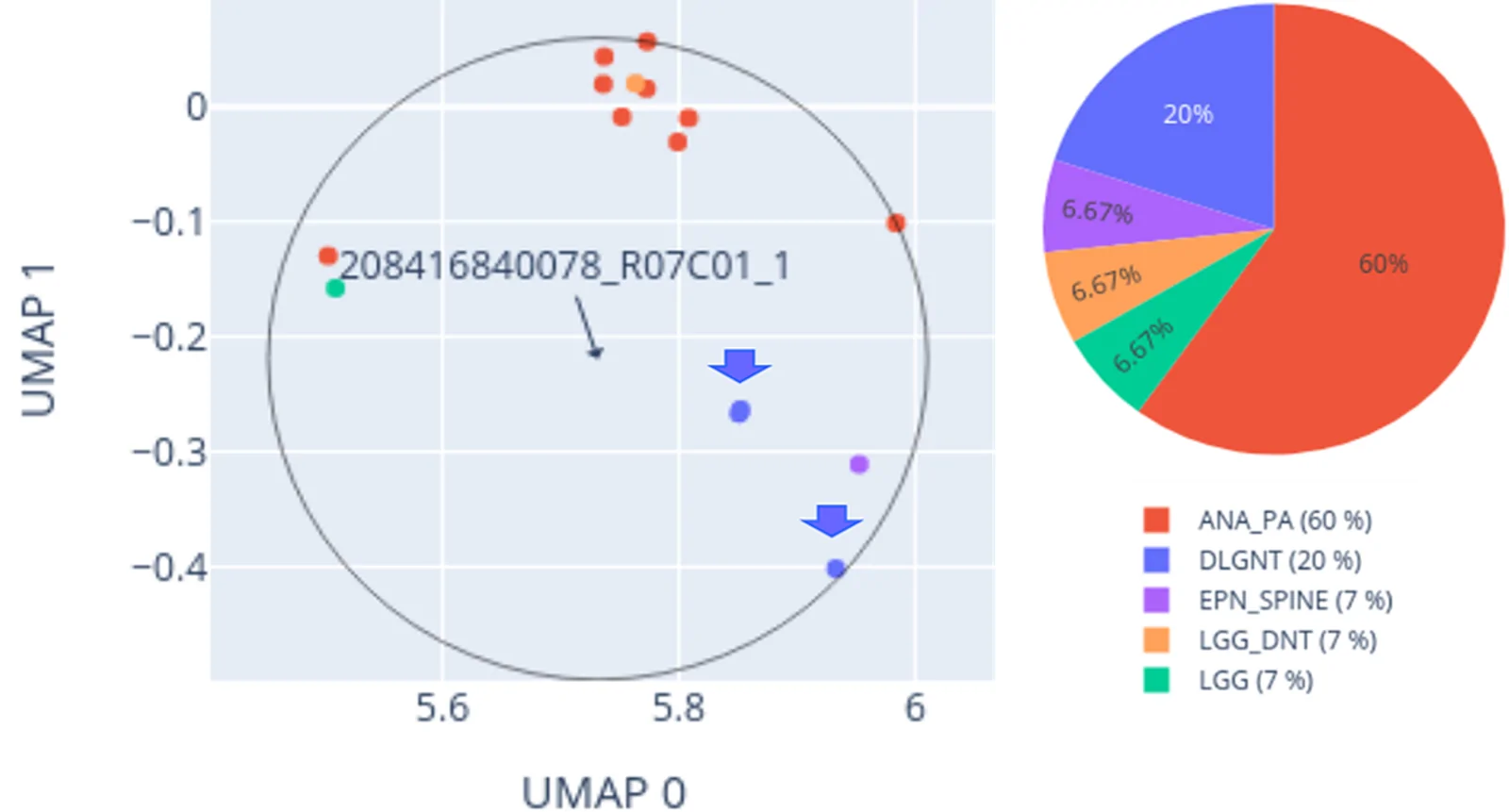
**Figure 3.** **Case 5 EpiDiP classification.** DLGNT was positioned among higher-grade categories in the methylation-based UMAP. Anaplastic pilocytic astrocytoma 60 % and DLGNT 20 % (blue dots and arrows). ANA_PA: anaplastic pilocytic astrocytoma; DLGNT: diffuse leptomeningeal glioneuronal tumor; EPN_SPINE: ependymomas spinal; LGG: low grade glioma; LGG_DNT: low grade glioma dysembryoplastic neuroepithelial tumor. Additional diagnostic classes are listed in Capper et al., **Supplementary Table 1** [9].

Within the EpiDiP discordances, four pilocytic astrocytomas and one diffuse leptomeningeal glioneuronal tumor (DLGNT) (**Table 1**, case 5; **Figure 2, Figure 3**) were positioned among higher-grade categories, specifically four anaplastic pilocytic astrocytomas and one high-grade glioma with pleomorphic and pseudopapillary features (HPAP).

**Figure 4:**
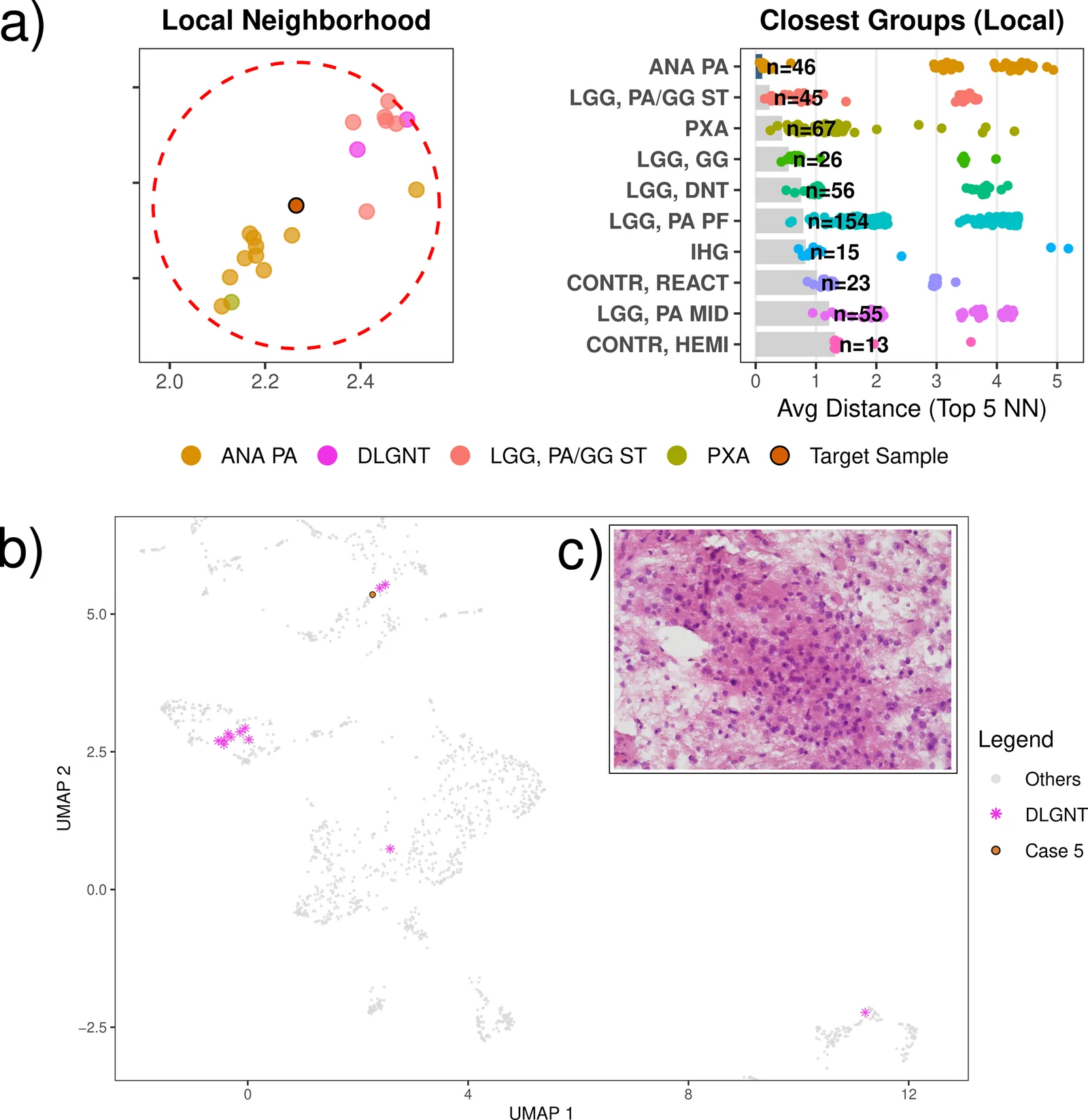
**Figure 4.** **Case 5.** **MAGIBU.** **a)** On the left, the local UMAP embedding visualizes the target sample (highlighted) and its spatially contiguous reference samples. On the right, bar plots quantify diagnostic affinity by calculating the mean Euclidean distance of the nearest neighbors. The bars indicate the methylation classes topologically closest (on average) to Case 5, providing a classification estimate based on epigenetic profile similarity; the individual points indicate the specific distances for each reference sample within the same class. **b)** The specific projection map visualizes the twelve DLGNT samples (magenta asterisks) included in the dataset, allowing evaluation of their spatial relationship to our target sample (orange point) within the global reference cohort (gray). Two DLGNT cases clustered closely with our sample, eight formed a distinct cluster, and two remained isolated. ANA PA: anaplastic pilocytic astrocytoma; DLGNT: diffuse leptomeningeal glioneuronal tumor; LGG, PA/GG ST: low grade glioma subclass hemispheric pilocytic astrocytoma and ganglioglioma; PXA: pleomorphic xanthoatrocytoma. For further successive diagnostic classes see Capper et al. **Supplementary Table 1** [9]. **c)** On microscopic examination, the tumor was composed of monomorphic, medium-sized cells with round nuclei and eosinophilic cytoplasm (Hematoxylin-Eosin, 20×).

Among the two MAGIBU discrepant cases, one represented a major misinterpretation: the DLGNT was positioned close to anaplastic pilocytic astrocytomas (**Table 1**, case 5; **Figure 2, Figure 3, Figure 4**). The second case reflected a minor imprecision rather than a true misinterpretation: an infratentorial FGFR1-altered pilocytic astrocytoma (**Table 1**, case 1) was positioned close to other infratentorial pilocytic astrocytomas without recognition of the specific molecular subtype.

**Figure 5:**
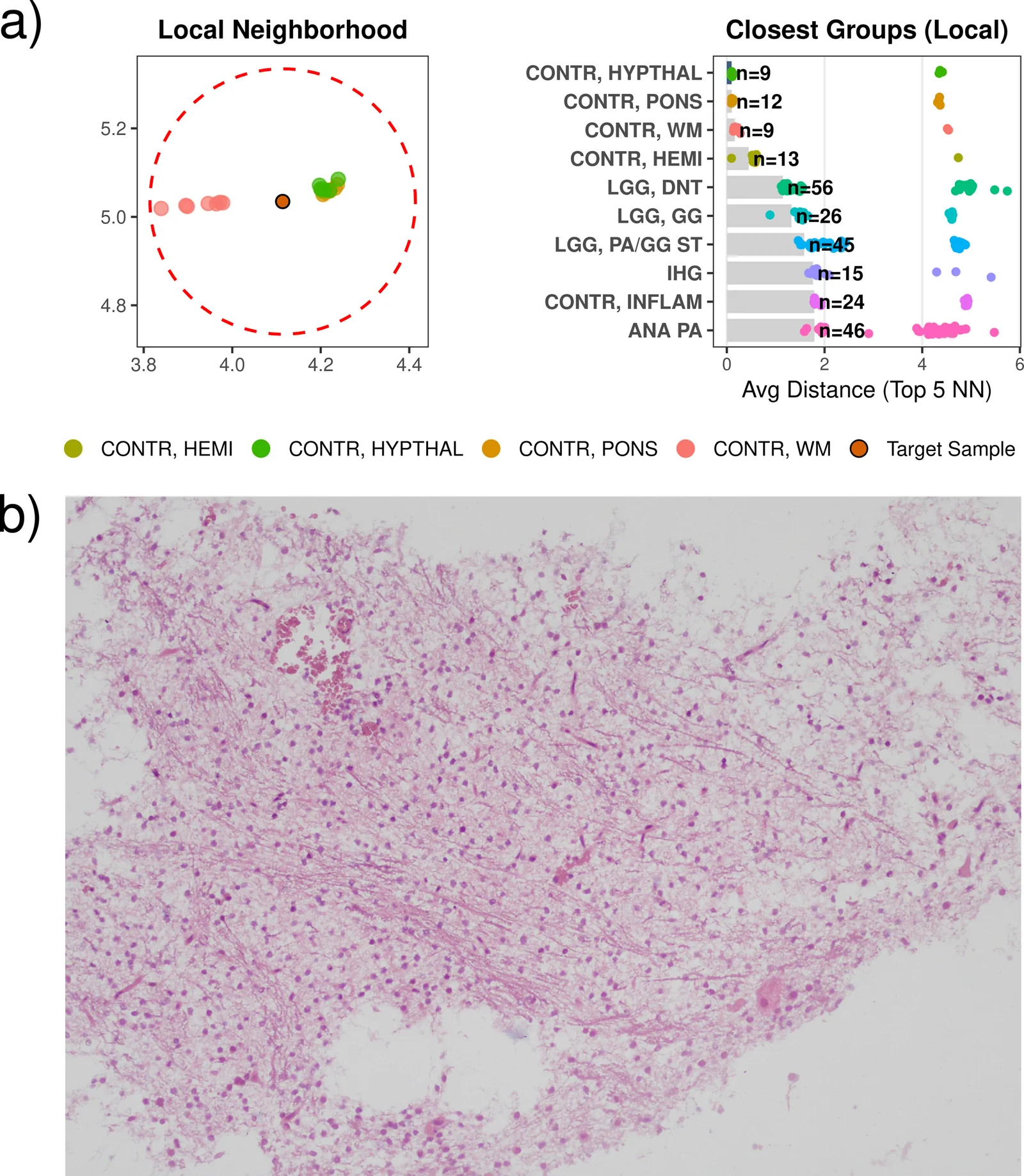
**Figure 5.** **Case 8.** **a)** The left panel shows the local UMAP neighborhood of Case 8 (highlighted), together with the reference samples that surround it in the embedding. The right panel summarizes diagnostic affinity as the mean Euclidean distance between Case 8 and the nearest reference samples of each methylation class: shorter mean distances denote greater epigenetic similarity, so the ranked bars provide a distance based classification estimate, while the individual points report the per sample distances within each class. CONTR, HYPTHAL: control hypothalamus; CONTR, PONS: control pons; CONTR, WM: control white matter. The full set of diagnostic classes is reported in Capper et al., **Supplementary Table 1** [9]. **b)** On microscopic examination, the tumor was composed of monomorphic, medium-sized cells with round nuclei and eosinophilic cytoplasm (Hematoxylin-Eosin, 20×).

Case 8 (**Table 1**), for which only Methylscape Analysis provided a definitive classification, was identified by EpiDiP as most closely related to HPAP. In contrast, MAGIBU positioned this case near non-neoplastic control tissues (**Table 1**, case 8; **Figure 5**), consistent with the Heidelberg CNS Tumor Methylation classifier ''no match'' with a maximum similarity score 0.48 for control tissue.

The complete set of MAGIBU results is provided in **Supplementary Figure 9** and **Table 1**. Per case nearest reference classes are listed in **Supplementary Table 4**.

Finally, we characterized the computational performance of MAGIBU, distinguishing the cost of classifying a new sample from the one time cost of building a reference model. Classifying a new sample reuses the stored projection and the frozen reference landscape without any retraining and is therefore effectively instantaneous on a standard desktop computer; it does not even require building a model locally, since each MAGIBU model is a standalone, shareable artifact that can be reused directly, and the CNS model used in this study is openly available from our GitHub repository (see Code and Data Availability). Model building, by contrast, is more demanding but is performed only once per reference: peak memory (approximately 82 to 96 GB) and wall clock time (approximately 7 to 13 hours) remained largely stable across the number of selected CpG sites and the number of allocated CPU cores (**Supplementary Figure 4**), because the dominant stages, namely loading the full reference matrix and computing the per probe variance before feature selection, are I/O bound and single threaded, whereas only the UMAP step is multithreaded. Peak memory scaled approximately linearly with the number of reference cases (from about 10 GB at 500 to about 73 GB at 3,905), while wall clock time grew faster than linearly (**Supplementary Figure 5**); this analysis isolates the sample count dimension and is intended as a demonstrative estimate. These computational costs are therefore incurred only once, at the construction of the reference model, and not at classification time.

## Discussion

In modern neuropathology, morphology alone may be insufficient to establish a definitive diagnosis, particularly in small stereotactic biopsies or in tumors with overlapping histological features [13,14,15]. The increasing complexity of the WHO classification of tumors of the CNS [2], which now includes numerous molecularly defined entities, further underscores the limitations of relying solely on traditional staining and immunohistochemistry. Comprehensive molecular profiling can therefore support diagnostic assessment and guide clinical management, as specific genetic and epigenetic alterations provide insights into prognosis and therapeutic strategies [16,17].

DNA methylation profiling has emerged as a key diagnostic tool in neuro-oncology. These epigenetic signatures remain stable throughout oncogenic transformation, providing a molecular fingerprint that reflects the tumor’s cell of origin [9,18,19]. This stability ensures that the diagnostic signal remains detectable even when morphological features are ambiguous or altered by secondary changes. Widely utilized tools include the Heidelberg CNS Tumor Methylation Classifier, which employs a Random Forest model to make probabilistic class assignments, and Methylscape Analysis, which uses a visualization-oriented, embedding-based approach to explore epigenetic similarity in reduced-dimensional space.

Our study included eight cases with inconclusive institutional and central review diagnoses, providing a context to evaluate MAGIBU in integrative CNS tumor classification and allowing comparison with EpiDiP. Unlike supervised classifiers such as the Heidelberg CNS Tumor Methylation Classifier, which assign categorical labels, neither EpiDiP nor MAGIBU provides a single, definitive diagnostic classification. Instead, both generate quantitative measures of epigenetic similarity and positional information within reference methylation spaces to support integrative interpretation [7,9,20,21].

EpiDiP provides an unsupervised framework integrating DNA methylation and CNV data, enabling visualization of individual tumors within a UMAP-based reference epigenomic landscape [21]. In our series, several low-grade tumors mapped in close proximity to higher-grade categories within the embedding space, without necessarily reflecting true biological relatedness. These observations highlight the need for caution when interpreting spatial relationships generated by dimensionality-reduction approaches, particularly in diagnostically ambiguous tumors or in entities underrepresented within reference cohorts.

The MAGIBU framework facilitates the integration of diverse genomic data types, overcoming the platform-specific biases and reproducibility issues inherent in dynamic embedding approaches. By leveraging a fixed- reference projection strategy, the CNS model maps prospective cases onto a static UMAP manifold constructed from 3,905 validated reference tumors. Rather than enforcing rigid categorical assignment, MAGIBU quantifies epigenetic similarity by calculating the mean Euclidean distance to the nearest local neighbors of each established methylation class. This metric yields a ranked differential diagnosis that captures the continuous nature of tumor methylation profiles, offering a transparent and quantitative description of a sample's relative position within the global reference landscape. In our series, MAGIBU results aligned with the reference classifications for most evaluated cases. One DLGNT (**Table 1, case 5**) was positioned closer to the anaplastic pilocytic astrocytomas. This finding likely reflects the limited representation of DLGNT within the reference cohort, which included only twelve cases. In the UMAP space (**Figure 4b**), two DLGNT cases clustered closely with our sample, eight formed a distinct cluster, and two remained isolated, underscoring the heterogeneous spatial distribution of this entity within the dataset and possibly indicating a degree of molecular heterogeneity that warrants further investigation. Notably, in our DLGNT case, CNV analysis by both Heidelberg CNS Tumor Methylation Classifier and Methylscape Analysis demonstrated a 1p/19q codeletion, consistent with the characteristic molecular profile of DLGNT (**Figure 2**).

For the recently defined WHO entity pilocytic astrocytoma, infratentorial, FGFR1-altered, MAGIBU correctly recognized the tumor as pilocytic astrocytoma, infratentorial but did not assign the specific molecular subtype, likely due to the absence of this recently recognized entity as a distinct class in the reference dataset. This entity is predominantly pediatric, typically arising in the cerebellum or brainstem, represents a small subset of pilocytic astrocytomas, and is characterized by FGFR1 alterations, which are uncommon but increasingly reported in molecular studies [22].

The comparison between MAGIBU and EpiDiP should be interpreted considering a structural difference between the two tools: they are built on distinct reference cohorts that differ in composition, size and versioning. In particular, EpiDiP relies on a substantially larger and pan-cancer reference, whereas the MAGIBU model used here is built on the comparatively smaller, CNS-restricted Capper reference (3,905 samples). The fact that MAGIBU achieved comparable or higher accuracy on this CNS subset despite drawing on a considerably smaller reference suggests that its projection and distance-based classification are at least as effective per reference sample, and points to the efficiency of the underlying algorithm rather than to an advantage conferred by reference size a factor that may in any case vary over time as the underlying reference cohorts evolve. The differences reported here nonetheless reflect the combined contribution of reference data composition, feature selection and the underlying classification algorithm. Finally, because EpiDiP must be queried manually through its web portal, this head-to-head comparison was performed on a curated subset rather than on the entire cohort; a fully unbiased comparison would require running EpiDiP on the complete validation set, and the figures reported here should therefore be regarded as indicative.

A further consideration is that EpiDiP is a pan-cancer classifier designed to position tumors across the entire neoplastic spectrum, whereas it was benchmarked here against a comparatively narrow set of CNS entities. A direct numerical comparison is therefore inherently asymmetric, because a fully balanced evaluation would also require tumor types outside the CNS, such as metastatic carcinomas and soft tissue tumors. This scope mismatch partly explains the higher discordance of EpiDiP within the epigenetically related domain of lower grade glial and glioneuronal tumors, where subtle distinctions are intrinsically harder for a tool that is not focused on this compartment.

A related limitation concerns tumor entities that are absent or underrepresented in the public reference used by MAGIBU. The current model is built on the publicly available Capper reference of CNS methylation classes, and some rare or recently defined entities are not captured: for example, high grade astrocytoma with piloid features is not represented as a distinct class and was not recovered in the broad CNS benchmark (0 of 3 cases; **Supplementary Table 2**), and high grade glioma subclasses that are defined only in classifiers backed by unpublished reference series, such as HGG_E and HGG_F in Methylscape Analysis, are likewise outside the public reference adopted here. This is an intrinsic constraint of any tool restricted to openly available data, and it underscores the need for a broader public release of reference methylation profiles. Because MAGIBU is designed to incorporate validated new samples back into its reference landscape, such entities can be added as soon as the corresponding public data become available. More broadly, because each MAGIBU model is stored as a standalone, shareable artifact built from a chosen reference, the framework lends itself to a distributed and incremental ecosystem: any group can construct a model on its own annotated cohort, including for rare or institution specific entities that are missing from public atlases, and release it openly. Reuse of such a model credits its originators through citation, providing a concrete academic incentive to share both data and models and, over time, to fill the gaps in publicly available reference data.

From a practical standpoint, these computational requirements do not limit the clinical applicability of MAGIBU: the memory and time reported above pertain only to the one time construction of a reference model, whereas routine projection of a new case is near instantaneous on a standard desktop and can rely on a pre-built, openly shared model, so that a diagnostic laboratory need never build a model itself.

Both EpiDiP and MAGIBU’s CNS models provide complementary insights into DNA methylation data. In this exploratory series, MAGIBU more frequently showed agreement with the consensus reference diagnosis for tumor type and histological grade, likely reflecting the contribution of quantitative proximity metrics integrated within the framework. By contrast, EpiDiP, which emphasizes visual interpretation of low-dimensional embedding, more often assigned calibrated confidence scores to low-grade tumors that were closer to higher-grade categories, highlighting the methodological differences between the two frameworks. As with other manifold-learning approaches, embedding-based visualization methods may be influenced by local manifold structure, dataset composition, and representation of rare entities. Tumors with intermediate, atypical, or heterogeneous methylation signatures, as well as rare entities represented by few reference samples, may occupy positions in the embedding space that do not closely correspond to a single established class, potentially affecting proximity-based rankings. These observations emphasize the importance of integrating methylation-based results with histopathological, molecular, and clinical data, and underscore the role of both platforms as decision-support tools rather than automated classifiers [7,18,20,21,23,24].

The eighth case further highlights the challenges of interpreting DNA methylation data in CNS tumors. While the Heidelberg CNS Tumor Methylation Classifier and MAGIBU indicated a non-tumoral profile, Methylscape Analysis assigned the sample to the ganglioglioma cluster with a high proximity score (0.91), and EpiDiP indicated closest similarity to HPAP. These divergent results illustrate how differences in algorithmic design, analytical strategy, and reference cohort composition may influence methylation-based interpretations in diagnostically ambiguous samples.

Supervised classifiers like Heidelberg CNS Tumor Methylation Classifier rely on trained predictive models with calibrated scoring thresholds, whereas Methylscape Analysis applies hierarchical scoring criteria to assign class and superfamily labels. Frameworks such as MAGIBU integrate quantitative similarity measures across different genomic platforms to support interpretive assessment rather than enforcing a single automated label. Visualization methods, including UMAP or t-SNE projections, can illustrate relationships among reference methylation profiles, but these serve primarily as interpretive aids. These observations highlight that quantitative proximity measures should be considered indicators of epigenetic similarity and decision-support tools rather than definitive classifiers. Taken together, these observations reinforce the central role of the pathologist, who integrates molecular, histological, and clinical information to establish the final diagnosis, emphasizing that definitive diagnosis in contemporary neuropathological practice remains an integrative, multidisciplinary process [25,26,27]. Our findings further illustrate both the potential and the current limitations of computational methylation-based approaches, particularly in rare or low-purity specimens, while providing an initial real-world evaluation of MAGIBU in diagnostically challenging CNS tumors.

In conclusion, the model generated through the MAGIBU framework demonstrated the feasibility of quantitative epigenetic proximity assessment for CNS tumor interpretation, showing agreement with consensus reference diagnoses in most evaluated cases. Compared with purely visualization-oriented embedding approaches, MAGIBU provides an additional quantitative framework for assessing epigenetic neighborhood relationships within the reference methylation landscape. Discordances for MAGIBU were mostly limited to rare or new entities, such as DLGNT and pilocytic astrocytoma, infratentorial, FGFR1-altered, reflecting their limited representation in the reference dataset. These findings support MAGIBU as a reliable decision-support tool for integrative CNS tumor assessment, with further performance gains expected as reference datasets expand. Crucially, the MAGIBU-based approach complements established classifiers by providing a standardized environment for creating quantitative models that guide integrative diagnostic assessment without replacing expert pathological judgment.

## Code and data availability

The MAGIBU framework, implemented in R, is available as an open-source repository on GitHub (https://github.com/GianlucaMattei/MAGIBU.git). The repository contains the complete source code for the training engine, projection module, and visualization tools, along with the pretrained CNS reference atlas. Detailed documentation, including tutorials for processing raw IDAT files, integrating heterogeneous input formats (Array, Nanopore), and reproducing the analysis figures, is provided to facilitate accessibility and reproducibility for the research community. For Nanopore analysis, the function ModkitResorter.sh from PoreMeth2 is mandatory for the preparation of the beta files (https://github.com/Lab-CoMBINE/PoreMeth2) [28]. The raw DNA methylation data (.idat files) generated for this study have been deposited in the NCBI Gene Expression Omnibus (GEO) and are accessible through accession number GSE319846. The raw input data for the head-to-head comparison with EpiDiP are publicly available (GSE289137), and the MAGIBU source code and reference model are openly available, so that both the MAGIBU and the EpiDiP classifications can be independently reproduced.

## Compliance with ethical standards

The Pediatric Ethics Committee of the Tuscany Region approved the study (PA_07/2025).

## Generative AI and AI-assisted technologies

The authors declare that they used LLM models to refine language, enhance clarity and coherence, and improve the overall readability of the manuscript.

## Contributions

Anna Maria Buccoliero and Chiara Caporalini performed the histopathological diagnosis. Mirko Scagnet, Rina Agushi, Federico Mussa and Lorenzo Genitori conducted the surgical excision of the tumor. Iacopo Sardi managed the patient’s oncological treatment. Gianluca Mattei, Vincenzo Yuto Civale, Alberto Magi and Laura Giunti carried out the molecular analyses.

## Conflict of interest statement

The authors declare no conflict of interest.

## Supplementary Material

Supplementary File 1. Supplementary figures 1–9 (PDF file, 2 MB)

Supplementary File 2. Supplementary tables 1–4 (xmlx file, 60 KB)

Supplementary File 3. Supplementary methods (PDF file, 0,3 MB)

Supplementary File 4. Supplementary data (PDF file, 17 MB)

## References

[R1] Sill M, Schrimpf D, Patel A, Sturm D, Jäger N, Sievers P, et al. (2026) Advancing CNS tumor diagnostics with expanded DNA methylation-based classification. Cancer Cell 44(2):340–354.e2. 10.1016/j.ccell.2025.11.002 41349541

[R2] WHO Classification of Tumours Editorial Board. (2021) WHO Classification of Tumours: Central Nervous System Tumours. 5th ed. Lyon: International Agency for Research on Cancer.

[R3] Drexler R, Brembach F, Sauvigny J, Ricklefs FL, Eckhardt A, Bode H, et al. (2024) Unclassifiable CNS tumors in DNA methylation-based classification: clinical challenges and prognostic impact. Acta Neuropathol Commun 12:1–9. 10.1186/s40478-024-01728-9 PMC10792852 38229158

[R4] Pratt D, Sahm F, Aldape K. (2021) DNA methylation profiling as a model for discovery and precision diagnostics in neuro oncology. Neuro Oncol 23 (Suppl 5):S16–S29. 10.1093/neuonc/noab143 PMC8561128 34725697

[R5] Schepke E, Löfgren M, Pietsch T, Olsson Bontell T, Kling T, Wenger A, et al. (2022) DNA methylation profiling improves routine diagnosis of paediatric central nervous system tumours: a prospective population based study. Neuropathol Appl Neurobiol 48(6):e12838. 10.1111/nan.12838 PMC9543790 35892159

[R6] Zhang W, Feng H, Wu H, Zheng X. (2017) Accounting for tumor purity improves cancer subtype classification from DNA methylation data. Bioinformatics 33(17):2651–2657. 10.1093/bioinformatics/btx303 PMC6410888 28472248

[R7] Al Sharie S, Sawaftah K, Qasim H, Al-Hussaini M. (2025) Methylation profiling in neuropathological tumors diagnosis: a comprehensive review. Front Oncol 15:1720458. 10.3389/fonc.2025.1720458 PMC12757286 41487568

[R8] Buccoliero AM, Giunti L, Scagnet M, Guidi M, Vergani D, Agushi R, et al. (2025) The contribution of methylation profiling in neuropathological diagnosis of central nervous system tumors in children, adolescent and young adults. Pathologica 117(5):475–485. 10.32074/1591-951X-1226 PMC12743366 41454766

[R9] Capper D, Jones DTW, Sill M, Hovestadt V, Schrimpf D, Sturm D, et al. (2018) DNA methylation-based classification of central nervous system tumours. Nature 555:469–474. 10.1038/nature26000 PMC6093218 29539639

[R10] Yuan D, Jugas R, Pokorna P, Sterba J et al. crossNN is an explainable framework for cross-platform DNA methylation-based classification of tumors. Nat Cancer 2025 Jul;6(7):1283–1294. 10.1038/s43018-025-00976-5 PMC12296554 40481322

[R11] Sturm D, Capper D, Andreiuolo F, Gessi M, Kölsche C, Reinhardt A, et al. (2023) Multiomic neuropathology improves diagnostic accuracy in pediatric neuro-oncology. Nat Med 29(4):917–926. 10.1038/s41591-023-02255-1 PMC10115638 36928815

[R12] Lilly JV, Rokita JL, Mason JL, Patton T, Stefankiewiz S, Higgins D, et al. (2023) The children's brain tumor network (CBTN) - accelerating research in pediatric central nervous system tumors through collaboration and open science. Neoplasia 35:100846. 10.1016/j.neo.2022.100846 PMC9641002 36335802

[R13] Sturm D, Orr BA, Toprak UH, Hovestadt V, Jones DTW, Capper D, et al. (2016) New brain tumor entities emerge from molecular classification of CNS-PNETs. Cell 164:1060–1072. 10.1016/j.cell.2016.01.015 PMC5139621 26919435

[R14] Viaene AN. (2023) Pediatric brain tumors: a neuropathologist's approach to the integrated diagnosis. Front Pediatr 11:1143363. 10.3389/fped.2023.1143363 PMC10030595 36969278

[R15] Horbinski C, Solomon DA, Lukas RV, Packer RJ, Brastianos P, Wen PY et al. (2025) Molecular testing for the World Health Organization classification of central nervous system tumors: a review. JAMA Oncol 11:317–328. 10.1001/jamaoncol.2024.5506 PMC12344474 39724142

[R16] Karimi S, Zuccato JA, Mamatjan Y, Mansouri S, Suppiah S, Nassiri F et al. (2019) The central nervous system tumor methylation classifier changes neuro-oncology practice for challenging brain tumor diagnoses and directly impacts patient care. Clin Epigenet 11:185. 10.1186/s13148-019-0766-2 PMC6896594 31806041

[R17] Kim M, Costello J. (2017) DNA methylation: an epigenetic mark of cellular memory. Exp Mol Med 49:e322. 10.1038/emm.2017.10 PMC6130213 28450738

[R18] Bertero L, Mangherini L, Ricci AA, Cassoni P, Sahm F. (2024) Molecular neuropathology: an essential and evolving toolbox for the diagnosis and clinical management of central nervous system tumors. Virchows Arch 484:181–194. 10.1007/s00428-023-03632-4 PMC10948579 37658995

[R19] Feinberg AP, Irizarry RA, Fradin D, Aryee MJ, Murakami P, Aspelund T, et al. (2010) Personalized epigenomic signatures that are stable over time and covary with body mass index. Sci Transl Med 2:49ra67. 10.1126/scitranslmed.3001262 PMC3137242 20844285

[R20] Moosa E, Alanany R, Sherif S, Ozer E, Dube S, Jabeen A, et al. (2026) Enhancing the accuracy of molecular classification of pediatric CNS tumors: a dual-classifier approach using DNA methylation profiling. Front Oncol 15:1701113. 10.3389/fonc.2025.1701113 PMC12916412 41726366

[R21] Hench J, Hultschig C, Brugger J, Mariani L, Guzman R, Soleman J, et al. (2024) EpiDiP/NanoDiP: a versatile unsupervised machine learning edge computing platform for epigenomic tumour diagnostics. Acta Neuropathol Commun 12:51. 10.1186/s40478-024-01759-2 PMC10993614 38576030

[R22] Lucas CG, Gupta R, Doo P, Lee JC, Cadwell CR, Ramani B, et al. (2020) Comprehensive analysis of diverse low-grade neuroepithelial tumors with FGFR1 alterations reveals a distinct molecular signature of rosette-forming glioneuronal tumor. Acta Neuropathol Commun 8:151. 10.1186/s40478-020-01027-z PMC7456392 32859279

[R23] Zhang YW, Zheng Y, Wang JZ, Lu XX, Wang Z, Chen LB, et al. (2014) Integrated analysis of DNA methylation and mRNA expression profiling reveals candidate genes associated with cisplatin resistance in non-small cell lung cancer. Epigenetics 9:896–909. 10.4161/epi.28601 PMC4065187 24699858

[R24] Bertogliat MJ, Morris-Blanco KC, Vemuganti R. (2019) Epigenetic mechanisms of neurodegenerative diseases and acute brain injury. Neurochem Int 133:104642. 10.1016/j.neuint.2019.104642 PMC8074401 31838024

[R25] Angerilli V, Galuppini F, Pagni F, Fusco N, Malapelle U, Fassan M, et al. (2021) The role of the pathologist in the next generation era of tumor molecular characterization. Diagnostics 11:339. 10.3390/diagnostics11020339 PMC7922586 33670699

[R26] Sharma S, George P, Waddell N. (2021) Precision diagnostics: integration of tissue pathology and genomics in cancer. Pathology 53(7):809–817. 10.1016/j.pathol.2021.08.003 34635323

[R27] AACR Pathology Task Force. (2022) Pathology: hub and integrator of modern, multidisciplinary precision oncology. Clin Cancer Res 28:265–270. 10.1158/1078-0432.CCR-21-1206 34893516

[R28] Mattei G, Baragli M, Gega B, Mingrino A, Chieca M, Ducci T et al. (2025) PoreMeth2 for decoding the evolution of methylome alterations with nanopore sequencing. Genome Res 35(11):2501–2512. 10.1101/gr.280259.124 PMC12581943 41115805

