# Supplementary material for: A MAGIBU-based model for pediatric and juvenile CNS tumors: an in-house epigenetic decision-support framework compared with online DNA methylation classifiers
Source: Free Neuropathol. 2026 Aug 24;7:21. doi: 10.17879/freeneuropathology-2026-9648 (PMC13532279; doi:10.17879/freeneuropathology-2026-9648)
Supplement: Supplementary file 1 [file freeneuropathol-07-21-9648-s1.pdf]

Supplementary material

Supplementary Figure 1

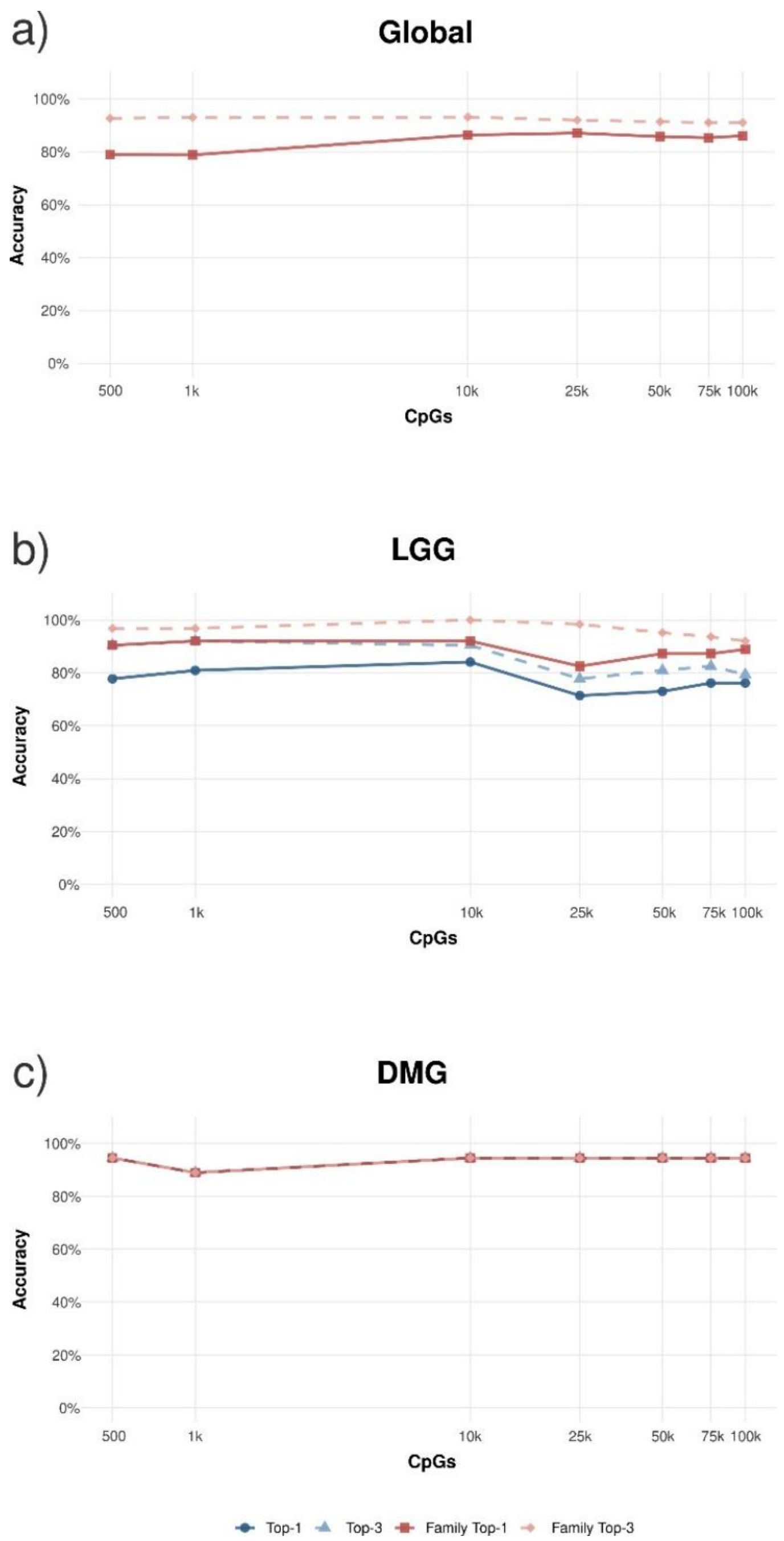

Supplementary Figure 1. MAGIBU classification accuracy as a function of model size. Family level accuracy of MAGIBU on the crossNN [10] array validation cohort (GSE289137, n = 678 EPICv1 and EPICv2 CNS samples), across seven PCA and UMAP models trained with increasing numbers of CpG sites (n_cpg = 500, 1,000, 10,000, 25,000, 50,000, 75,000, 100,000). **a)** Global CNS performance: Top 1 = ground truth family in the first prediction; Top 3 = ground truth family among the first three predictions. **b)** Low grade glioma cohort (LGG: PA, LGNT, PXA, SEGA; n = 63); subclass level metrics use canonical codes (PA, GG, CN, RGNT, DNT, DLGNT, PXA, SEGA). **c)** Diffuse midline glioma cohort (DMG, n = 18, all H3 K27 altered); a single subclass (K27) is annotated, so subclass and family metrics overlap. Samples annotated as non-CNS were excluded.

Copyright: © 2026 The author(s). This is an open access article distributed under the terms of the Creative Commons Attribution 4.0 International License (https://creativecommons.org/licenses/by/4.0/), which permits unrestricted use, distribution, and reproduction in any medium, provided the original author and source are credited, a link to the Creative Commons license is provided, and any changes are indicated. The Creative Commons Public Domain Dedication waiver (https://creativecommons.org/publicdomain/zero/1.0/) applies to the data made available in this article, unless otherwise stated.

Supplementary Figure 2

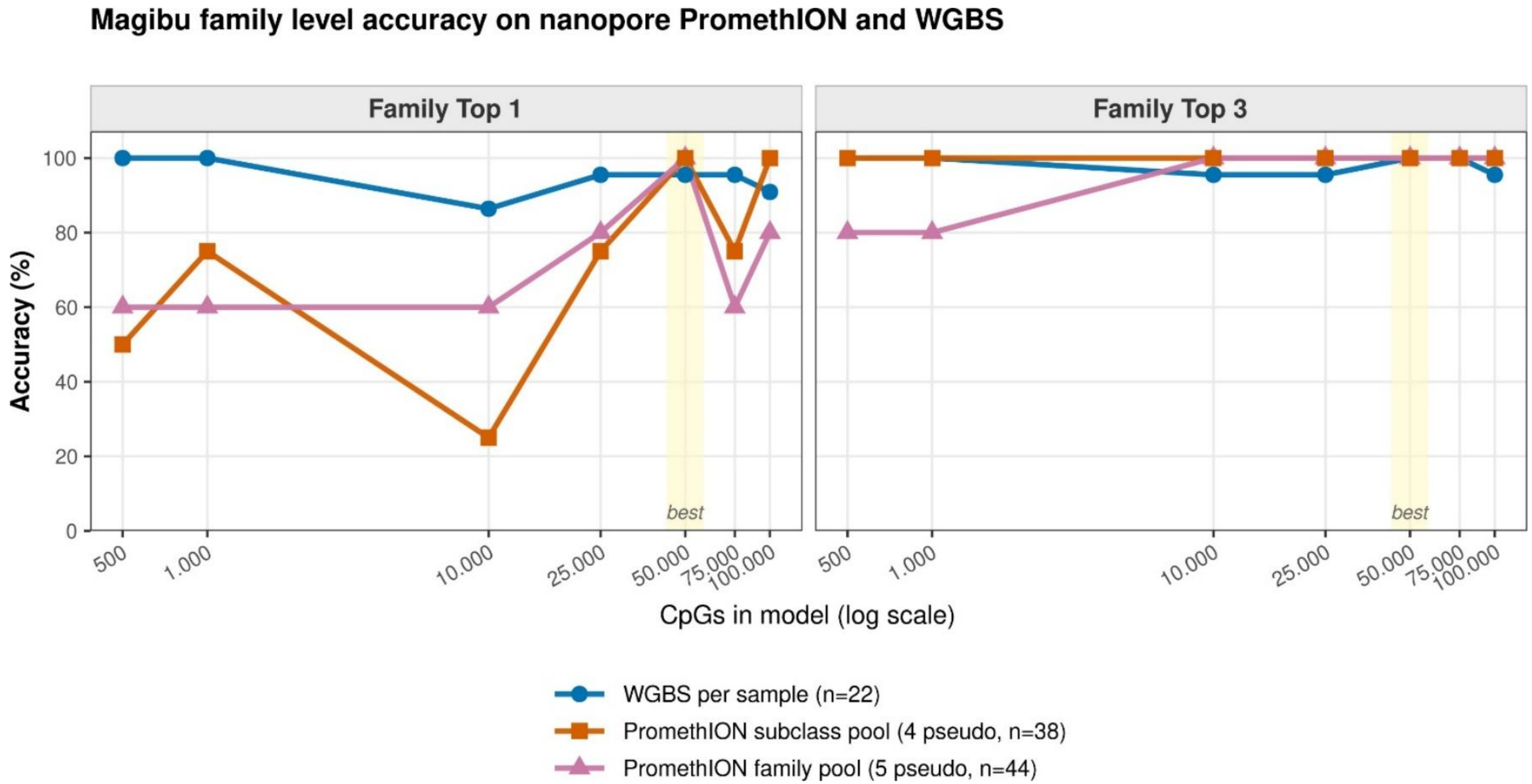

Supplementary Figure 2. Family level classification accuracy of MAGIBU on Nanopore PromethION and bisulfite WGBS validation data across seven CpG models that differ by the number of CpGs. Three independent conditions are shown for samples from GSE289246: WGBS NovaSeq 6000 samples projected individually (blue circles, n = 22, GPL24676), PromethION samples aggregated into pseudo samples by exact WHO subclass (orange squares, 4 pools combining 38 GPL26167 samples), and PromethION samples aggregated by canonical family (pink triangles, 5 pools combining 44 GPL26167 samples). The left panel reports Top 1 family accuracy (the nearest training subclass must collapse to the correct family); the right panel reports Top 3 accuracy (correct family appearing in any of the three nearest training subclasses). The yellow band marks the best performing model range (n_cpg approximately 50,000) at which all PromethION pools and at least 95 % of WGBS samples are classified correctly.

freeneuropathology.org

Copyright: © 2026 The author(s). This is an open access article distributed under the terms of the Creative Commons Attribution 4.0 International License (https://creativecommons.org/licenses/by/4.0/), which permits unrestricted use, distribution, and reproduction in any medium, provided the original author and source are credited, a link to the Creative Commons license is provided, and any changes are indicated. The Creative Commons Public Domain Dedication waiver (https://creativecommons.org/publicdomain/zero/1.0/) applies to the data made available in this article, unless otherwise stated.

Supplementary Figure 3

Initial Cohorts

Source	Samples
Sturm_panCNS	524
Cbtn	349
Sturm_pnet	73
Internal	30
Total Samples	976

Published diagnosis was NOT in LGG-family nor DMG K27

Source	Samples	Diagnoses
Sturm_panCNS	109	GBM (67)<br>Anaplastic Astrocytoma (41)<br>HGG NOS (1)
Cbtn	78	GBM (36)<br>HGG (27)<br>Anaplastic Astrocytoma (13)<br>Supratentorial PNET (2)
Sturm_pnet	43	Unclassificable (22)<br>HGG RTK/MYCN (7)<br>CNS-HGNET MN1/BCOR (5)<br>CNS-NB-FOXR2, Ctrl (2)<br>DIG, Ependymoma, Ewing Sarc., MB, Pineal (1)
Internal	4	HGG NOS (3)<br>GBM (1)
Total Samples	234	

Elegible Cohorts

Source	LGG-family	DMG	Total
Sturm_panCNS	380	35	415
Cbtn	271	24	271
Sturm_pnet	30	20	30
Internal	26	1	26
Total Samples	662	80	742

Supplementary Figure 3. Sample selection and exclusion flow for the focused LGG and DMG validation. Starting from 976 cases across the four cohorts (sturm_panCNS, GSE215240; CBTN; sturm_pnet, GSE73801; in-house), cases whose published diagnosis fell outside the LGG family or DMG were excluded, leaving 742 eligible cases; after restricting to samples whose ground truth and MAGIBU prediction were both either LGG or DMG, 670 cases were analyzable. The diagram reports the number of samples retained and excluded at each filtering step.

Copyright: © 2026 The author(s). This is an open access article distributed under the terms of the Creative Commons Attribution 4.0 International License (https://creativecommons.org/licenses/by/4.0/), which permits unrestricted use, distribution, and reproduction in any medium, provided the original author and source are credited, a link to the Creative Commons license is provided, and any changes are indicated. The Creative Commons Public Domain Dedication waiver (https://creativecommons.org/publicdomain/zero/1.0/) applies to the data made available in this article, unless otherwise stated.

Supplementary Figure 4

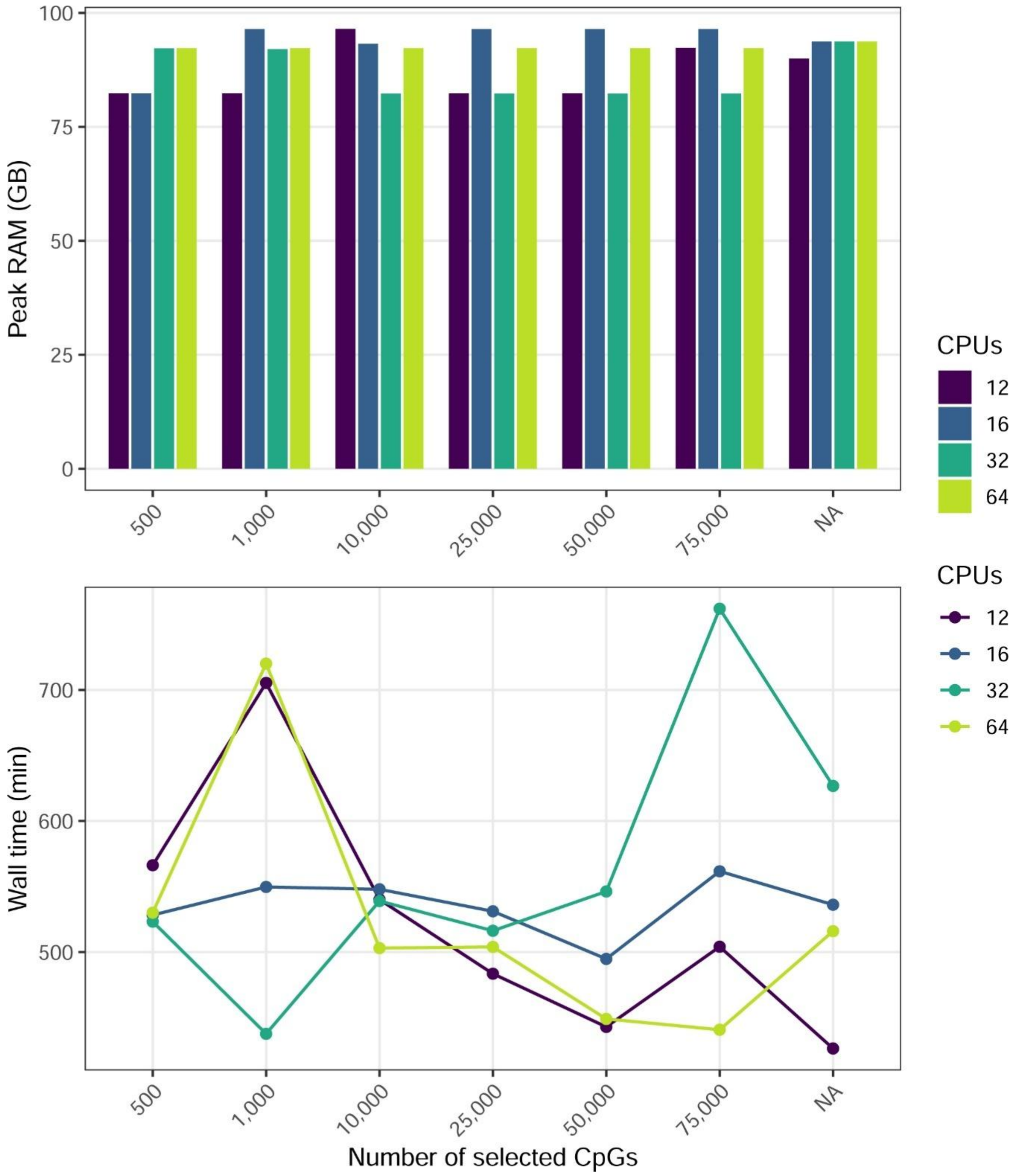

Supplementary Figure 4. Computational performance of the MAGIBU reference model build across varying CPU counts and numbers of selected CpGs. Benchmarks were run on the full reference matrix (485,512 CpGs by 3,905 samples) for seven CpG selection sizes (500 to 100,000) and four CPU configurations (12, 16, 32, 64 cores). (Top) Peak memory usage (GB), shown as grouped bars by CPU count, remains essentially constant (about 84 to 96 GB) across all CpG sizes and core counts, because the complete reference matrix is loaded into memory before CpG selection. (Bottom) Wall clock time (minutes) shows no consistent reduction with increasing CPU count and varies non mono-tonically (range 426 to 762 min). This reflects that the dominant cost is the I/O bound parallel loading of 3,905 per sample files rather than a CPU bound computation; runtime is therefore largely insensitive to both the number of cores and the number of selected CpGs. Each configuration was run once (n = 1).

freeneuropathology.org

Copyright: © 2026 The author(s). This is an open access article distributed under the terms of the Creative Commons Attribution 4.0 International License (https://creativecommons.org/licenses/by/4.0/), which permits unrestricted use, distribution, and reproduction in any medium, provided the original author and source are credited, a link to the Creative Commons license is provided, and any changes are indicated. The Creative Commons Public Domain Dedication waiver (https://creativecommons.org/publicdomain/zero/1.0/) applies to the data made available in this article, unless otherwise stated.

Supplementary Figure 5

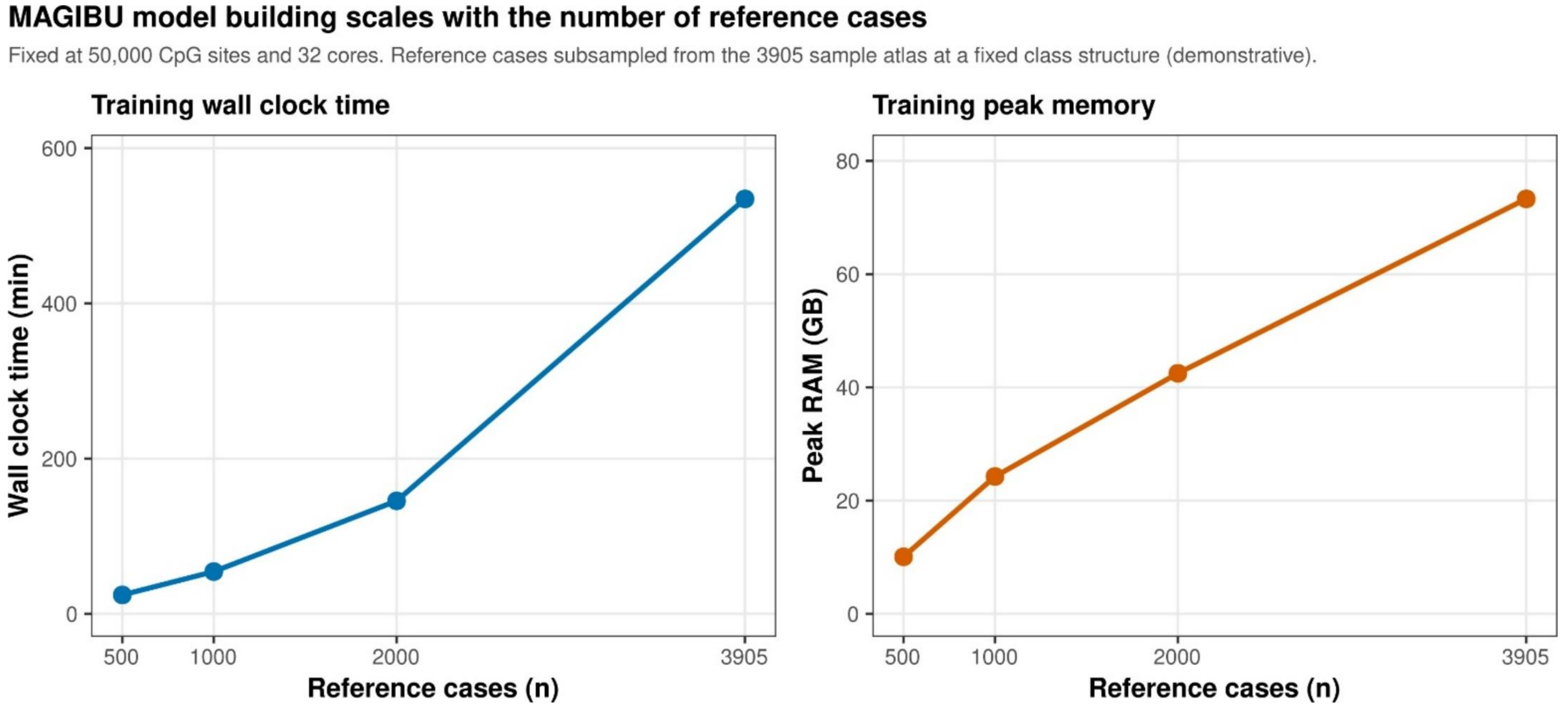

Supplementary Figure 5. Scaling of MAGIBU model building with the number of reference cases. Models were trained on random subsets of 500, 1000, 2000 and 3905 reference cases at 50,000 CpG sites and 32 cores. (Left) Wall clock time and (right) peak memory as a function of the number of reference cases. Peak memory grows approximately linearly with the number of reference cases (about 10 to 73 GB), while wall clock time grows faster than linearly. Because in a real reference the number of represented classes and the number of samples increase together, the subsets were drawn at a fixed class structure; this benchmark therefore isolates the sample count dimension and is intended as a demonstrative estimate.

Copyright: © 2026 The author(s). This is an open access article distributed under the terms of the Creative Commons Attribution 4.0 International License (https://creativecommons.org/licenses/by/4.0/), which permits unrestricted use, distribution, and reproduction in any medium, provided the original author and source are credited, a link to the Creative Commons license is provided, and any changes are indicated. The Creative Commons Public Domain Dedication waiver (https://creativecommons.org/publicdomain/zero/1.0/) applies to the data made available in this article, unless otherwise stated.

Supplementary Figure 6

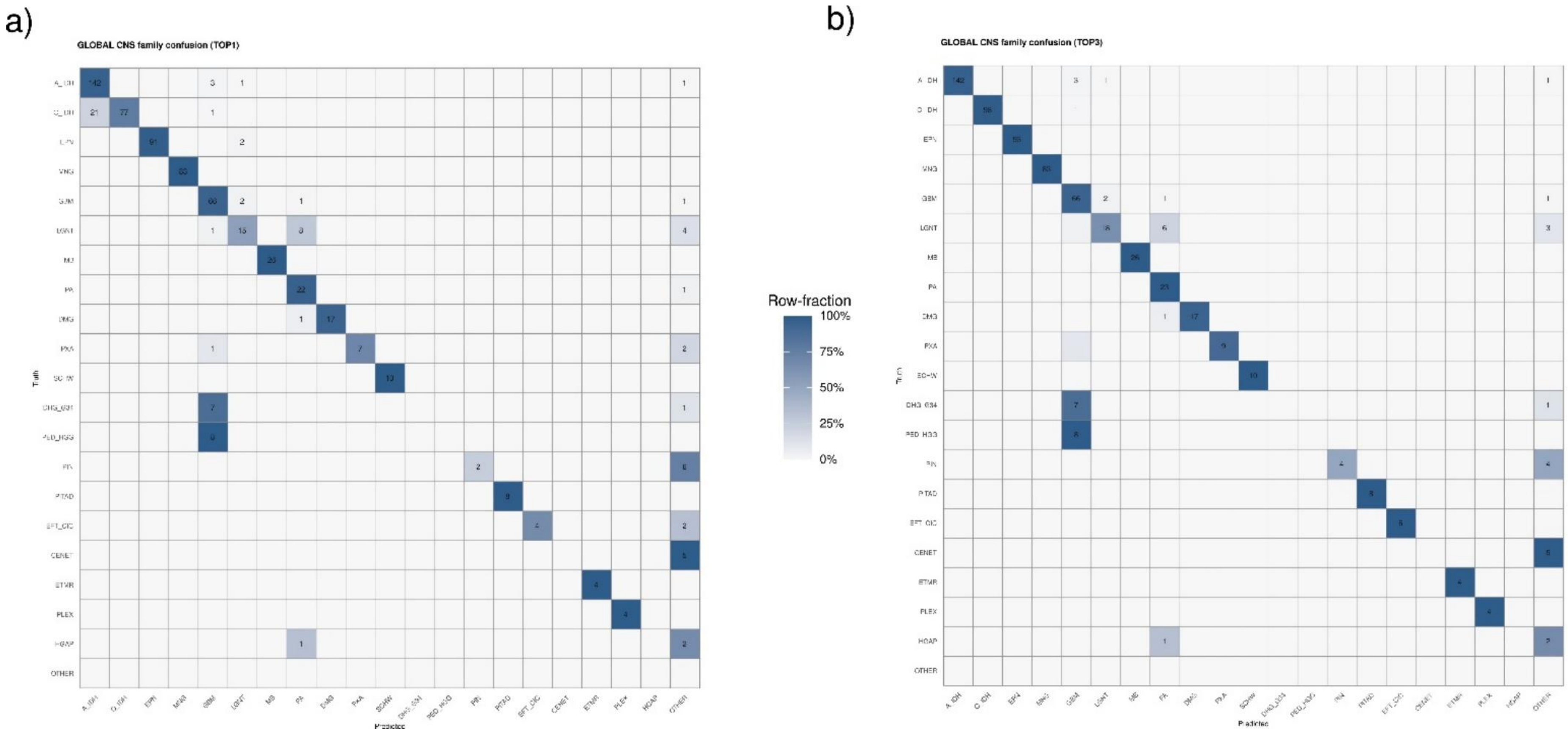

Supplementary Figure 6. Global confusion matrices of MAGIBU family level predictions on the CNS array validation cohort (GSE289137, n = 678). **a)** Top 1 (ground truth family in the first prediction) and **b)** Top 3 (ground truth family among the first three predictions). Rows show the GEO ground truth family, columns show the predicted family; cells report the count of samples. Truth labels are restricted to the 20 most frequent families in the cohort; predictions that fall outside these 20 families are pooled into the OTHER column to keep the matrix readable. The OTHER column captures misclassifications towards rare families absent from the top 20.

Copyright: © 2026 The author(s). This is an open access article distributed under the terms of the Creative Commons Attribution 4.0 International License (https://creativecommons.org/licenses/by/4.0/), which permits unrestricted use, distribution, and reproduction in any medium, provided the original author and source are credited, a link to the Creative Commons license is provided, and any changes are indicated. The Creative Commons Public Domain Dedication waiver (https://creativecommons.org/publicdomain/zero/1.0/) applies to the data made available in this article, unless otherwise stated.

Supplementary Figure 7

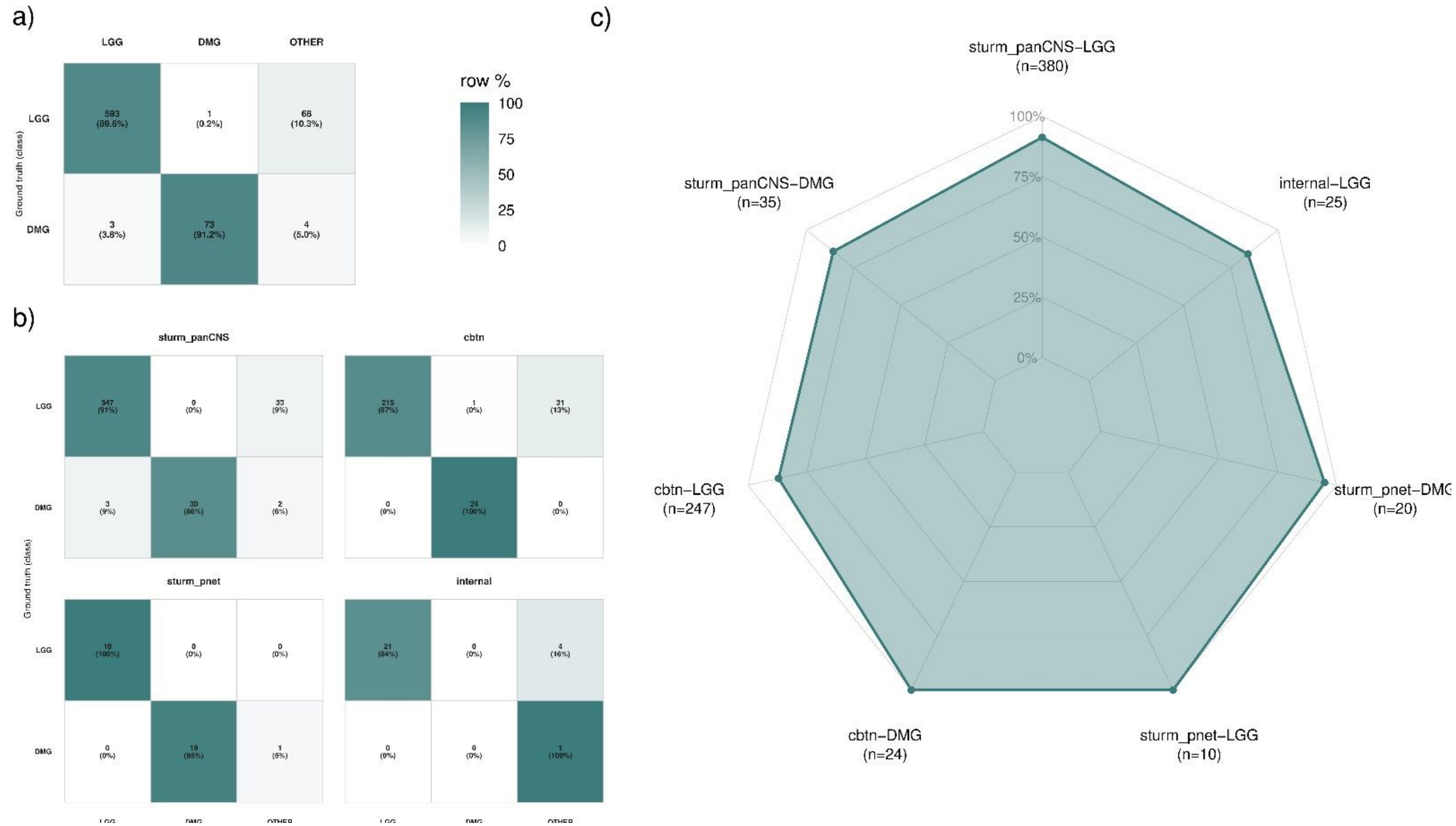

Supplementary Figure 7. a) Global class-level confusion matrix. Confusion matrix for MAGIBU class-level predictions on the pooled validation cohorts (n = 670). Rows indicate the ground-truth class and columns indicate the MAGIBU predicted class. Cell color encodes the row-normalized proportion (recall along the diagonal); cell labels report absolute counts. Samples whose ground truth or MAGIBU prediction fell outside the LGG/DMG dichotomy were excluded from both sides. Supplementary Figure **b)** Per-cohort class-level confusion matrices. Cohort-stratified confusion matrices for MAGIBU class-level predictions, faceted by validation cohort (sturm_panCNS, GSE215240; cbtn, Children's Brain Tumor Network; sturm_pnet, GSE73801; in-house). Encoding as in Supplementary Figure **a)** The in-house panel lacks a DMG row because no DMG sample passed the inclusion filter. **c)** Radar plot of class-level recall by cohort and class. Radar plot of LGG and DMG recall, with vertices defined by each combination of cohort and class passing the n ≥ 3 filter (seven vertices). Each vertex reports the recall (%) of one ground-truth class within one cohort; the closed polygon traces the overall macro-performance (mean = 98.6 %). Concentric grid lines mark recall increments of 20 percentage points, with the outer ring at 100 %.

Copyright: © 2026 The author(s). This is an open access article distributed under the terms of the Creative Commons Attribution 4.0 International License (https://creativecommons.org/licenses/by/4.0/), which permits unrestricted use, distribution, and reproduction in any medium, provided the original author and source are credited, a link to the Creative Commons license is provided, and any changes are indicated. The Creative Commons Public Domain Dedication waiver (https://creativecommons.org/publicdomain/zero/1.0/) applies to the data made available in this article, unless otherwise stated.

Supplementary Figure 8

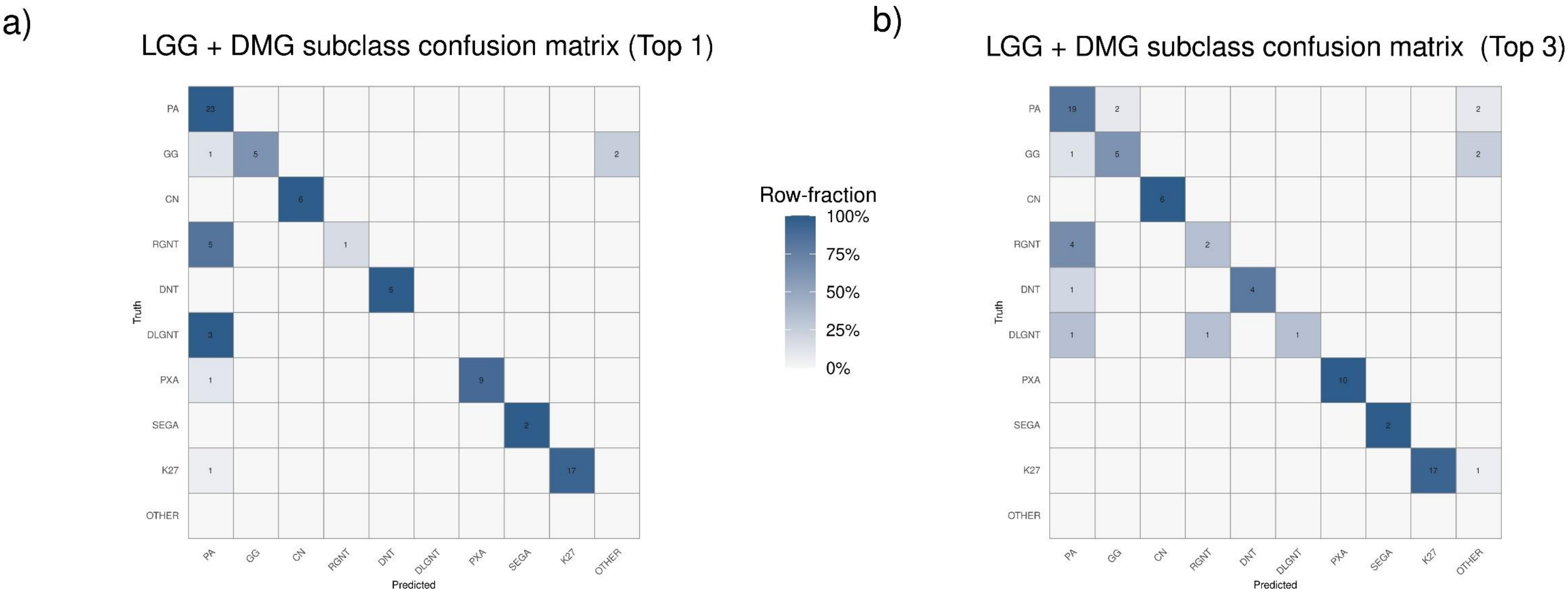

Supplementary Figure 8. Subclass level confusion matrices combining the low grade glioma (n = 63) and diffuse midline glioma (n = 18) cohorts, at the best performing model for each cohort, for **a)** Top 1 and **b)** Top 3 predictions. Rows are ground truth subclasses (canonical codes: PA, GG, CN, RGNT, DNT, DLGNT, PXA, SEGA for LGG; K27 for DMG H3 K27 altered). Columns are MAGIBU predictions canonicalized to the same vocabulary; predictions outside the LGG/DMG vocabulary are grouped under OTHER. Tile color encodes the row fraction (per truth recall); cell numbers show absolute sample counts.

Copyright: © 2026 The author(s). This is an open access article distributed under the terms of the Creative Commons Attribution 4.0 International License (https://creativecommons.org/licenses/by/4.0/), which permits unrestricted use, distribution, and reproduction in any medium, provided the original author and source are credited, a link to the Creative Commons license is provided, and any changes are indicated. The Creative Commons Public Domain Dedication waiver (https://creativecommons.org/publicdomain/zero/1.0/) applies to the data made available in this article, unless otherwise stated.

DOI: https://doi.org/10.17879/freeneuropathology-2026-9648

Supplementary Figure 9

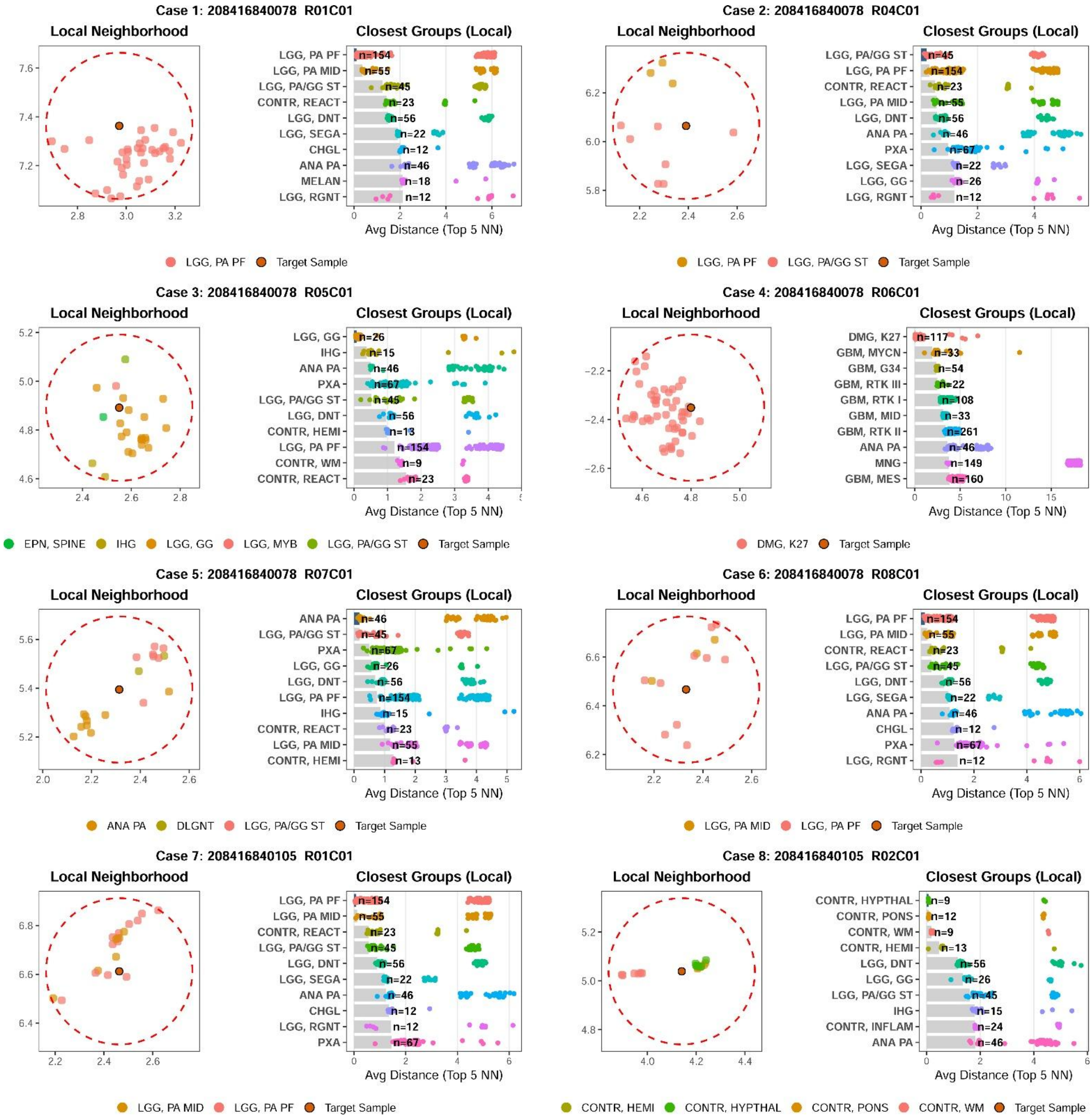

Supplementary Figure 9. Global outcomes derived from the MAGIBU model, illustrating the results for all 8 experimental samples. LGG: low grade glioma; NOS: not otherwise specified; PNET: primitive neuroectodermal tumor; ANA_PA: anaplastic pilocytic astrocytoma; DLGNT: diffuse leptomeningeal glioneuronal tumor; LGG_PA_PF: low grade pilocytic astrocytoma posterior fossa; EPN_SPINE: ependymomas spinal; HPAP: high-grade glioma with pleomorphic and pseudopapillary features; LGG_PA_GG_ST: low grade glioma subclass hemispheric pilocytic astrocytoma and ganglioglioma; LG_PA_MID: low grade glioma subclass midline pilocytic astrocytomas; DMG_K27: diffuse midline glioma H3K27M mutant; SUBEPN_ST: subependymoma supratentorial; CONTR, HYPTHAL: control hypothalamus; CONTR, PONS: control pons; CONTR, WM: control white matter. For the remaining diagnostic classes, see Capper et al., Supplementary Table 1 [15].

freeneuropathology.org

Copyright: © 2026 The author(s). This is an open access article distributed under the terms of the Creative Commons Attribution 4.0 International License (https://creativecommons.org/licenses/by/4.0/), which permits unrestricted use, distribution, and reproduction in any medium, provided the original author and source are credited, a link to the Creative Commons license is provided, and any changes are indicated. The Creative Commons Public Domain Dedication waiver (https://creativecommons.org/publicdomain/zero/1.0/) applies to the data made available in this article, unless otherwise stated.
