# Supplementary material for: A MAGIBU-based model for pediatric and juvenile CNS tumors: an in-house epigenetic decision-support framework compared with online DNA methylation classifiers
Source: Free Neuropathol. 2026 Aug 24;7:21. doi: 10.17879/freeneuropathology-2026-9648 (PMC13532279; doi:10.17879/freeneuropathology-2026-9648)
Supplement: Supplementary file 2 [file freeneuropathol-07-21-9648-s3.pdf]

Supplementary Methods

Cross platform validation on PromethION and WGBS data

Validation samples were obtained from GSE289246, which combines three sequencing platforms profiling the same panel of CNS tumors. Series matrices were parsed to assign each GEO accession to one of: MinION (GPL24106, R9 flow cells), NovaSeq 6000 WGBS (GPL24676, NEBNext Methyl seq, S4 PE150) or PromethION/P2 Solo (GPL26167, R10.4.1). Per sample truth labels were taken from the tumor type field of each GSE289246 series matrix; both truth labels and MAGIBU predictions were then collapsed to a shared canonical family vocabulary (see Supplementary Table 1) and compared at the family level.

WGBS handling

Per sample bedMethyl files were converted into MAGIBU input TSVs by retaining the Illumina probe IDs, computing Beta as percentMeth / 100, and shifting the BED 0 based start coordinate by +1 to obtain the 1 based hg38 position used by the Illumina manifest. All 22 WGBS samples were projected individually onto each of the seven UMAP models.

PromethION pooling

To compensate for the near 1x per CpG coverage of PromethION single read BEDs, samples were aggregated by subclass (minimum 5 samples per pool, four pools: GBM n = 21, A_IDH grade 3 n = 7, O_IDH n = 5, PA n = 5) and by canonical family (minimum 2 samples per pool, five pools: GBM n = 22, A_IDH n = 9, PA n = 6, O_IDH n = 5, LGNT n = 2). For each pool, methylated read counts and total coverage were summed across contributing BED files at every CpG site, and a minimum aggregated coverage of 1 was applied. Pseudo sample Beta was recomputed as Σn_meth / Σn_cov; for duplicated probe IDs the coverage weighted mean was used. The resulting probe level TSVs were annotated with hg38 coordinates via the EPICv1 manifest and contained from 322000 to 478000 probes per pool (median coverage 1.3 to 6.8x).

Projection and classification

Each TSV (per sample WGBS and pooled PromethION pseudo samples) was projected onto seven MAGIBU UMAP models trained at n_cpg ∈ {500; 1.000; 10.000; 25.000; 50.000; 75.000; 100.000}. Missing CpGs were first imputed by proximity within 500 bp. The top three nearest training subclasses were extracted with the KNN 5 voting scheme implemented in the get_top_3_distances function. Top 1 accuracy is the fraction of samples (or pools) for which the closest neighbor belongs to the correct family; Top 3 accuracy is the fraction for which the correct family is recovered by at least one of the three nearest neighbors. Exact subclass matching was not evaluated because MAGIBU predictions use the Capper subclass code (e.g. GBM, MES) while GEO tumor type annotations use WHO 2021 long form diagnoses (e.g. Glioblastoma, IDH wildtype); the two vocabularies are coarse grained at different levels and not bijectively mappable.

Copyright: © 2026 The author(s). This is an open access article distributed under the terms of the Creative Commons Attribution 4.0 International License (https://creativecommons.org/licenses/by/4.0/), which permits unrestricted use, distribution, and reproduction in any medium, provided the original author and source are credited, a link to the Creative Commons license is provided, and any changes are indicated. The Creative Commons Public Domain Dedication waiver (https://creativecommons.org/publicdomain/zero/1.0/) applies to the data made available in this article, unless otherwise stated.
