# Supplementary material for: A MAGIBU-based model for pediatric and juvenile CNS tumors: an in-house epigenetic decision-support framework compared with online DNA methylation classifiers
Source: Free Neuropathol. 2026 Aug 24;7:21. doi: 10.17879/freeneuropathology-2026-9648 (PMC13532279; doi:10.17879/freeneuropathology-2026-9648)

Supplementary Data

Supplementary Data 1. EpiDiP classification reports for the 31 crossNN head to head samples. Individual EpiDiP web portal reports for the 31 array samples from GSE289137 used in the head-to-head comparison with MAGIBU (**Supplementary Table 3**). For each sample the report shows the methylation UMAP positioning of the case relative to the EpiDiP reference landscape and the corresponding genome wide copy number profile; samples are ordered as in **Supplementary Table 3**.

freeneuropathology.org

Copyright: © 2026 The author(s). This is an open access article distributed under the terms of the Creative Commons Attribution 4.0 International License (https://creativecommons.org/licenses/by/4.0/), which permits unrestricted use, distribution, and reproduction in any medium, provided the original author and source are credited, a link to the Creative Commons license is provided, and any changes are indicated. The Creative Commons Public Domain Dedication waiver (https://creativecommons.org/publicdomain/zero/1.0/) applies to the data made available in this article, unless otherwise stated.

EpiDiP Report for Sample 201465970005_R01C01

Generated on	:	epidip02 / 20260616_172156
Detected barcode	:	undetermined
Valid reads	:	0
450K overlap CpG count	:	0
Reference data	:	AllIDATv2_20210804_HPAP_Sarc

Methylation UMAP plot

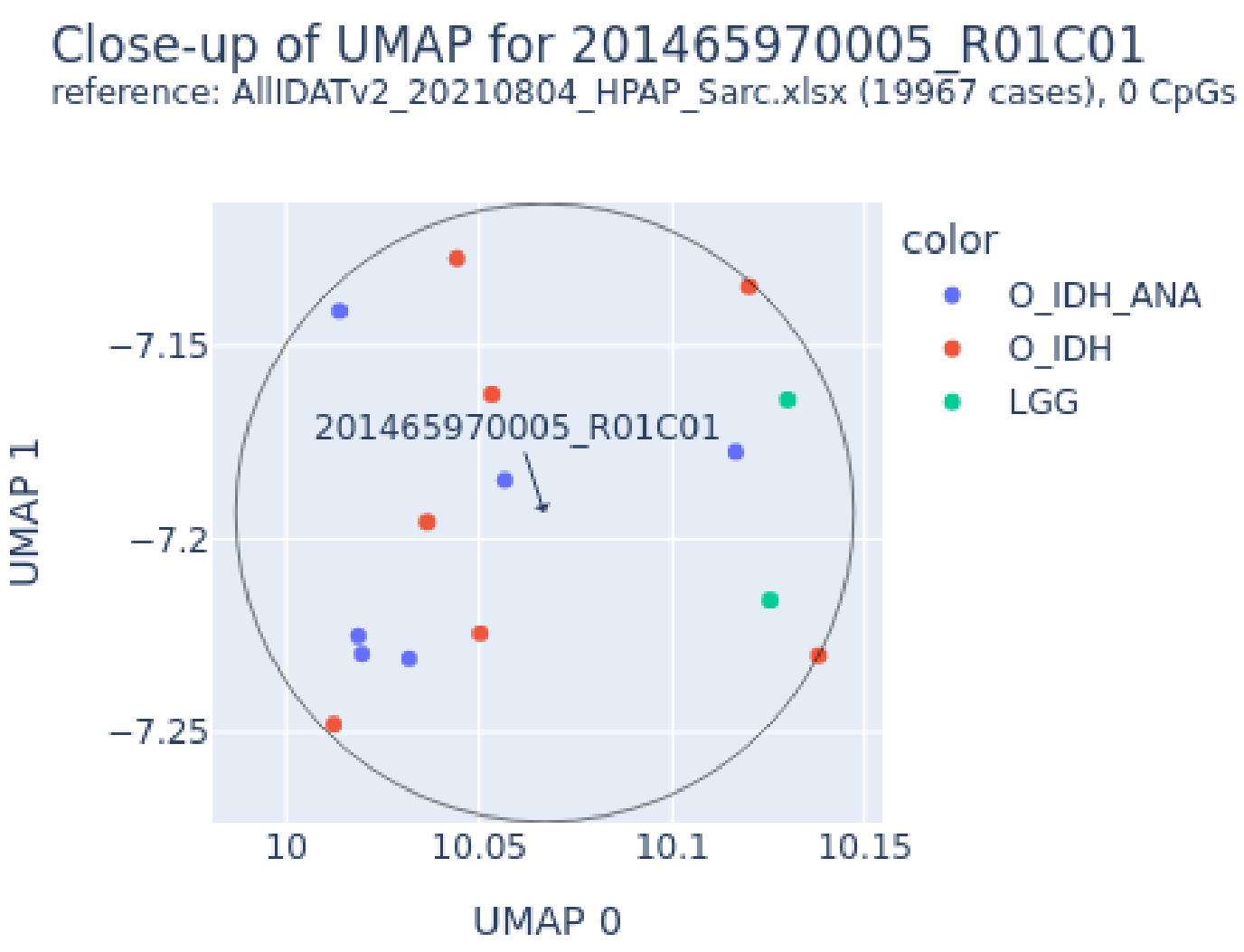

Methylation UMAP Score

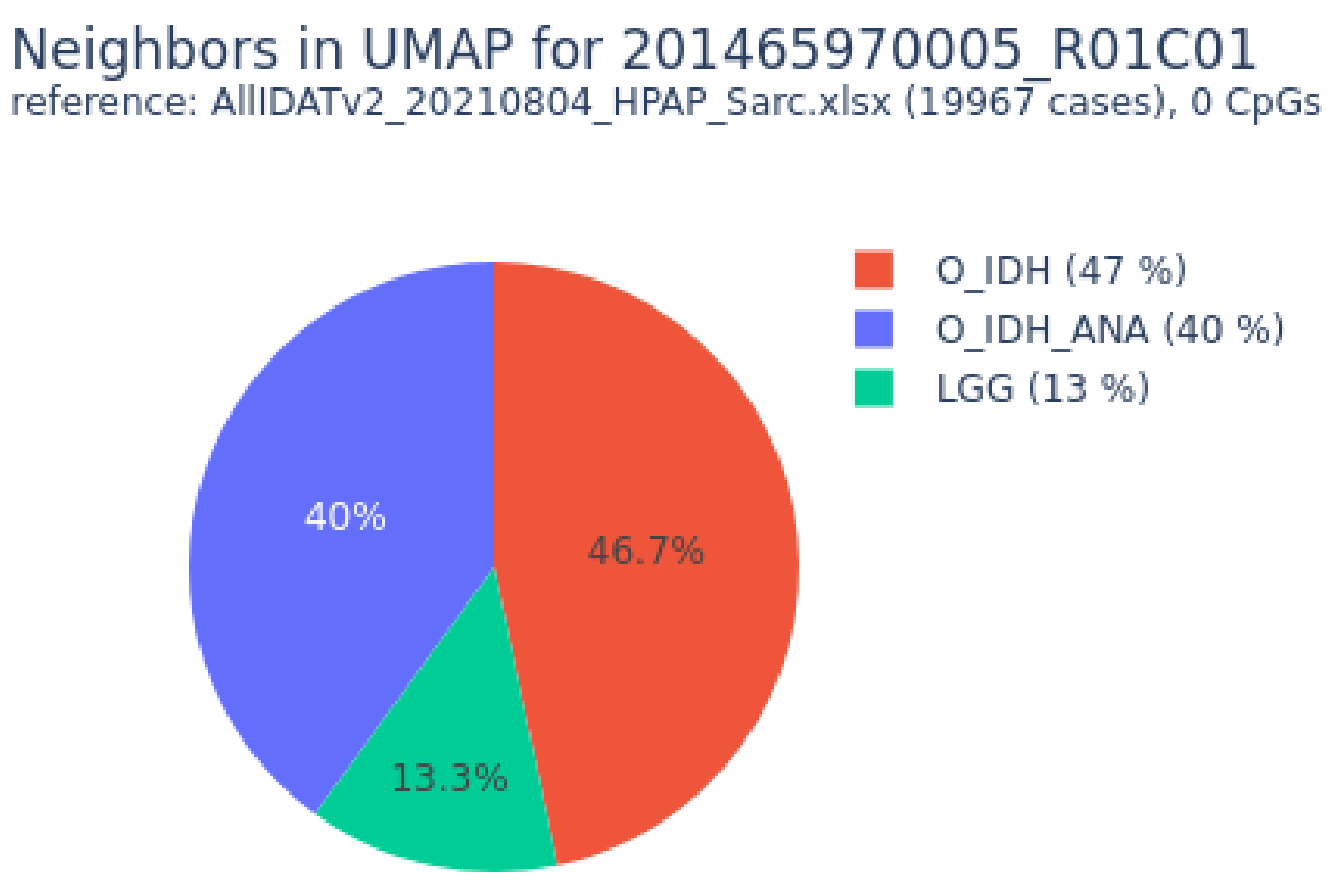

Copy number plot

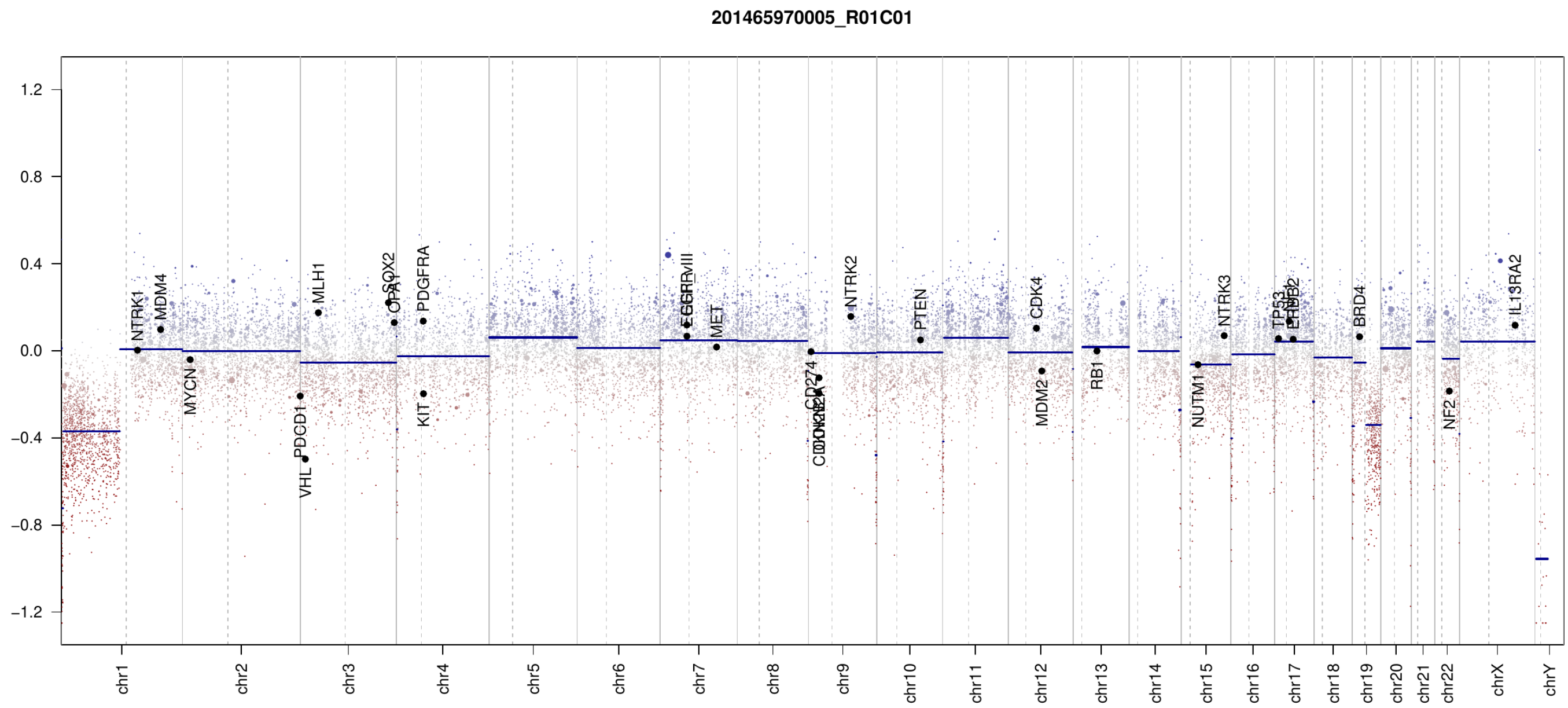

EpiDiP Report for Sample 202995850067_R07C01

Generated on	:	epidip02 / 20260616_172028
Detected barcode	:	undetermined
Valid reads	:	0
450K overlap CpG count	:	0
Reference data	:	AllIDATv2_20210804_HPAP_Sarc

Methylation UMAP plot

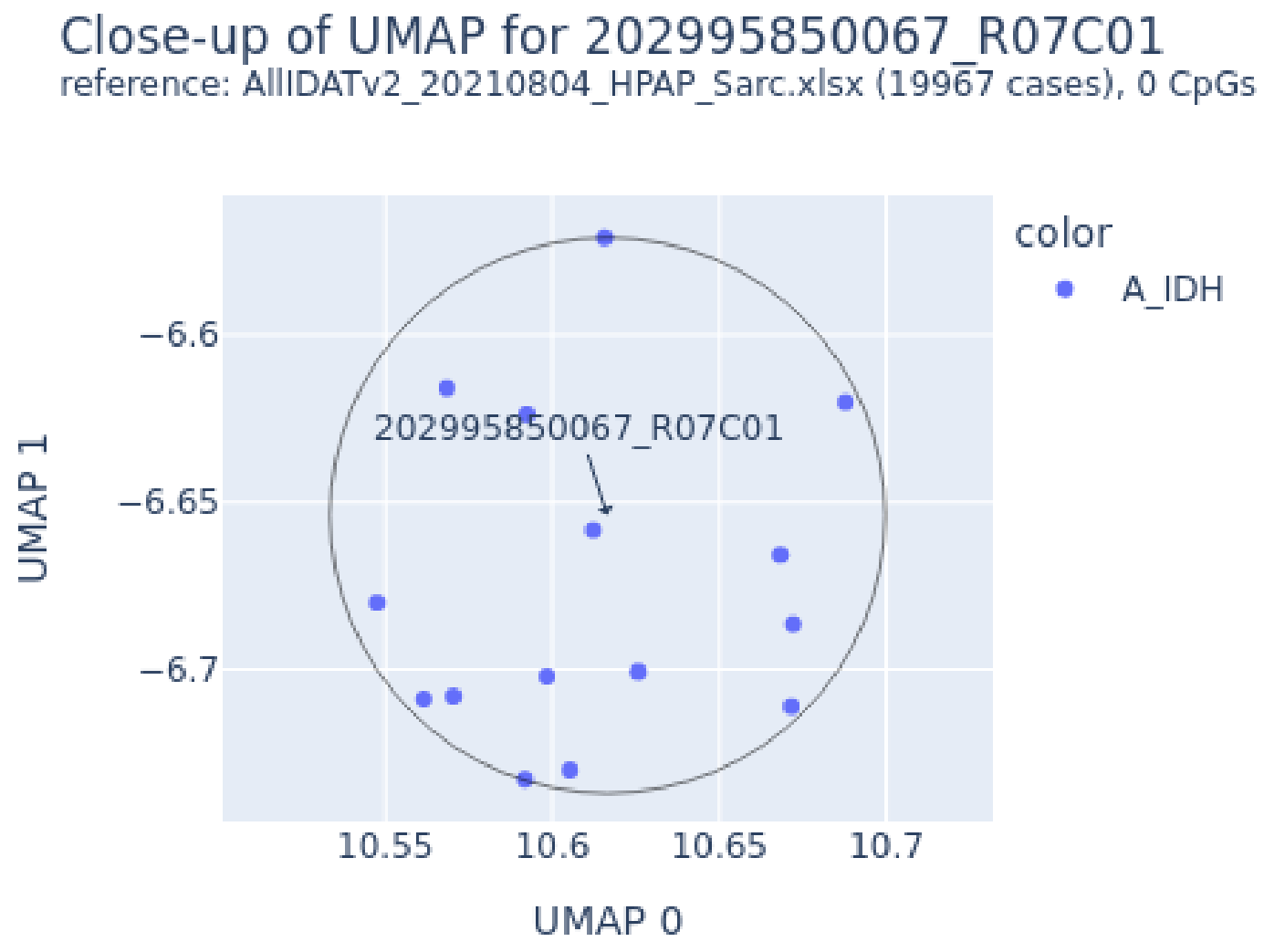

Methylation UMAP Score

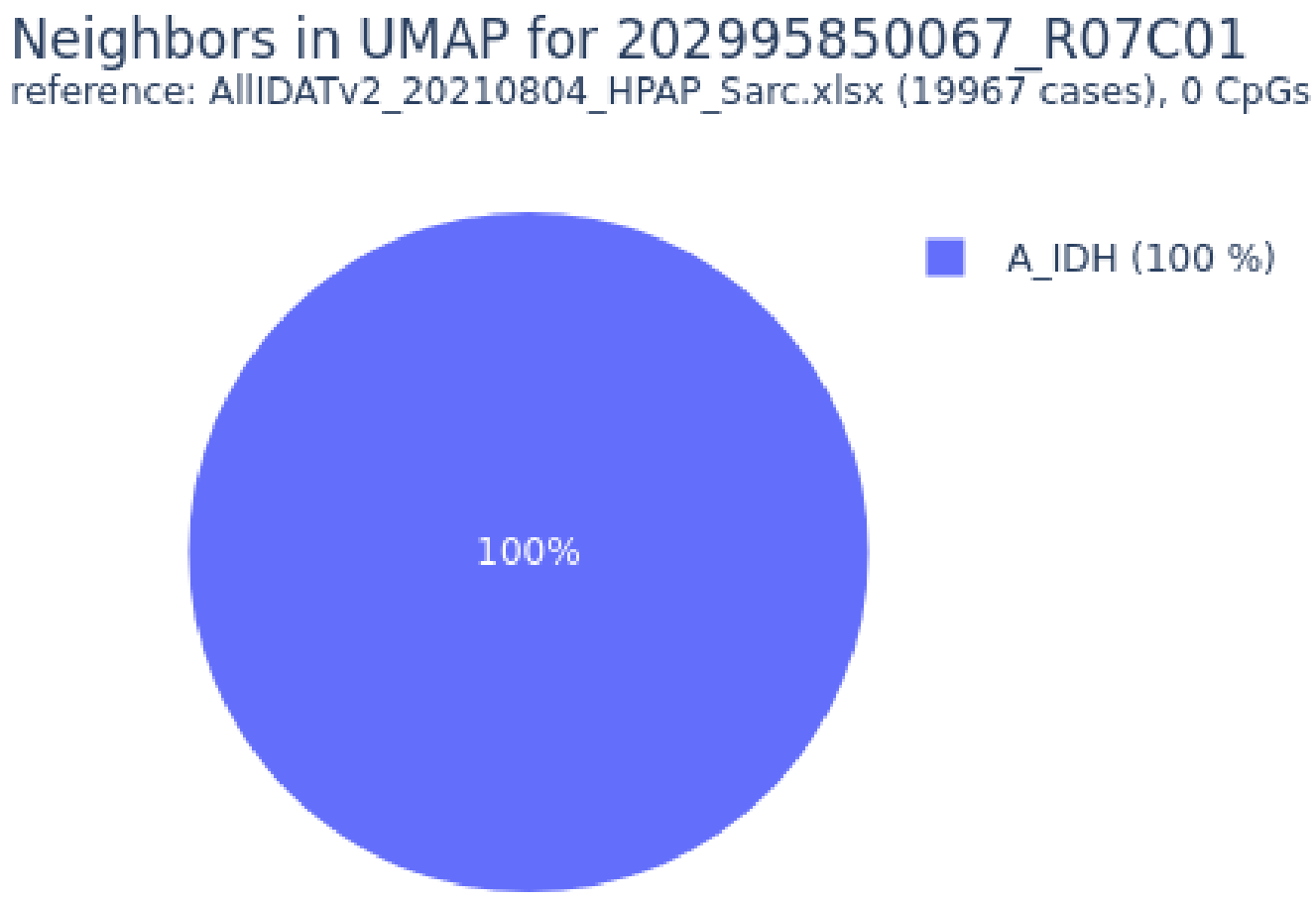

Copy number plot

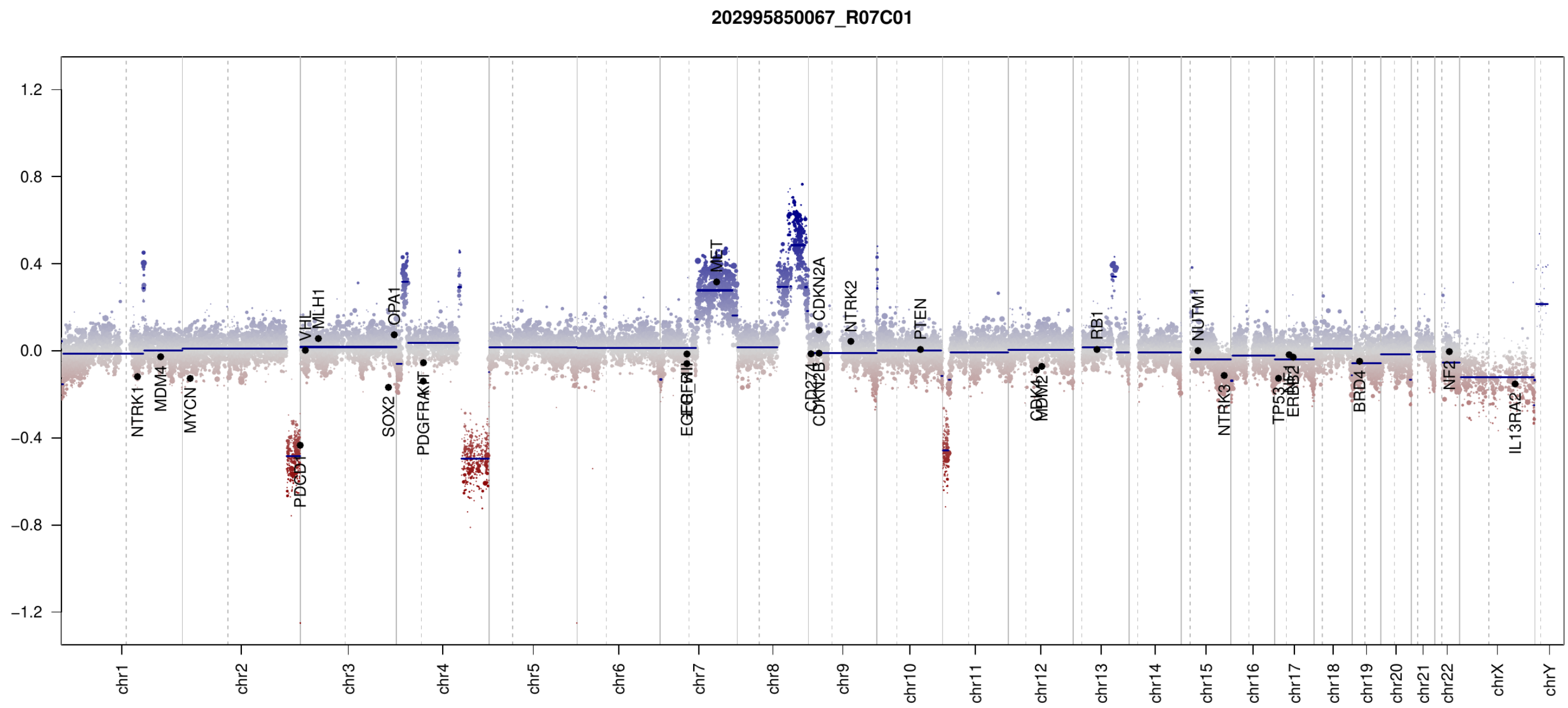

EpiDiP Report for Sample 203013220103_R07C01

Generated on	:	epidip02 / 20260616_172435
Detected barcode	:	undetermined
Valid reads	:	0
450K overlap CpG count	:	0
Reference data	:	AllIDATv2_20210804_HPAP_Sarc

Methylation UMAP plot

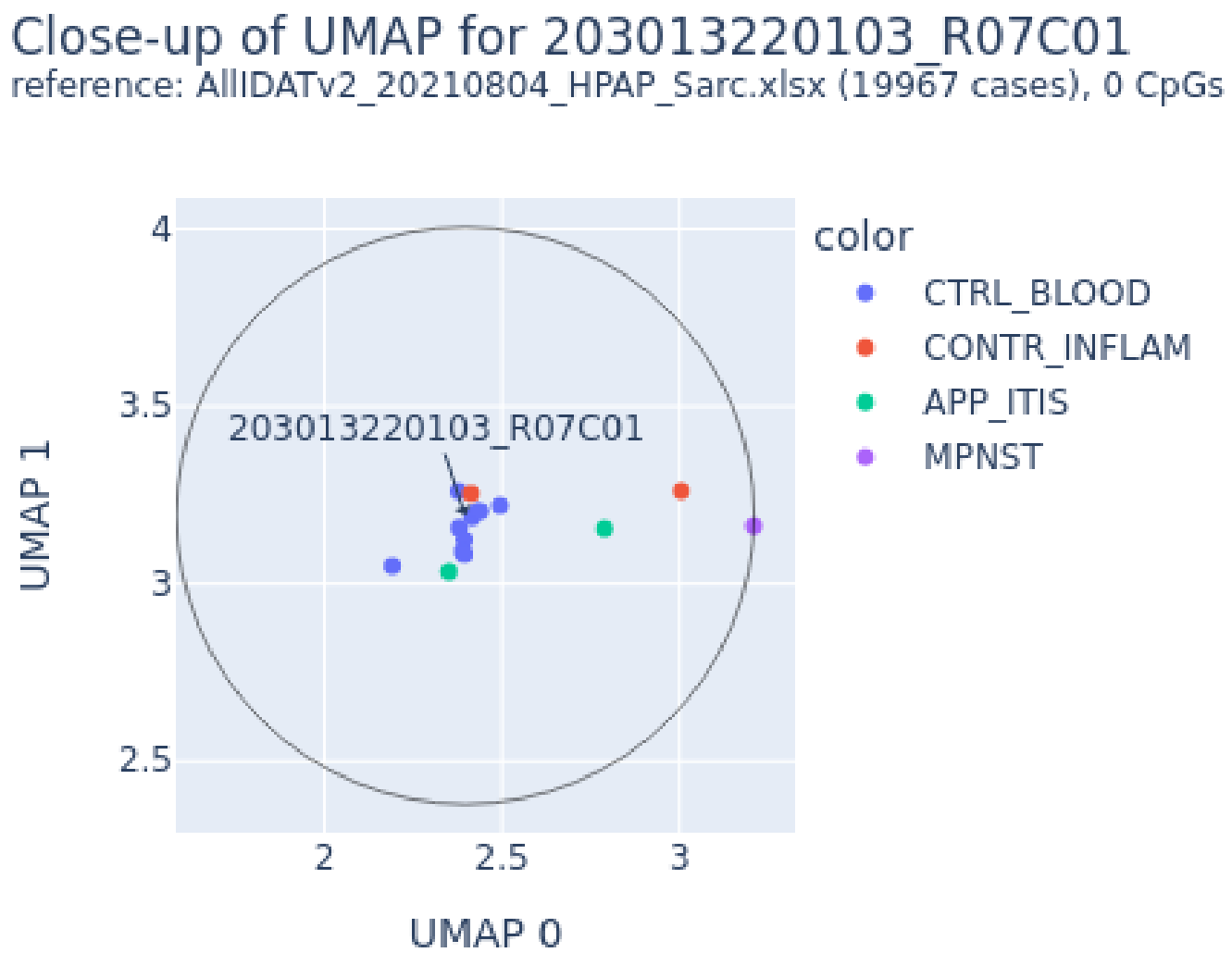

Methylation UMAP Score

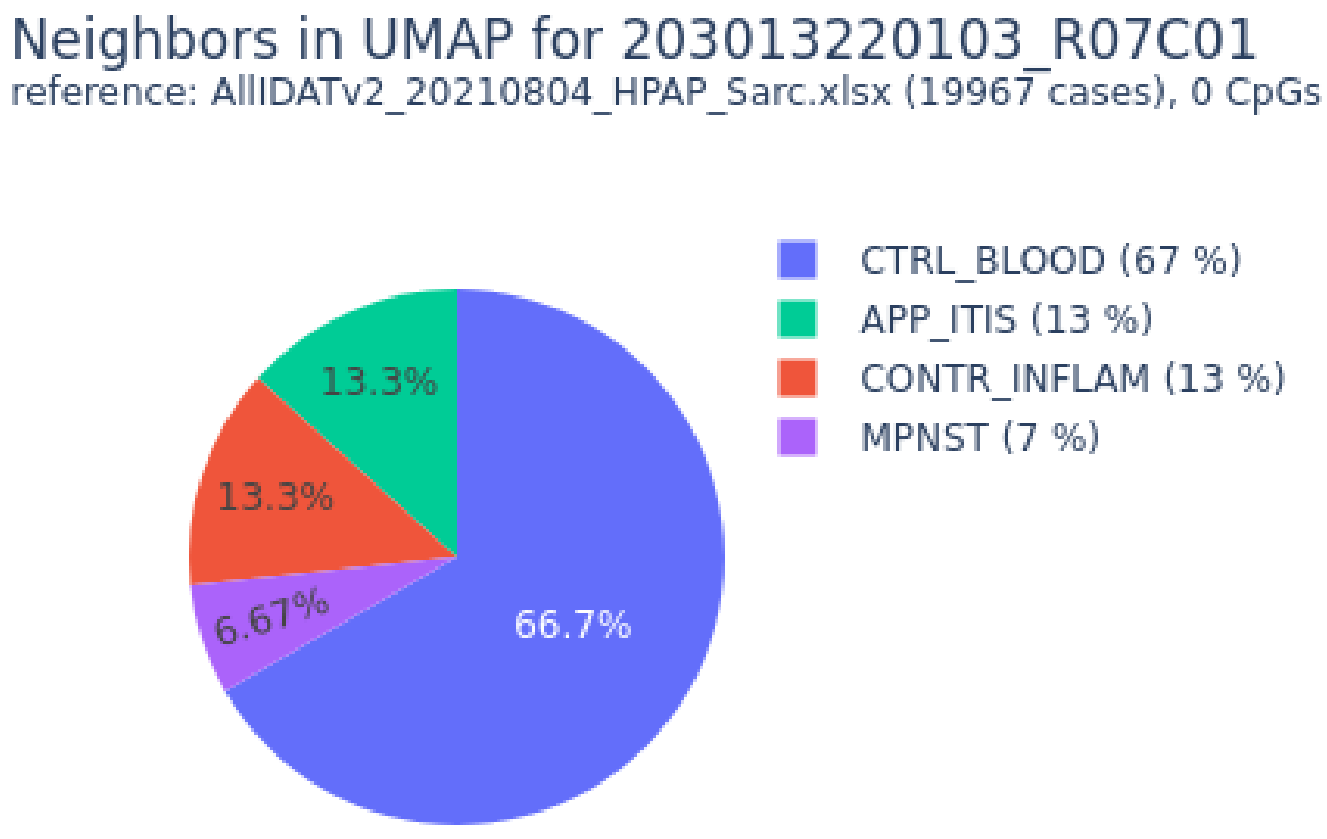

Copy number plot

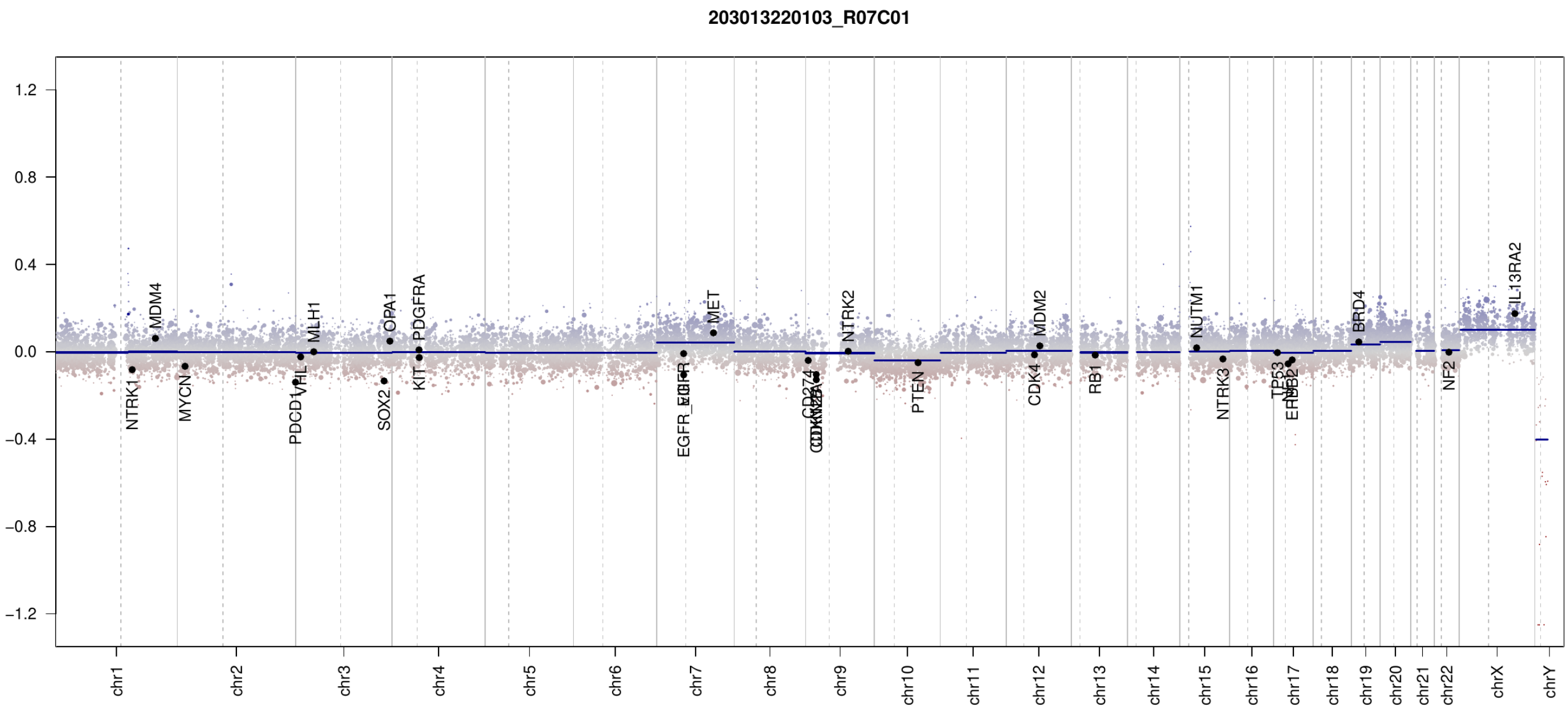

EpiDiP Report for Sample 203017150041_R02C01

Generated on	:	epidip02 / 20260616_172054
Detected barcode	:	undetermined
Valid reads	:	0
450K overlap CpG count	:	0
Reference data	:	AllIDATv2_20210804_HPAP_Sarc

Methylation UMAP plot

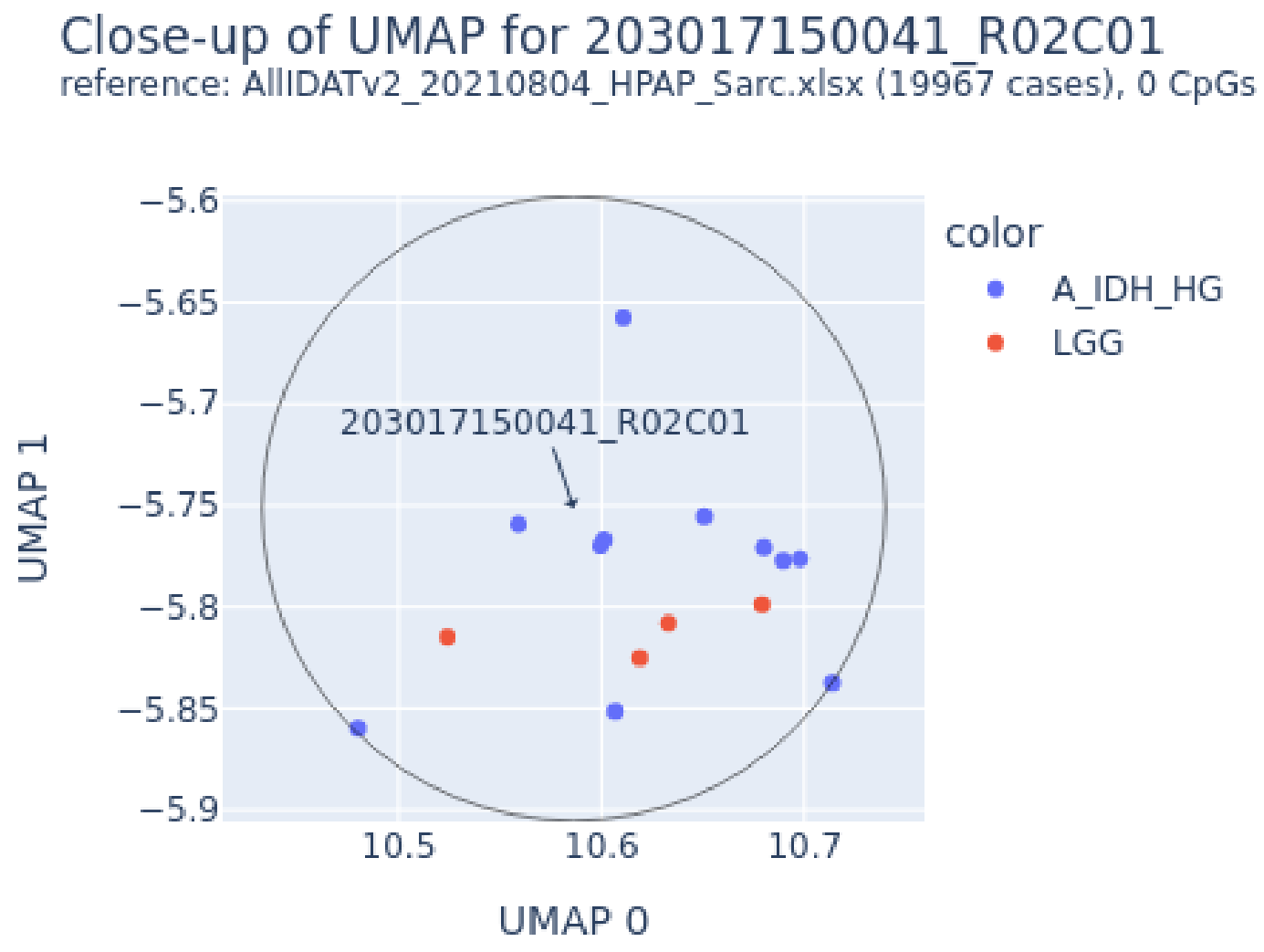

Methylation UMAP Score

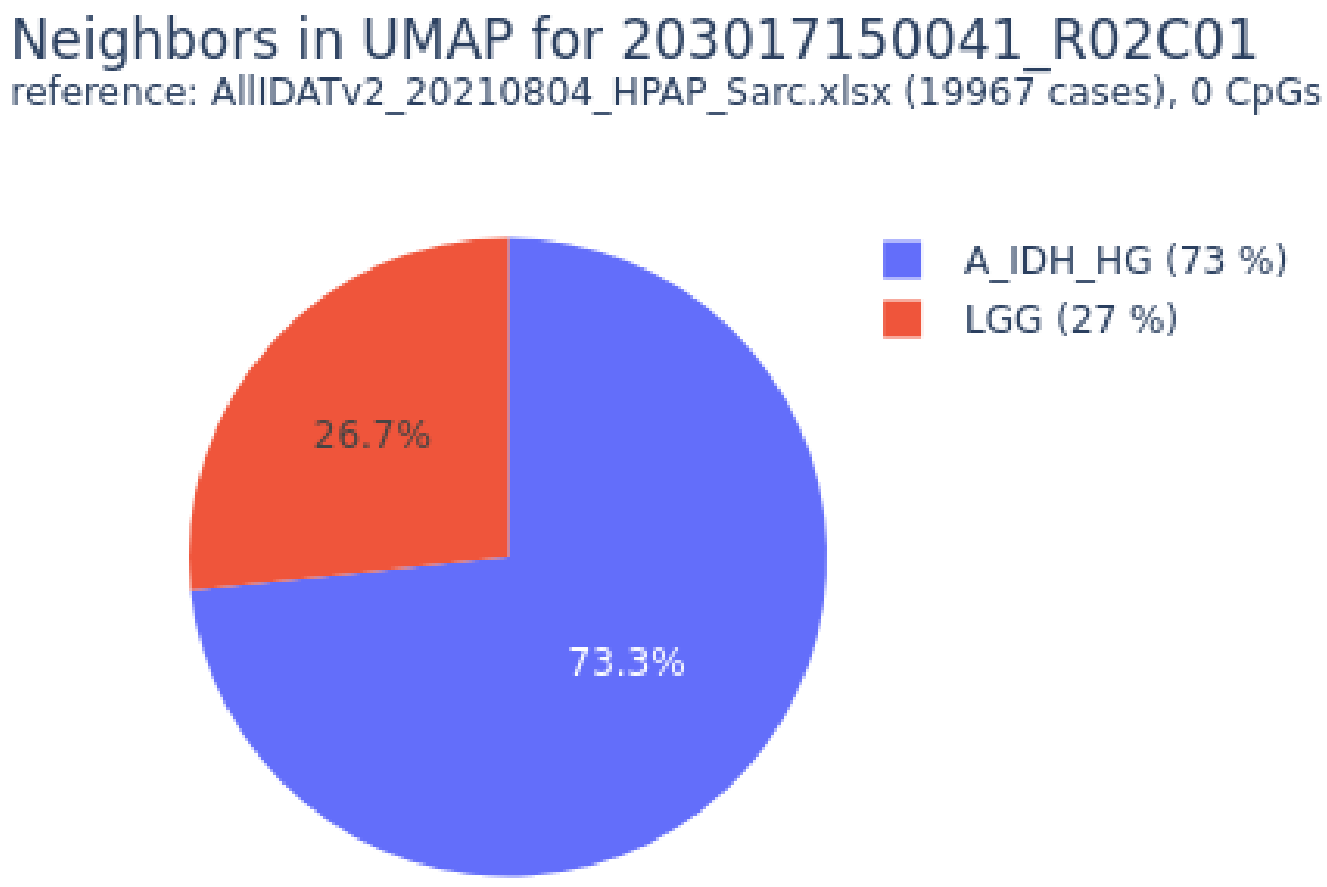

Copy number plot

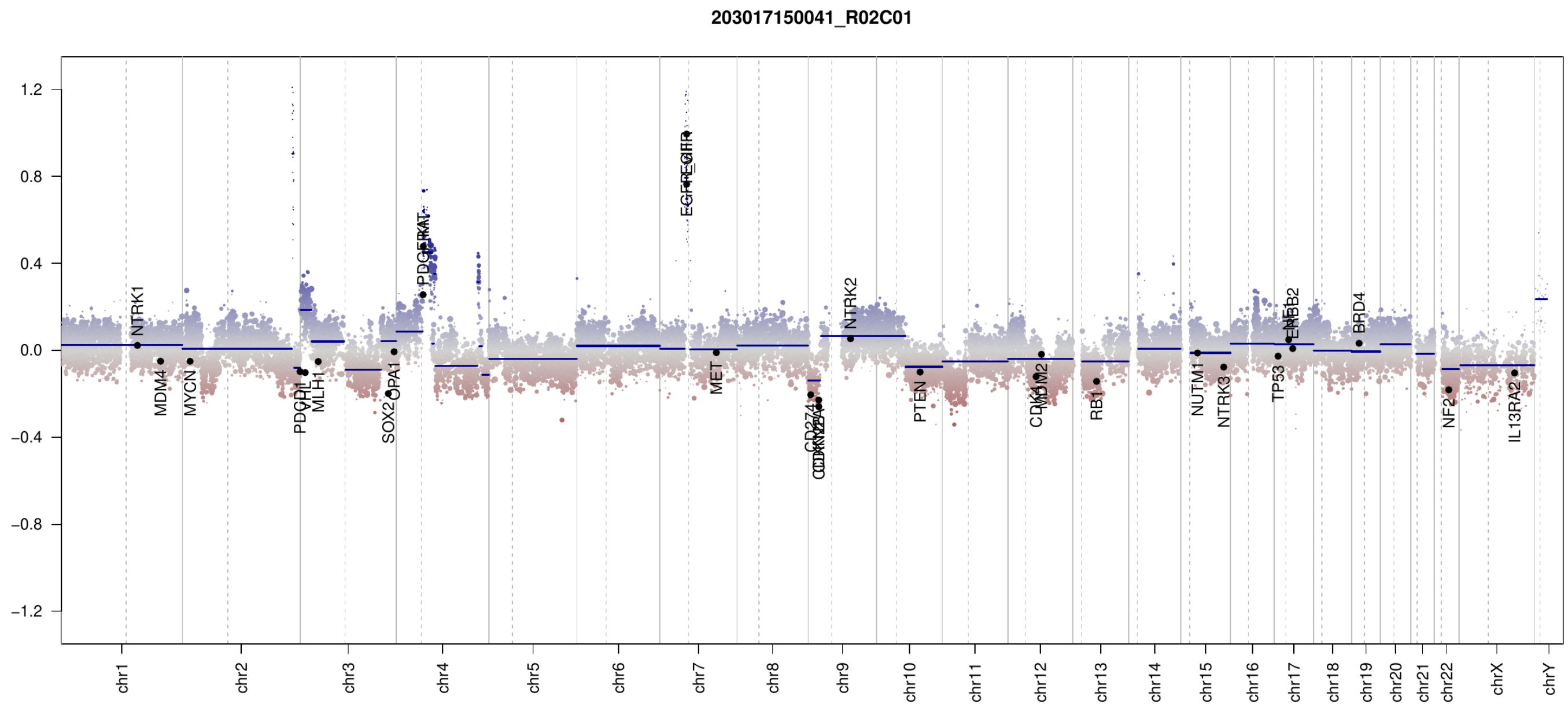

EpiDiP Report for Sample 203810690044_R07C01

Generated on	:	epidip02 / 20260616_172739
Detected barcode	:	undetermined
Valid reads	:	0
450K overlap CpG count	:	0
Reference data	:	AllIDATv2_20210804_HPAP_Sarc

Methylation UMAP plot

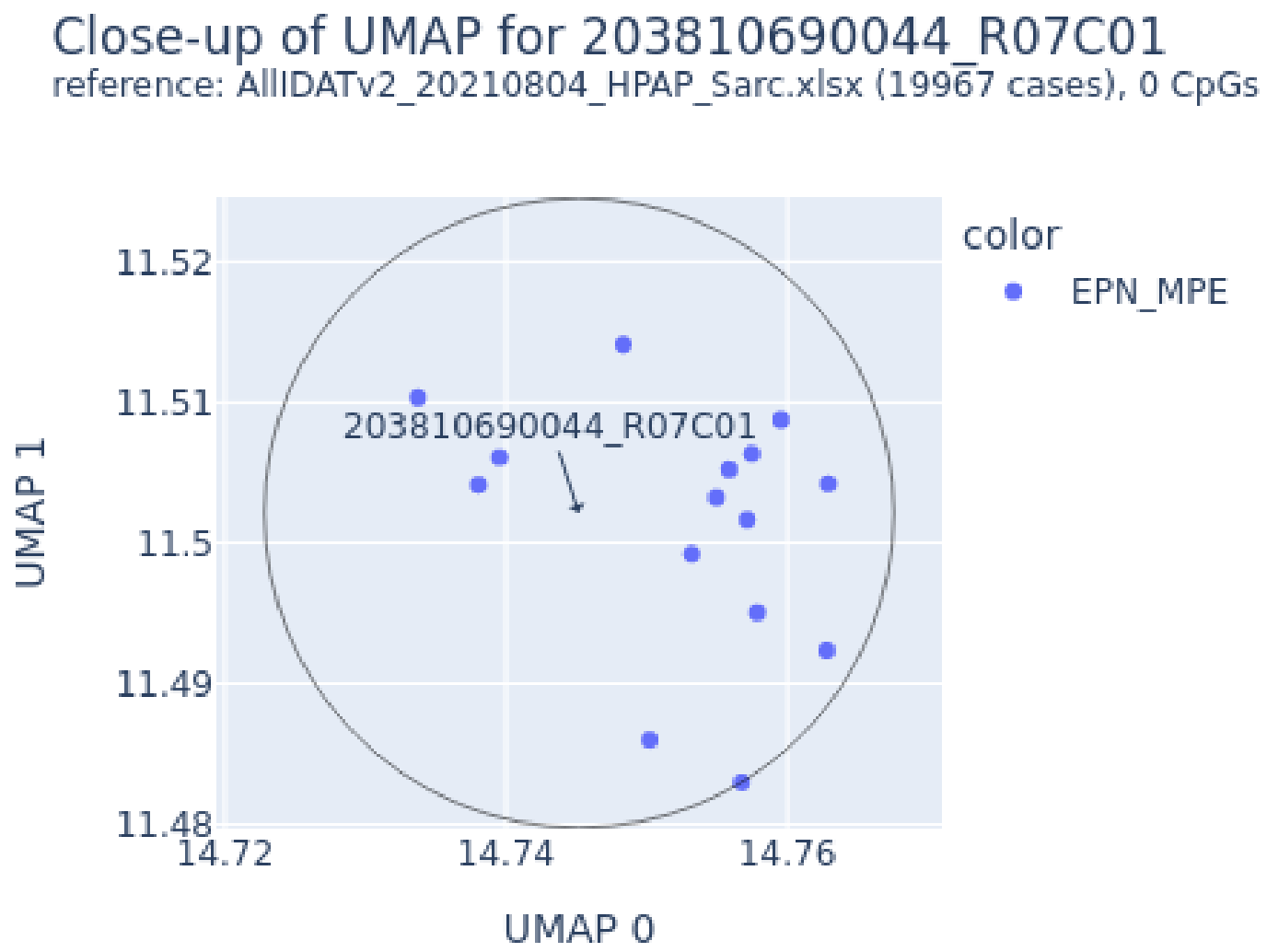

Methylation UMAP Score

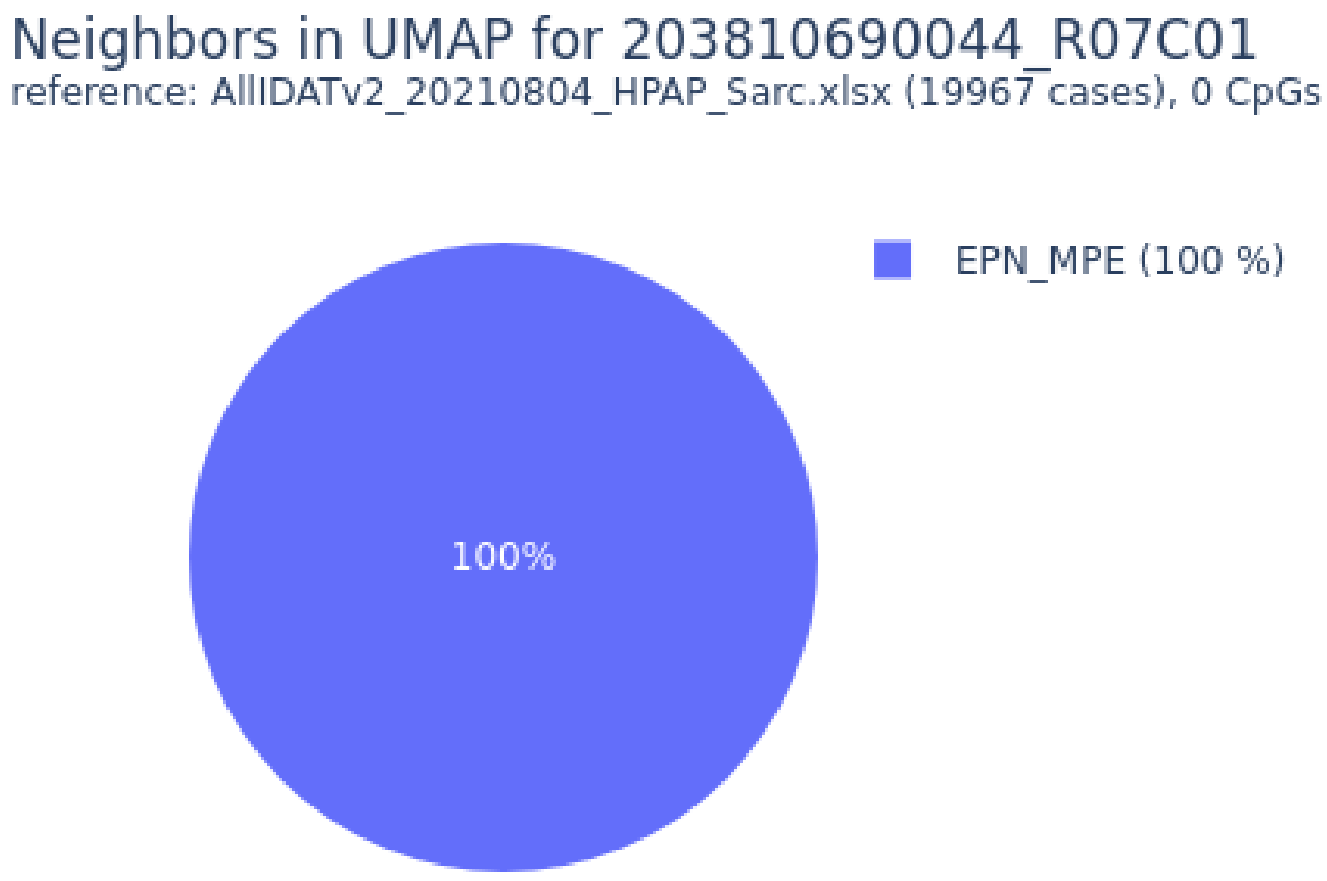

Copy number plot

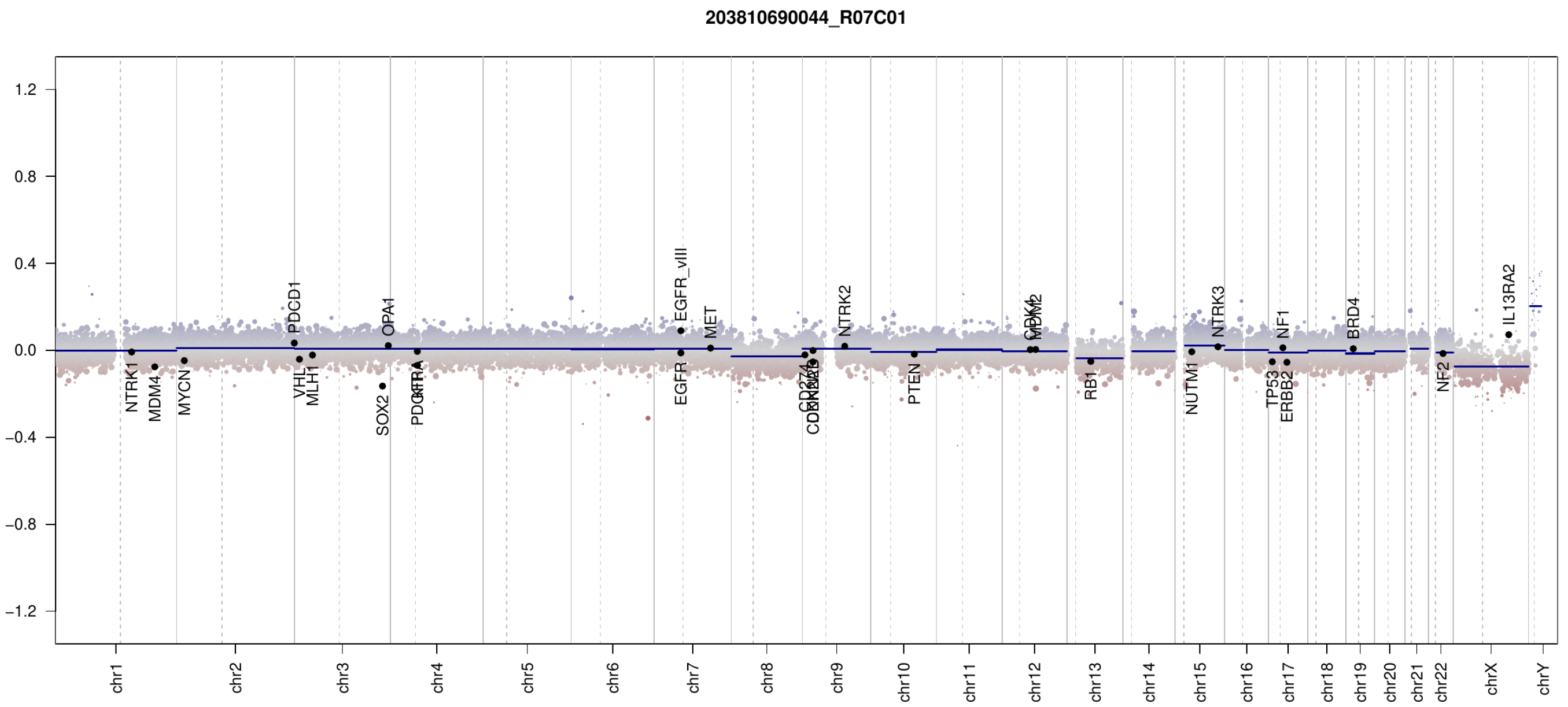

EpiDiP Report for Sample 203821710008_R04C01

Generated on	:	epidip02 / 20260616_171034
Detected barcode	:	undetermined
Valid reads	:	0
450K overlap CpG count	:	0
Reference data	:	AllIDATv2_20210804_HPAP_Sarc

Methylation UMAP plot

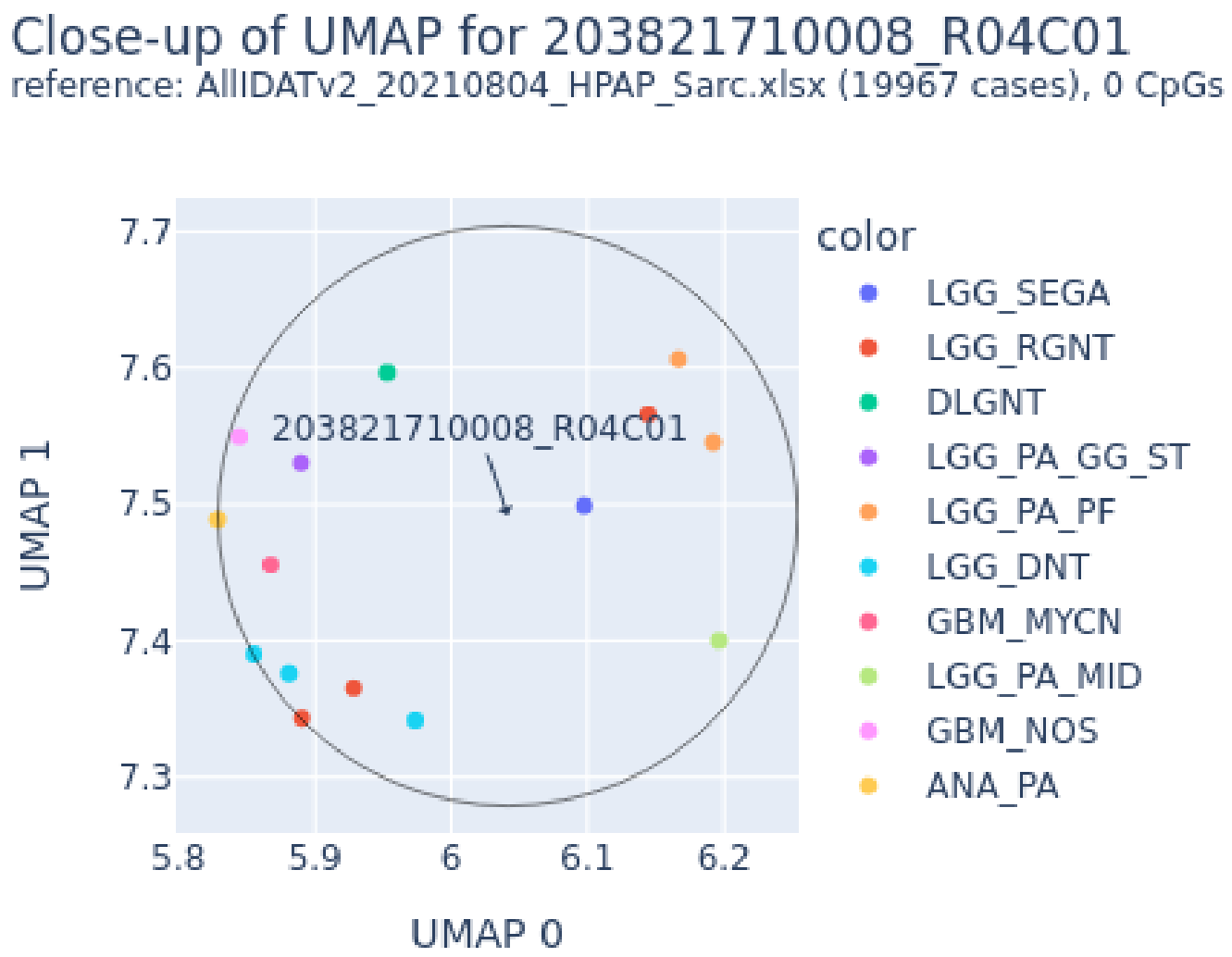

Methylation UMAP Score

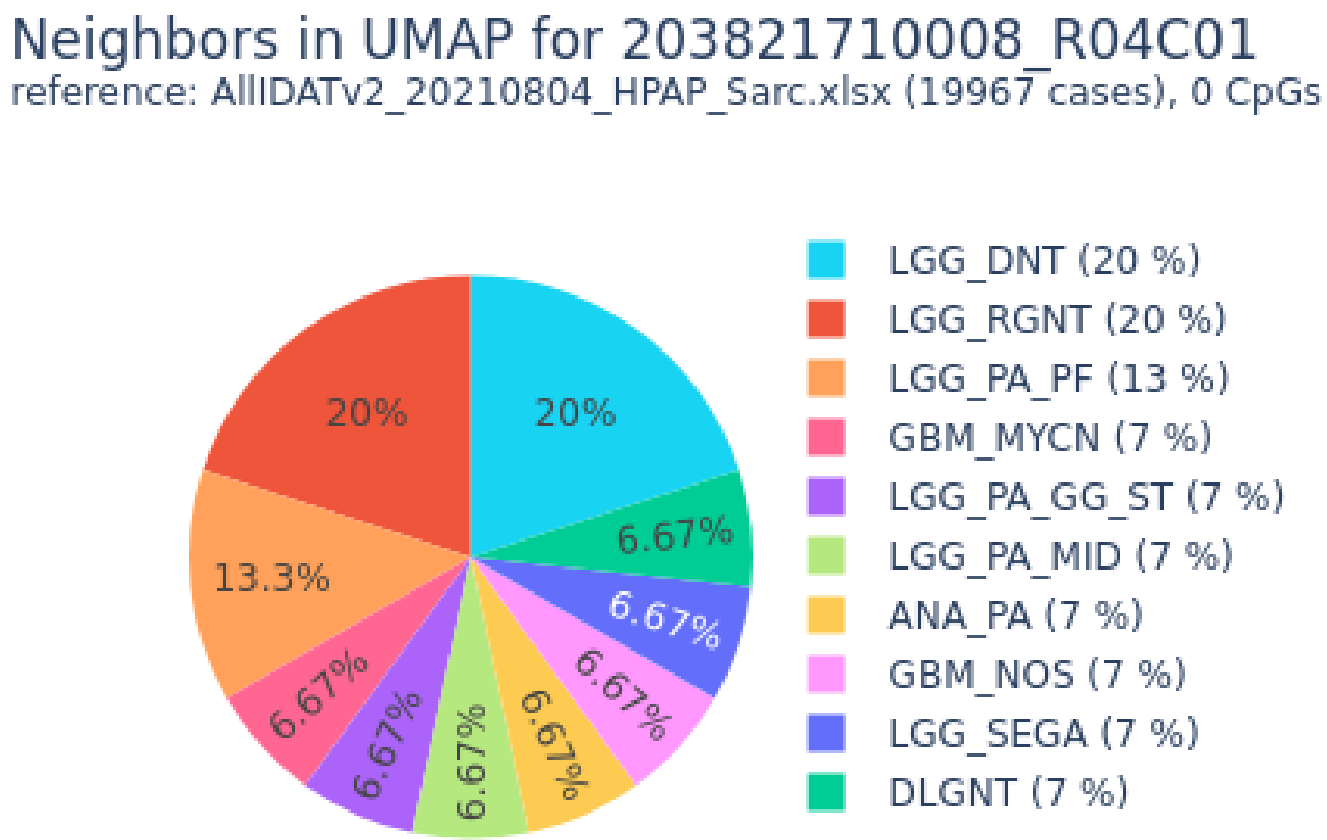

Copy number plot

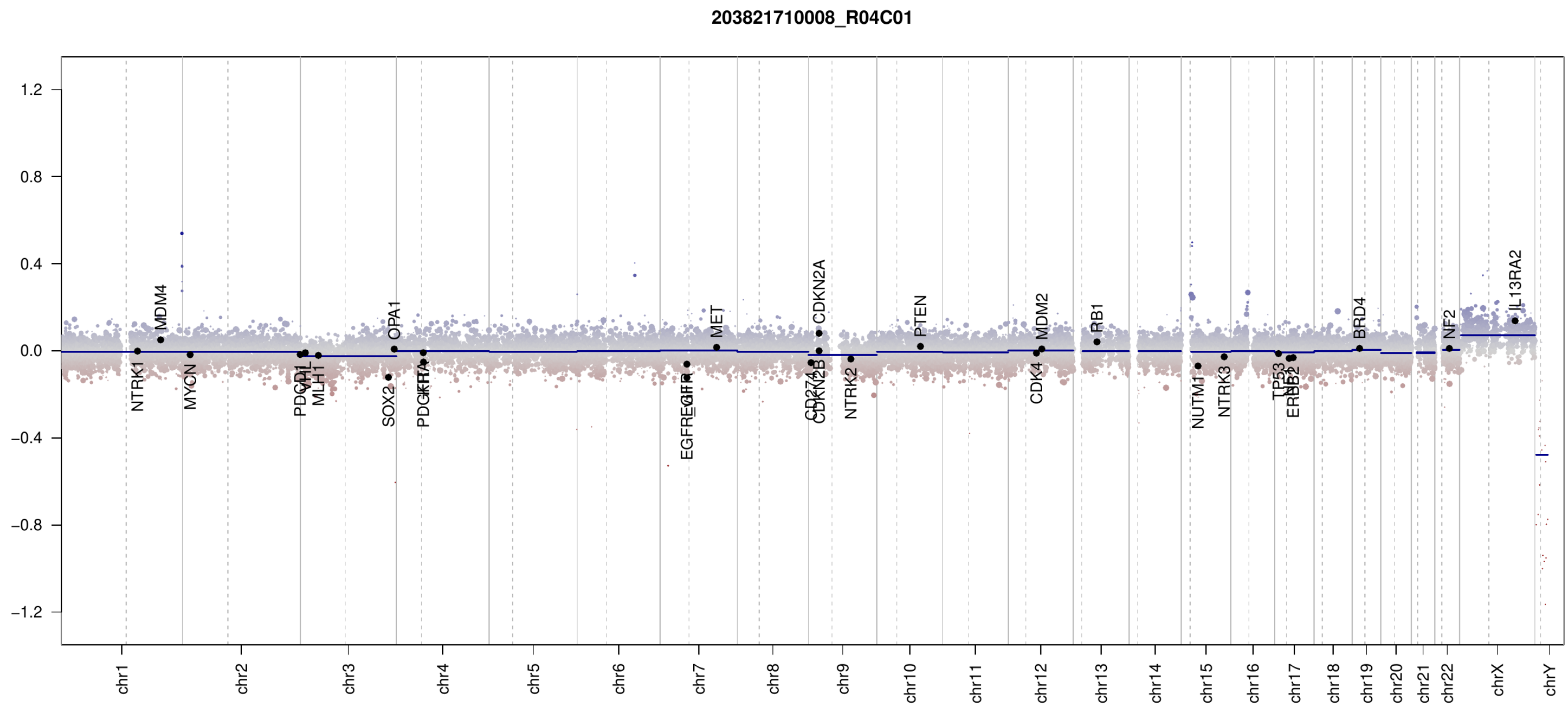

EpiDiP Report for Sample 204425700133_R04C01

Generated on	:	epidip02 / 20260616_171435
Detected barcode	:	undetermined
Valid reads	:	0
450K overlap CpG count	:	0
Reference data	:	AllIDATv2_20210804_HPAP_Sarc

Methylation UMAP plot

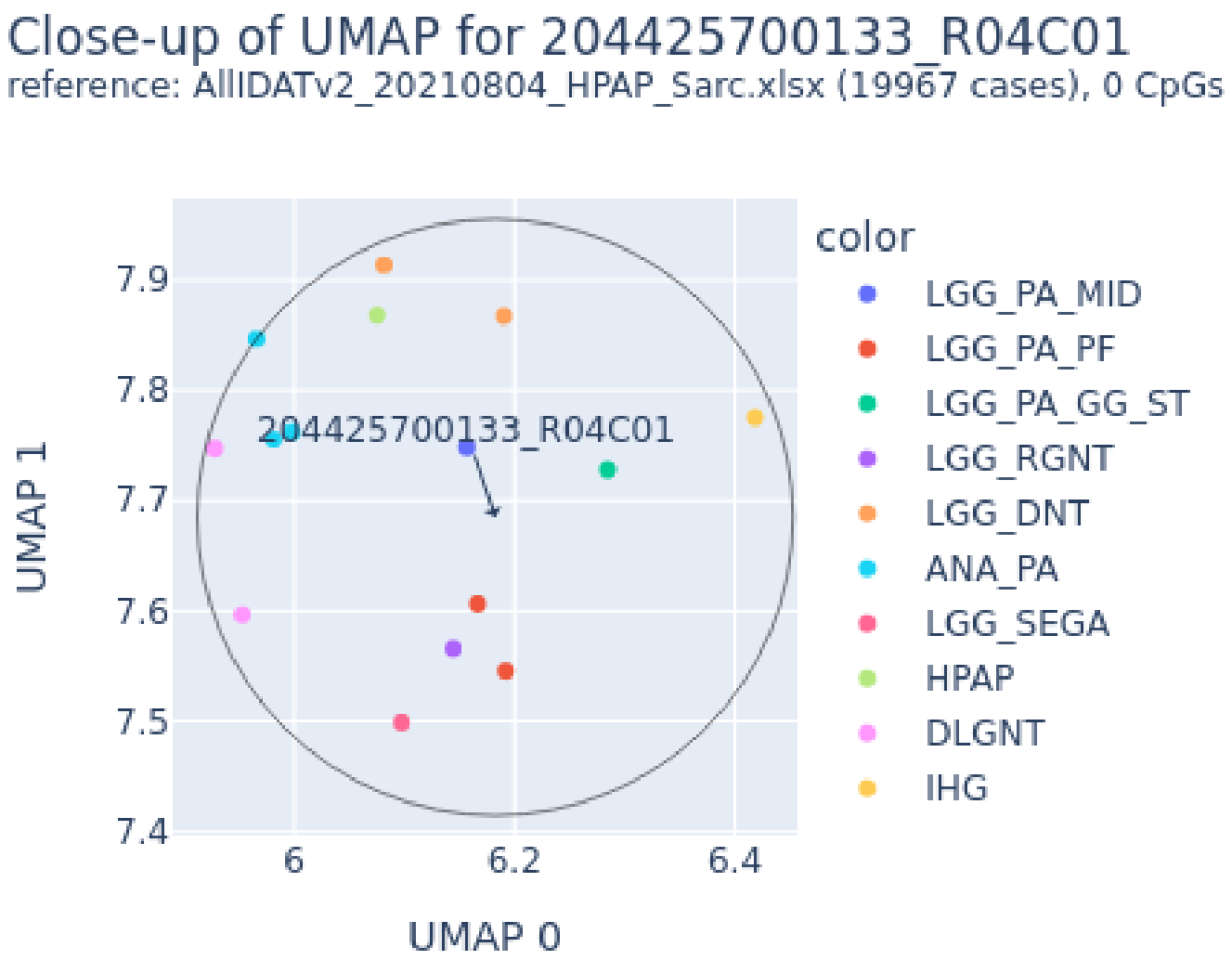

Methylation UMAP Score

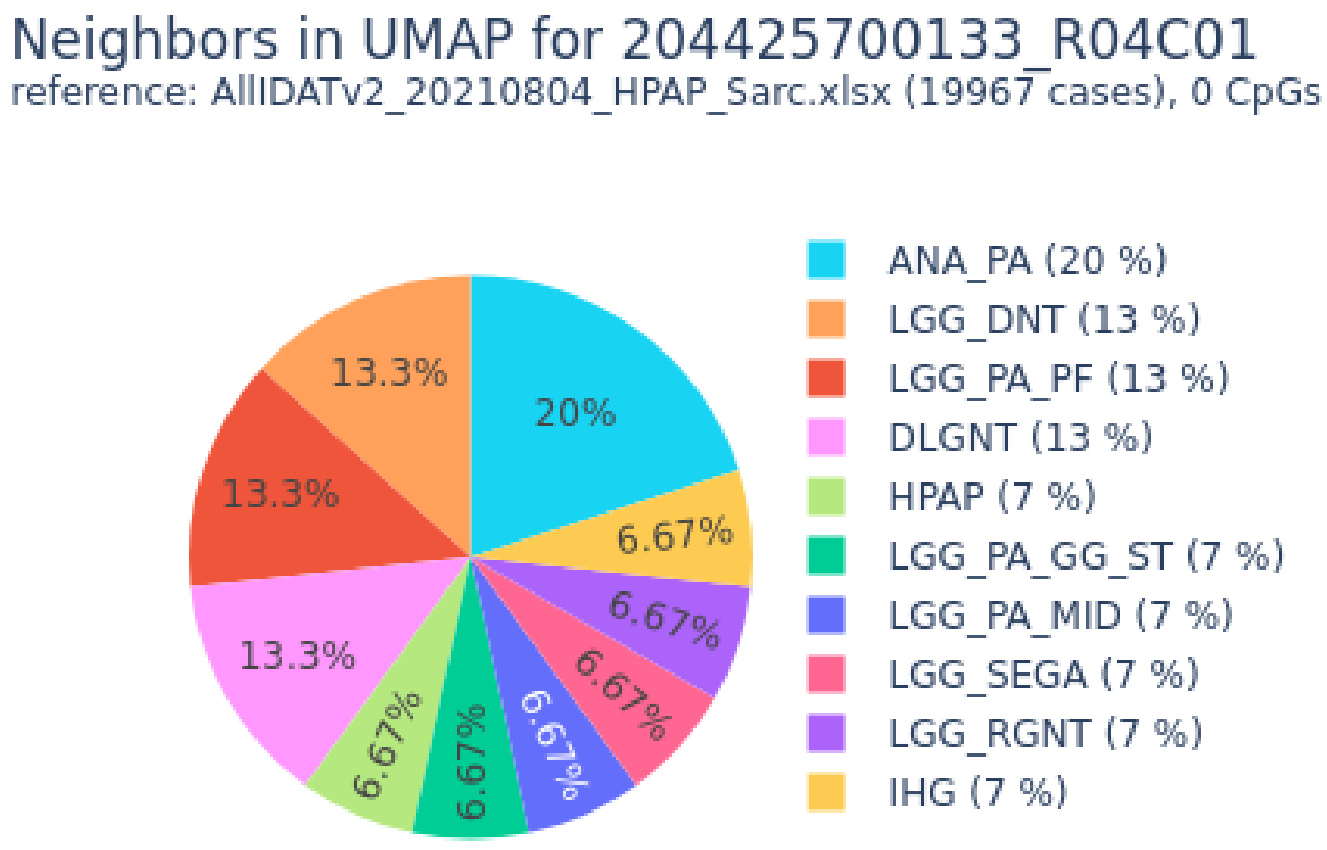

Copy number plot

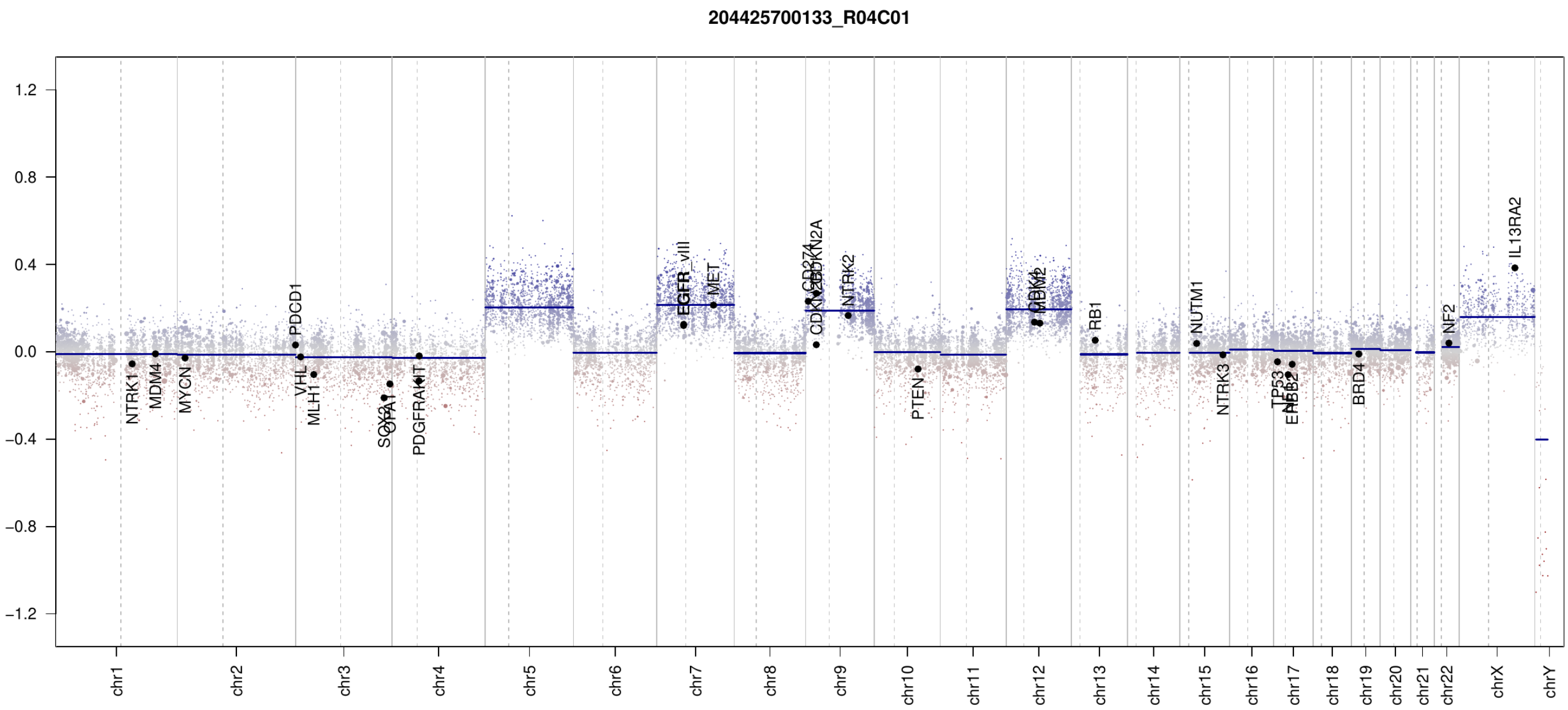

EpiDiP Report for Sample 204425700133_R07C01

Generated on	:	epidip02 / 20260616_172118
Detected barcode	:	undetermined
Valid reads	:	0
450K overlap CpG count	:	0
Reference data	:	AllIDATv2_20210804_HPAP_Sarc

Methylation UMAP plot

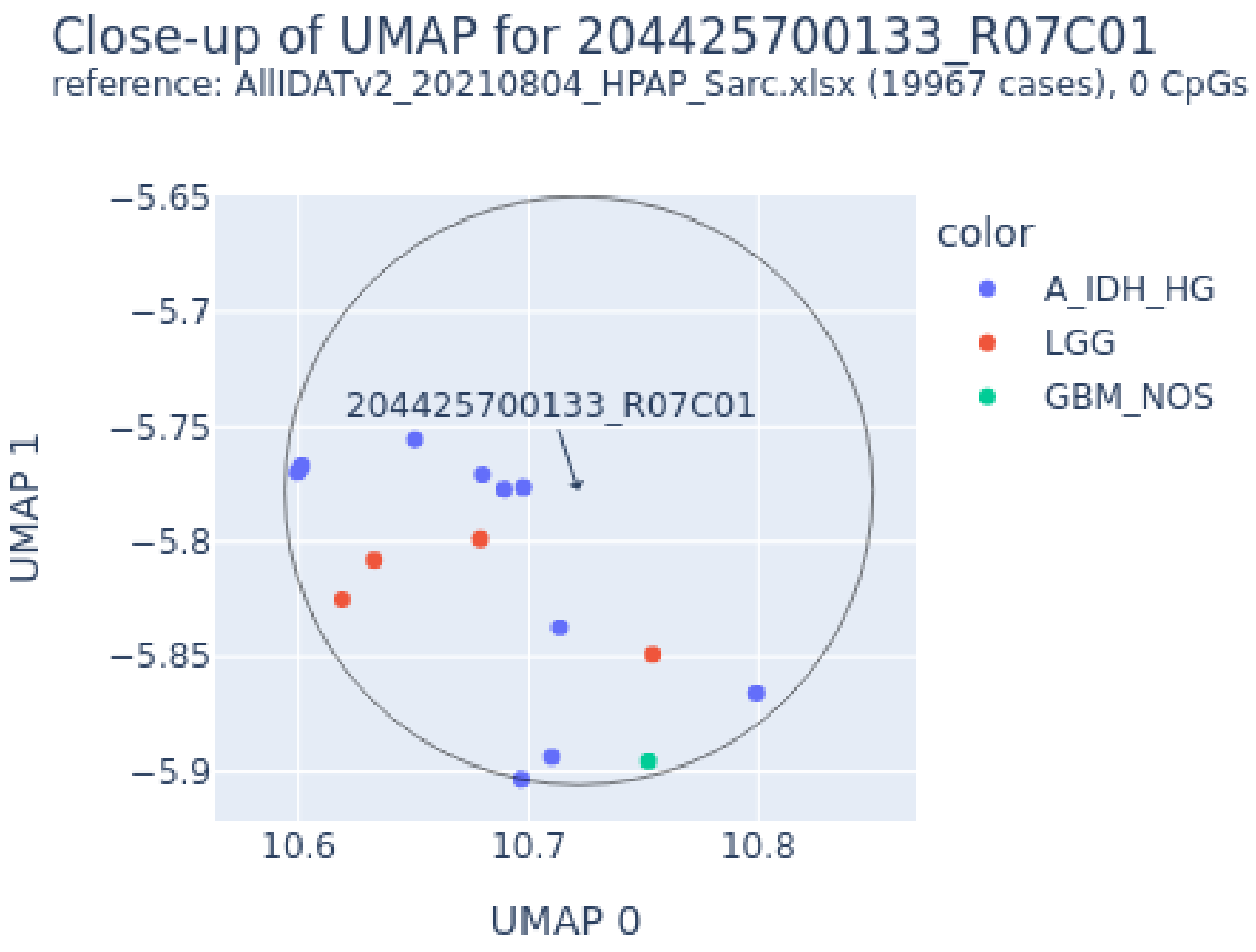

Methylation UMAP Score

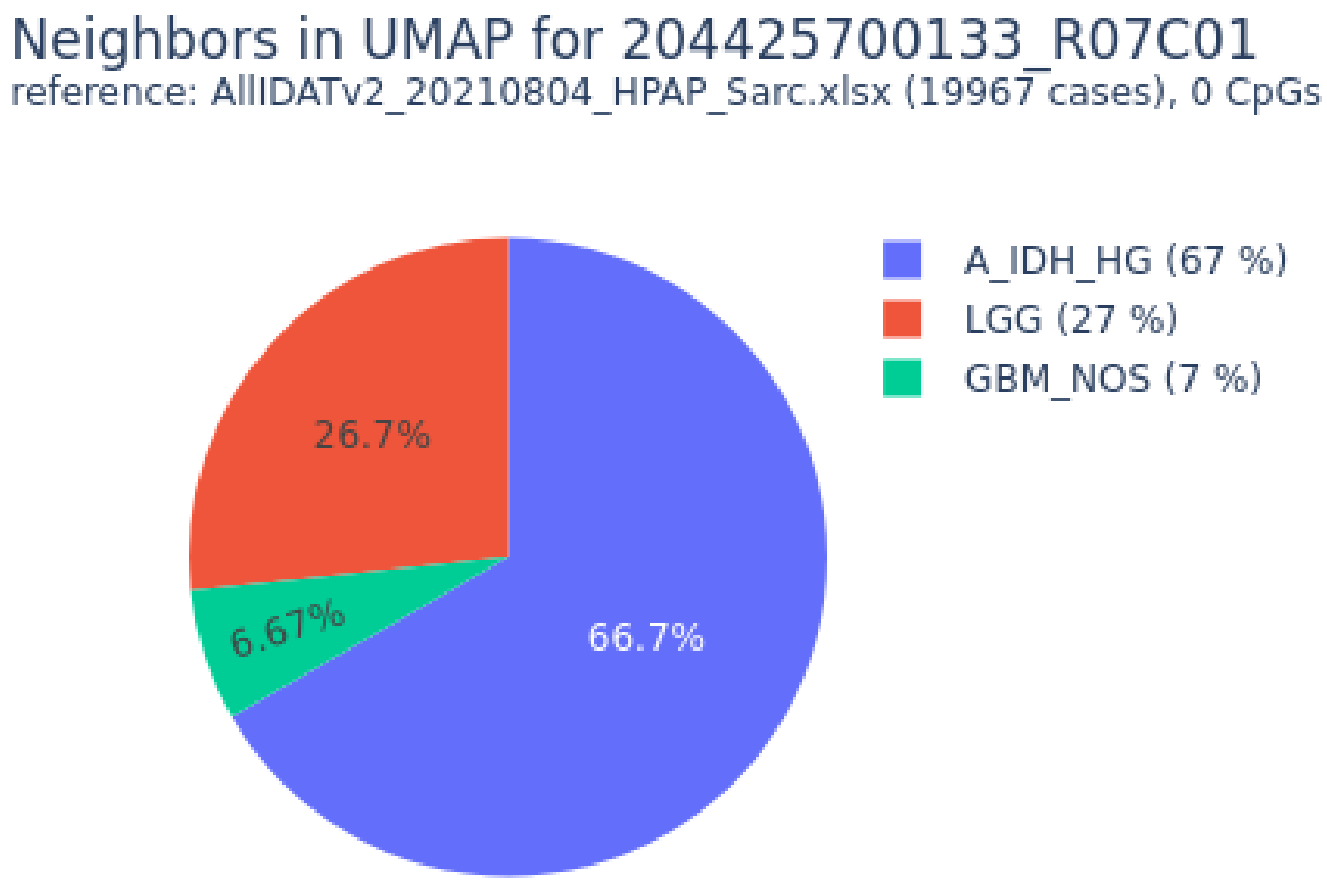

Copy number plot

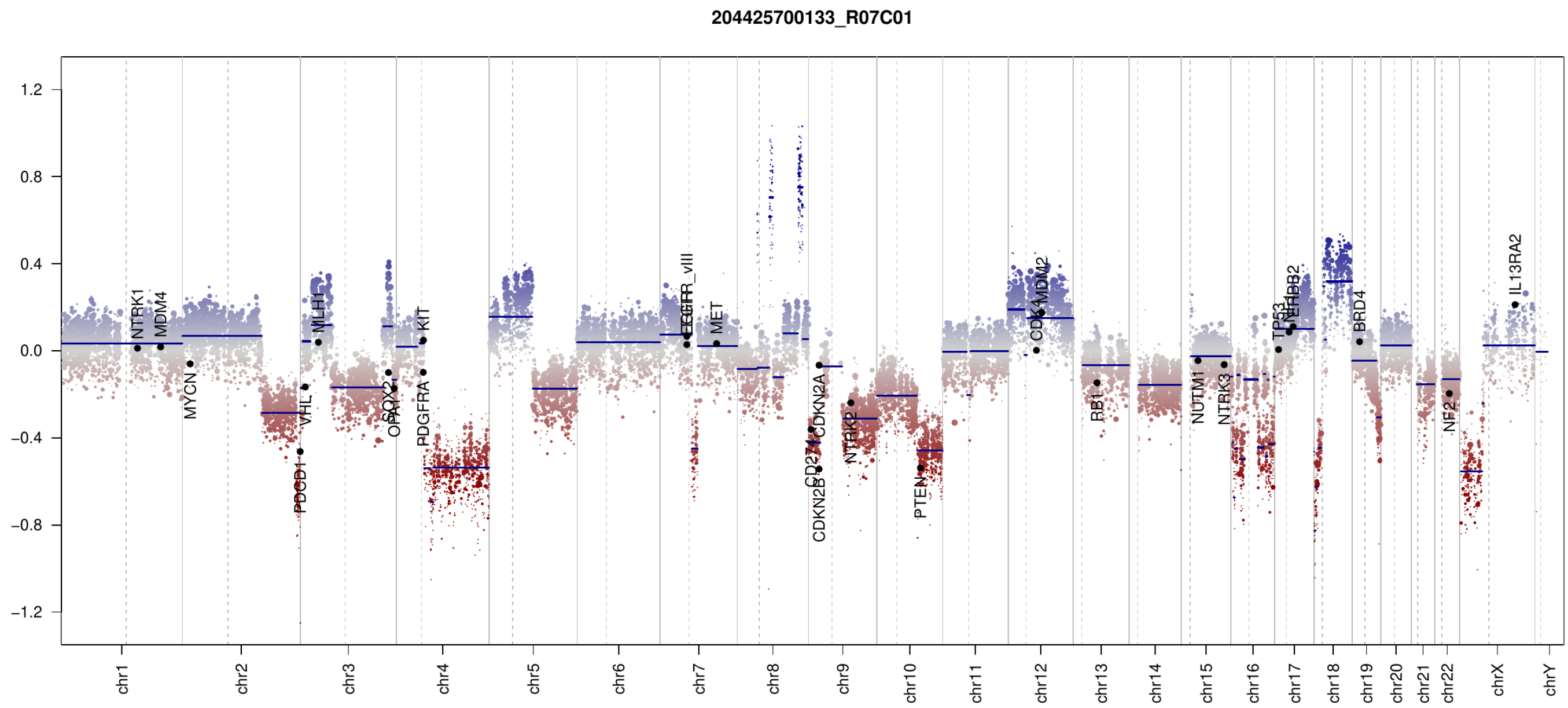

EpiDiP Report for Sample 205046550112_R05C01

Generated on	:	epidip02 / 20260616_172001
Detected barcode	:	undetermined
Valid reads	:	0
450K overlap CpG count	:	0
Reference data	:	AllIDATv2_20210804_HPAP_Sarc

Methylation UMAP plot

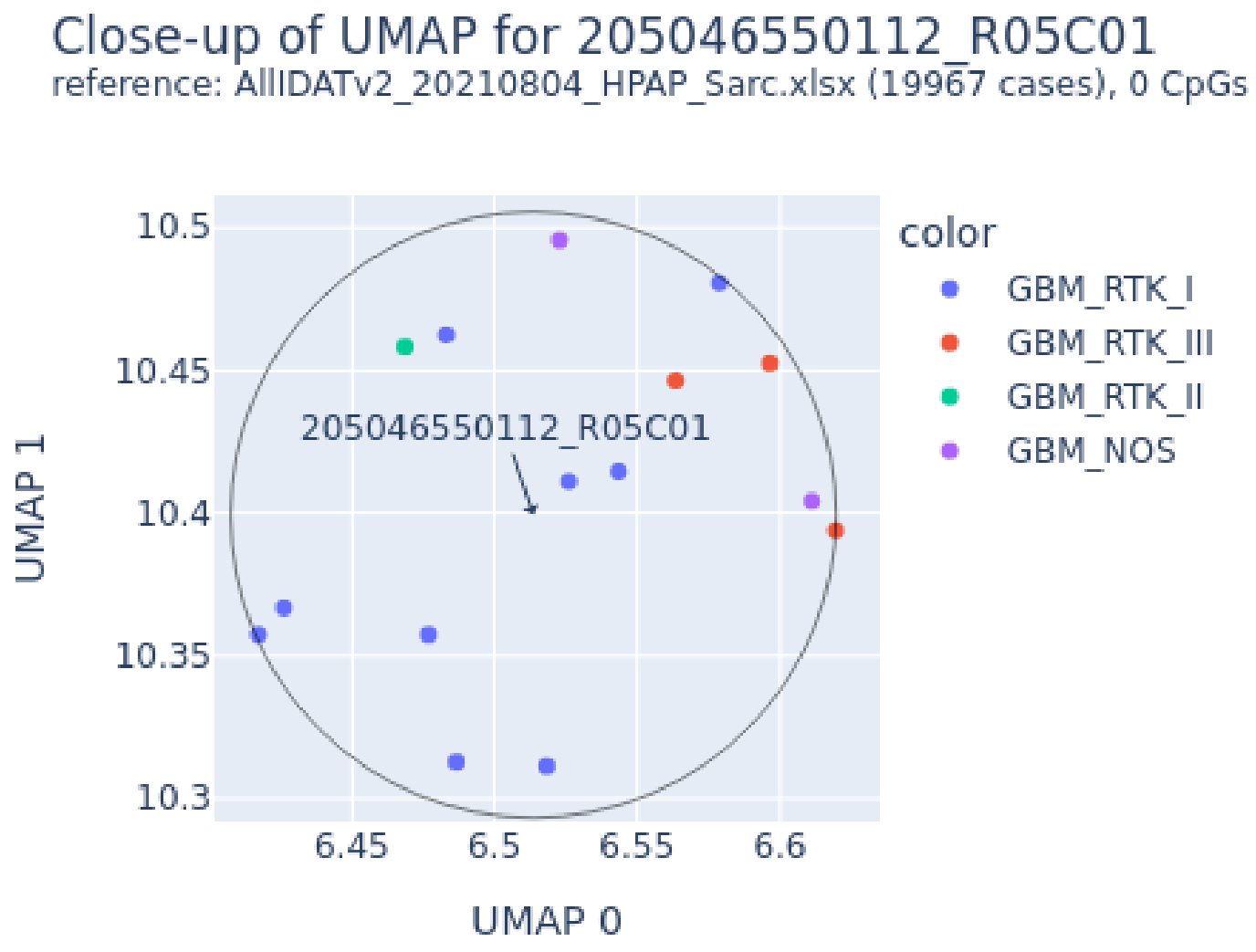

Methylation UMAP Score

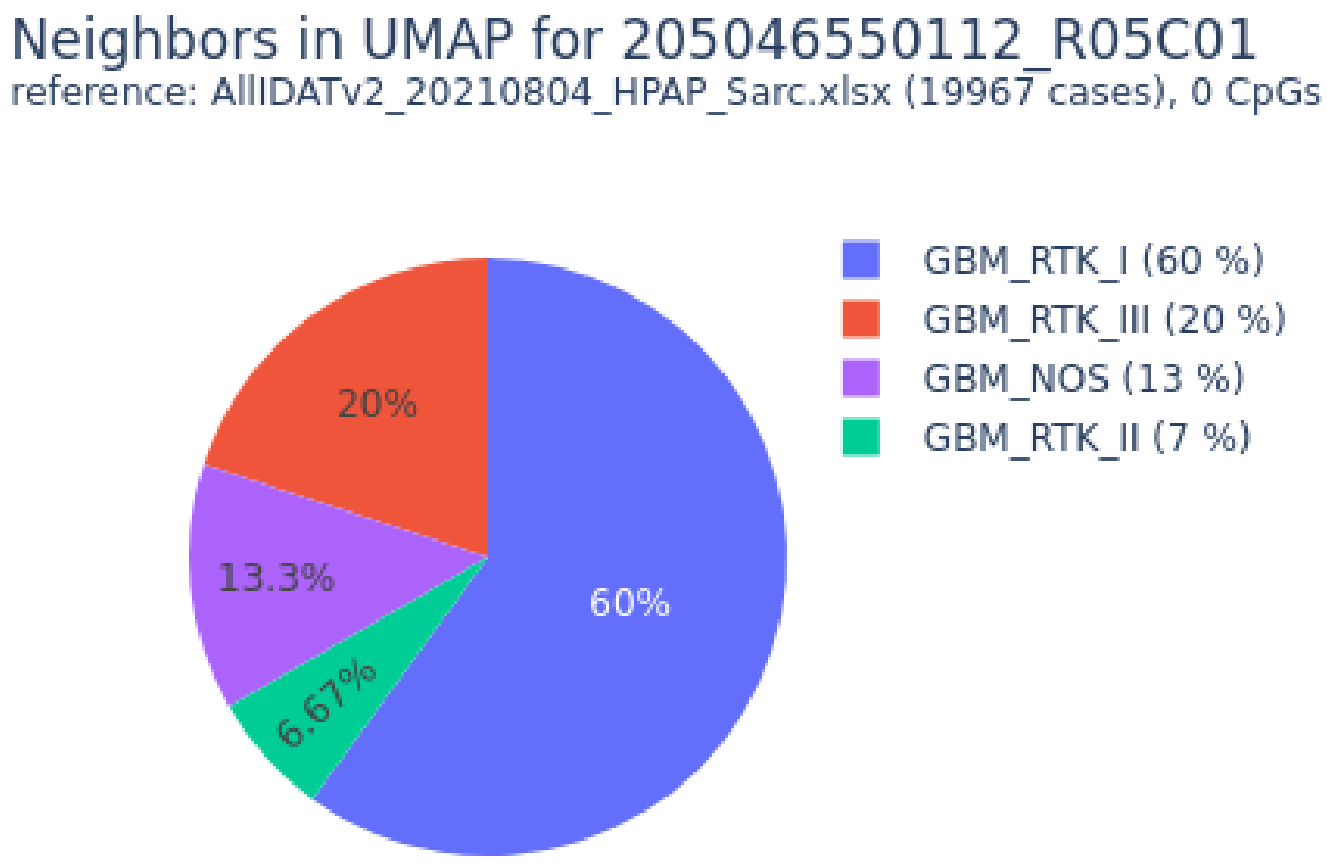

Copy number plot

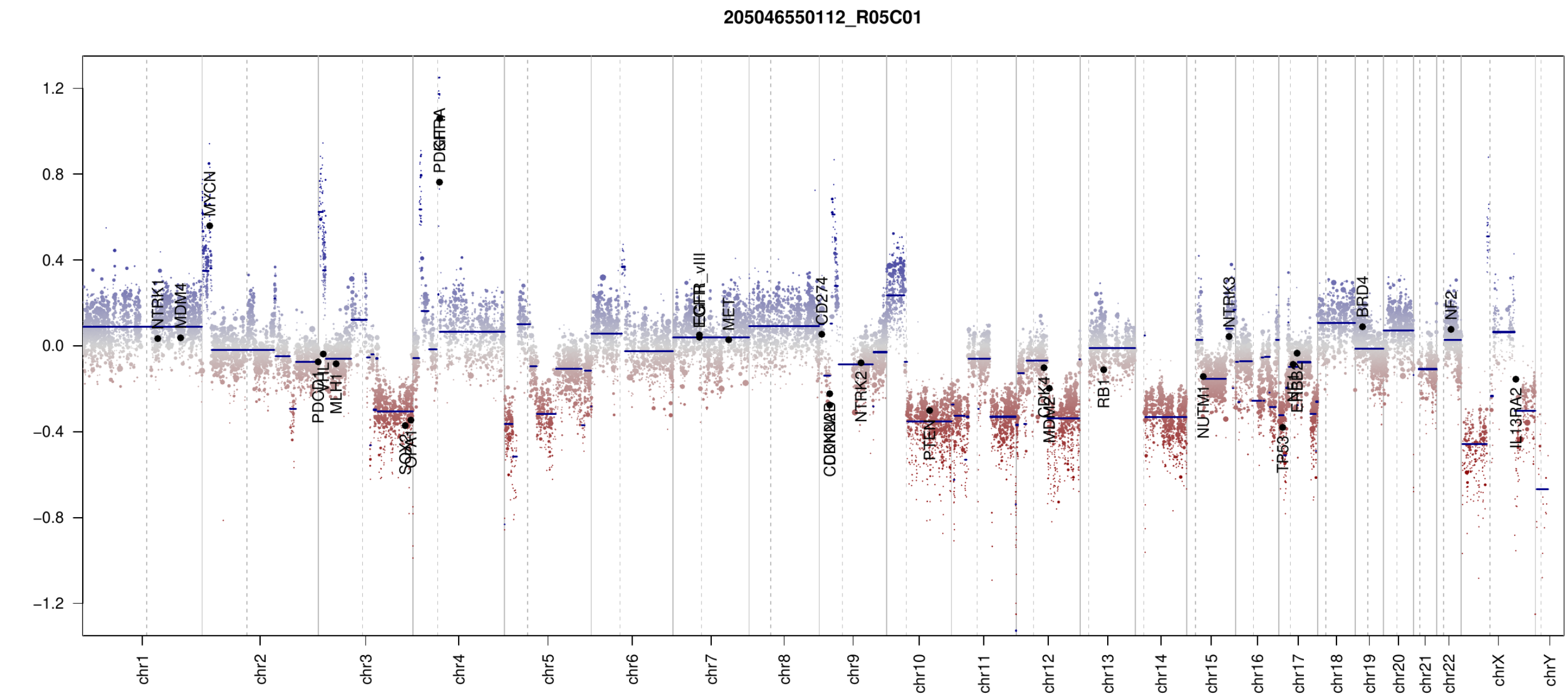

EpiDiP Report for Sample 205310760157_R03C01

Generated on	:	epidip02 / 20260616_170502
Detected barcode	:	undetermined
Valid reads	:	0
450K overlap CpG count	:	0
Reference data	:	AllIDATv2_20210804_HPAP_Sarc

Methylation UMAP plot

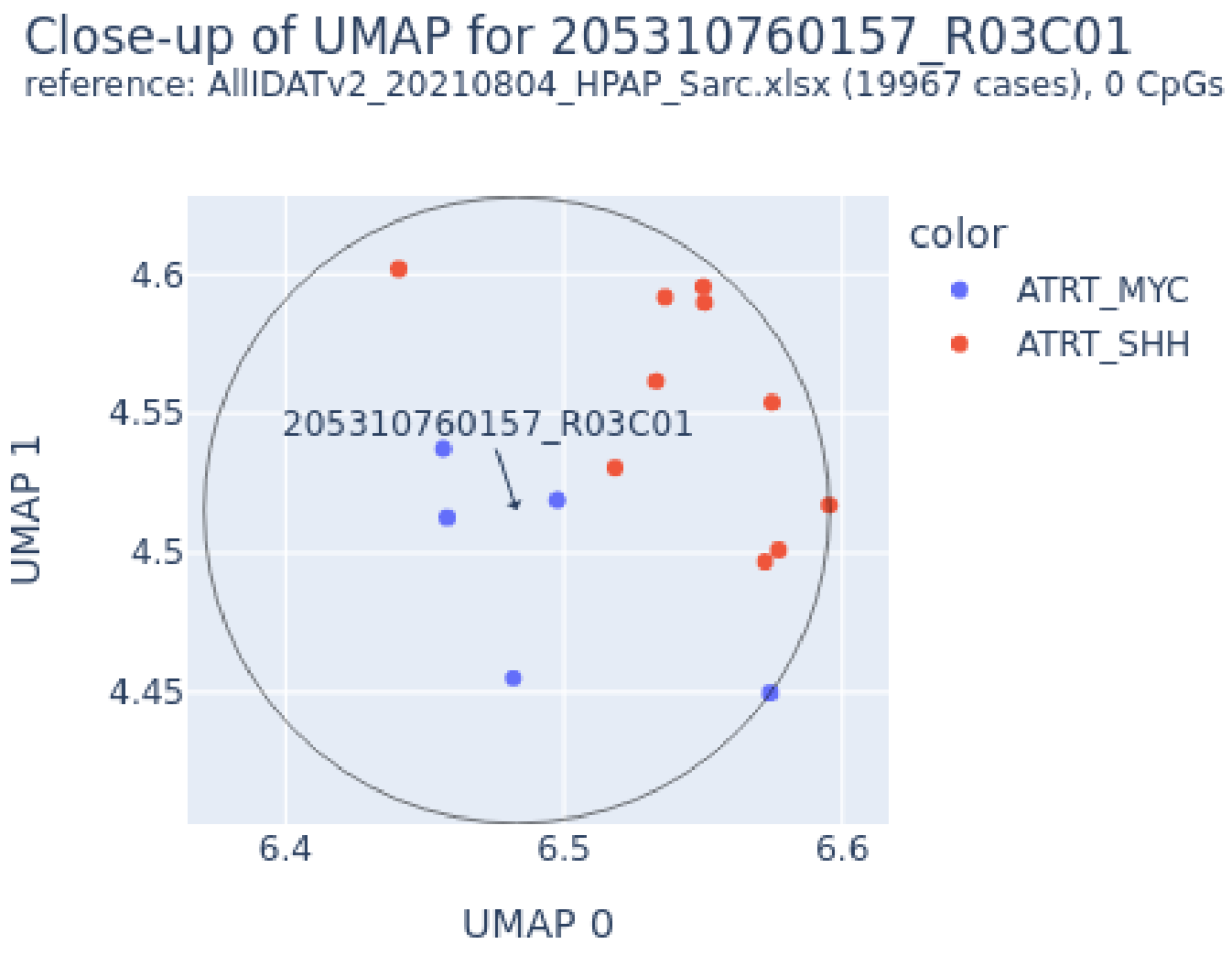

Methylation UMAP Score

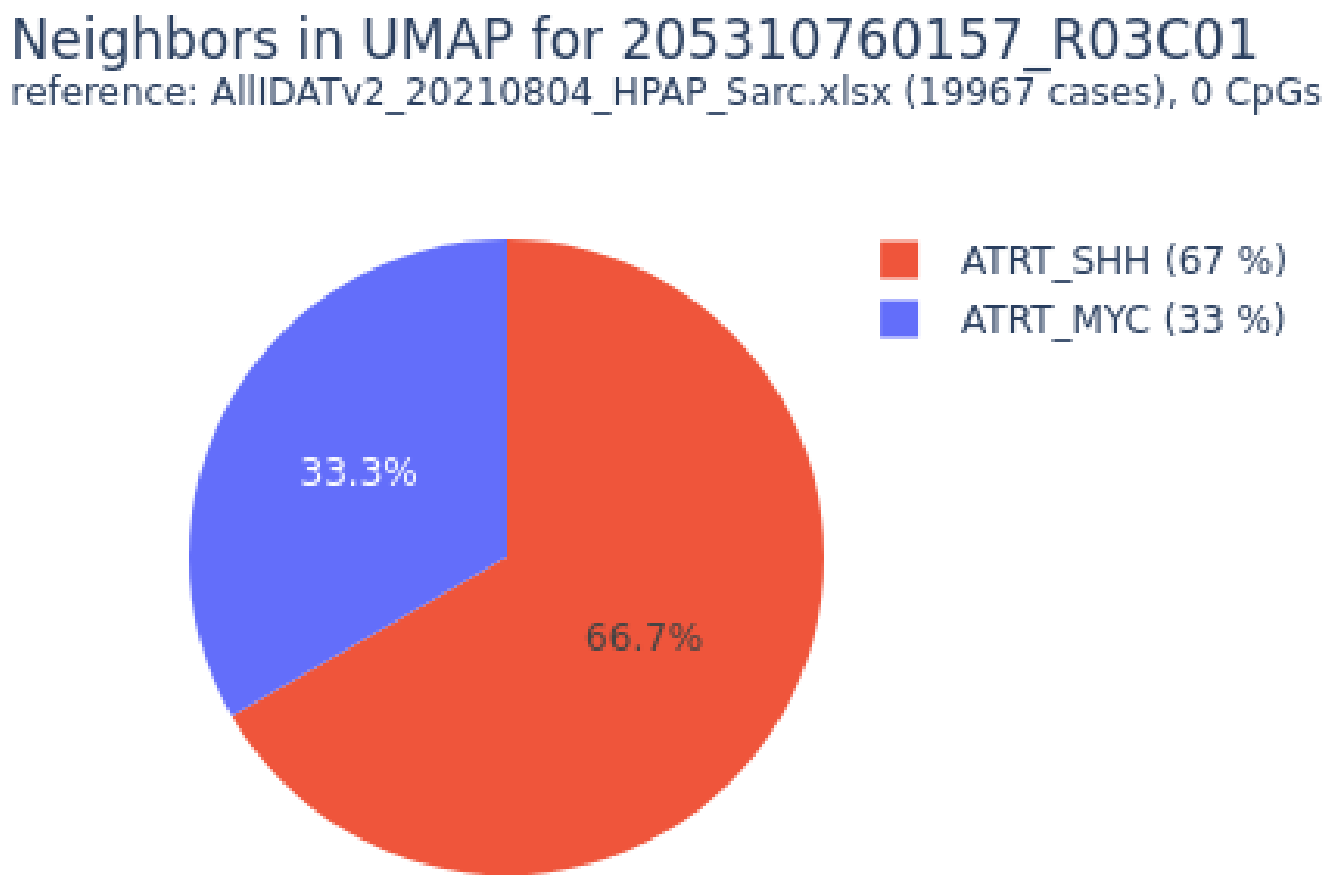

Copy number plot

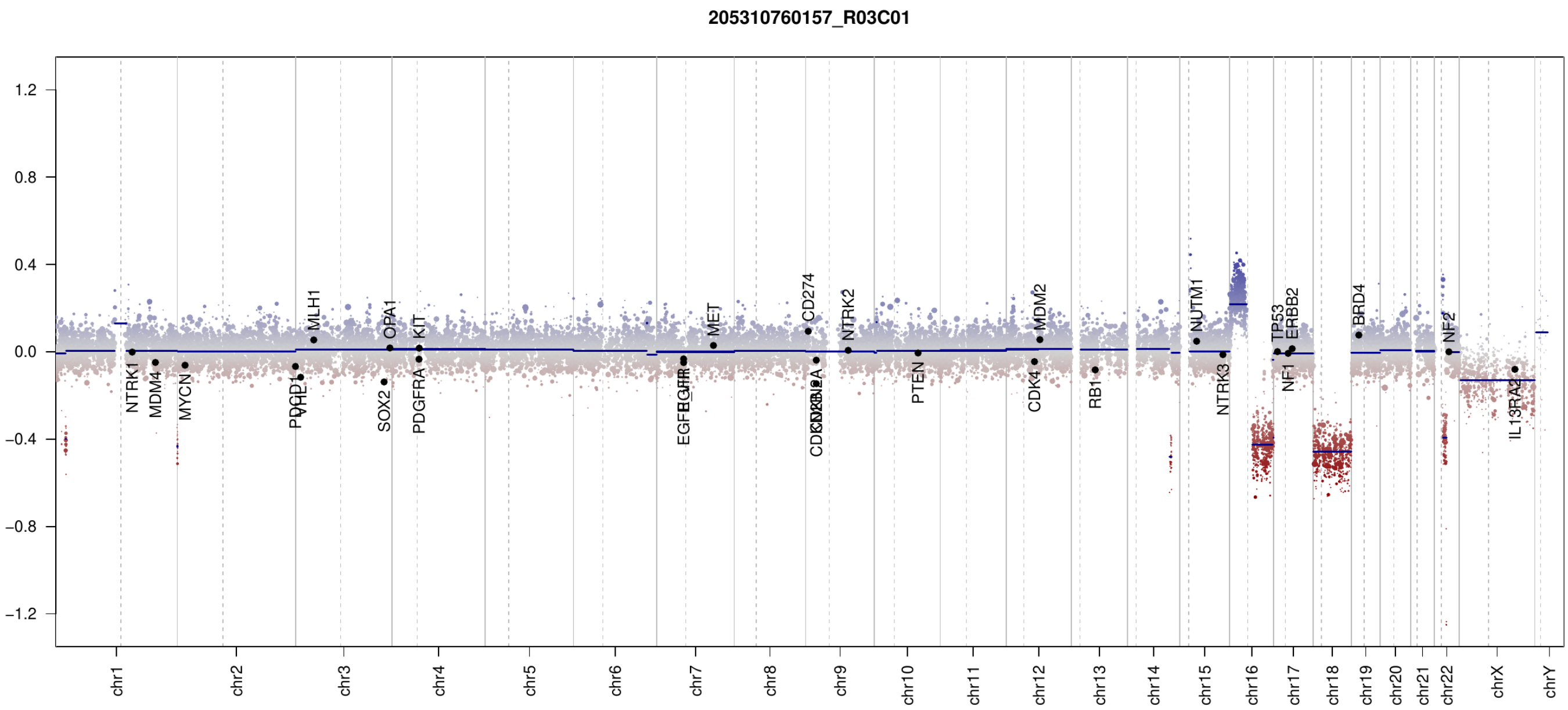

EpiDiP Report for Sample 205495890138_R06C01

Generated on	:	epidip02 / 20260616_172408
Detected barcode	:	undetermined
Valid reads	:	0
450K overlap CpG count	:	0
Reference data	:	AllIDATv2_20210804_HPAP_Sarc

Methylation UMAP plot

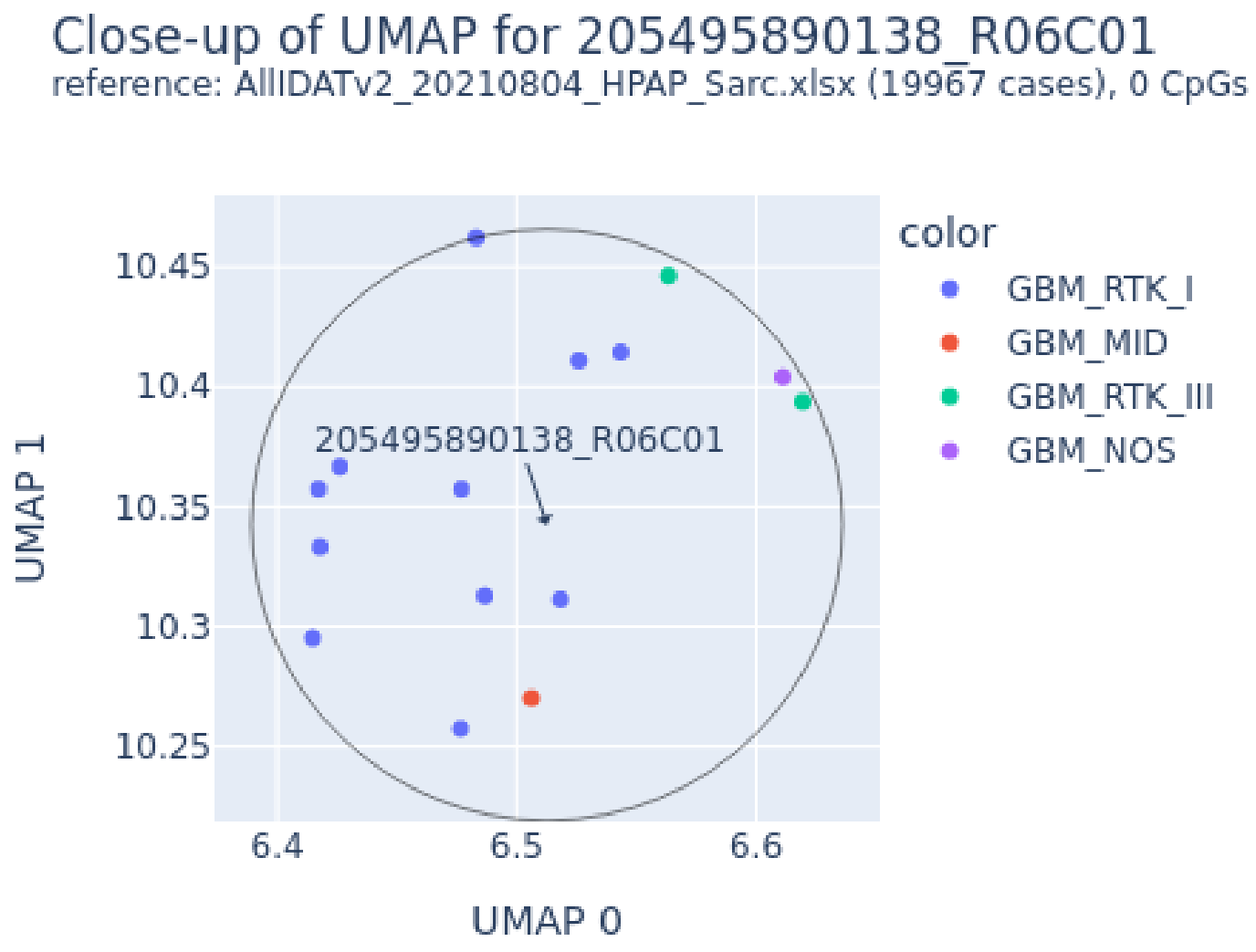

Methylation UMAP Score

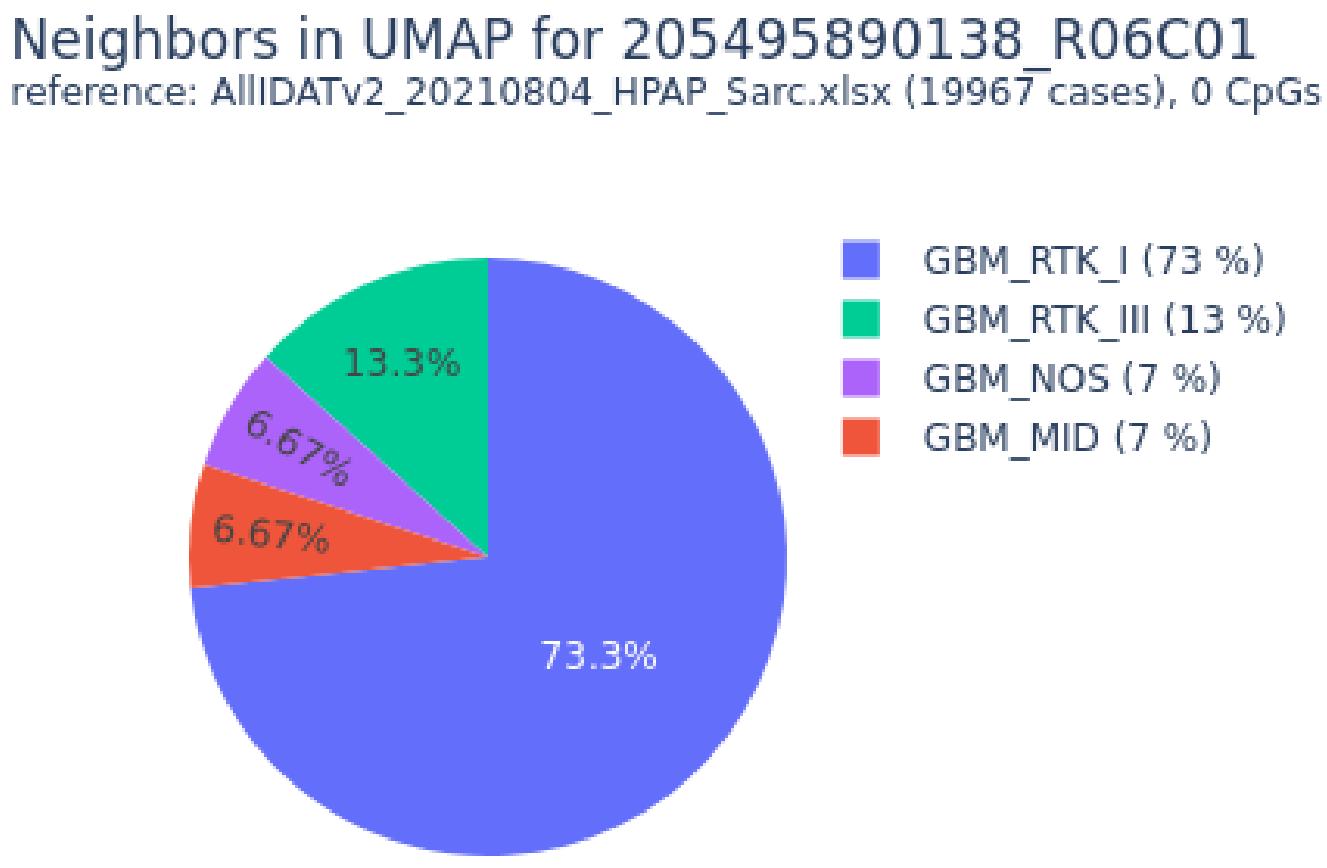

Copy number plot

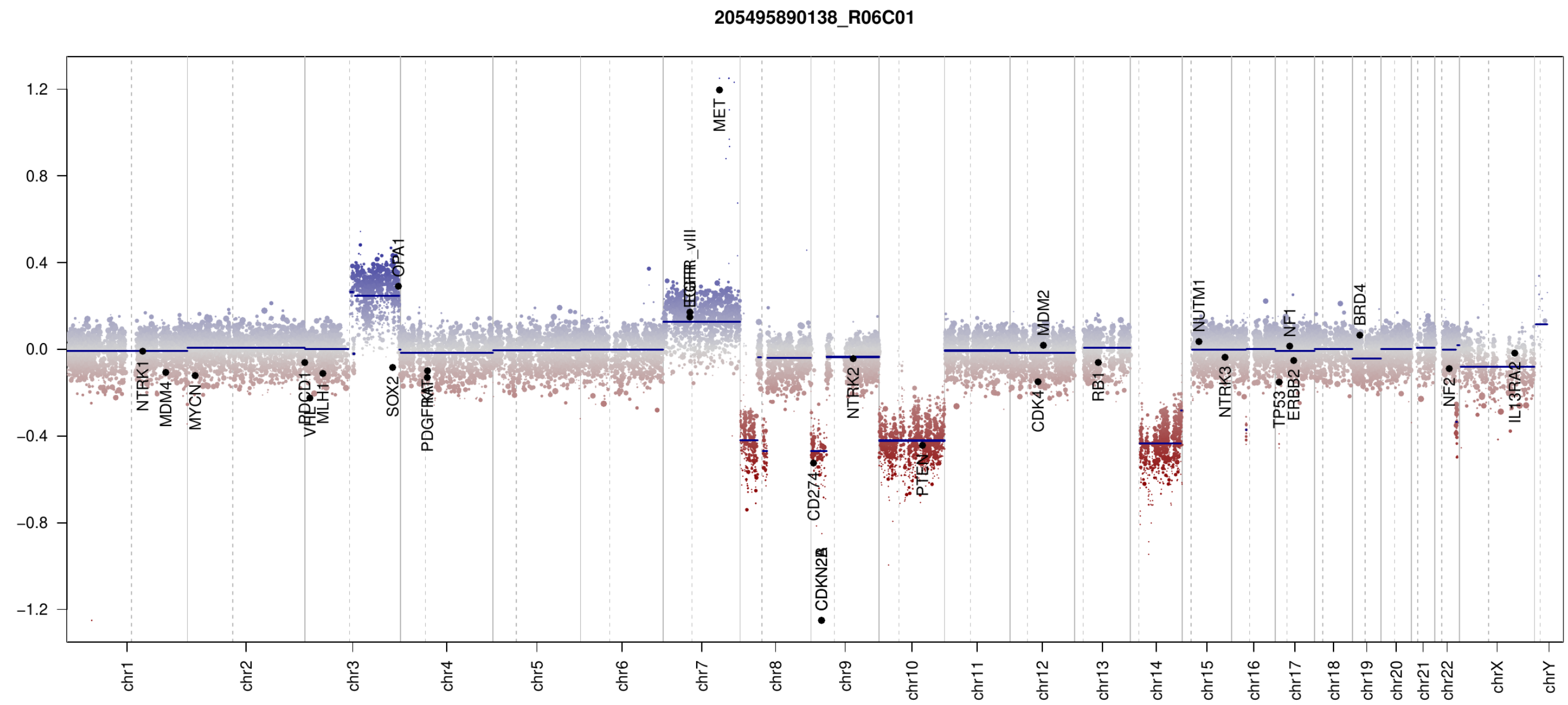

EpiDiP Report for Sample 205814880164_R06C01

Generated on	:	epidip02 / 20260616_171759
Detected barcode	:	undetermined
Valid reads	:	0
450K overlap CpG count	:	0
Reference data	:	AllIDATv2_20210804_HPAP_Sarc

Methylation UMAP plot

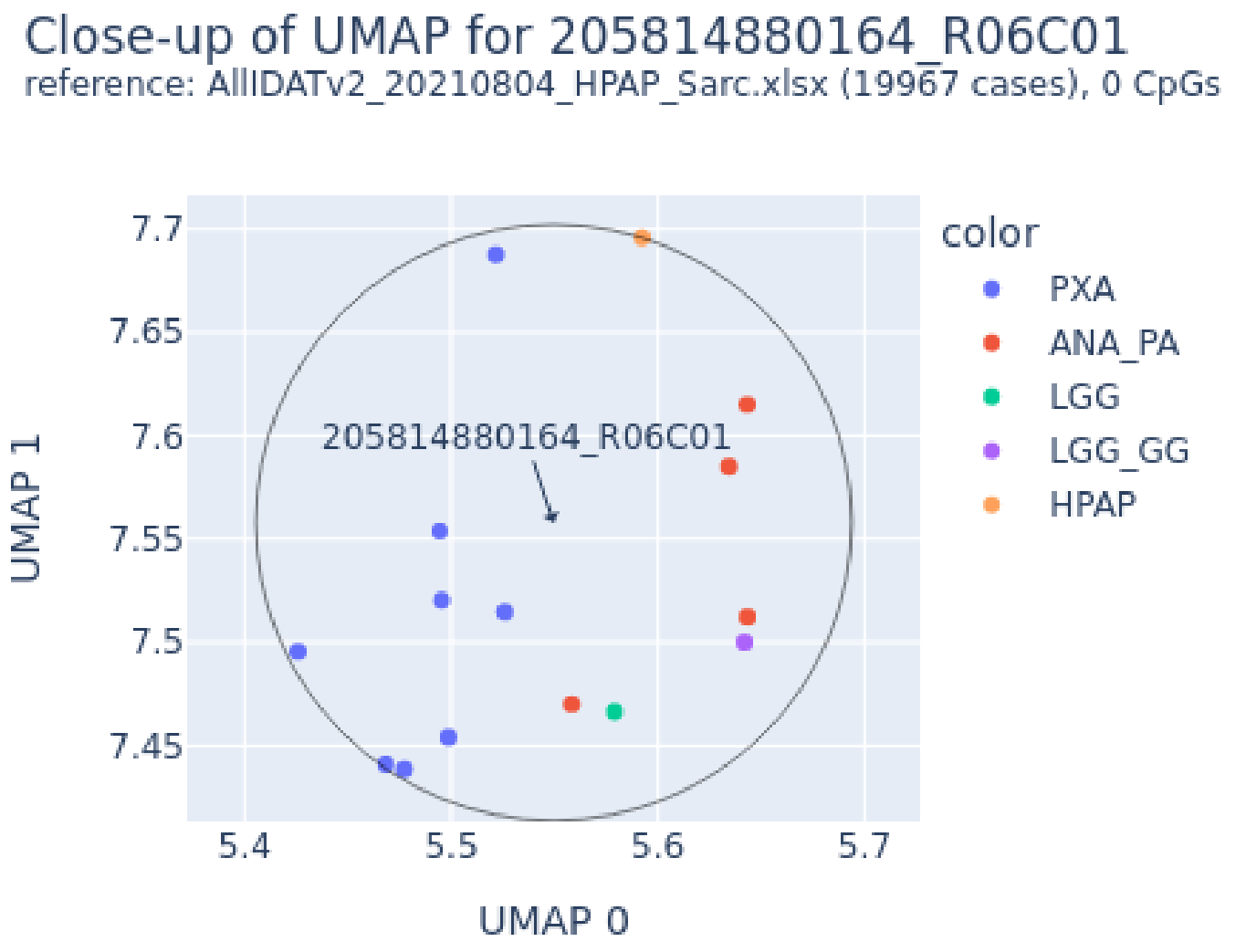

Methylation UMAP Score

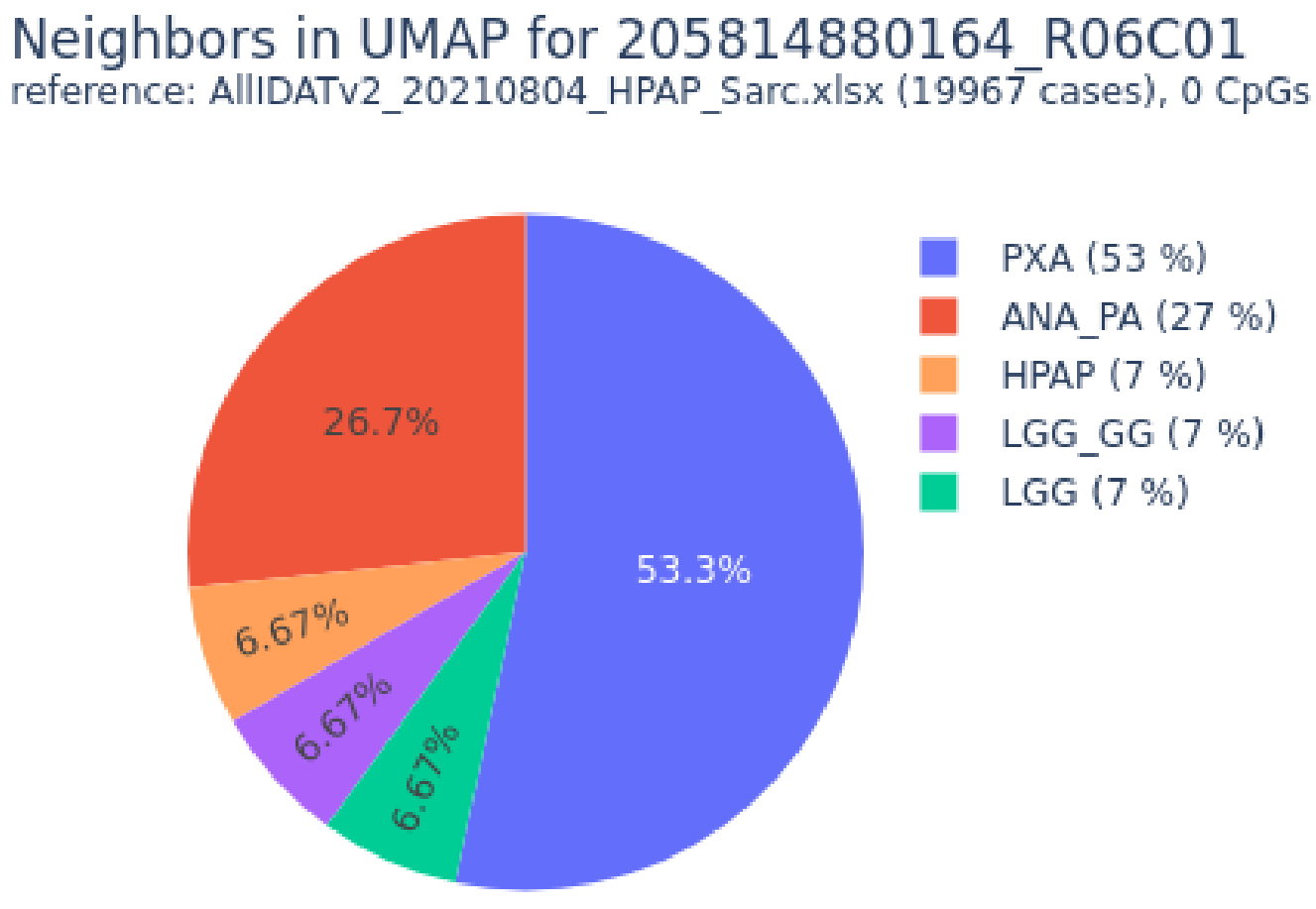

Copy number plot

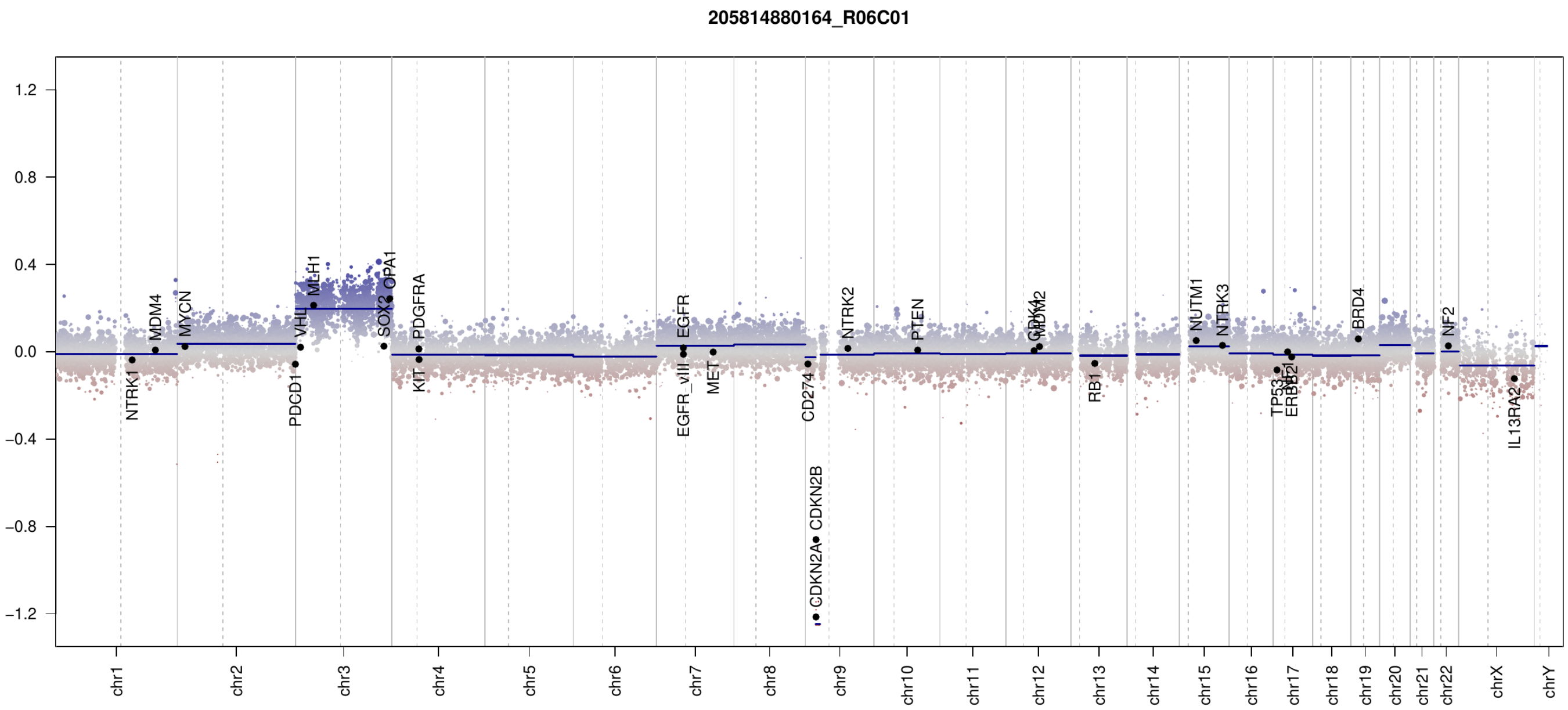

EpiDiP Report for Sample 205828600180_R05C01

Generated on	:	epidip02 / 20260616_171541
Detected barcode	:	undetermined
Valid reads	:	0
450K overlap CpG count	:	0
Reference data	:	AllIDATv2_20210804_HPAP_Sarc

Methylation UMAP plot

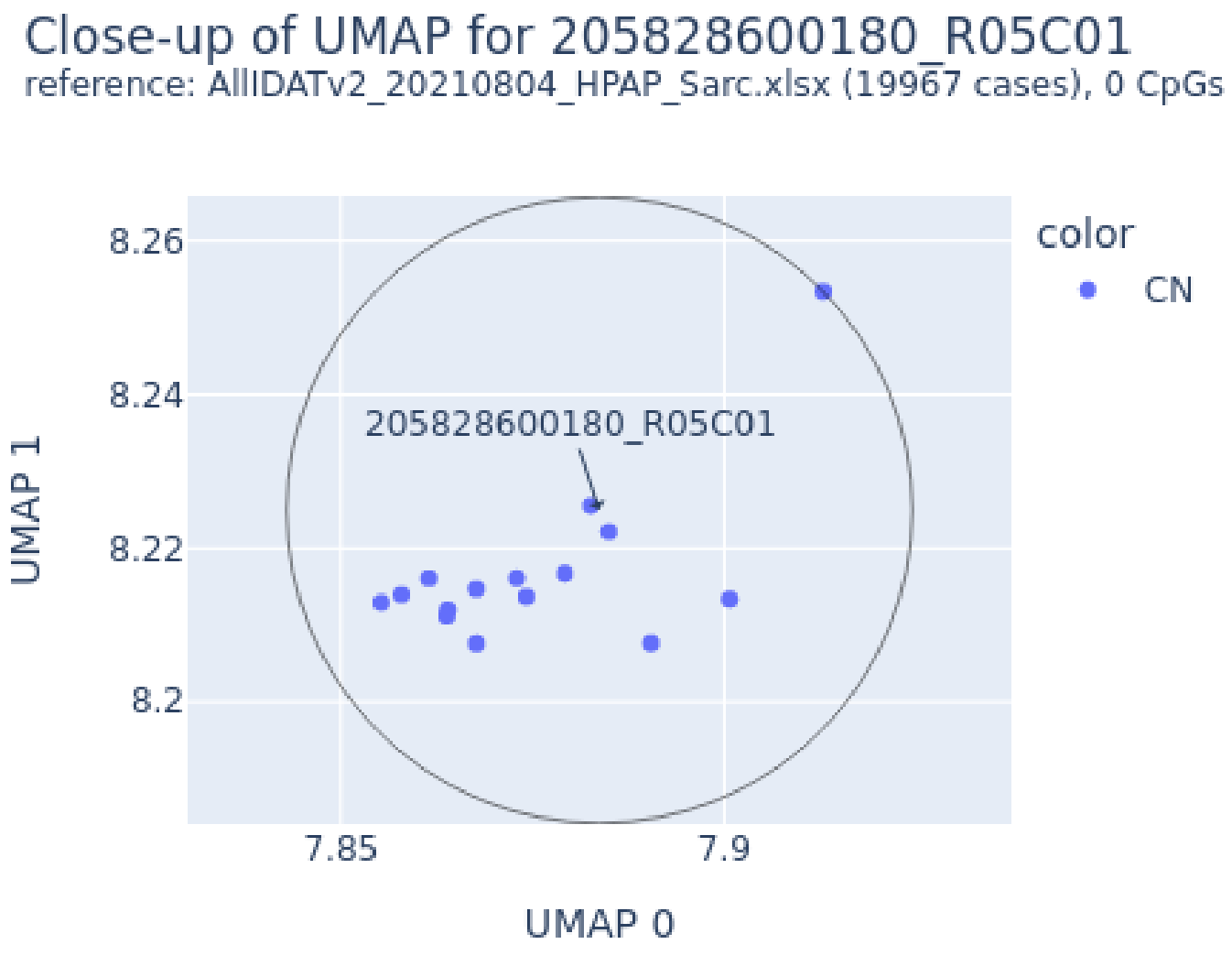

Methylation UMAP Score

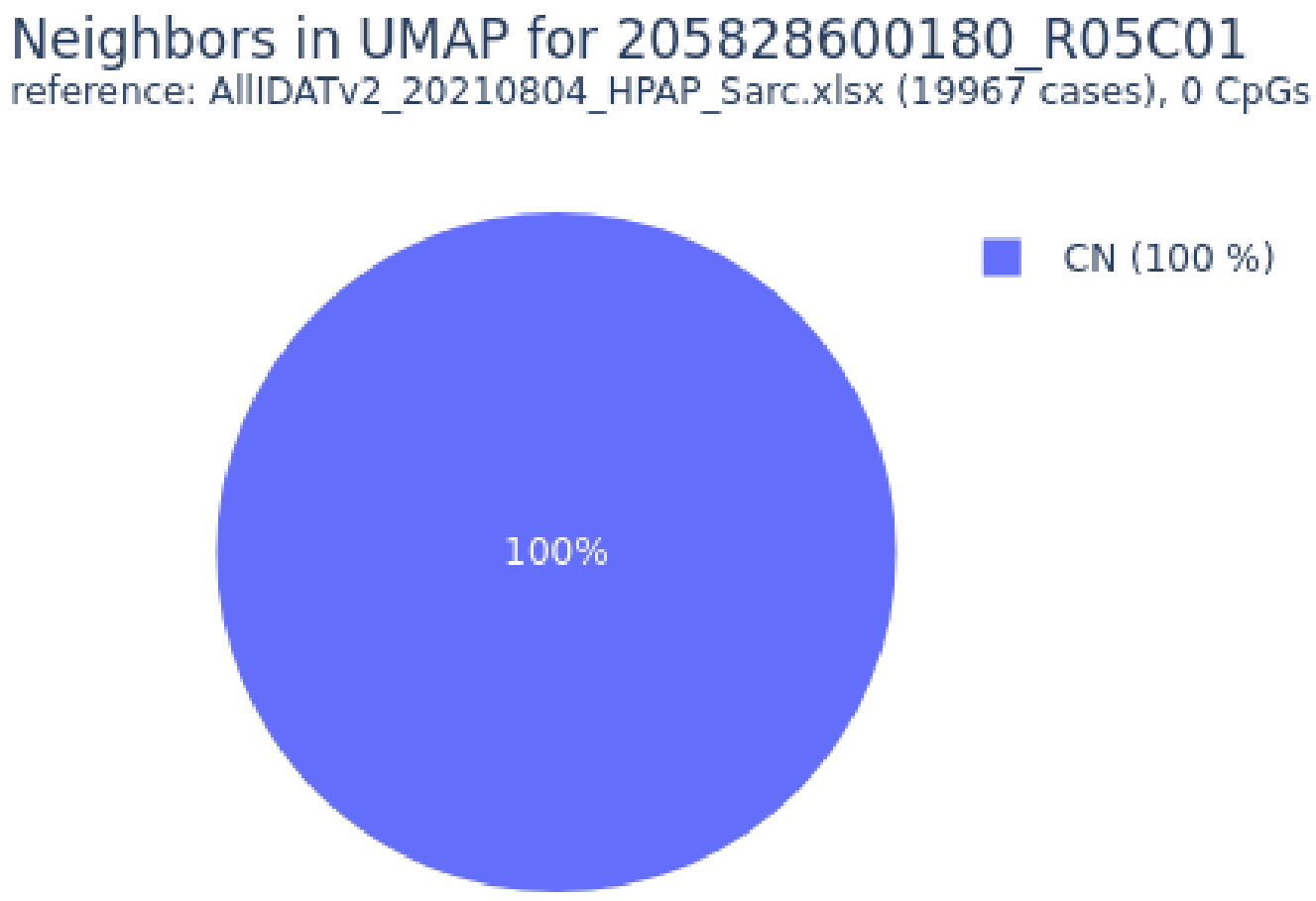

Copy number plot

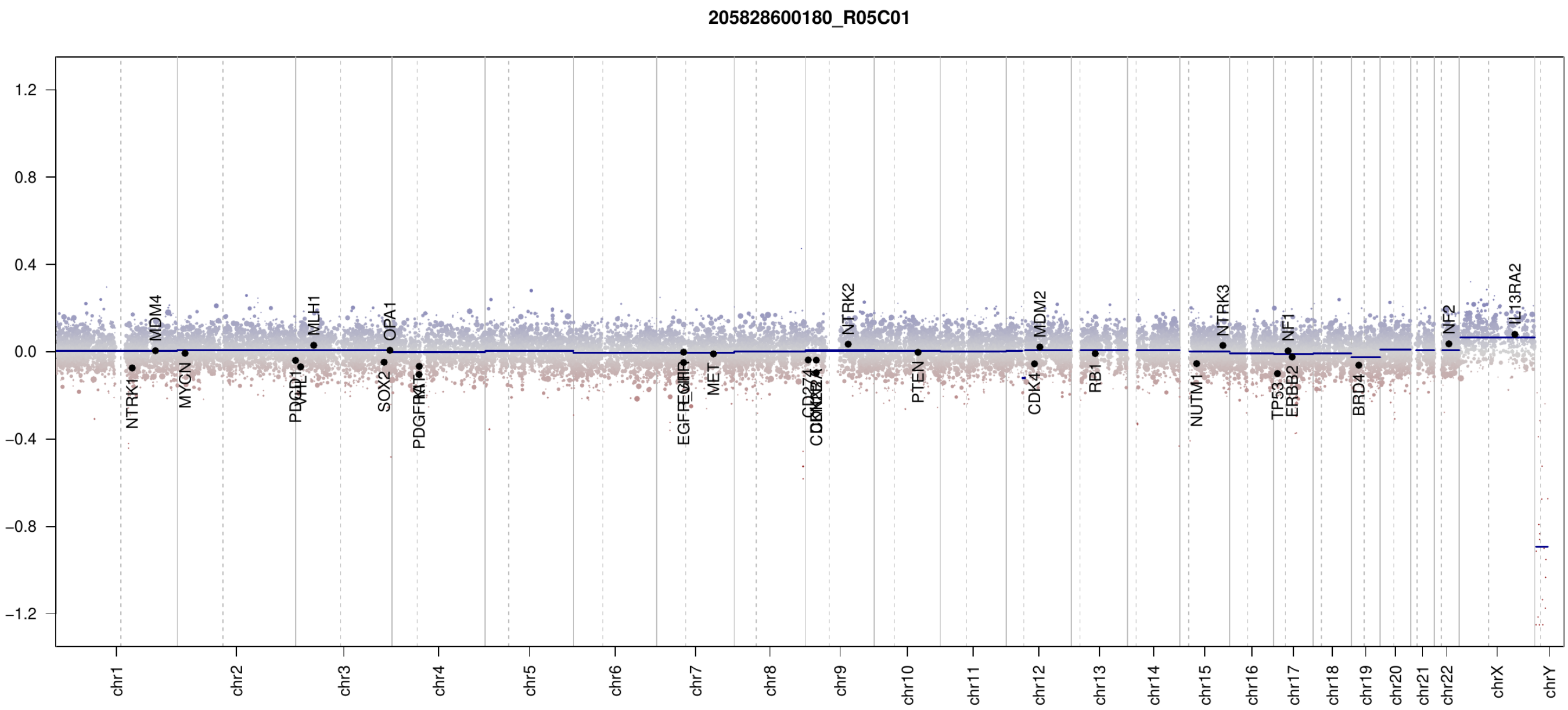

EpiDiP Report for Sample 205862290139_R04C01

Generated on	:	epidip02 / 20260616_171200
Detected barcode	:	undetermined
Valid reads	:	0
450K overlap CpG count	:	0
Reference data	:	AllIDATv2_20210804_HPAP_Sarc

Methylation UMAP plot

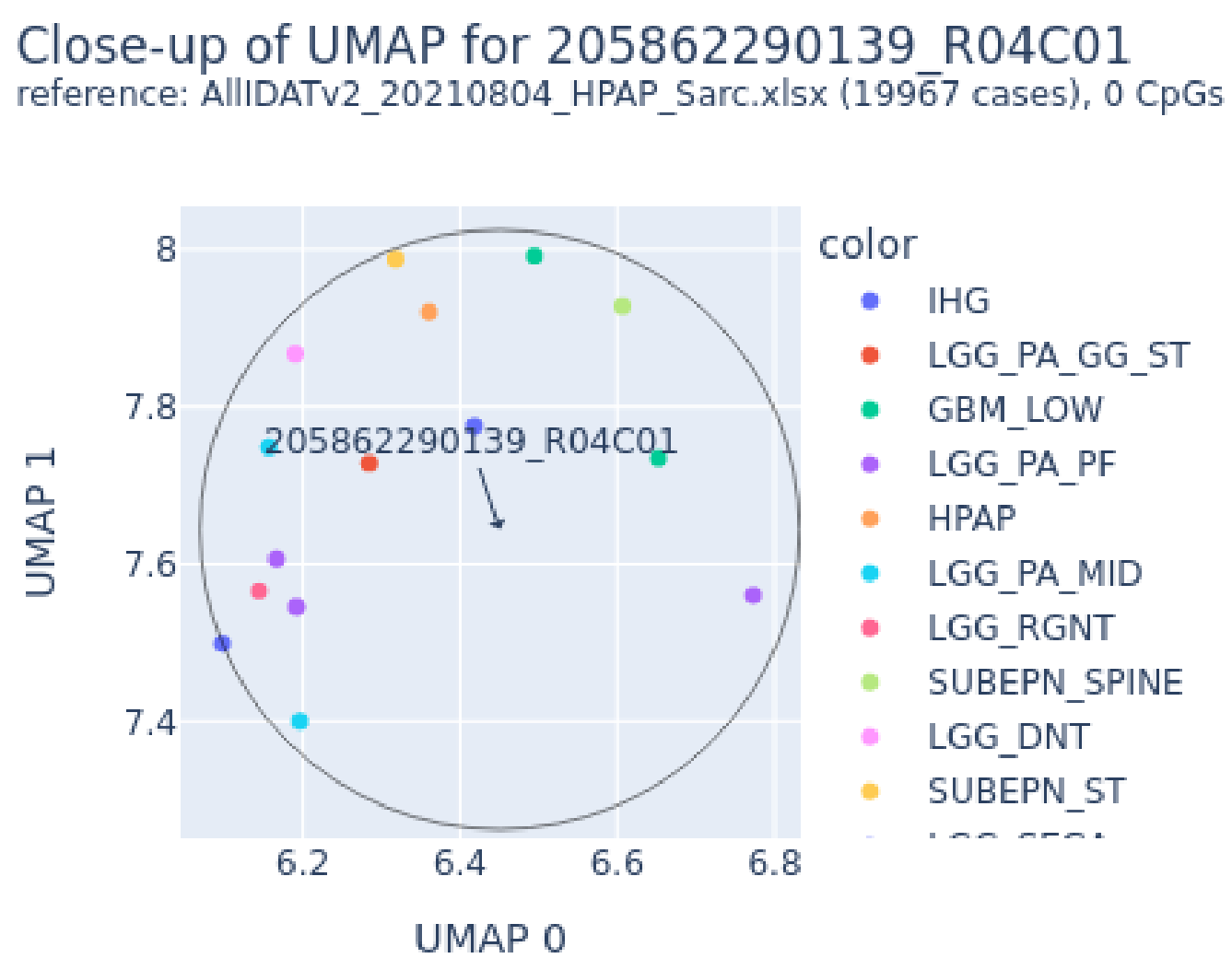

Methylation UMAP Score

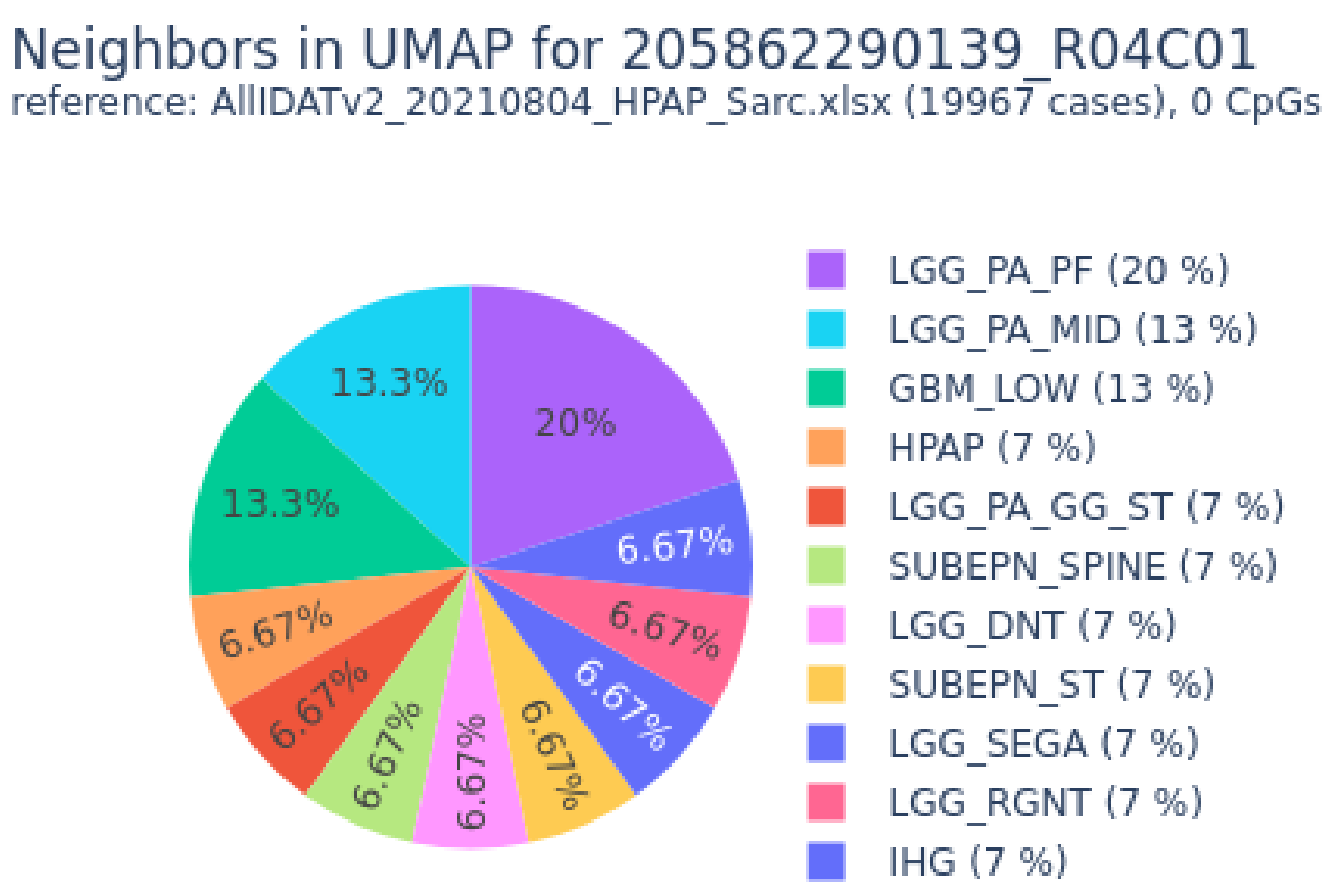

Copy number plot

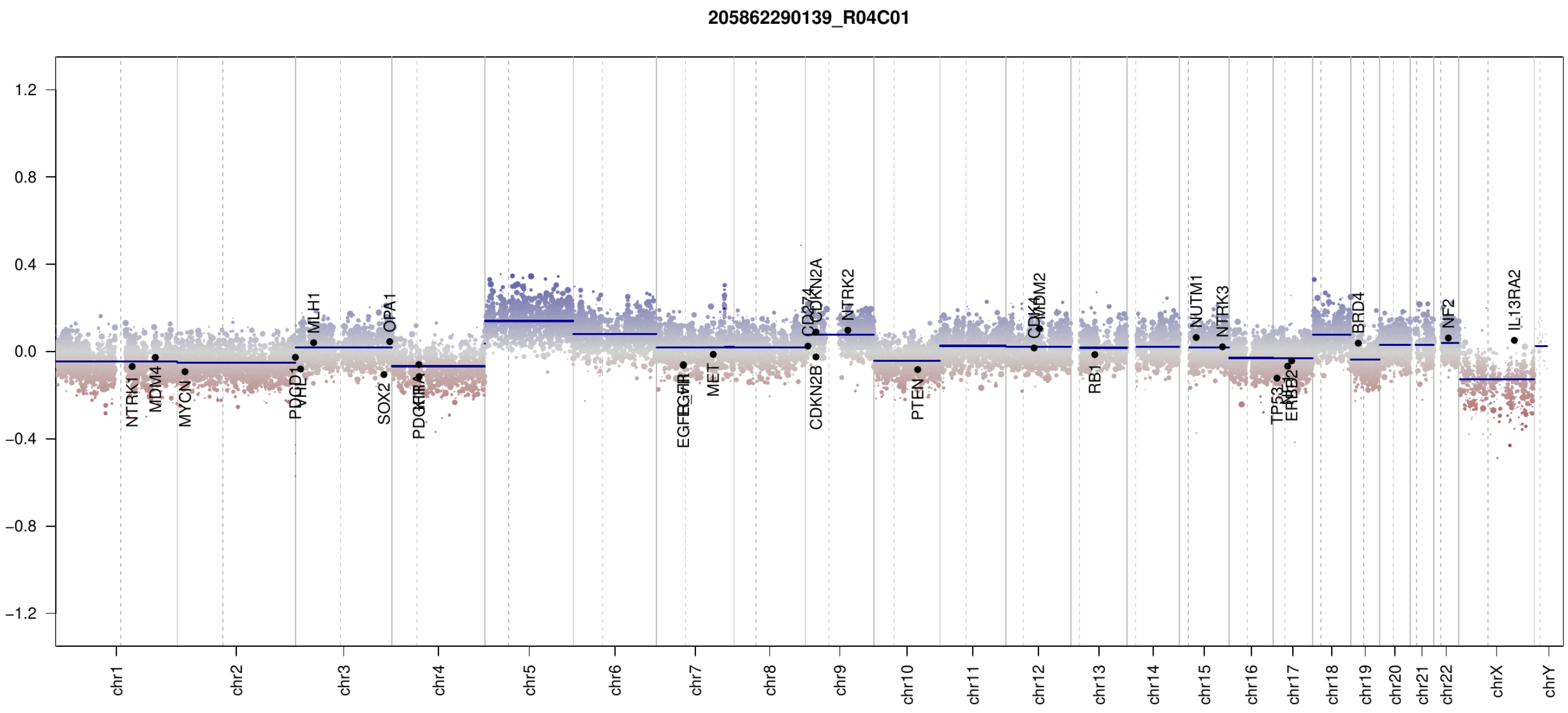

EpiDiP Report for Sample 206439840021_R04C01

Generated on	:	epidip02 / 20260616_171830
Detected barcode	:	undetermined
Valid reads	:	0
450K overlap CpG count	:	0
Reference data	:	AllIDATv2_20210804_HPAP_Sarc

Methylation UMAP plot

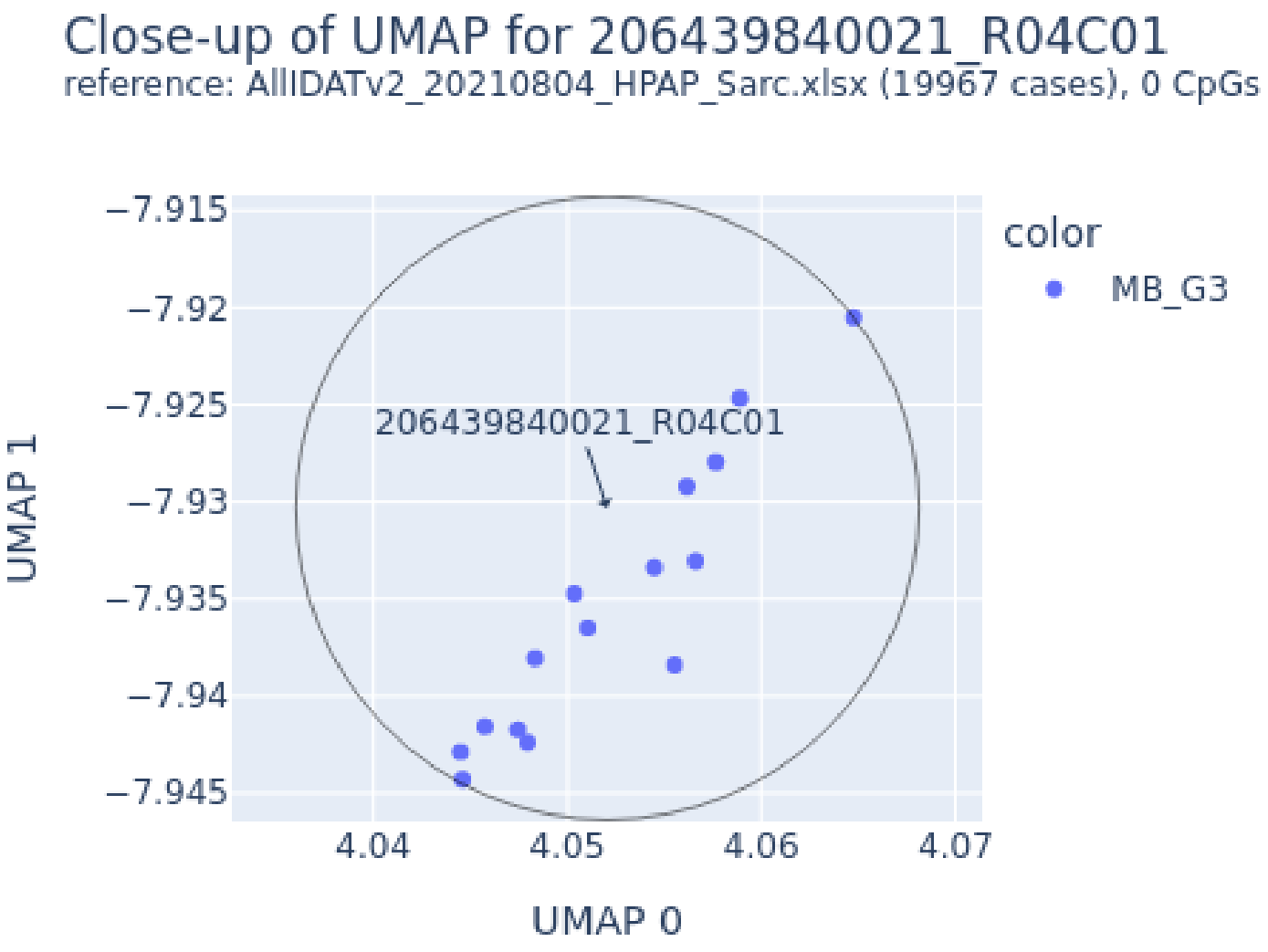

Methylation UMAP Score

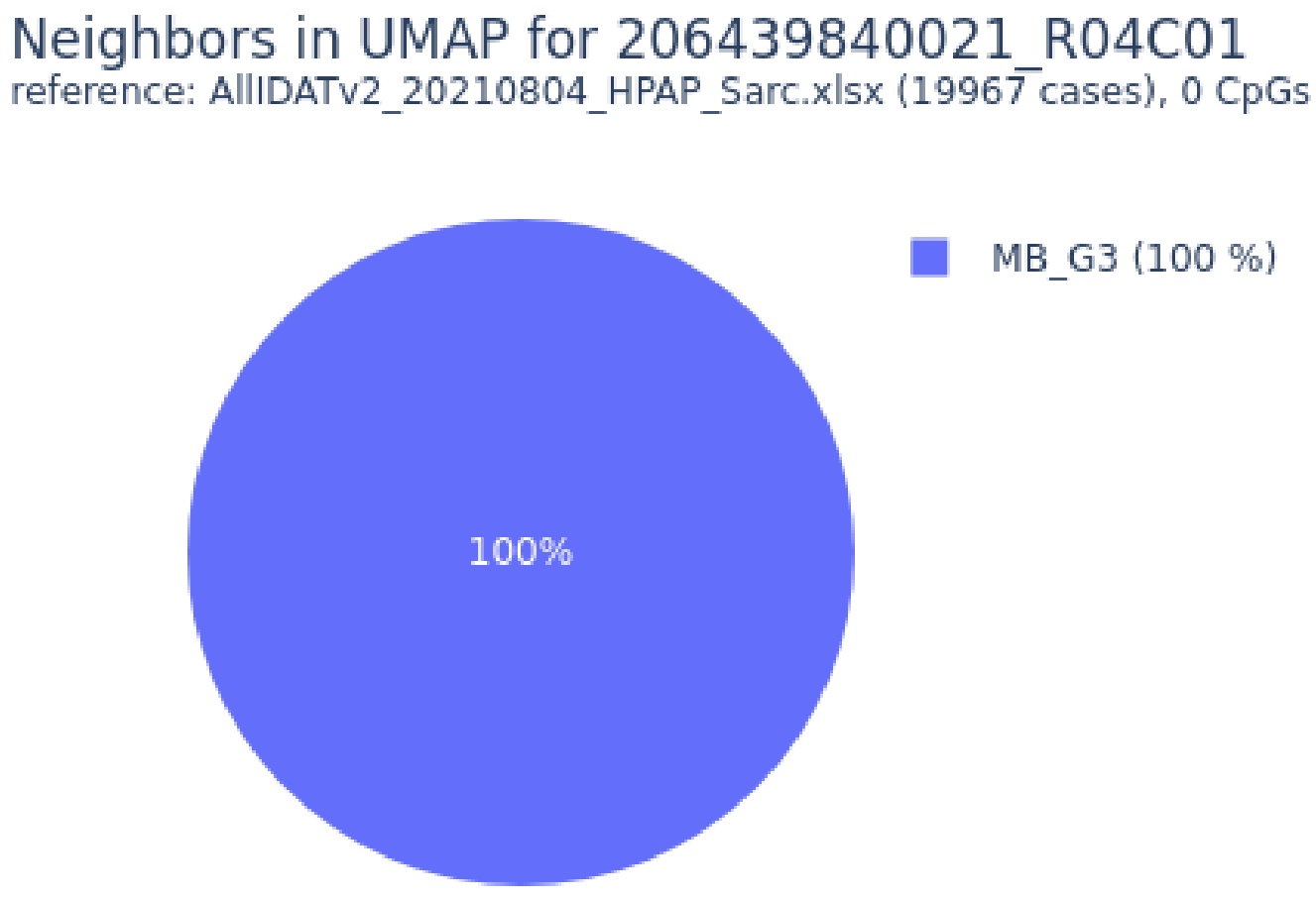

Copy number plot

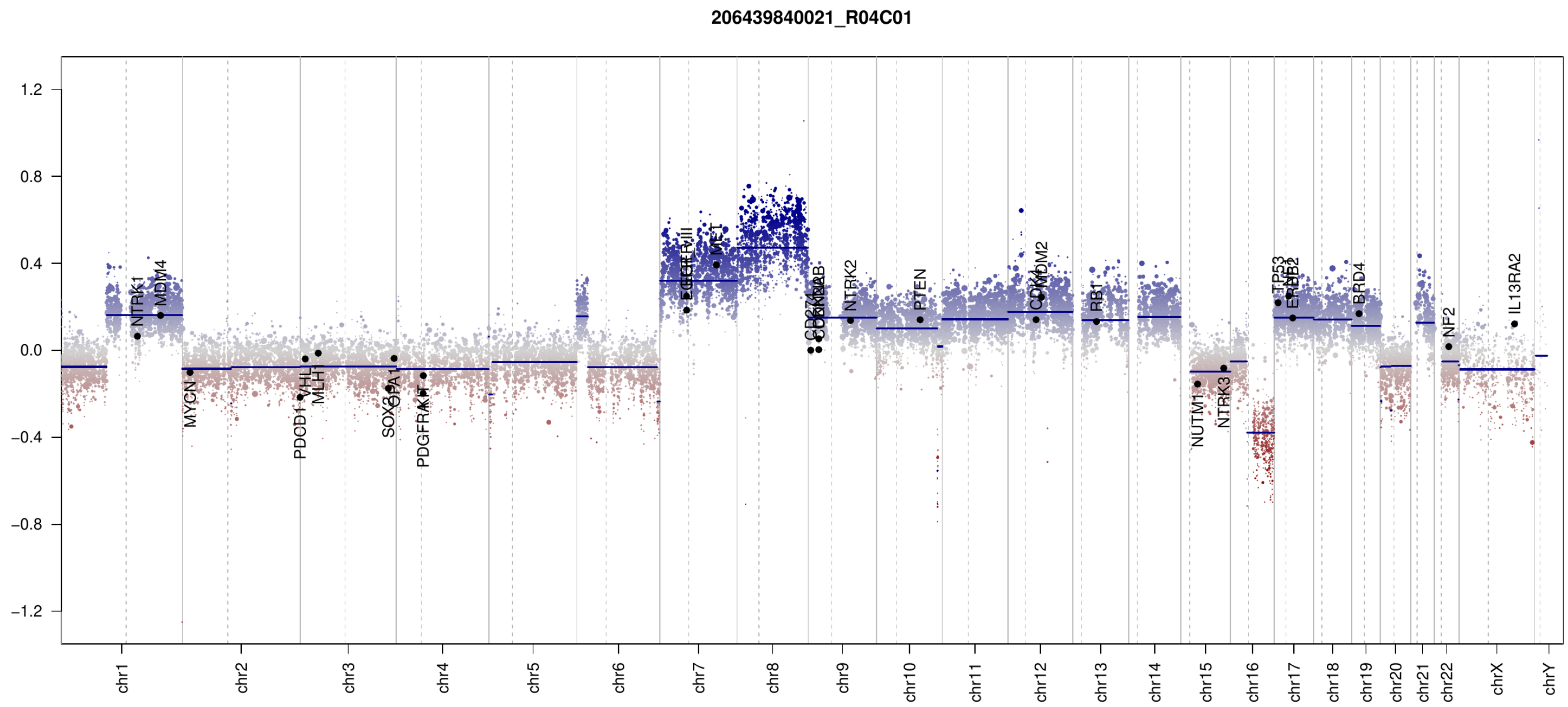

EpiDiP Report for Sample 206486310052_R06C01

Generated on	:	epidip02 / 20260616_172908
Detected barcode	:	undetermined
Valid reads	:	0
450K overlap CpG count	:	0
Reference data	:	AllIDATv2_20210804_HPAP_Sarc

Methylation UMAP plot

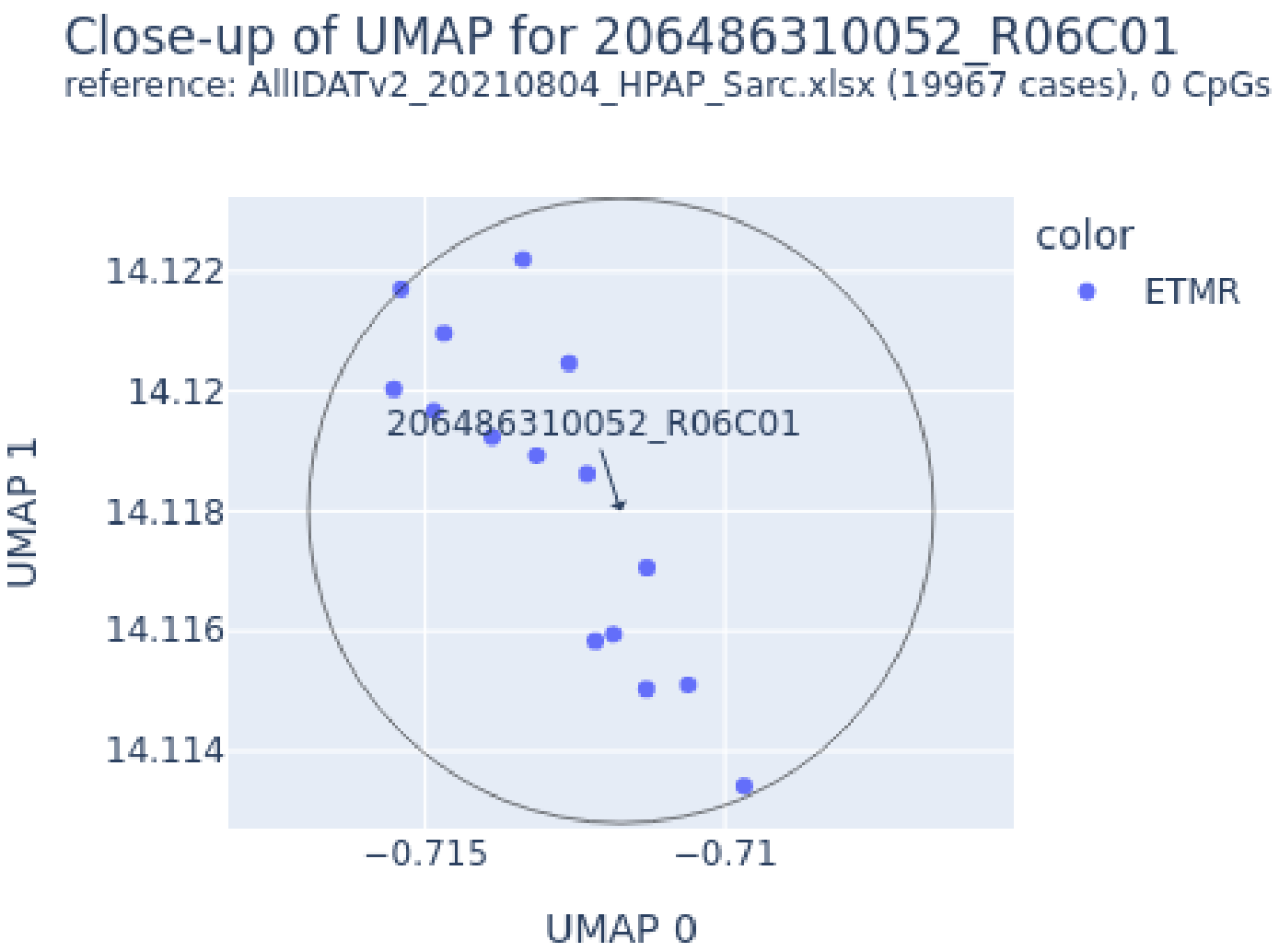

Methylation UMAP Score

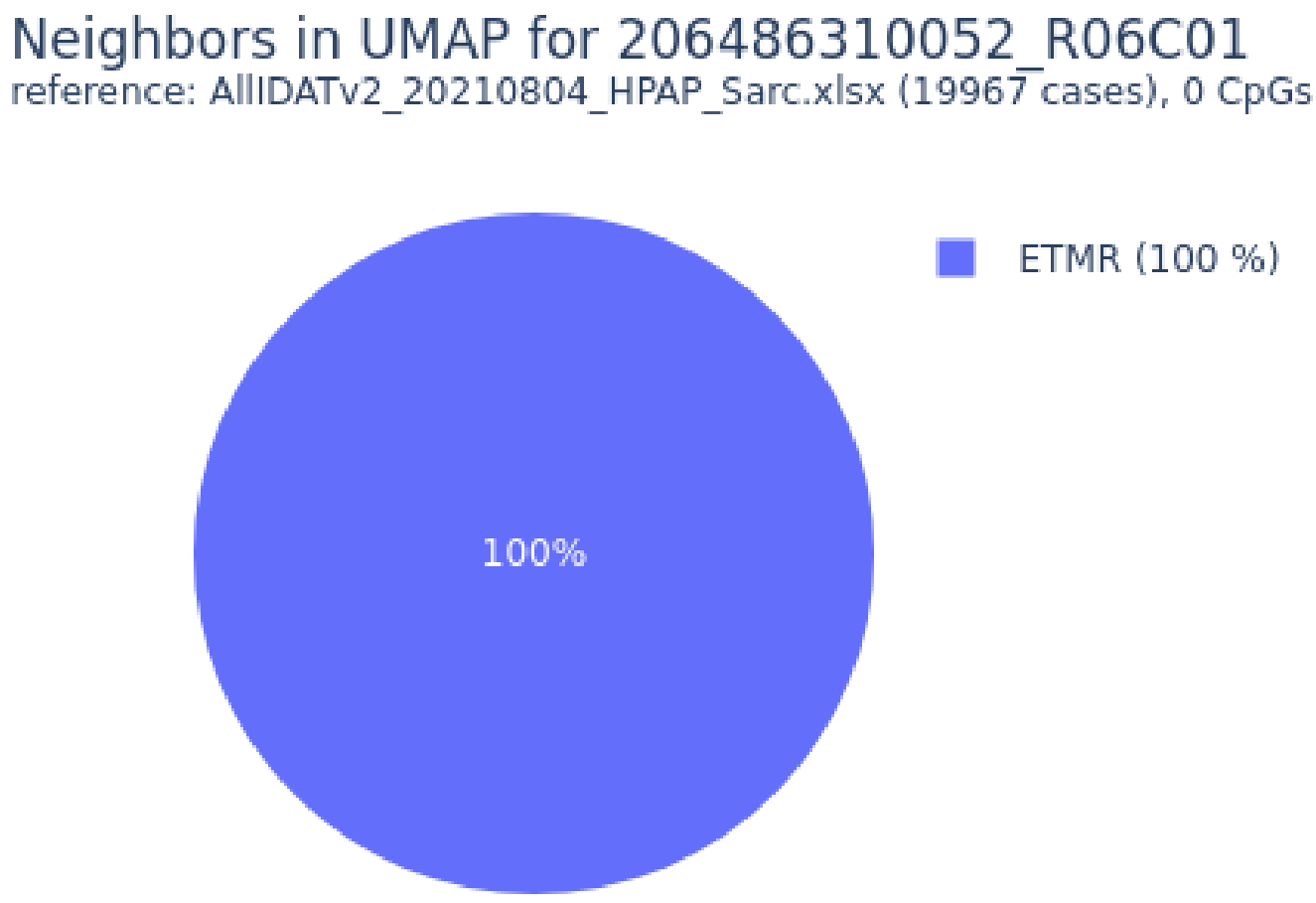

Copy number plot

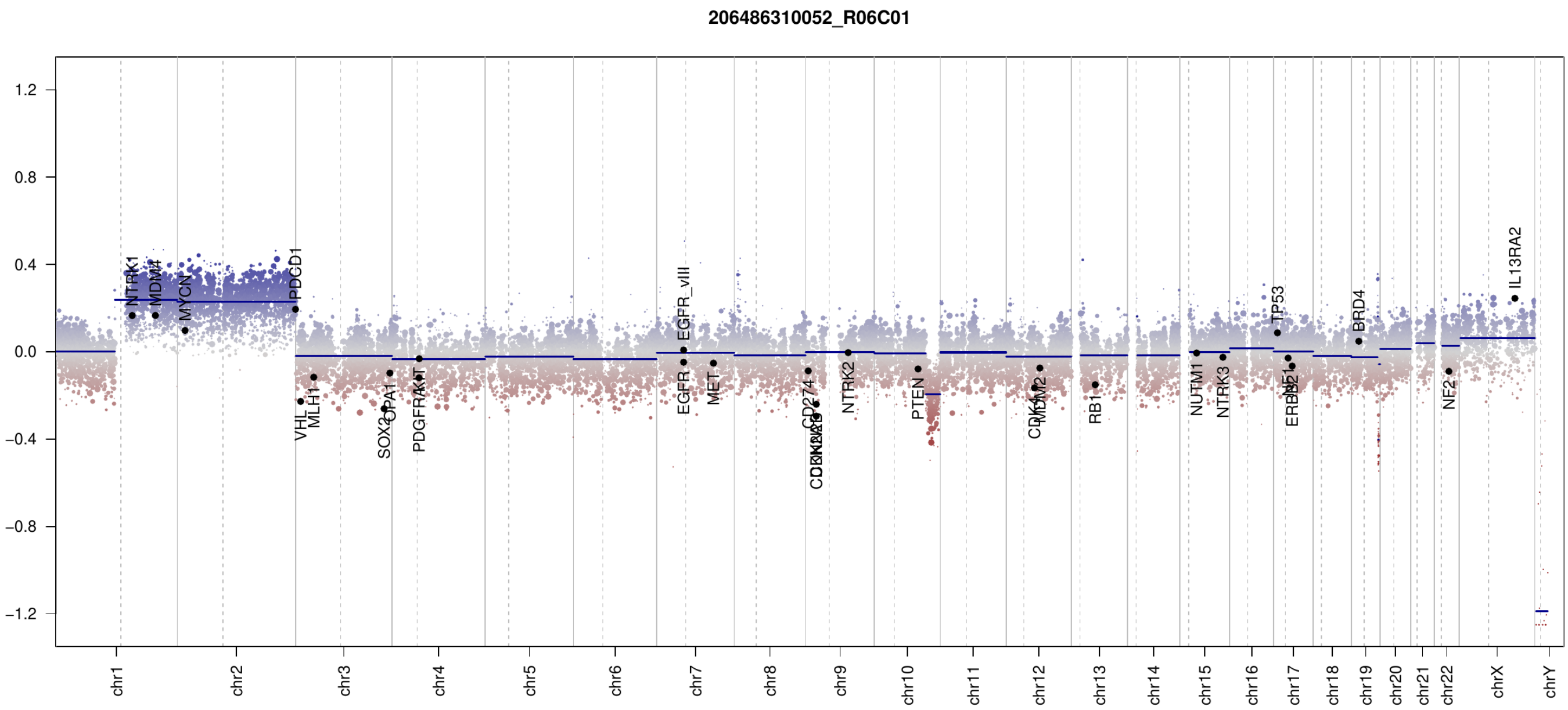

EpiDiP Report for Sample 206486310053_R07C01

Generated on	:	epidip02 / 20260616_172228
Detected barcode	:	undetermined
Valid reads	:	0
450K overlap CpG count	:	0
Reference data	:	AllIDATv2_20210804_HPAP_Sarc

Methylation UMAP plot

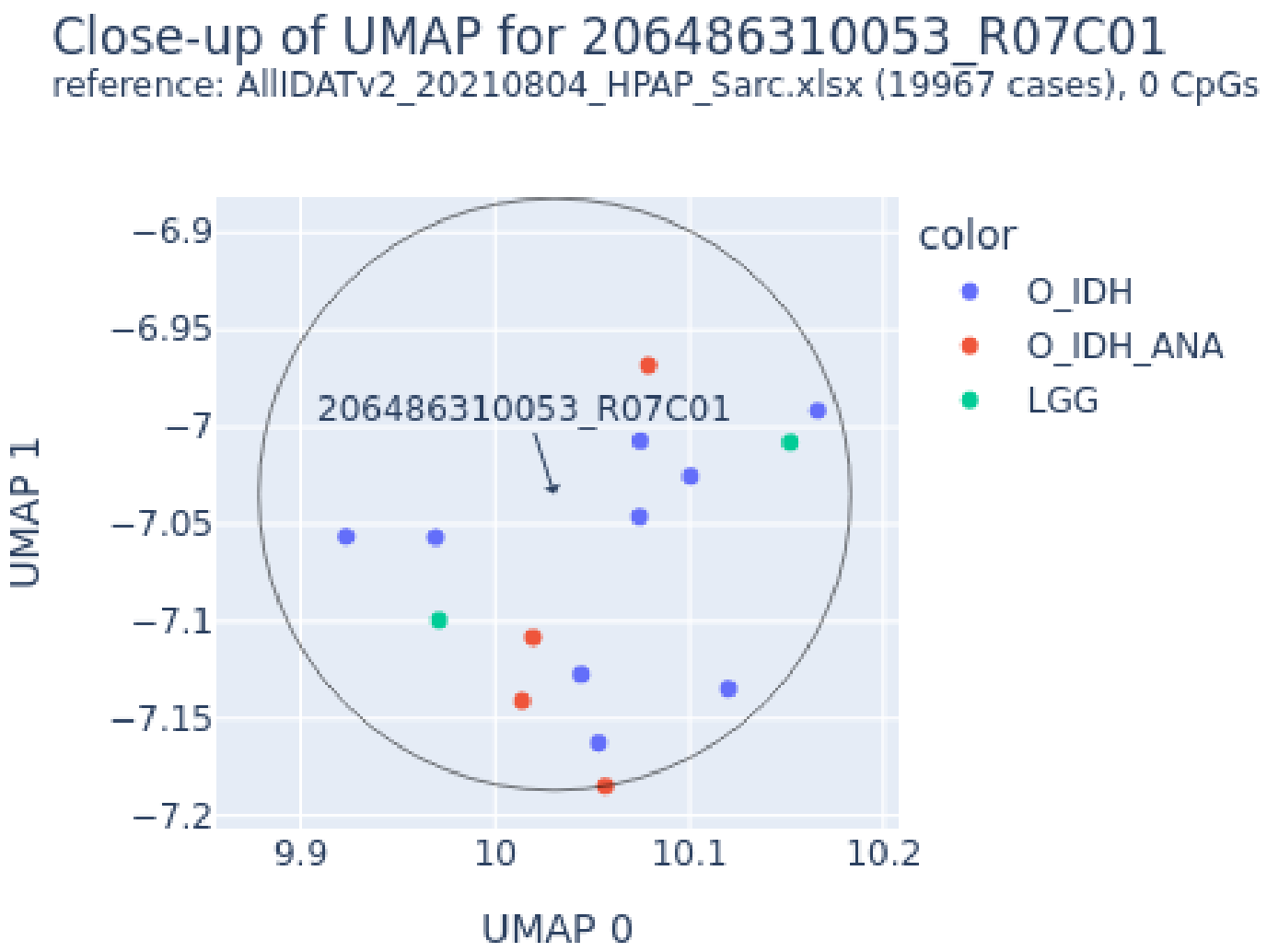

Methylation UMAP Score

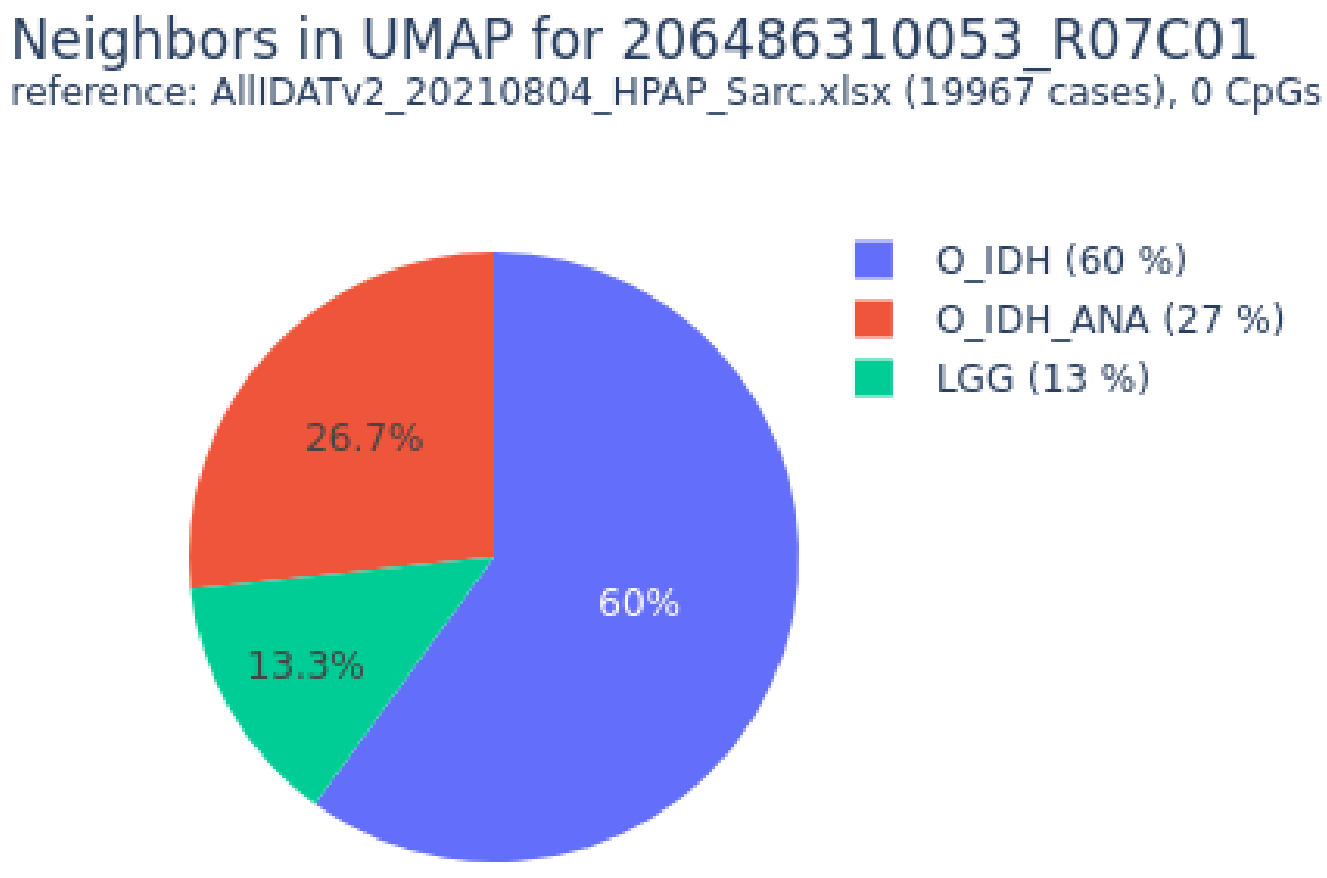

Copy number plot

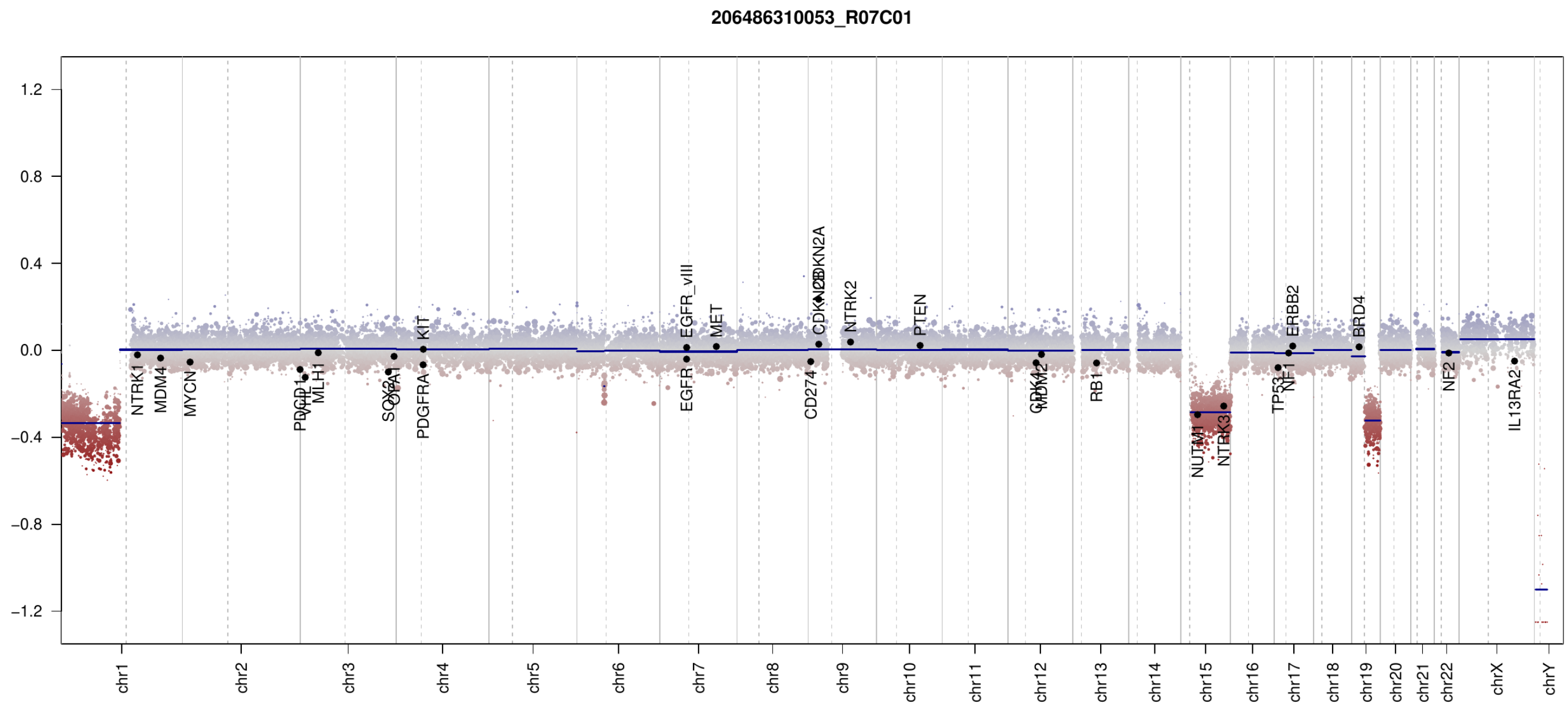

EpiDiP Report for Sample 206503150053_R02C01

Generated on	:	epidip02 / 20260616_172632
Detected barcode	:	undetermined
Valid reads	:	0
450K overlap CpG count	:	0
Reference data	:	AllIDATv2_20210804_HPAP_Sarc

Methylation UMAP plot

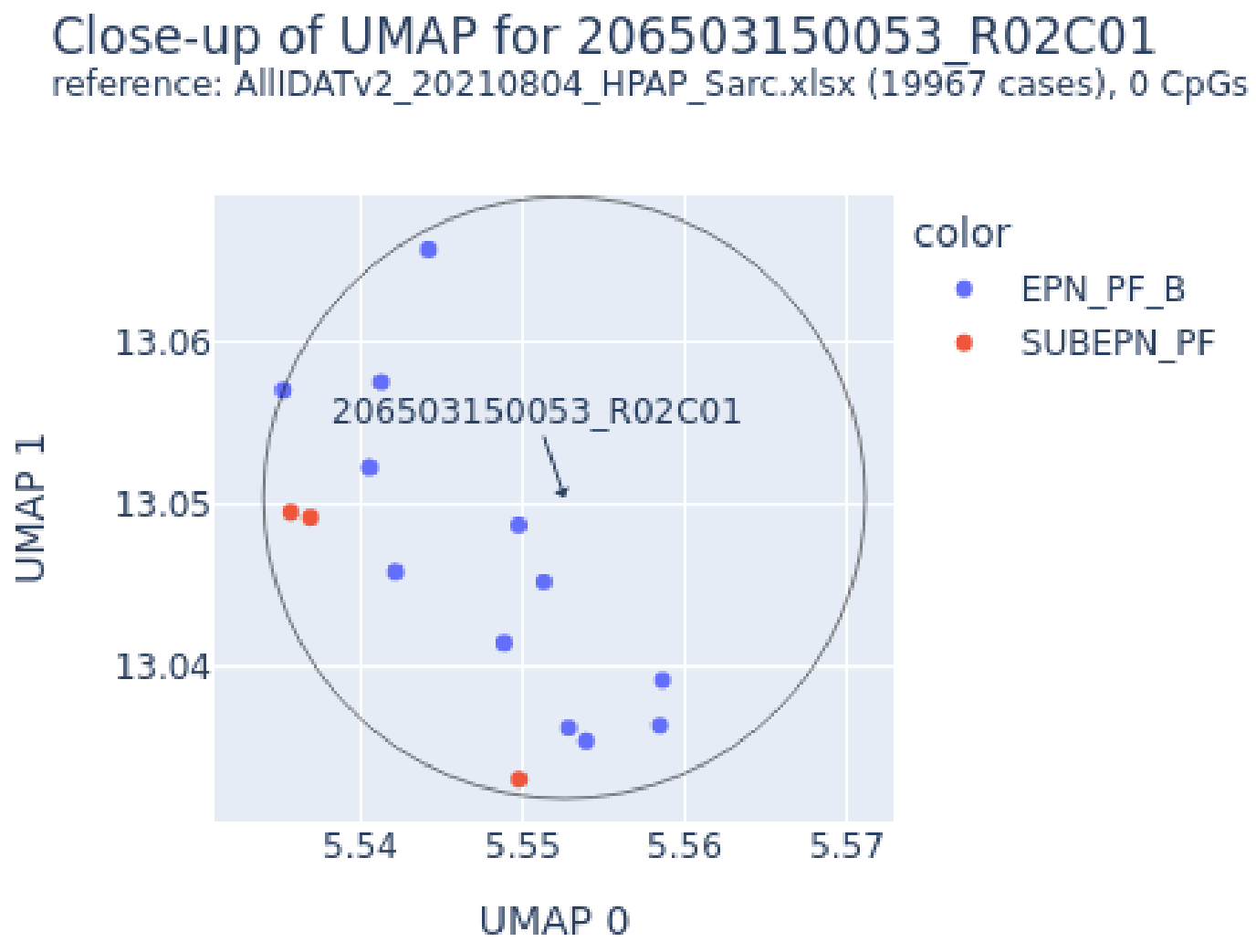

Methylation UMAP Score

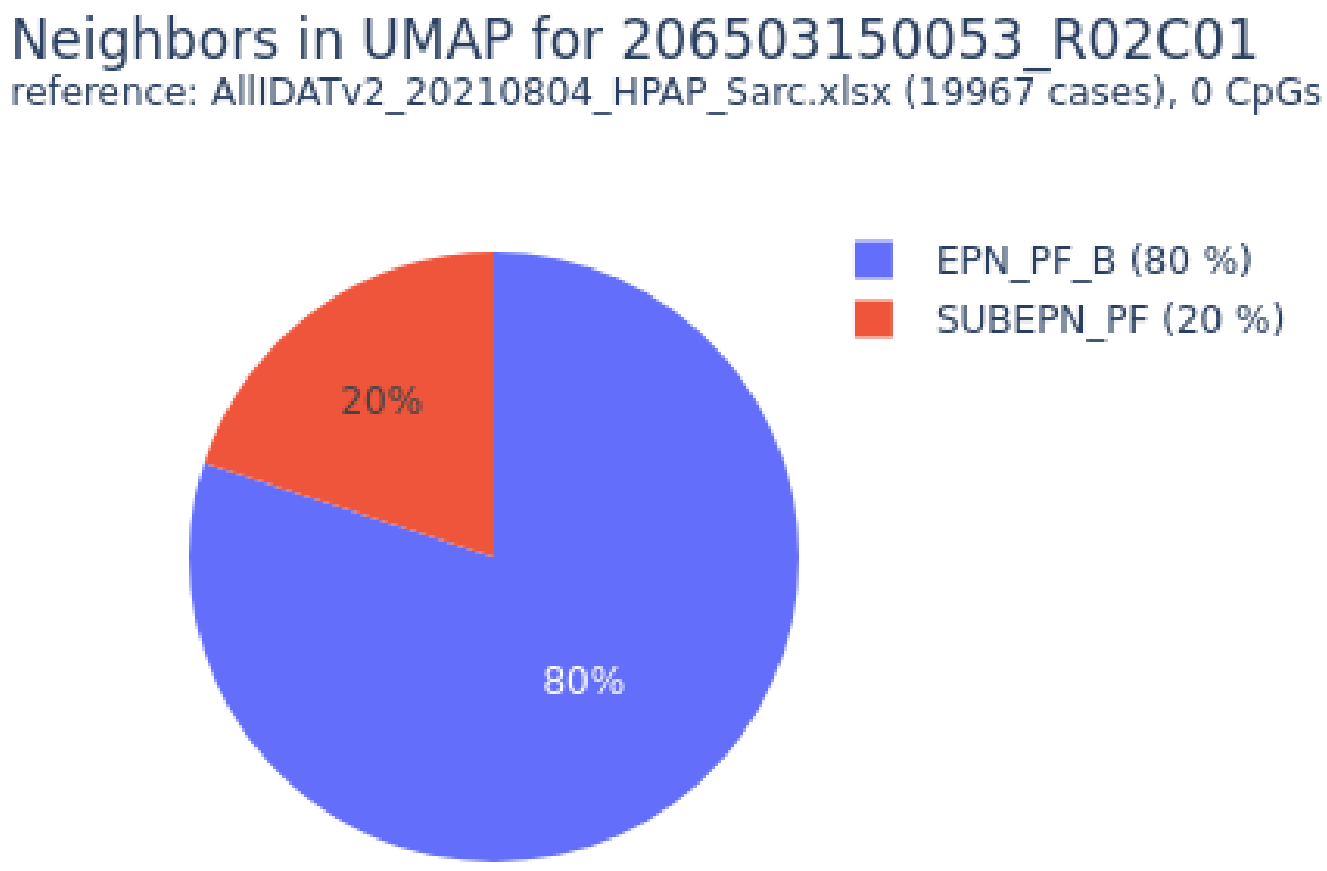

Copy number plot

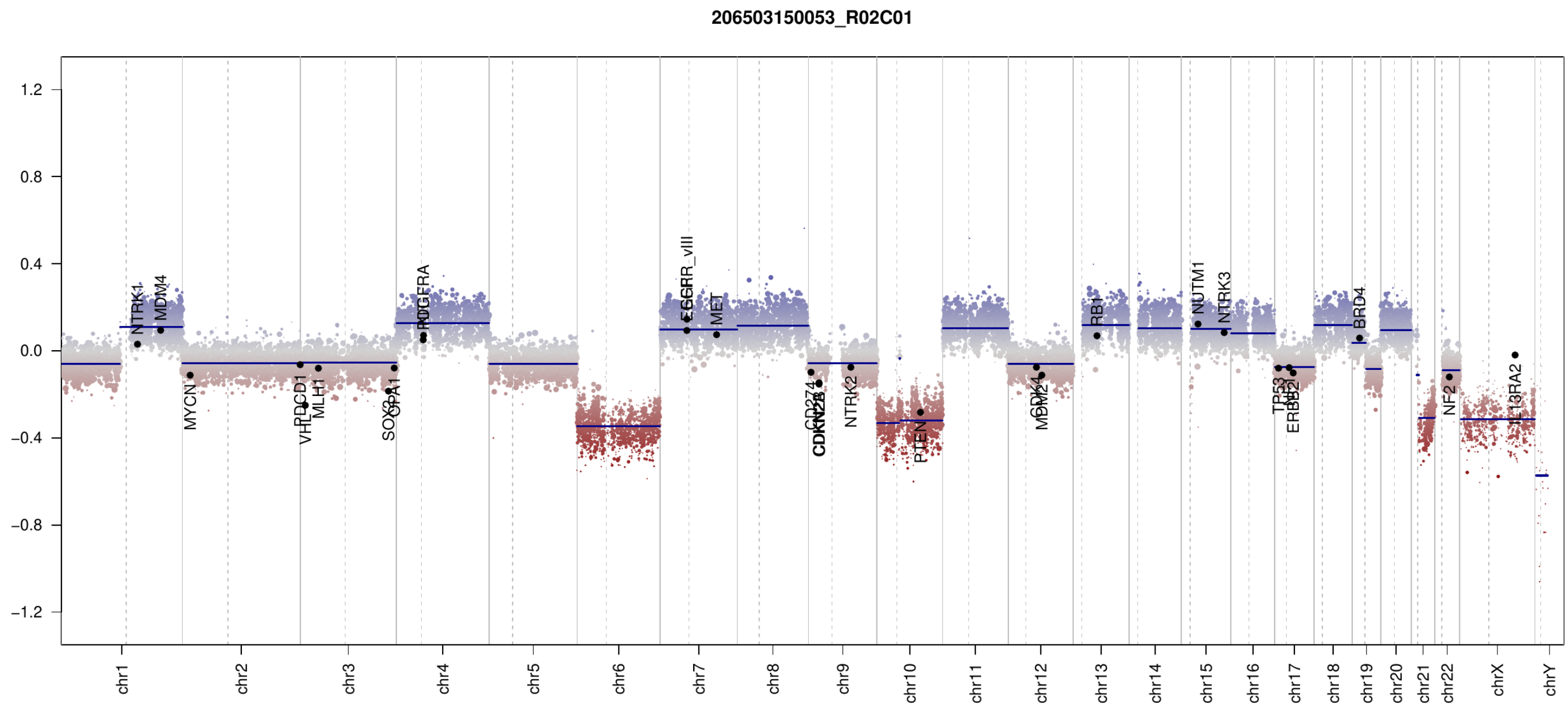

EpiDiP Report for Sample 207015200146_R01C01

Generated on	:	epidip02 / 20260616_171731
Detected barcode	:	undetermined
Valid reads	:	0
450K overlap CpG count	:	0
Reference data	:	AllIDATv2_20210804_HPAP_Sarc

Methylation UMAP plot

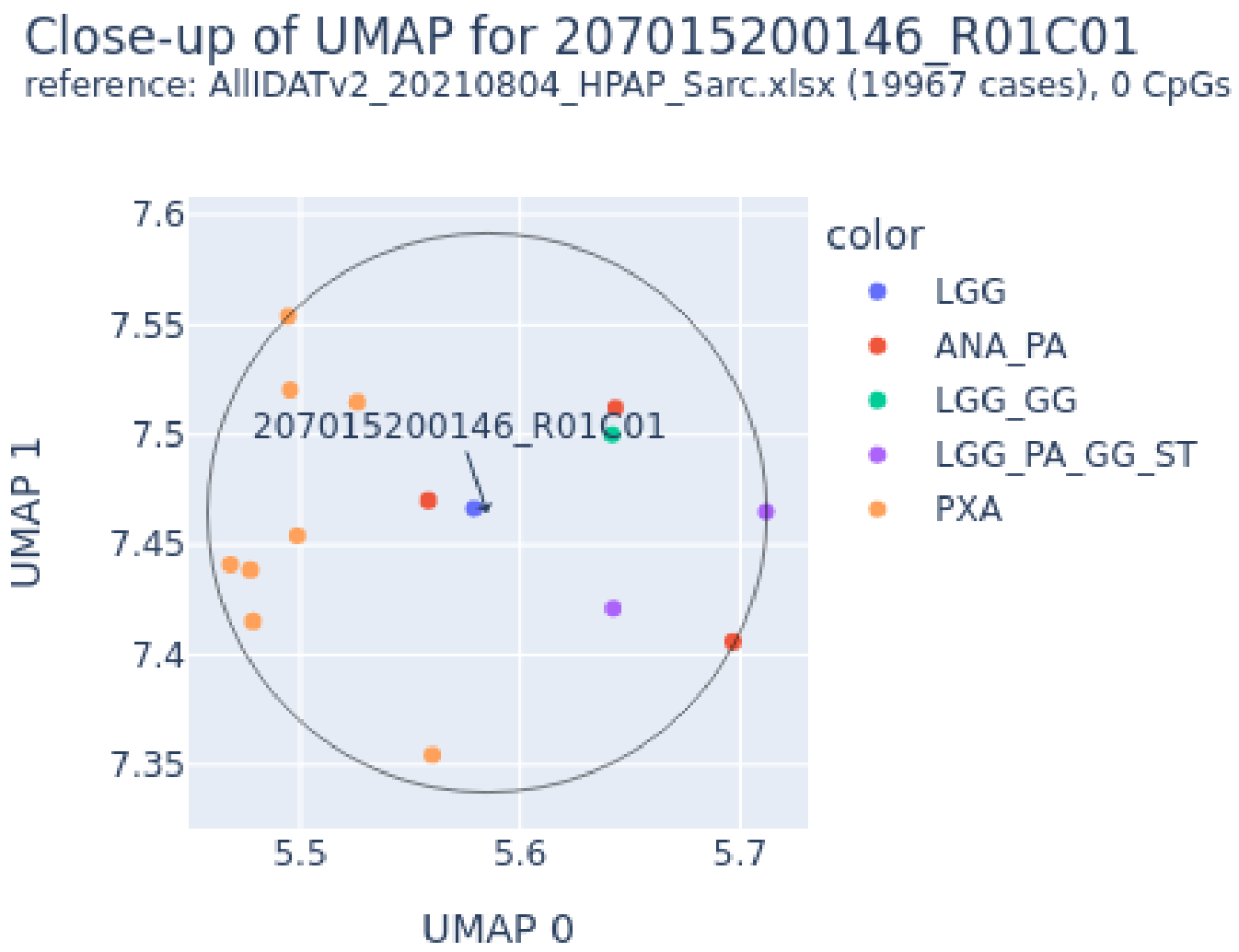

Methylation UMAP Score

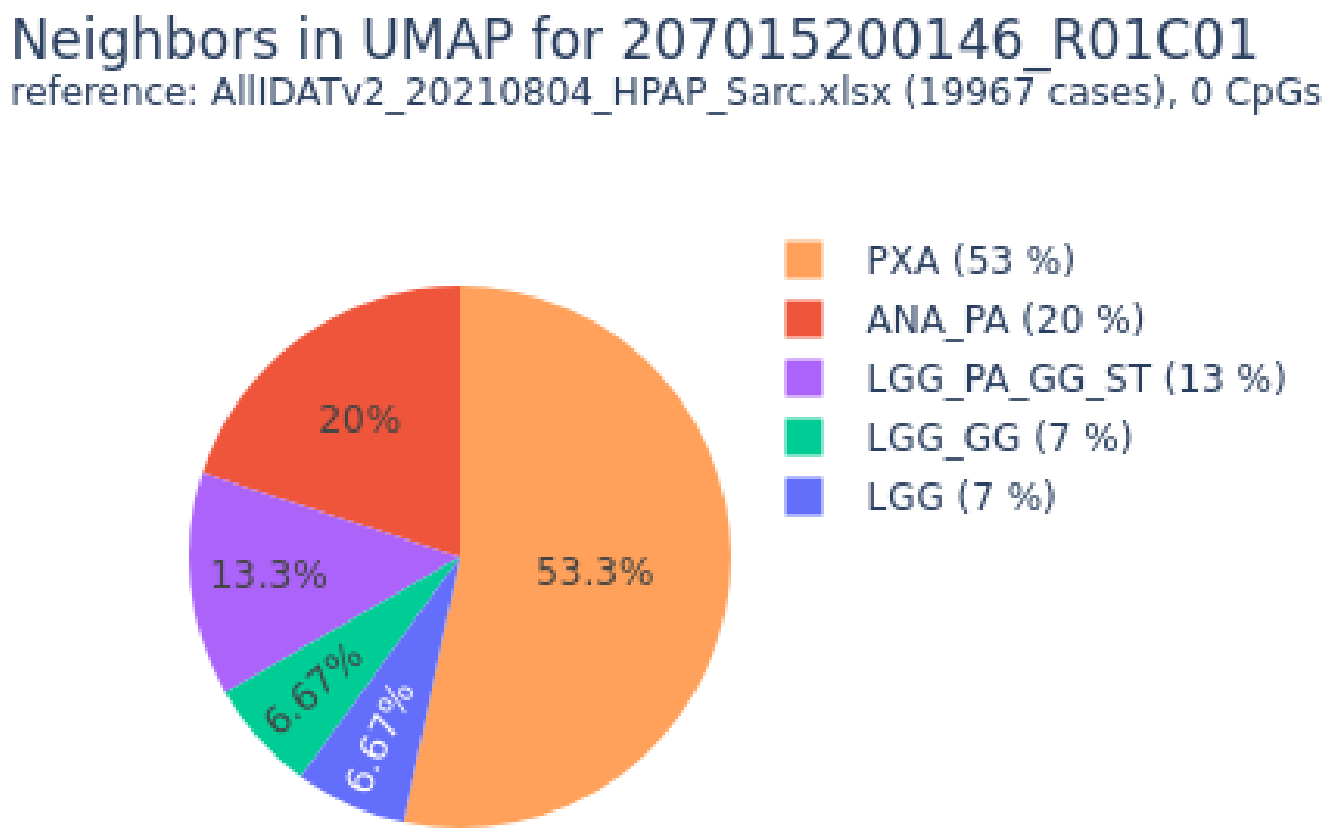

Copy number plot

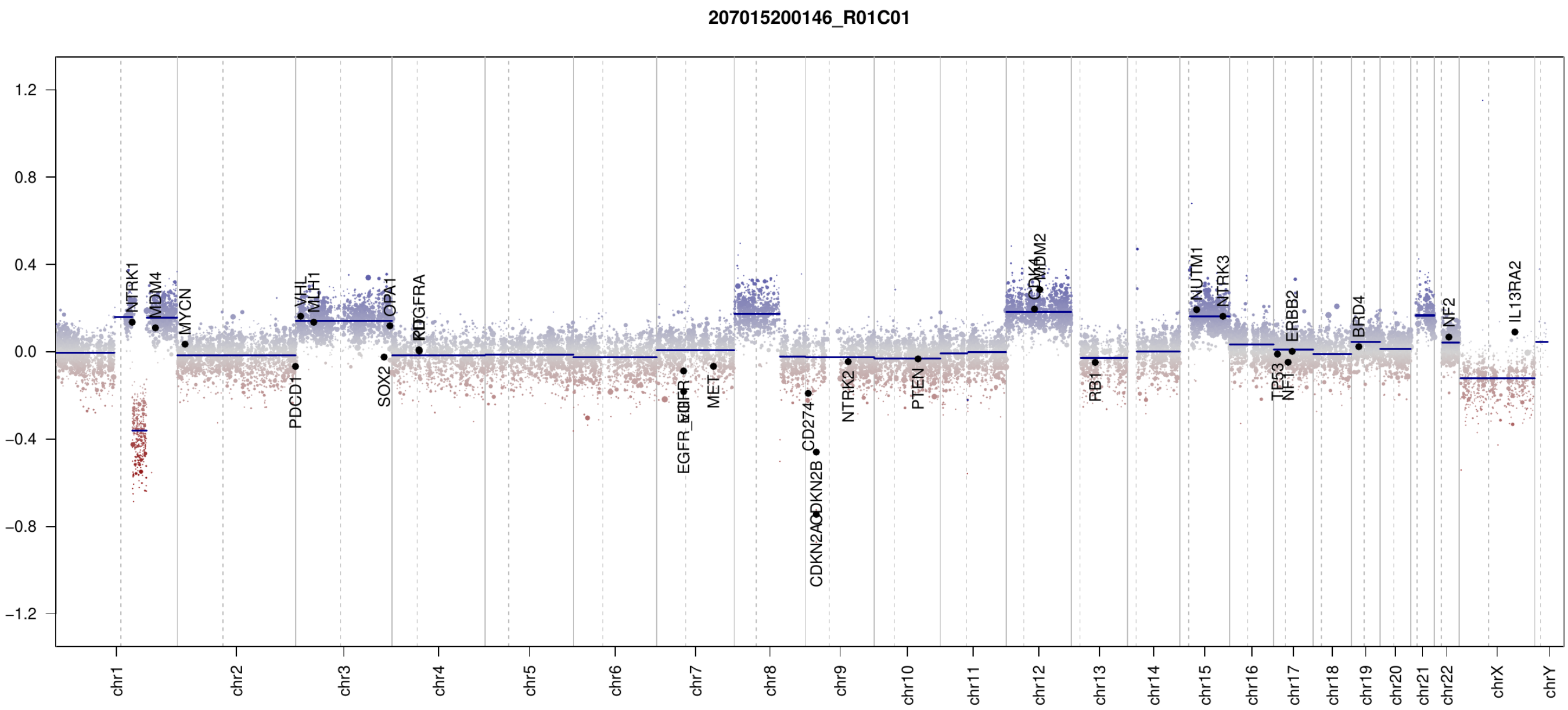

EpiDiP Report for Sample 207335100018_R04C01

Generated on	:	epidip02 / 20260616_172338
Detected barcode	:	undetermined
Valid reads	:	0
450K overlap CpG count	:	0
Reference data	:	AllIDATv2_20210804_HPAP_Sarc

Methylation UMAP plot

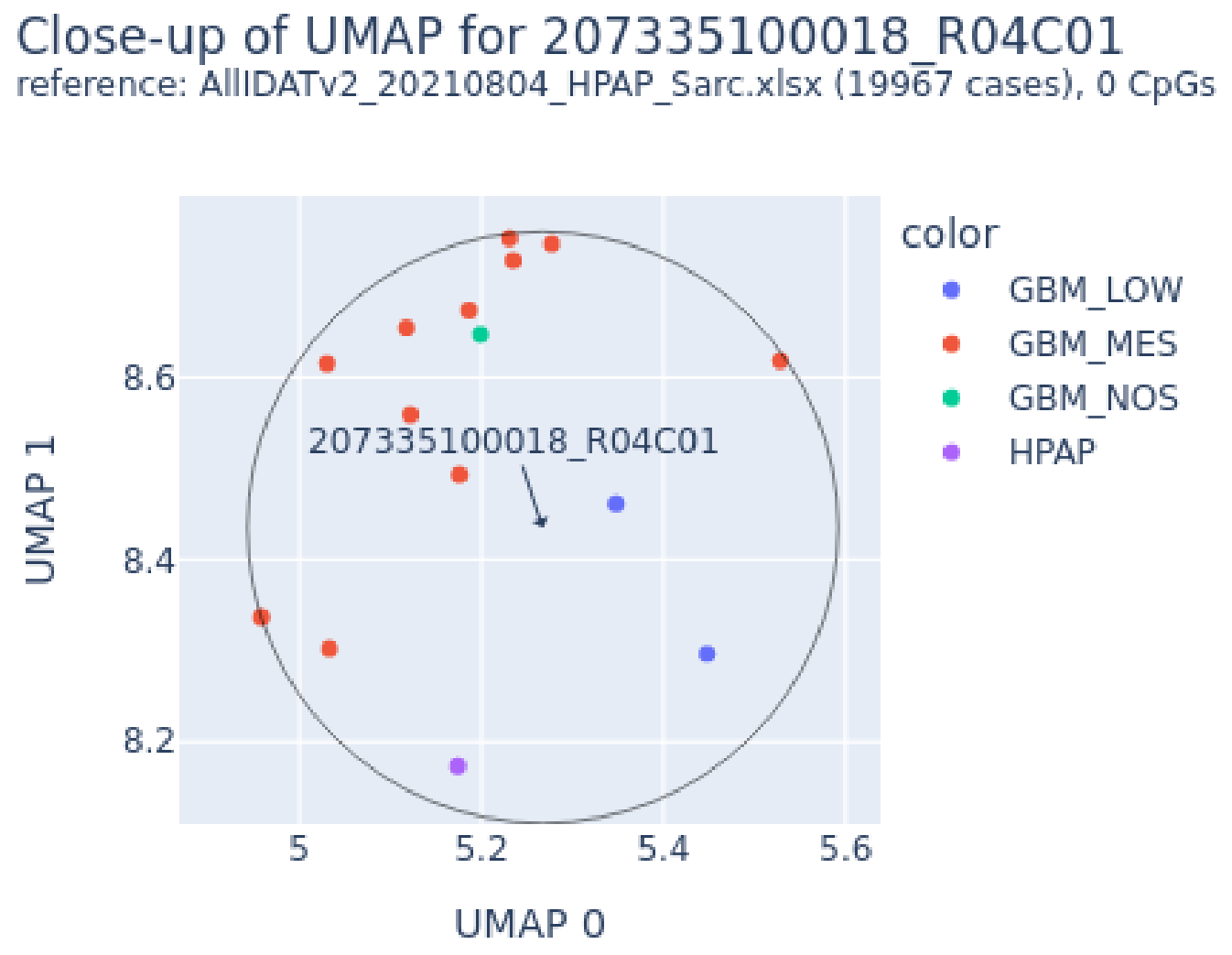

Methylation UMAP Score

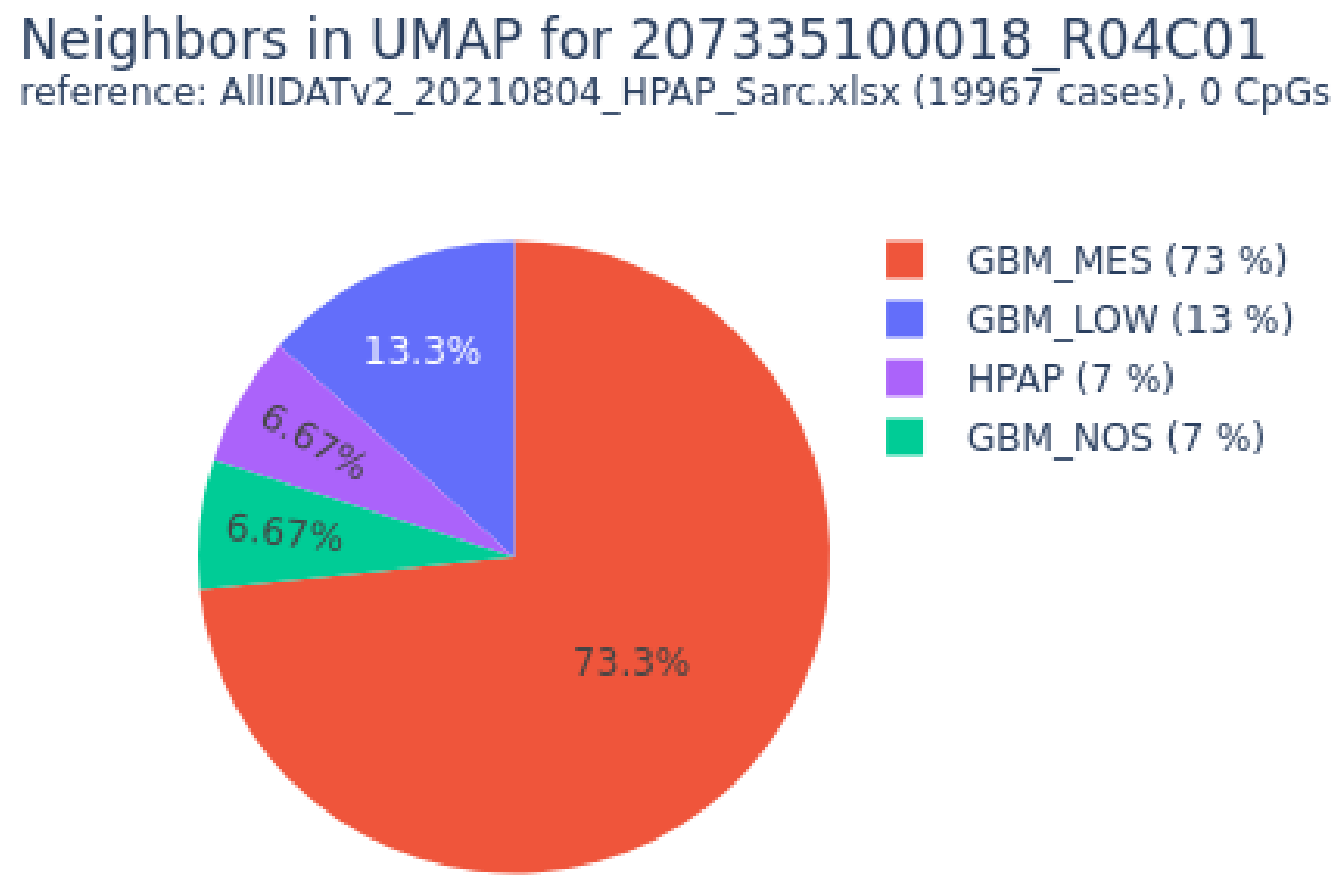

Copy number plot

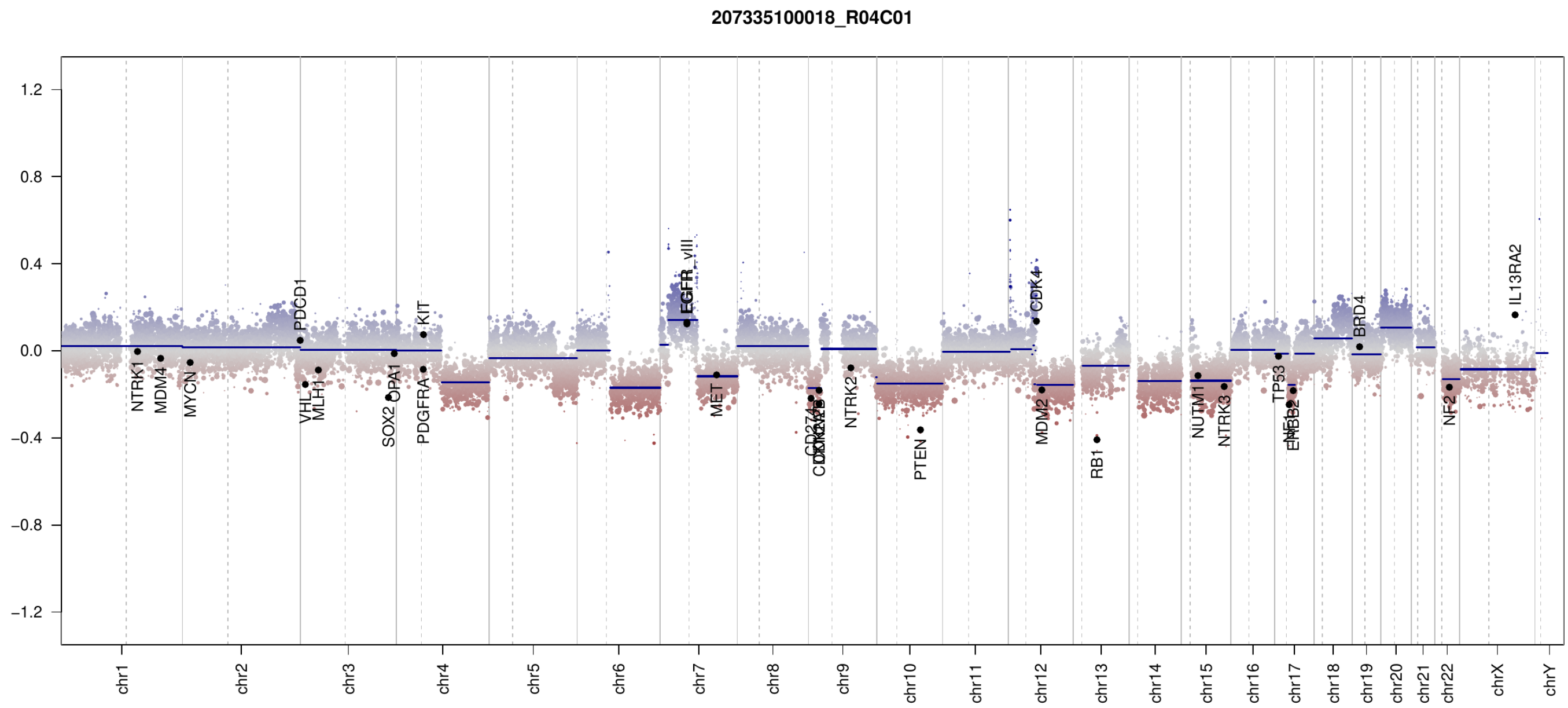

EpiDiP Report for Sample 207335100077_R06C01

Generated on	:	epidip02 / 20260616_173424
Detected barcode	:	undetermined
Valid reads	:	0
450K overlap CpG count	:	0
Reference data	:	AllIDATv2_20210804_HPAP_Sarc

Methylation UMAP plot

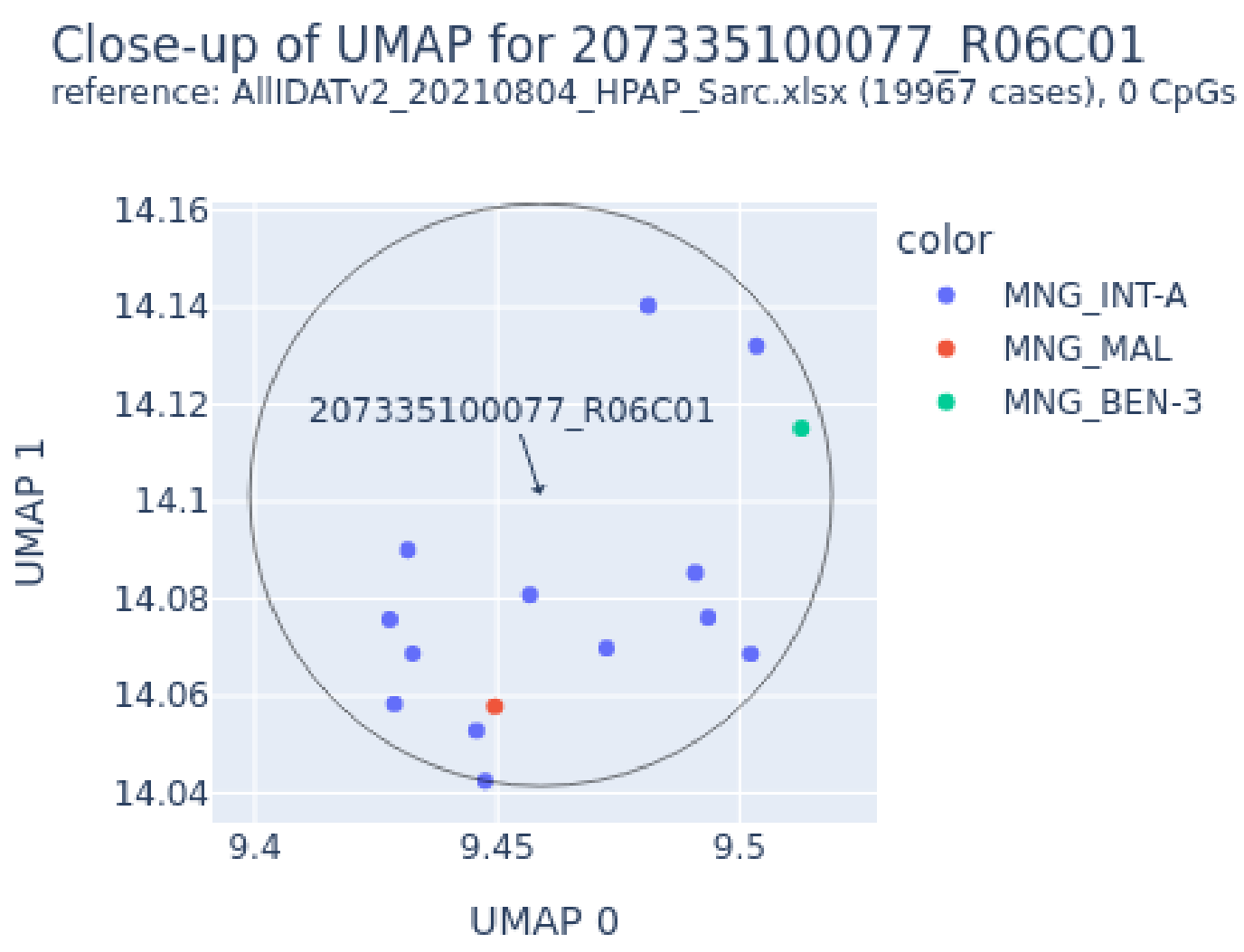

Methylation UMAP Score

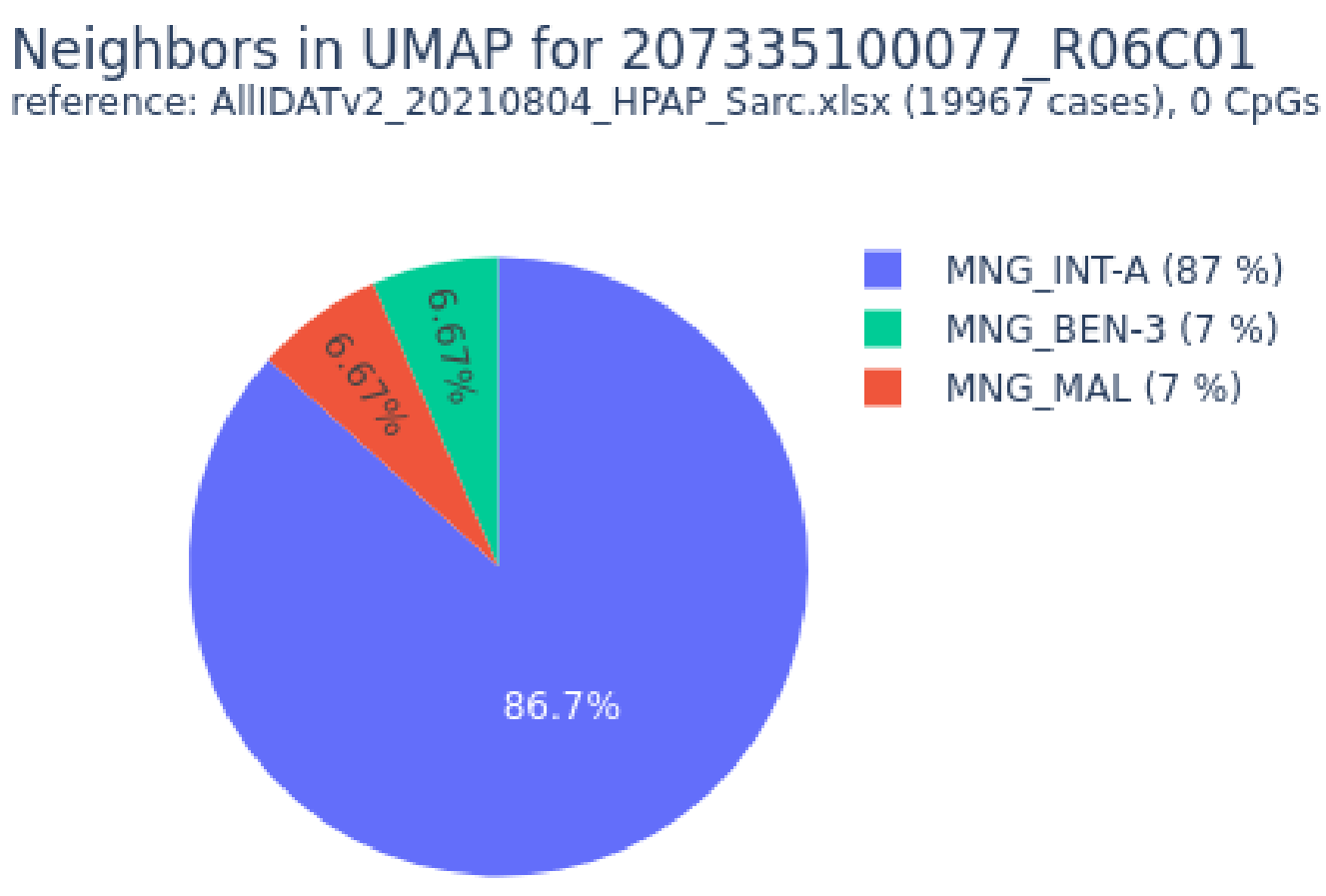

Copy number plot

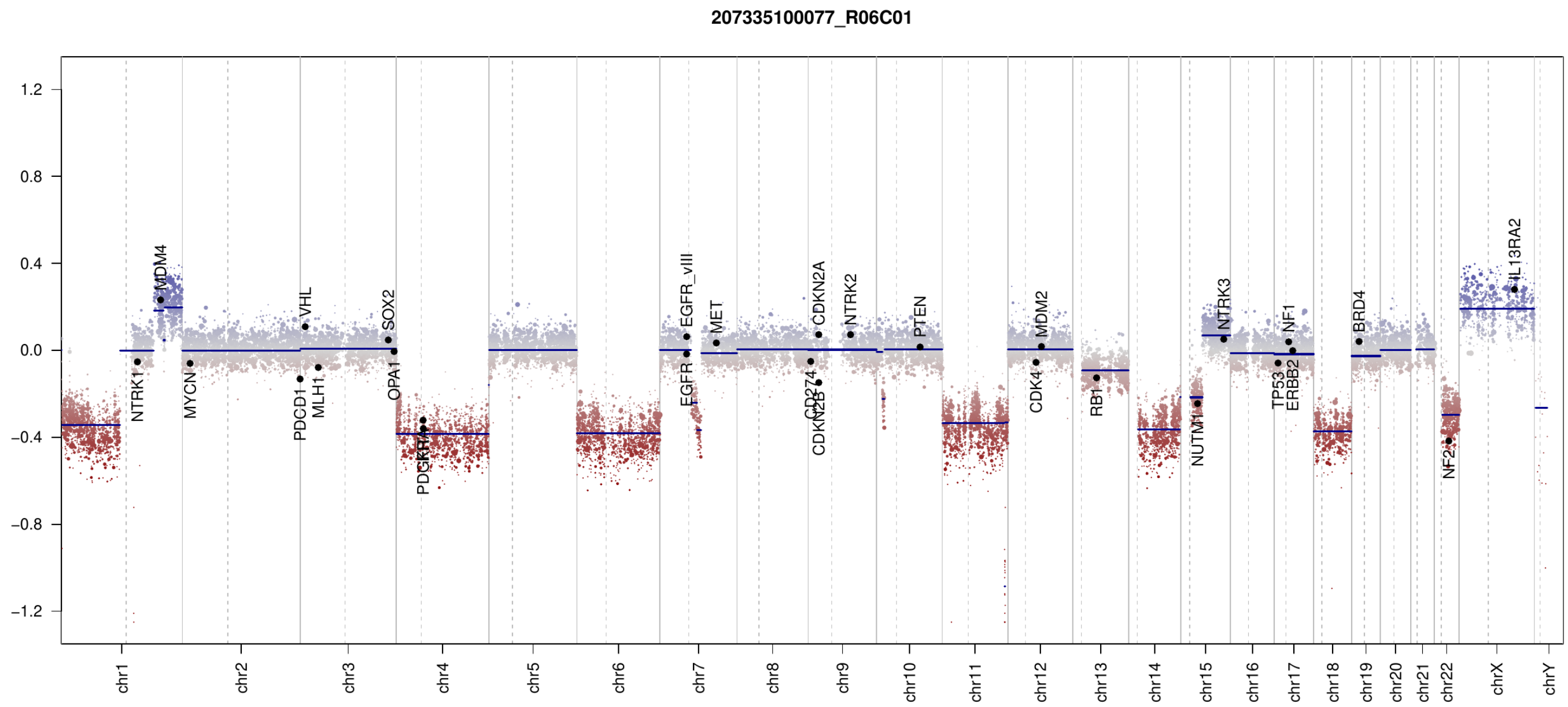

EpiDiP Report for Sample 207716530064_R06C01

Generated on	:	epidip02 / 20260616_172802
Detected barcode	:	undetermined
Valid reads	:	0
450K overlap CpG count	:	0
Reference data	:	AllIDATv2_20210804_HPAP_Sarc

Methylation UMAP plot

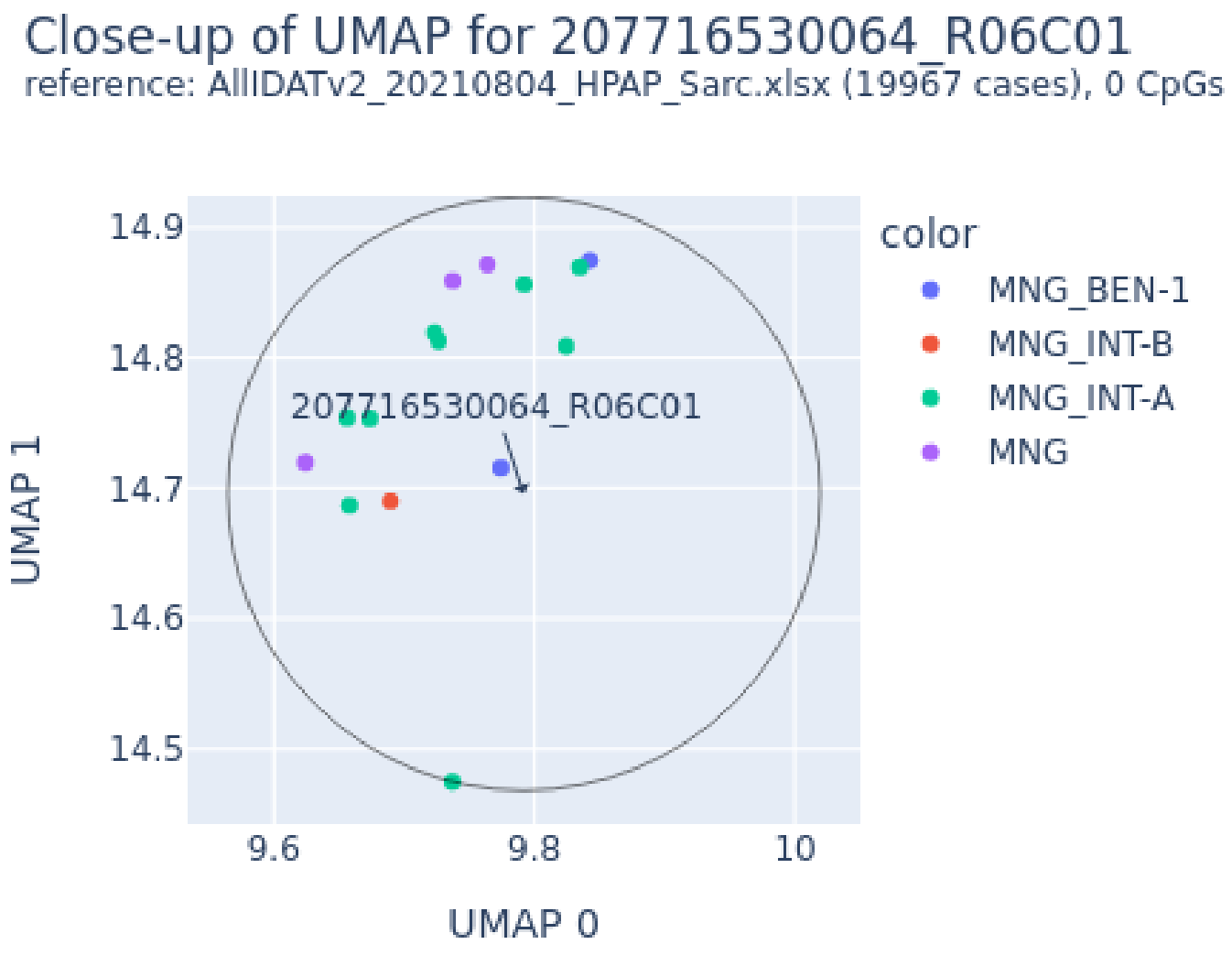

Methylation UMAP Score

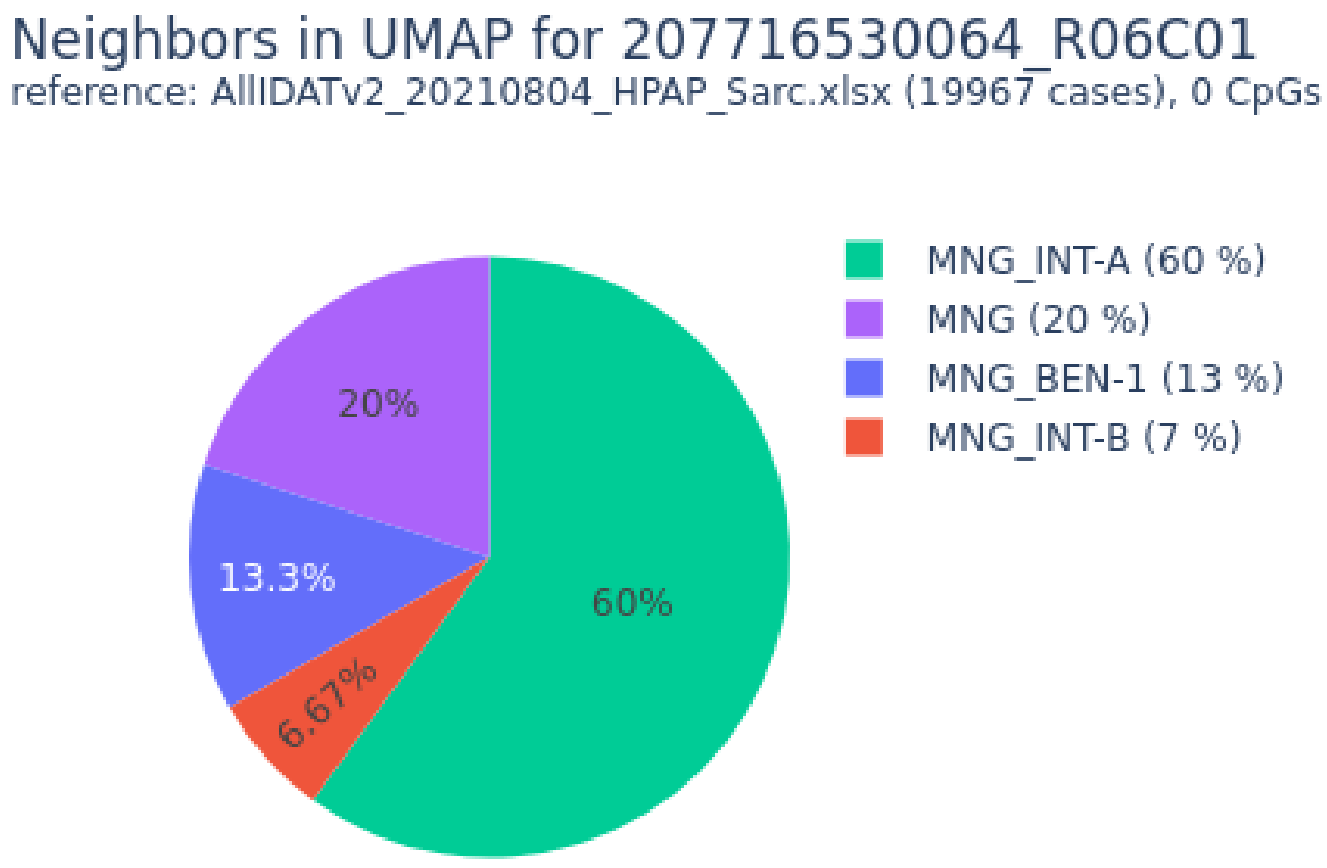

Copy number plot

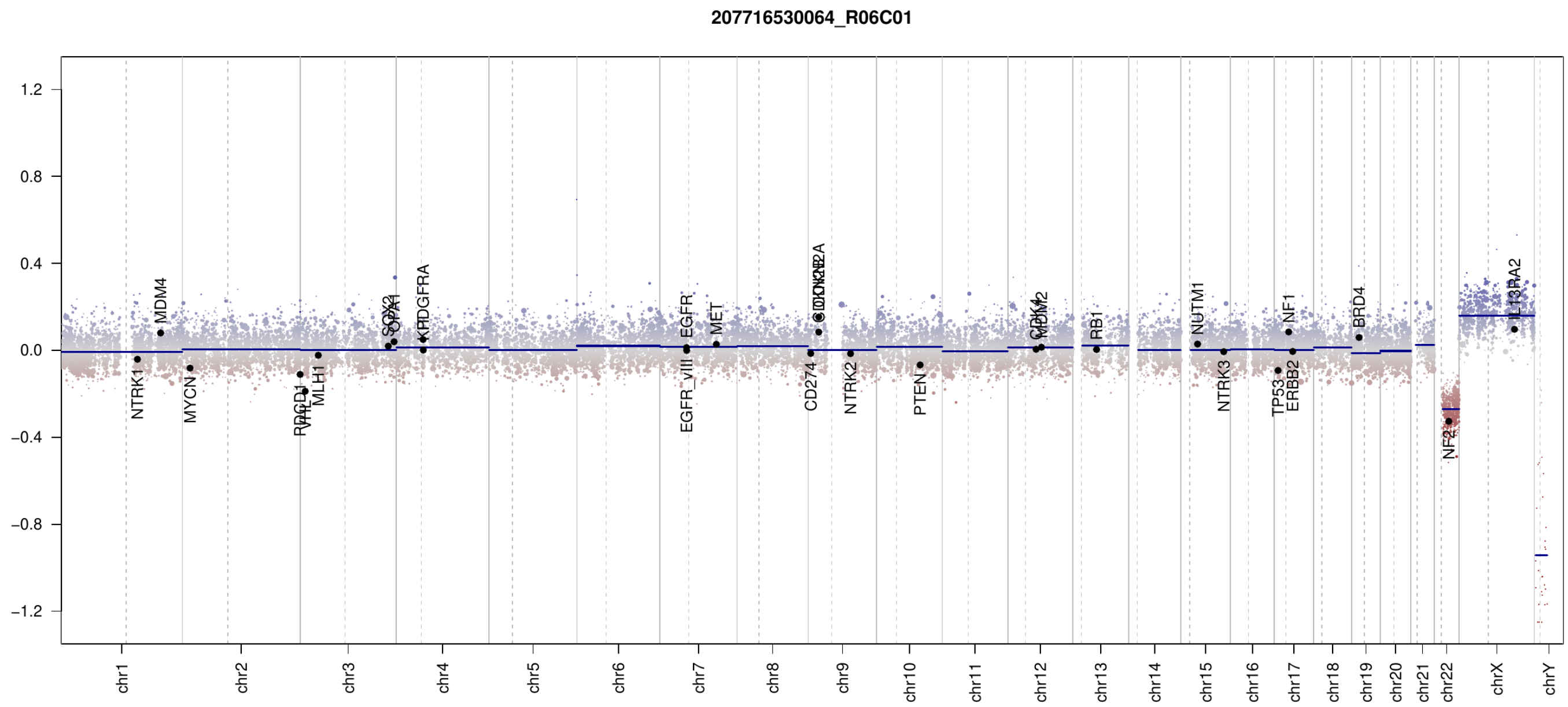

EpiDiP Report for Sample 207716530064_R07C01

Generated on	:	epidip02 / 20260616_172506
Detected barcode	:	undetermined
Valid reads	:	0
450K overlap CpG count	:	0
Reference data	:	AllIDATv2_20210804_HPAP_Sarc

Methylation UMAP plot

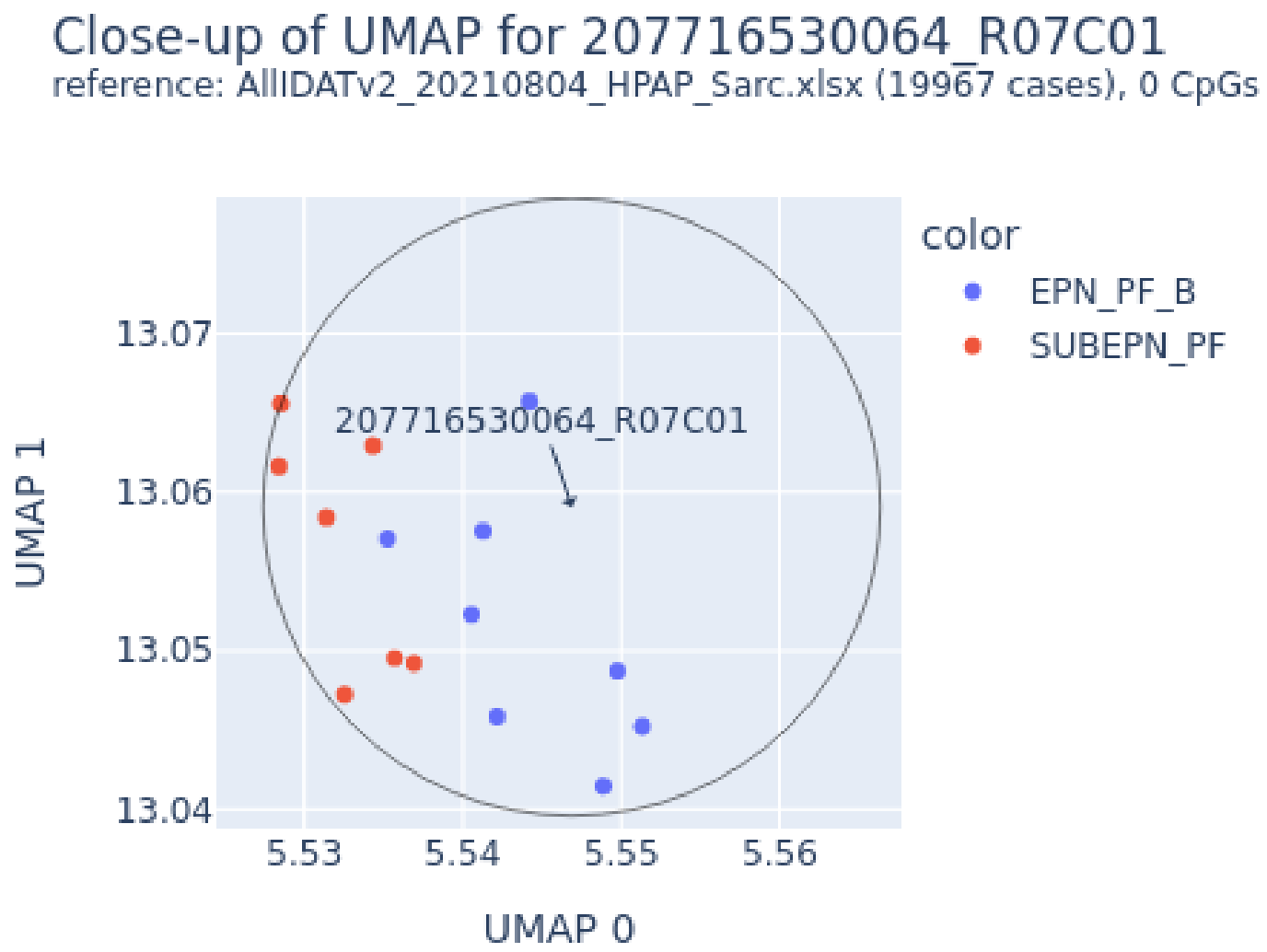

Methylation UMAP Score

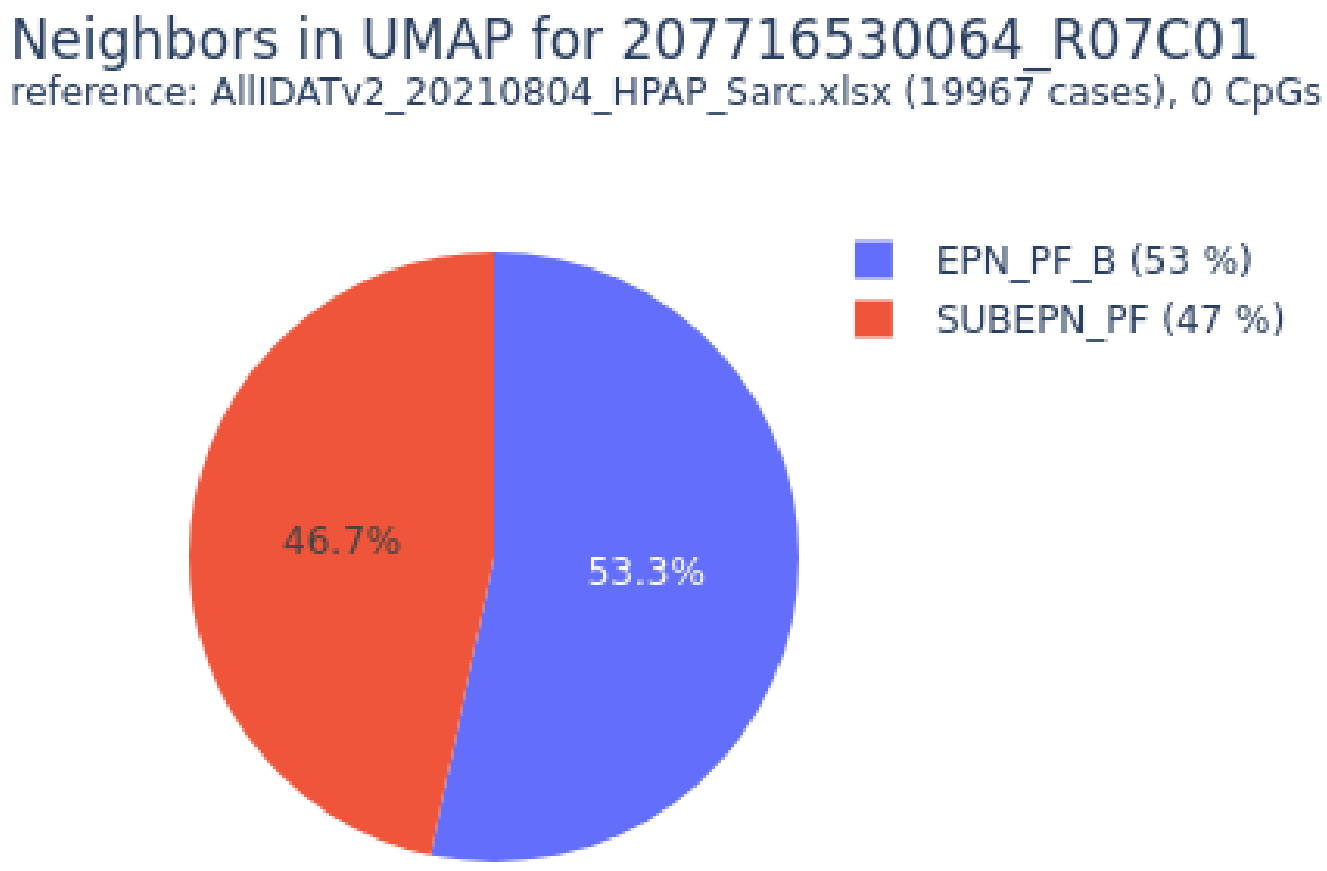

Copy number plot

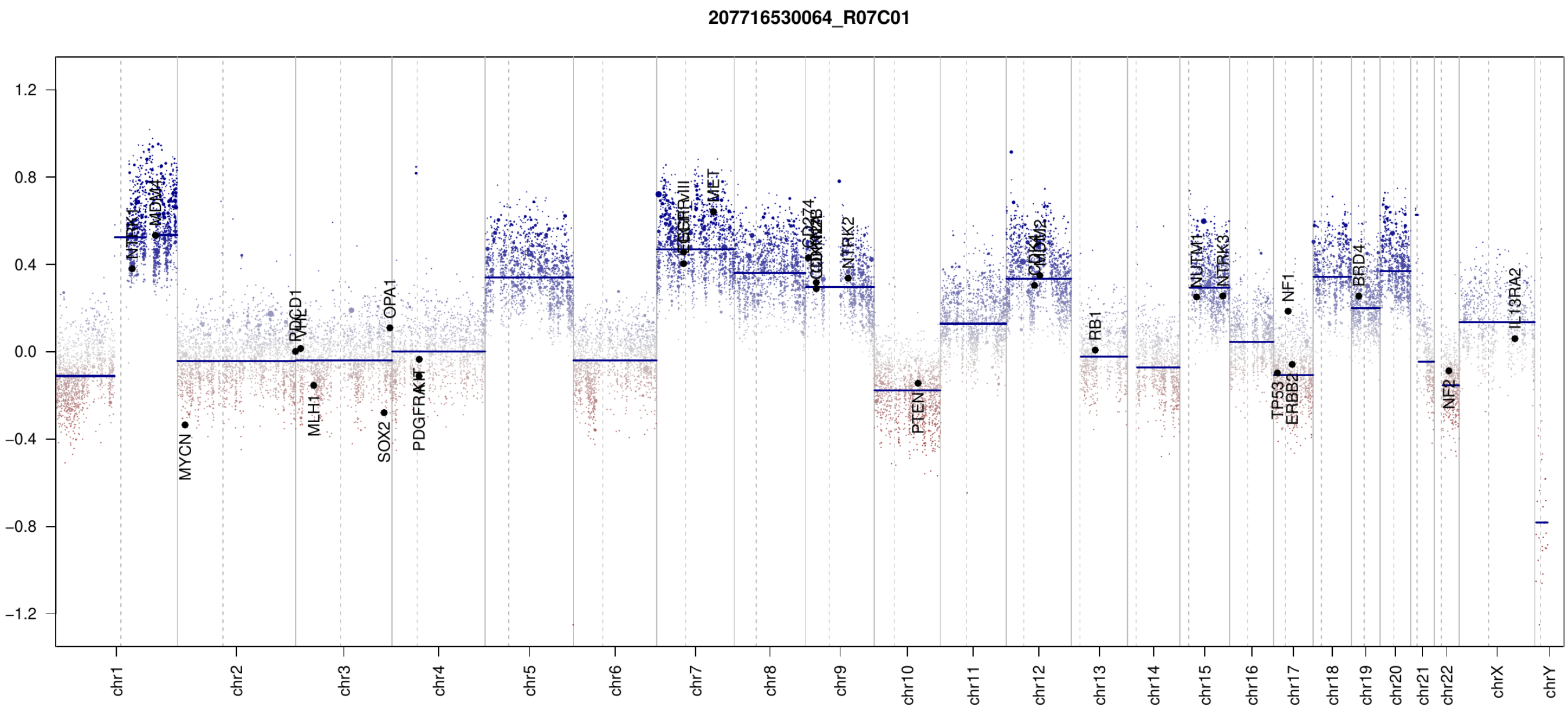

EpiDiP Report for Sample 207716530065_R04C01

Generated on	:	epidip02 / 20260616_171900
Detected barcode	:	undetermined
Valid reads	:	0
450K overlap CpG count	:	0
Reference data	:	AllIDATv2_20210804_HPAP_Sarc

Methylation UMAP plot

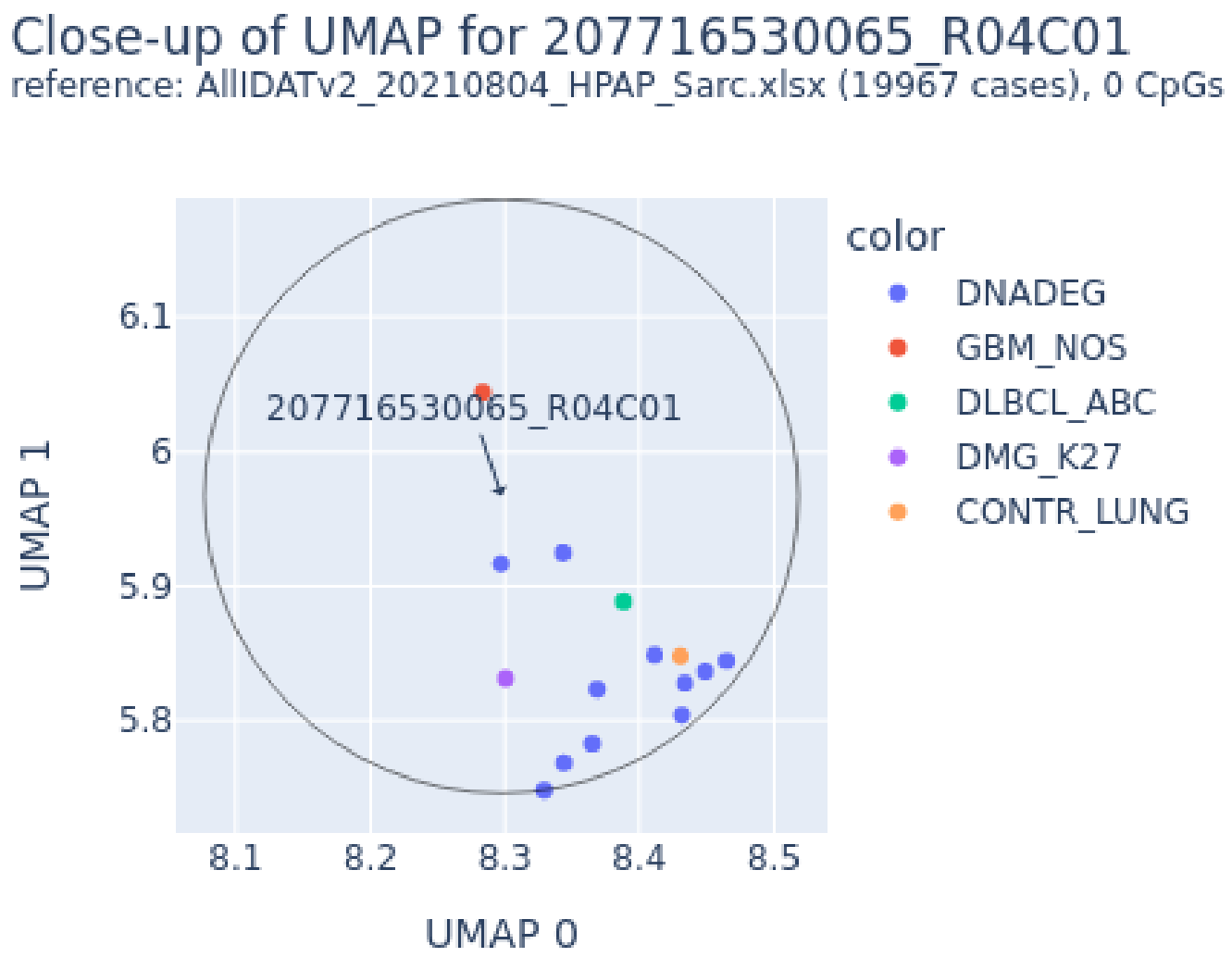

Methylation UMAP Score

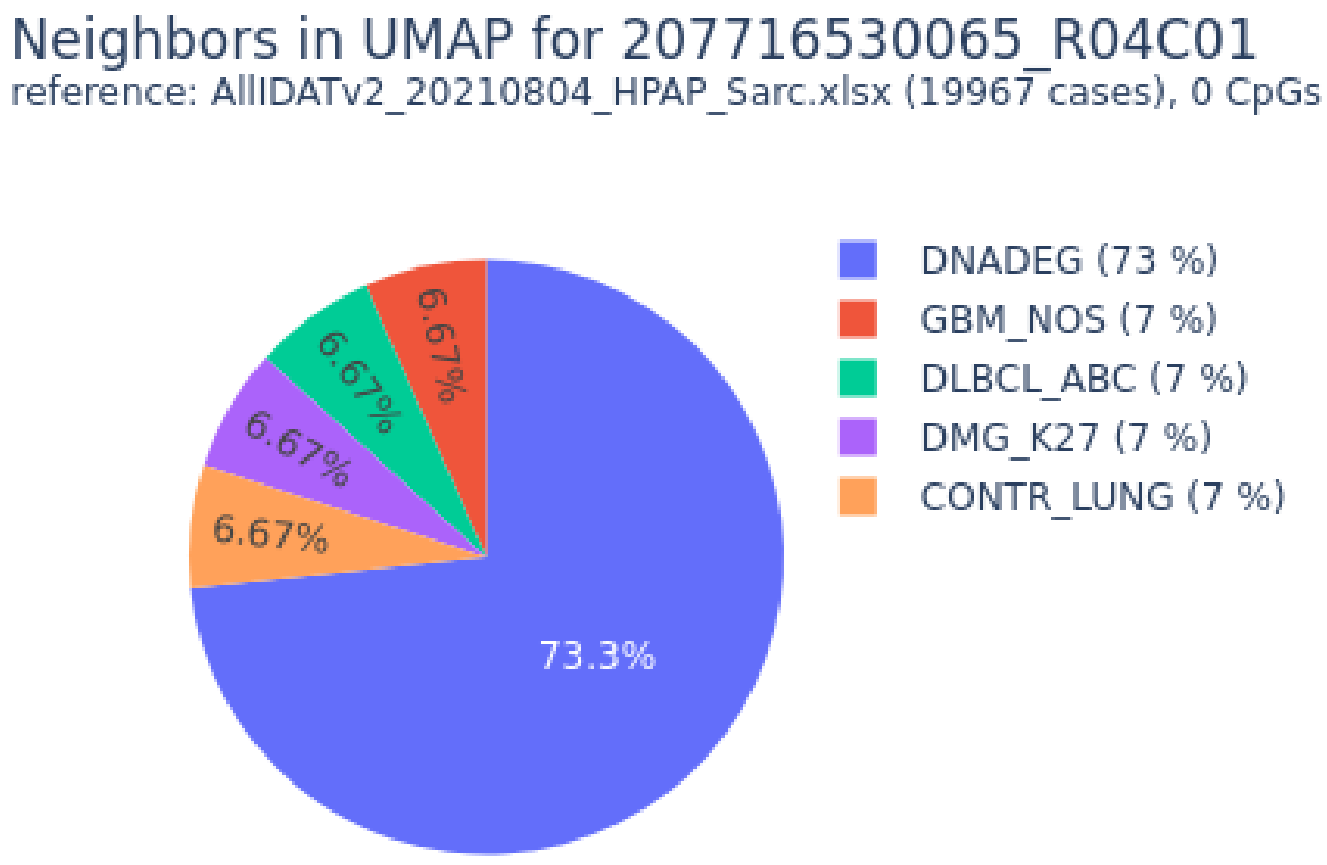

Copy number plot

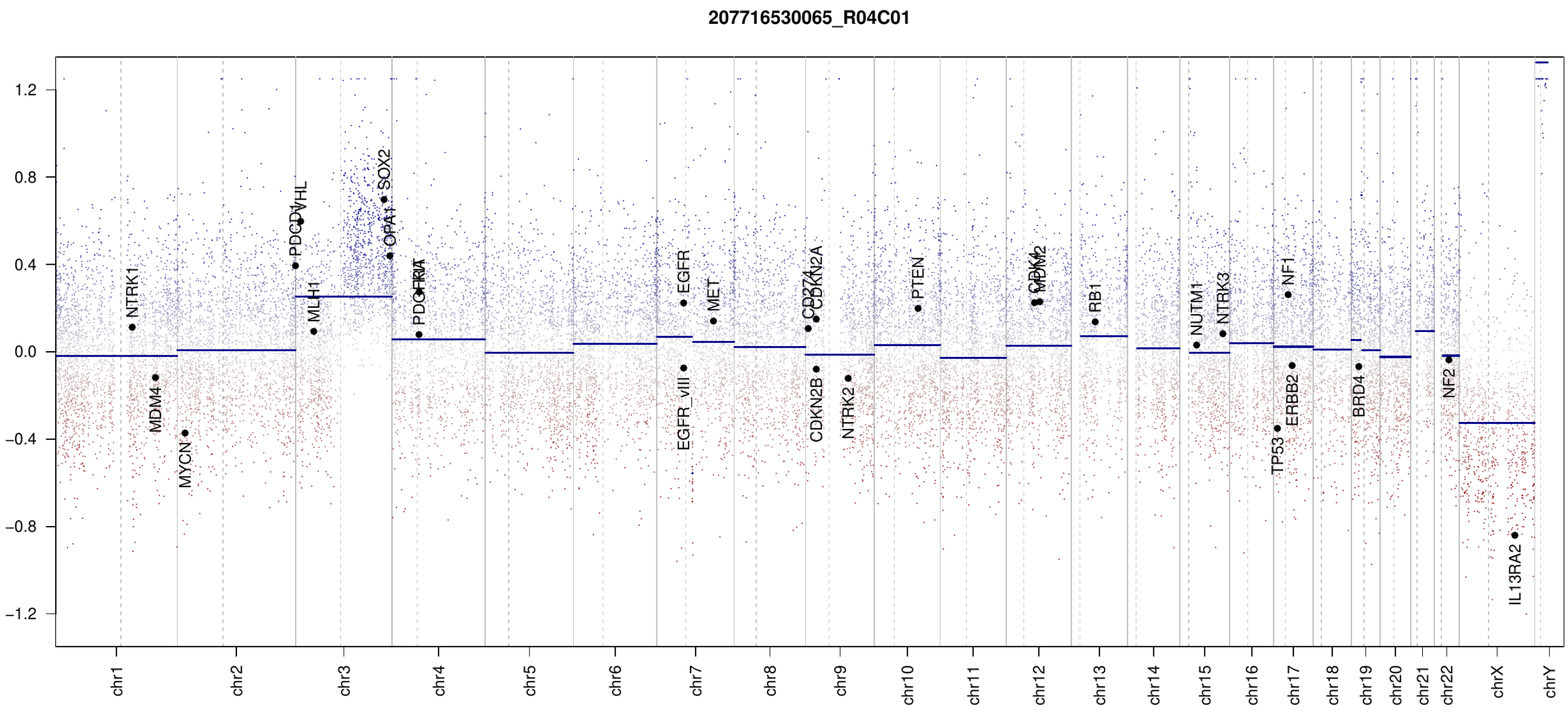

EpiDiP Report for Sample 207755830124_R03C01

Generated on	:	epidip02 / 20260616_171607
Detected barcode	:	undetermined
Valid reads	:	0
450K overlap CpG count	:	0
Reference data	:	AllIDATv2_20210804_HPAP_Sarc

Methylation UMAP plot

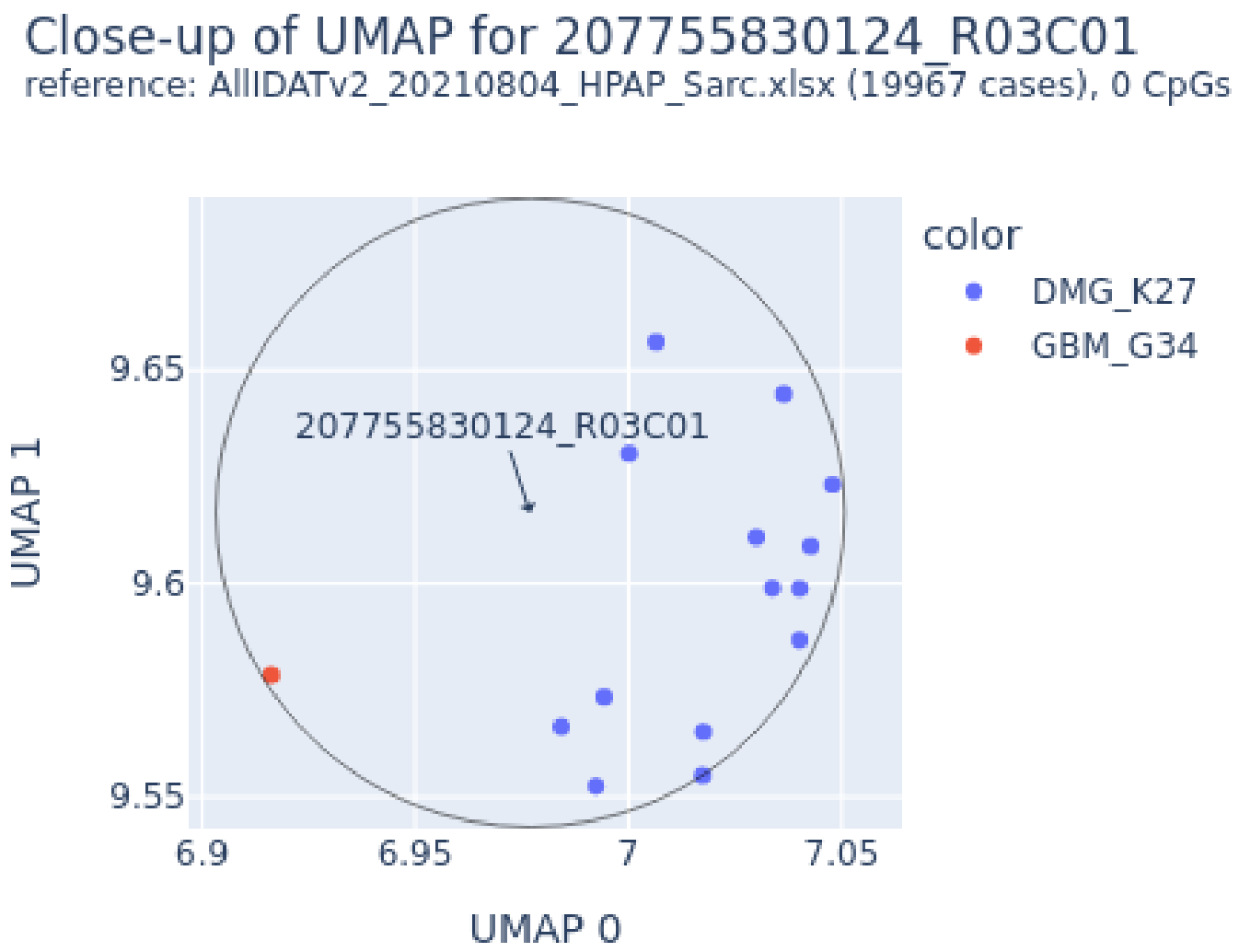

Methylation UMAP Score

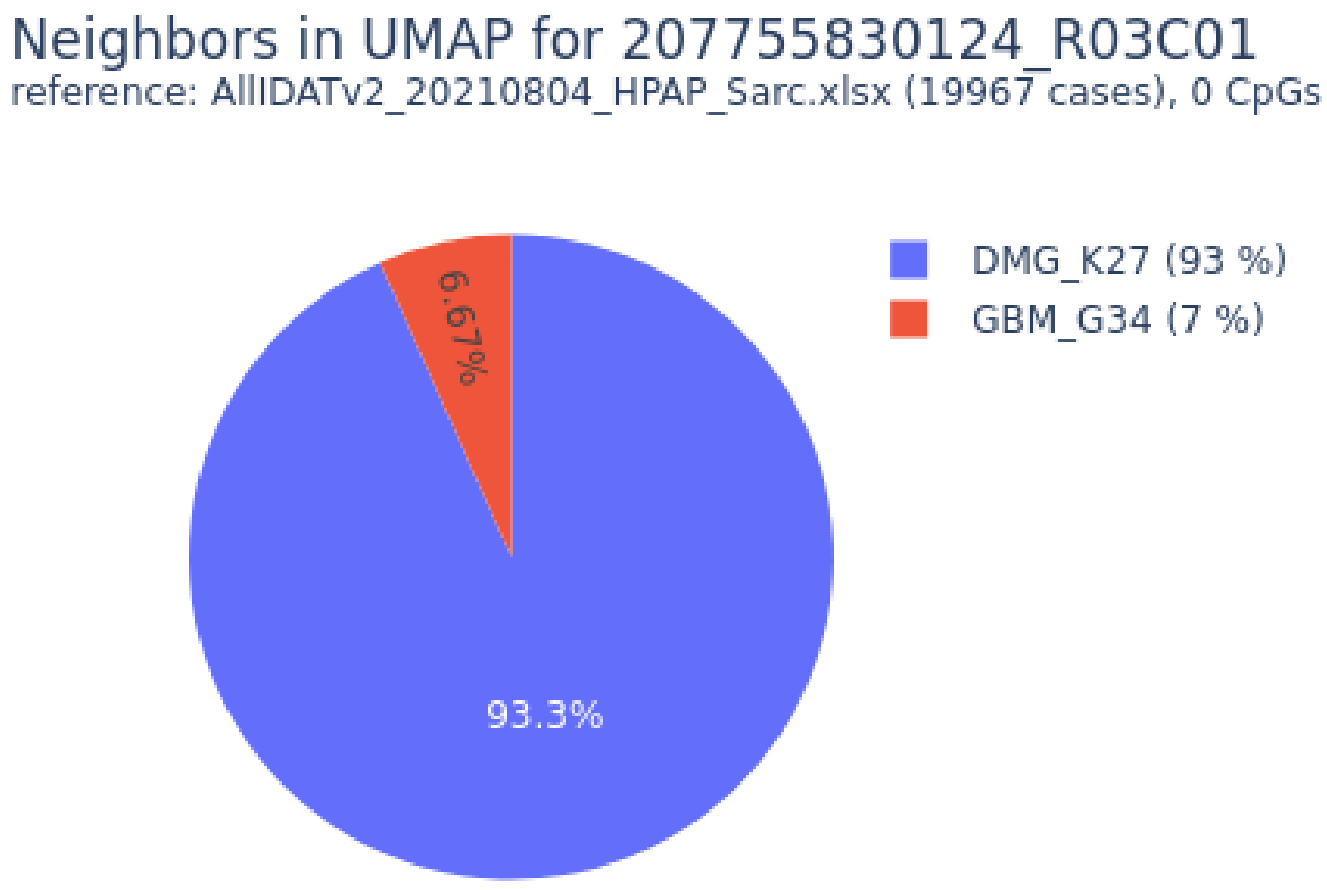

Copy number plot

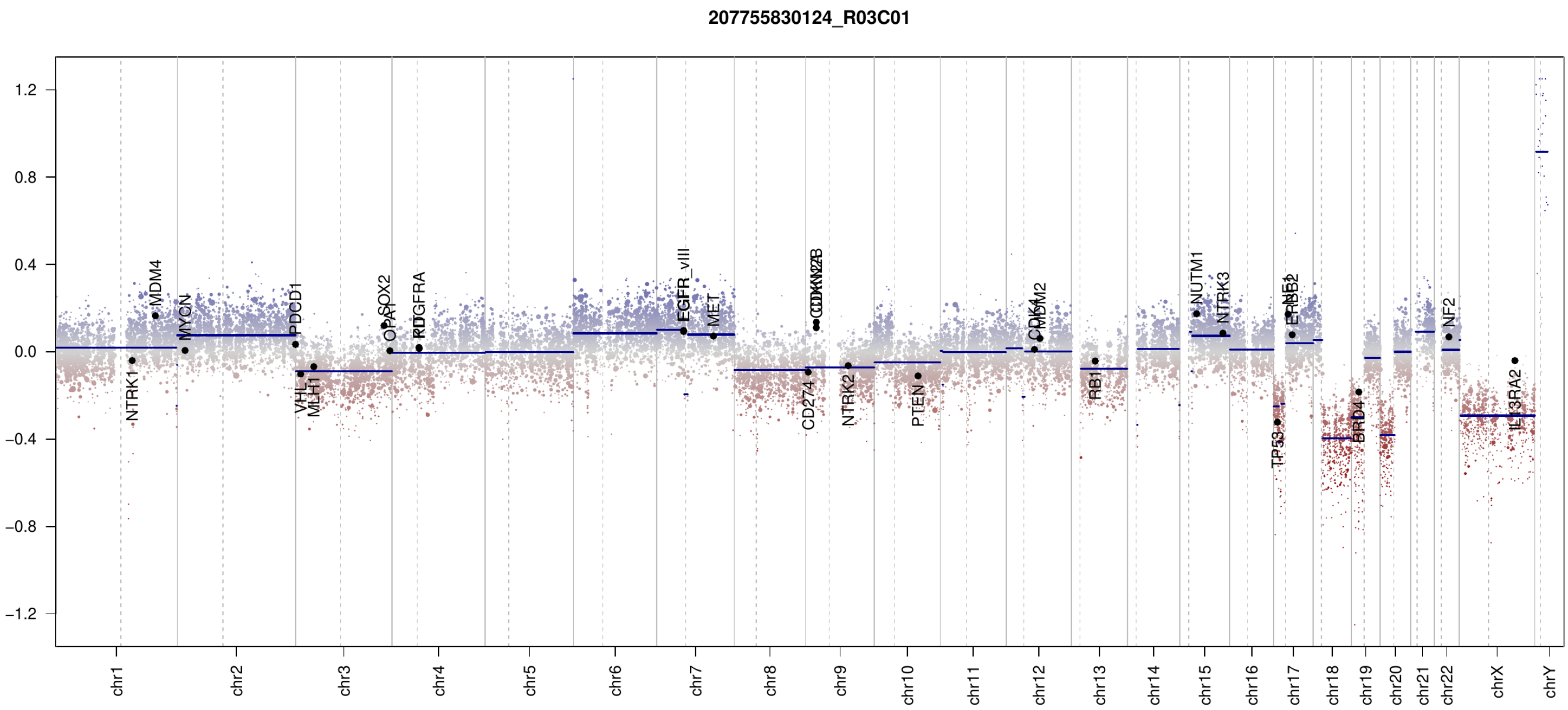

EpiDiP Report for Sample 207953350017_R04C01

Generated on	:	epidip02 / 20260616_173503
Detected barcode	:	undetermined
Valid reads	:	0
450K overlap CpG count	:	0
Reference data	:	AllIDATv2_20210804_HPAP_Sarc

Methylation UMAP plot

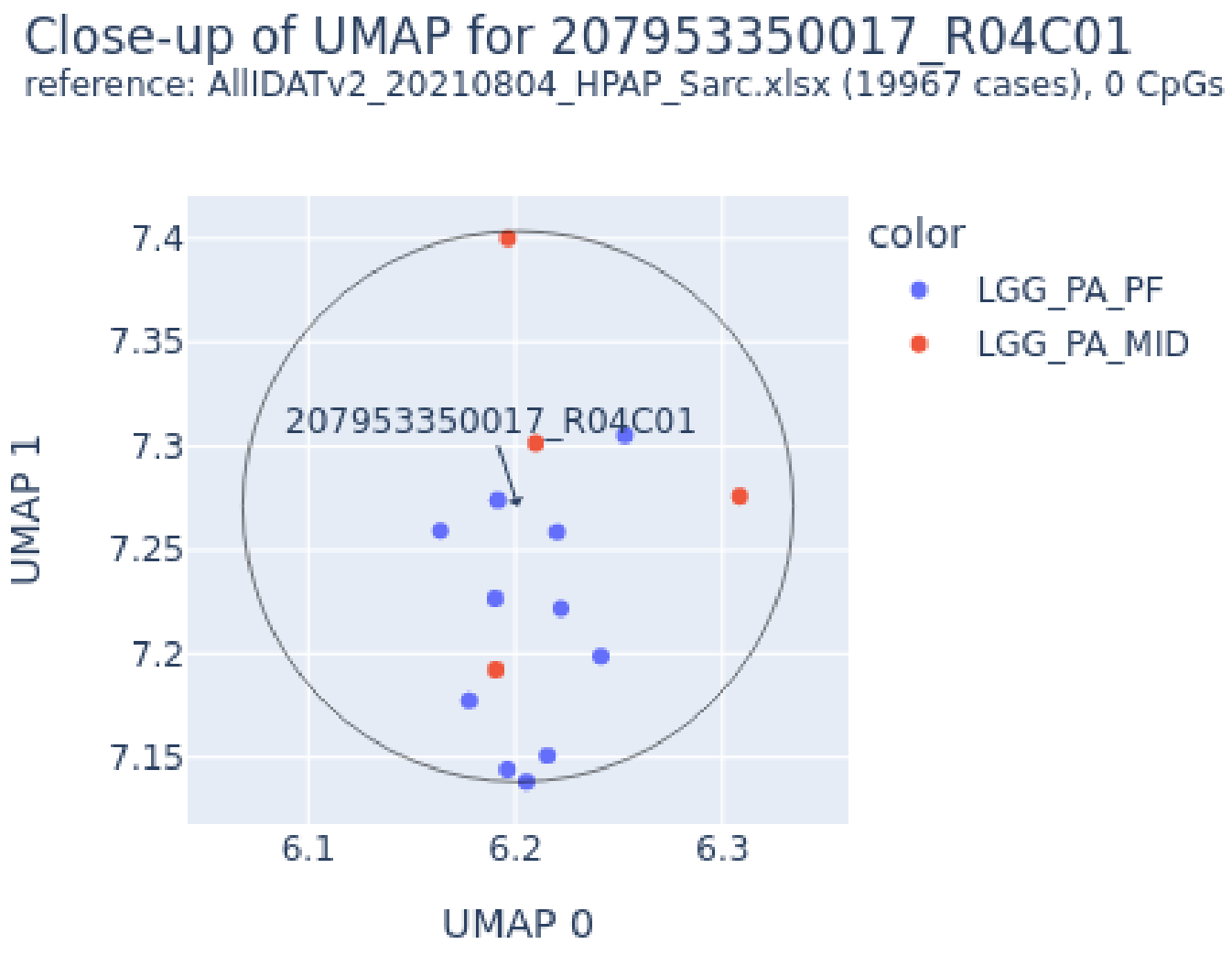

Methylation UMAP Score

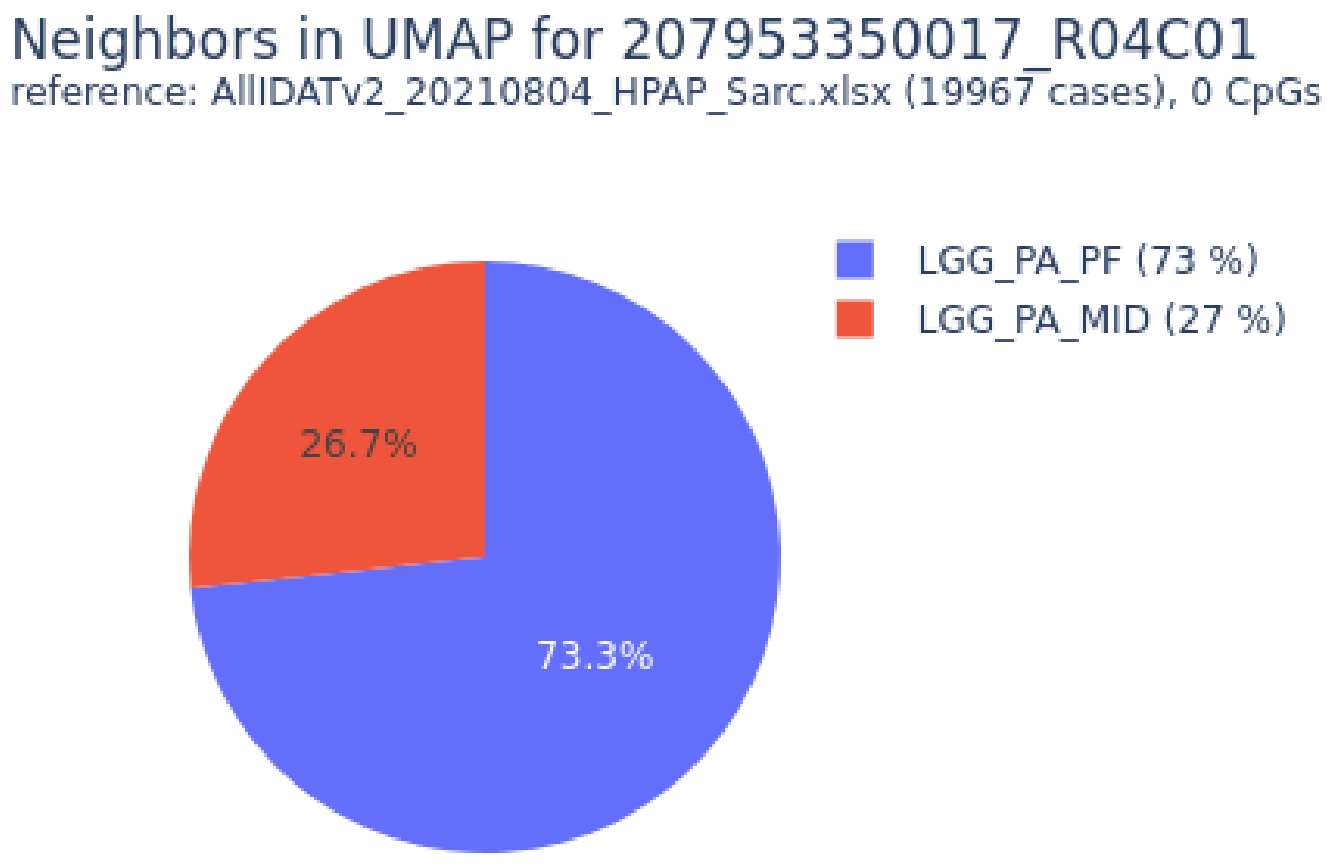

Copy number plot

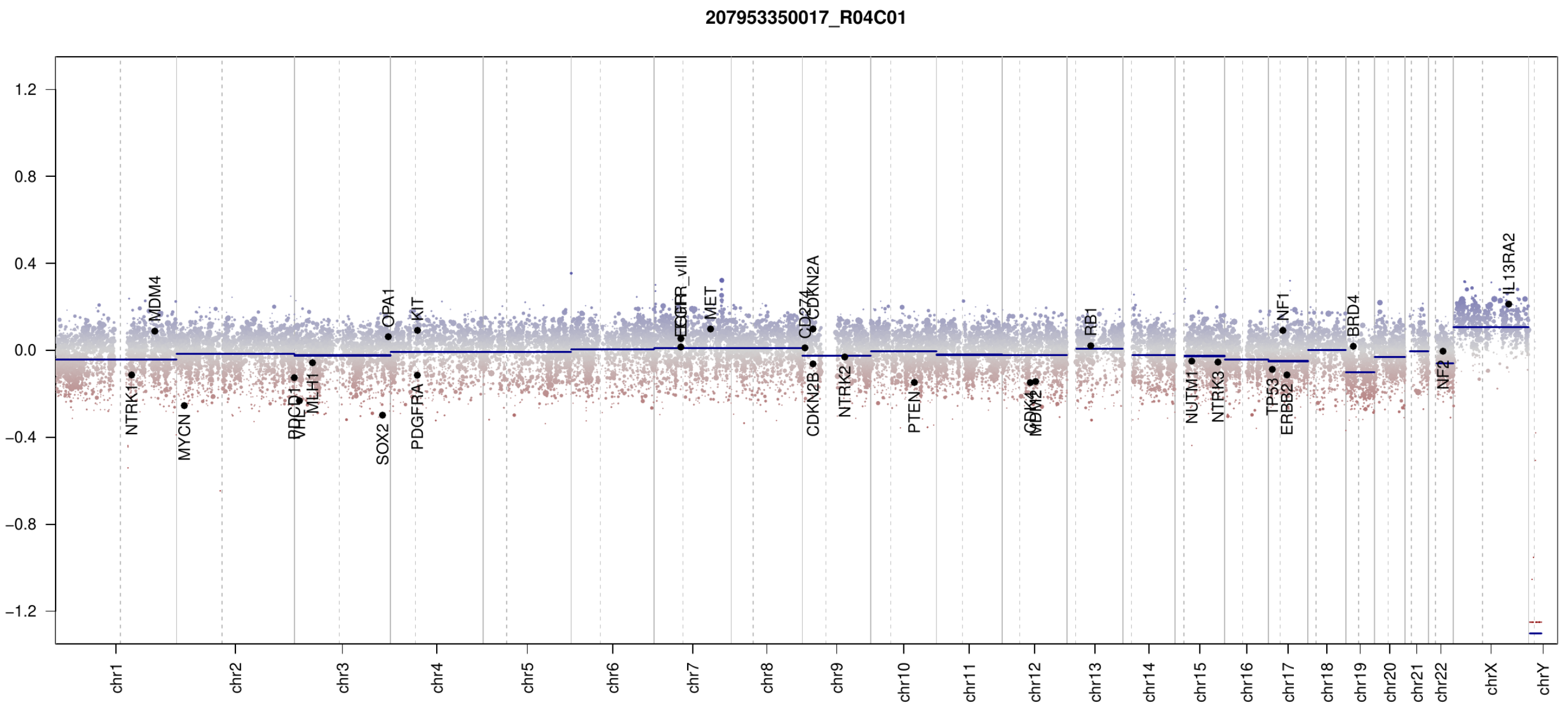

EpiDiP Report for Sample 208010440031_R02C01

Generated on	:	epidip02 / 20260616_171701
Detected barcode	:	undetermined
Valid reads	:	0
450K overlap CpG count	:	0
Reference data	:	AllIDATv2_20210804_HPAP_Sarc

Methylation UMAP plot

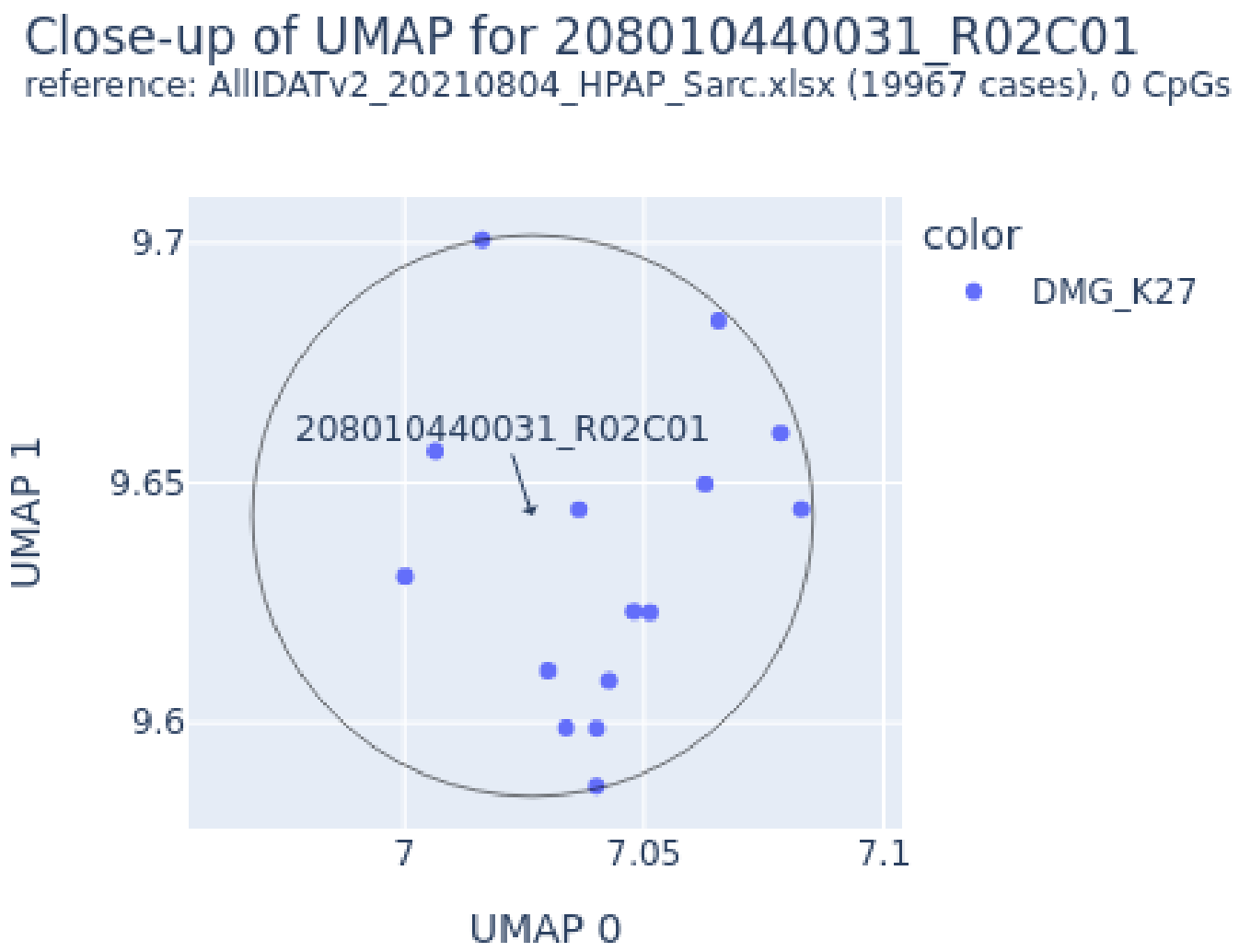

Methylation UMAP Score

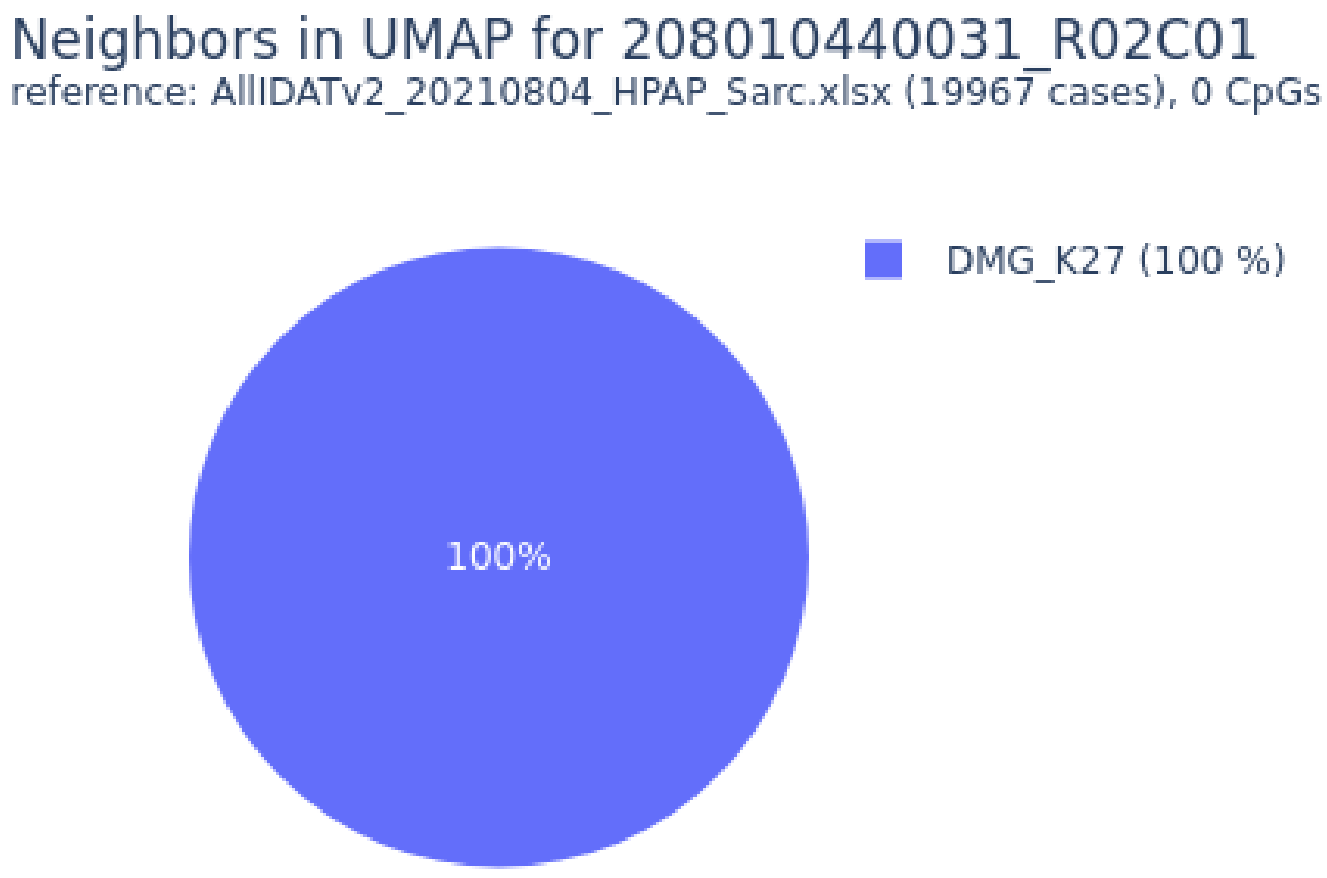

Copy number plot

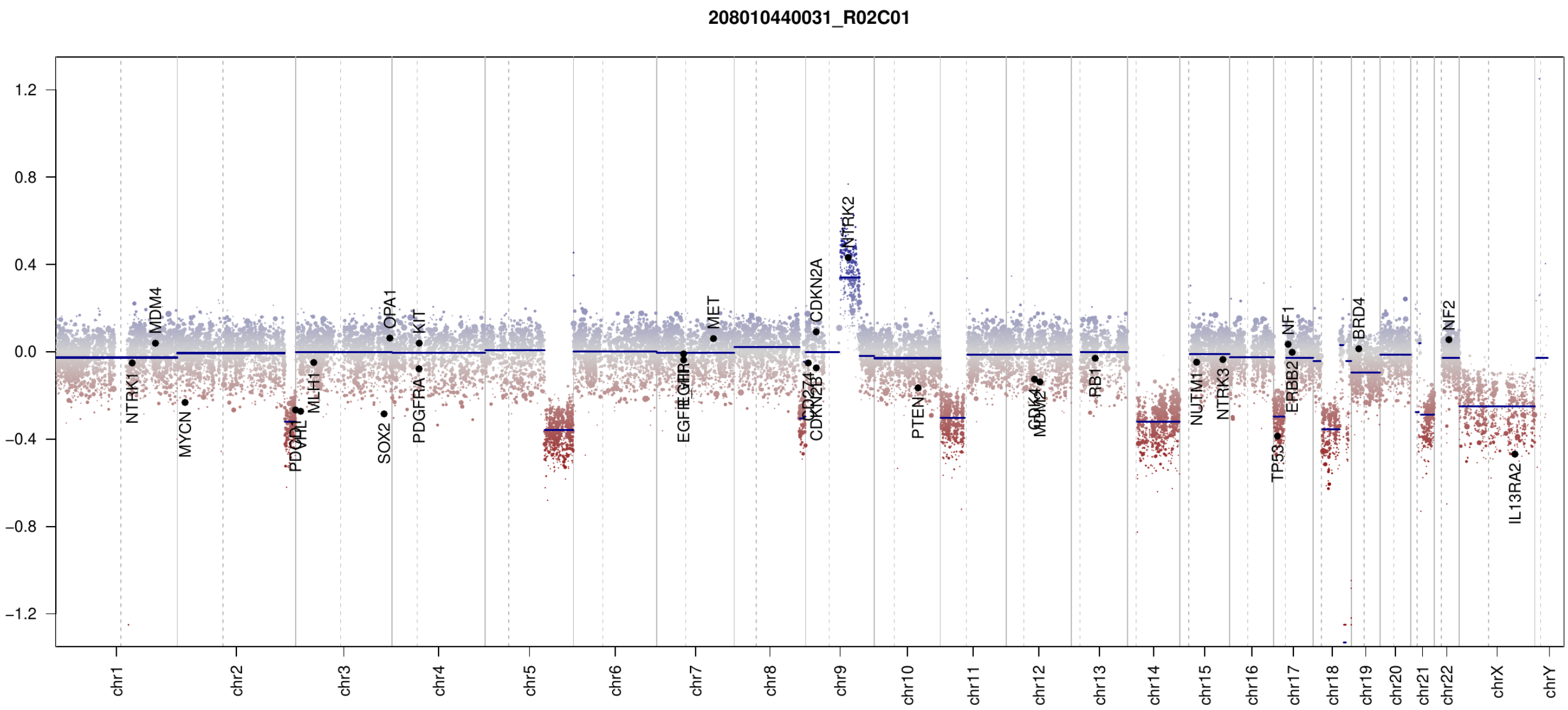

EpiDiP Report for Sample 208066090054_R07C01

Generated on	:	epidip02 / 20260616_172315
Detected barcode	:	undetermined
Valid reads	:	0
450K overlap CpG count	:	0
Reference data	:	AllIDATv2_20210804_HPAP_Sarc

Methylation UMAP plot

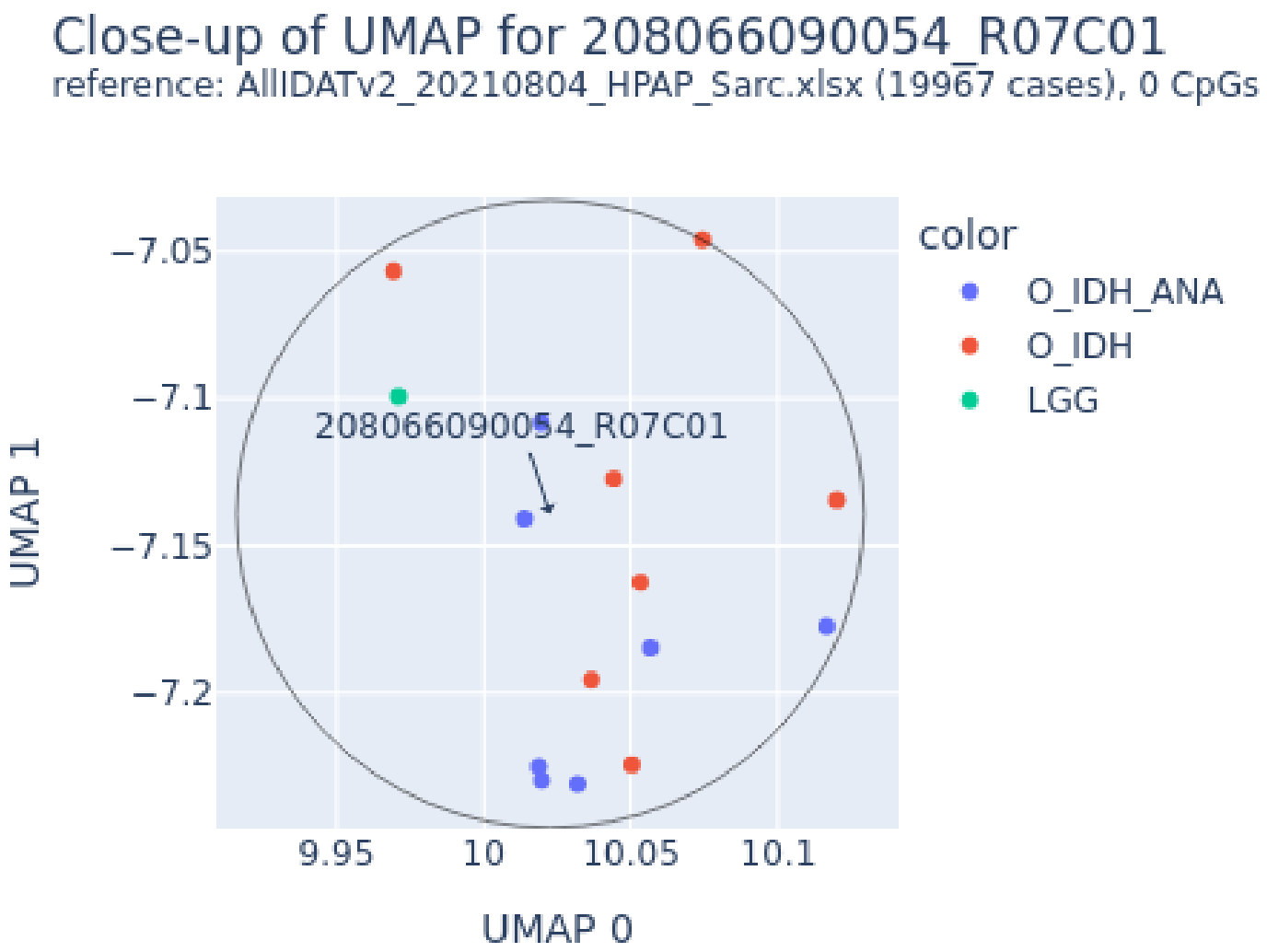

Methylation UMAP Score

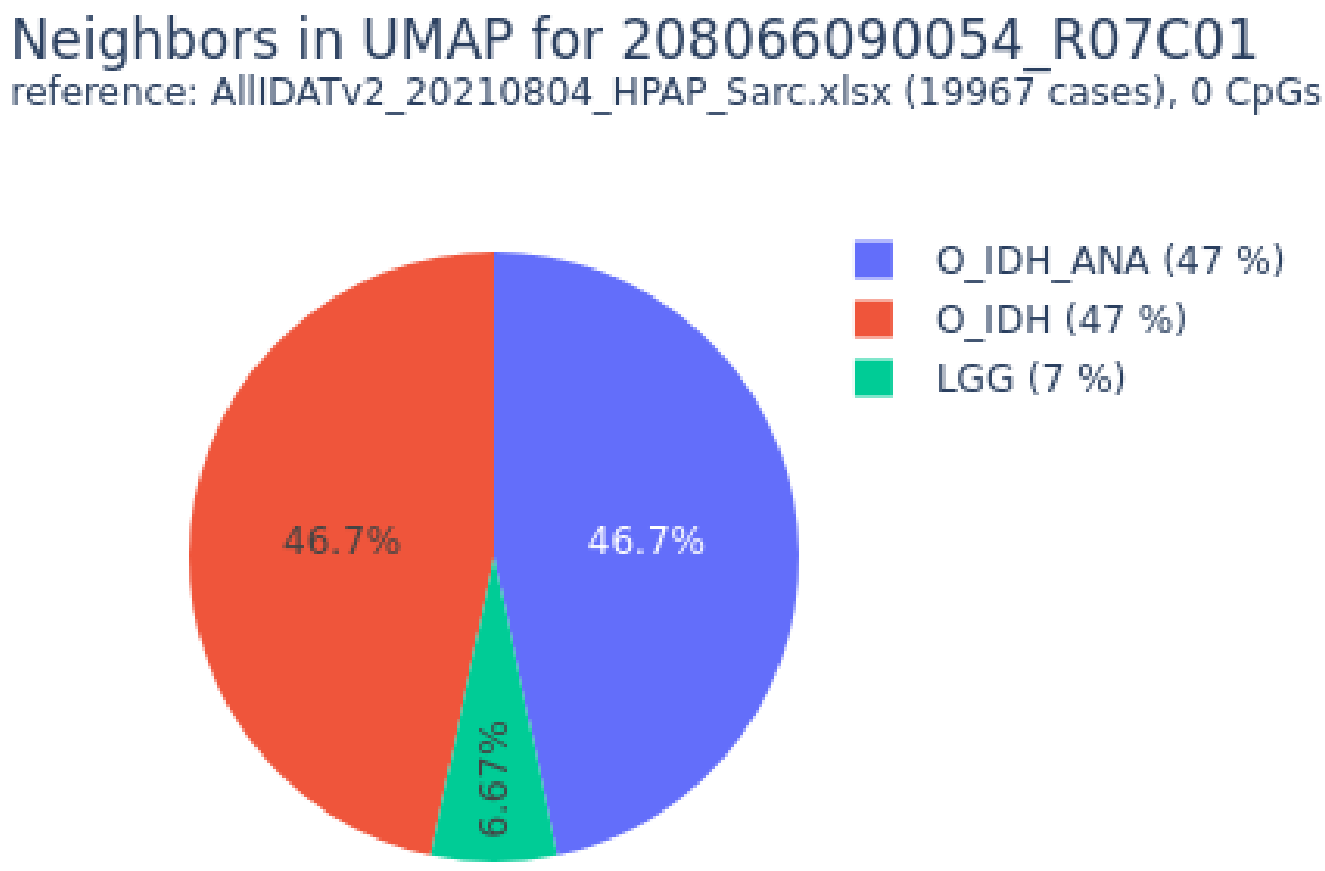

Copy number plot

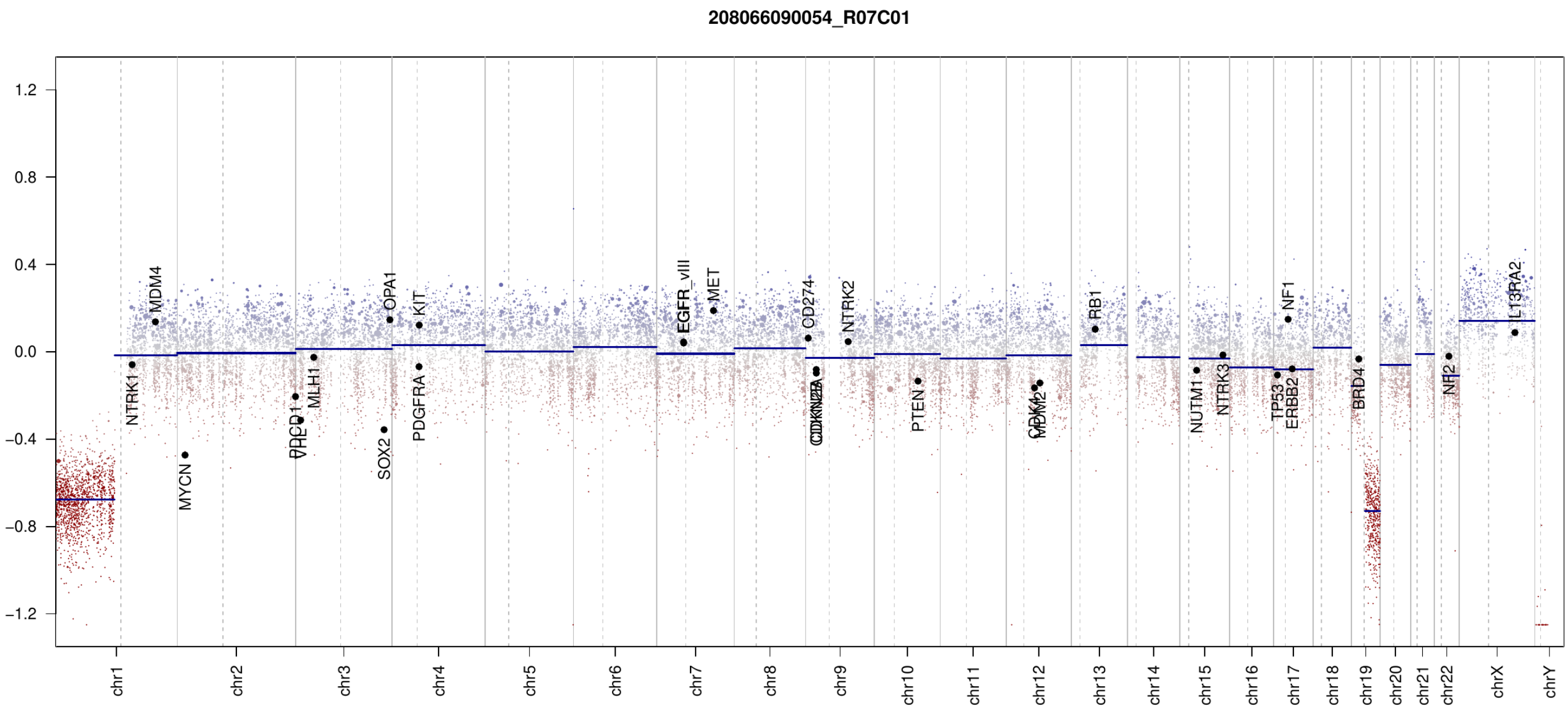

EpiDiP Report for Sample 208069740057_R02C01

Generated on	:	epidip02 / 20260616_172842
Detected barcode	:	undetermined
Valid reads	:	0
450K overlap CpG count	:	0
Reference data	:	AllIDATv2_20210804_HPAP_Sarc

Methylation UMAP plot

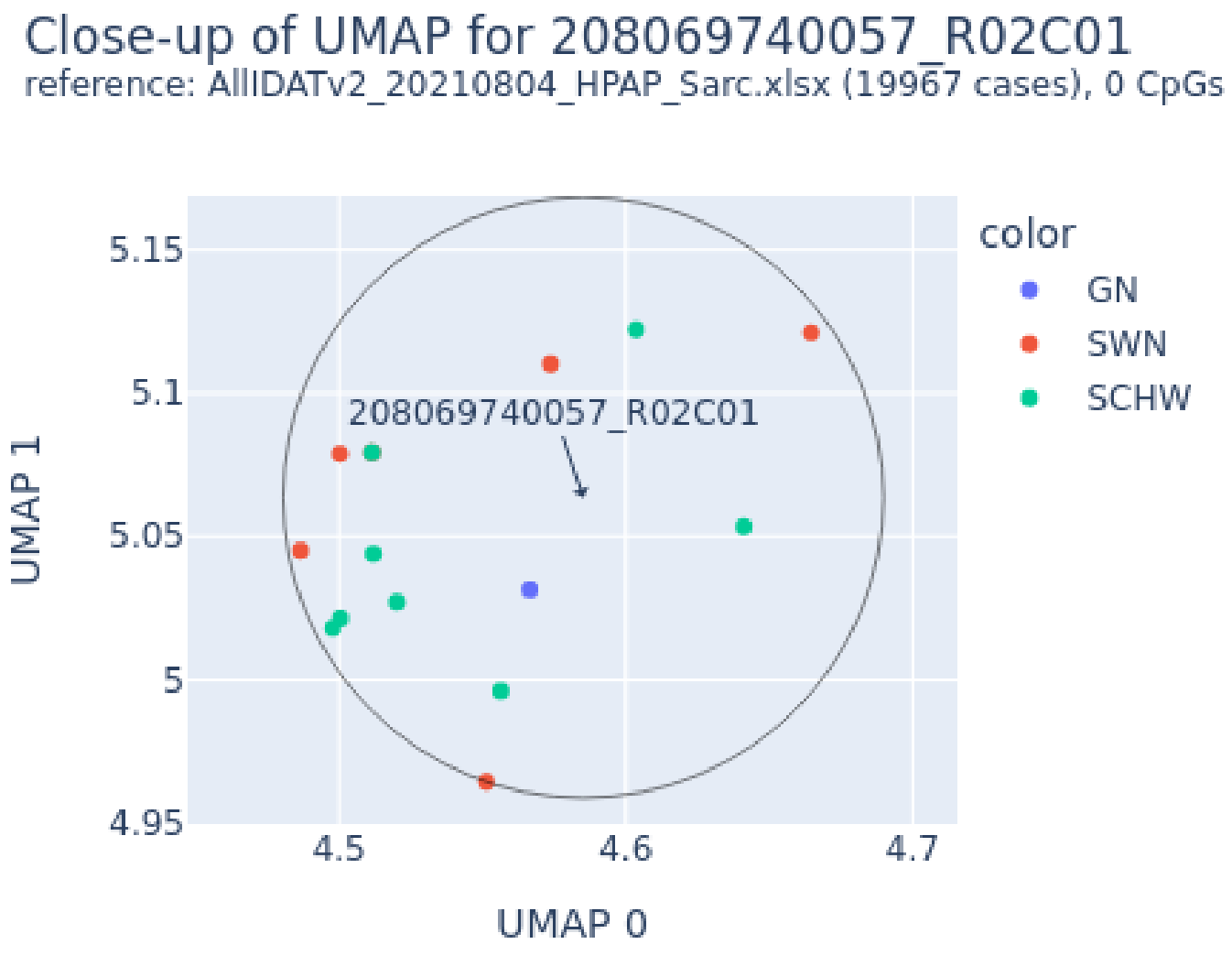

Methylation UMAP Score

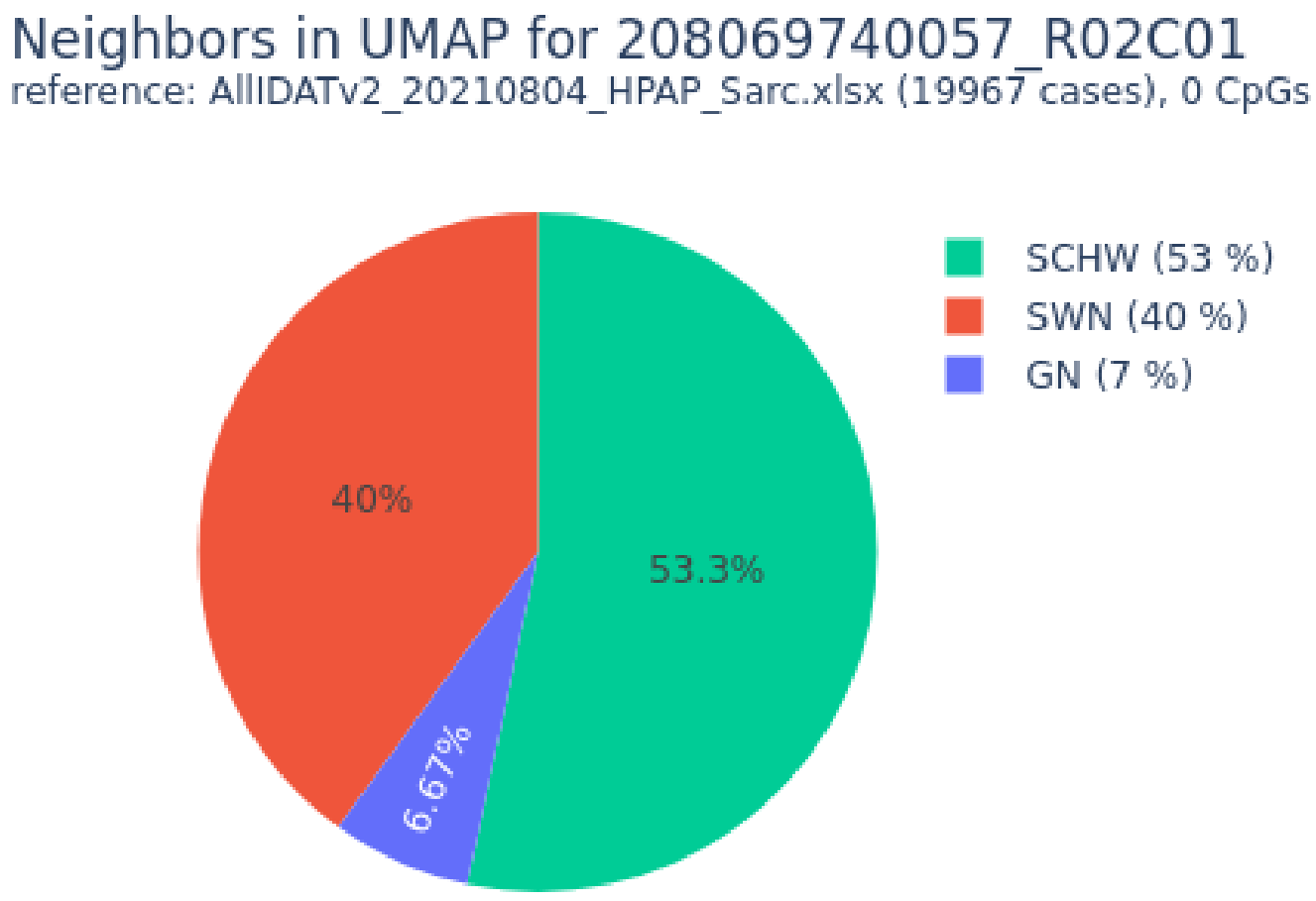

Copy number plot

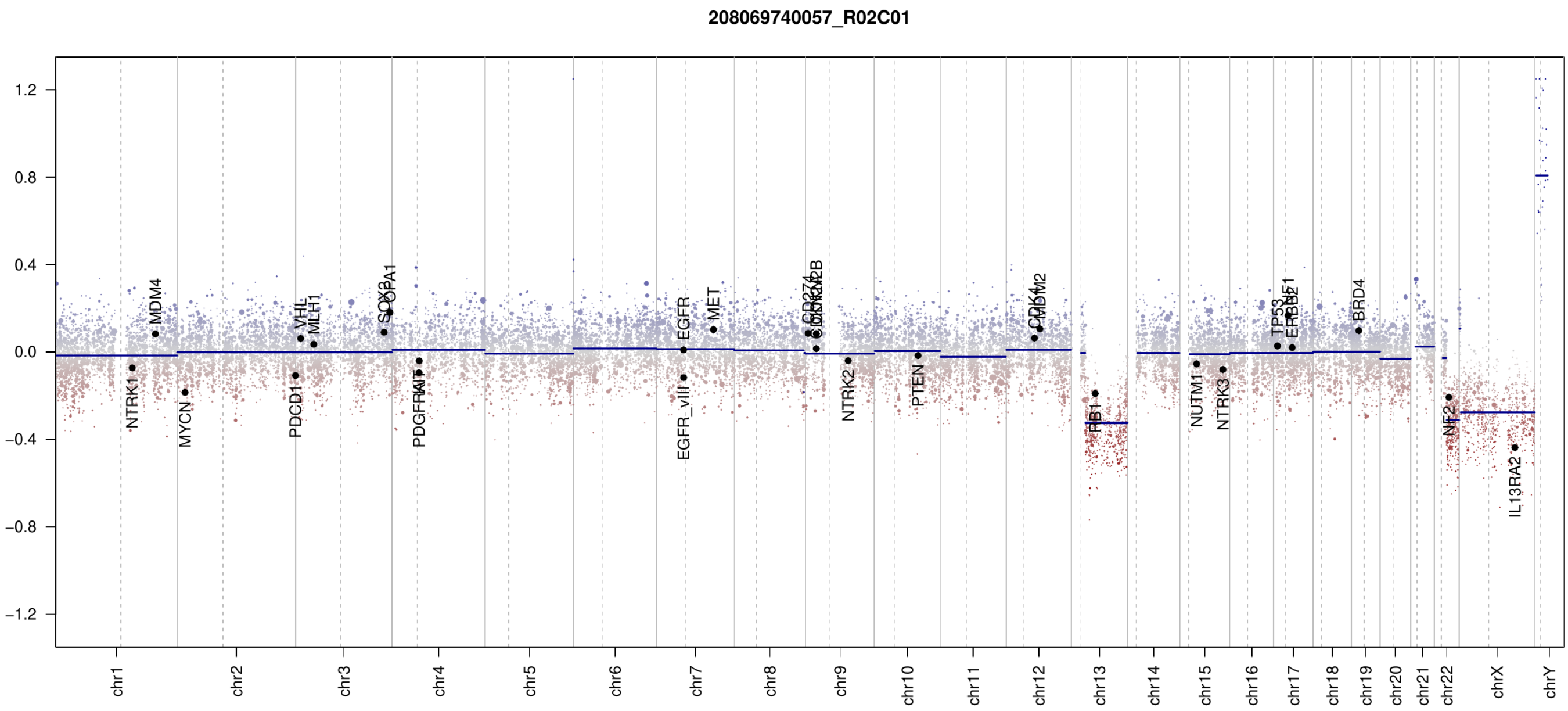

EpiDiP Report for Sample 208160540160_R04C01

Generated on	:	epidip02 / 20260616_171630
Detected barcode	:	undetermined
Valid reads	:	0
450K overlap CpG count	:	0
Reference data	:	AllIDATv2_20210804_HPAP_Sarc

Methylation UMAP plot

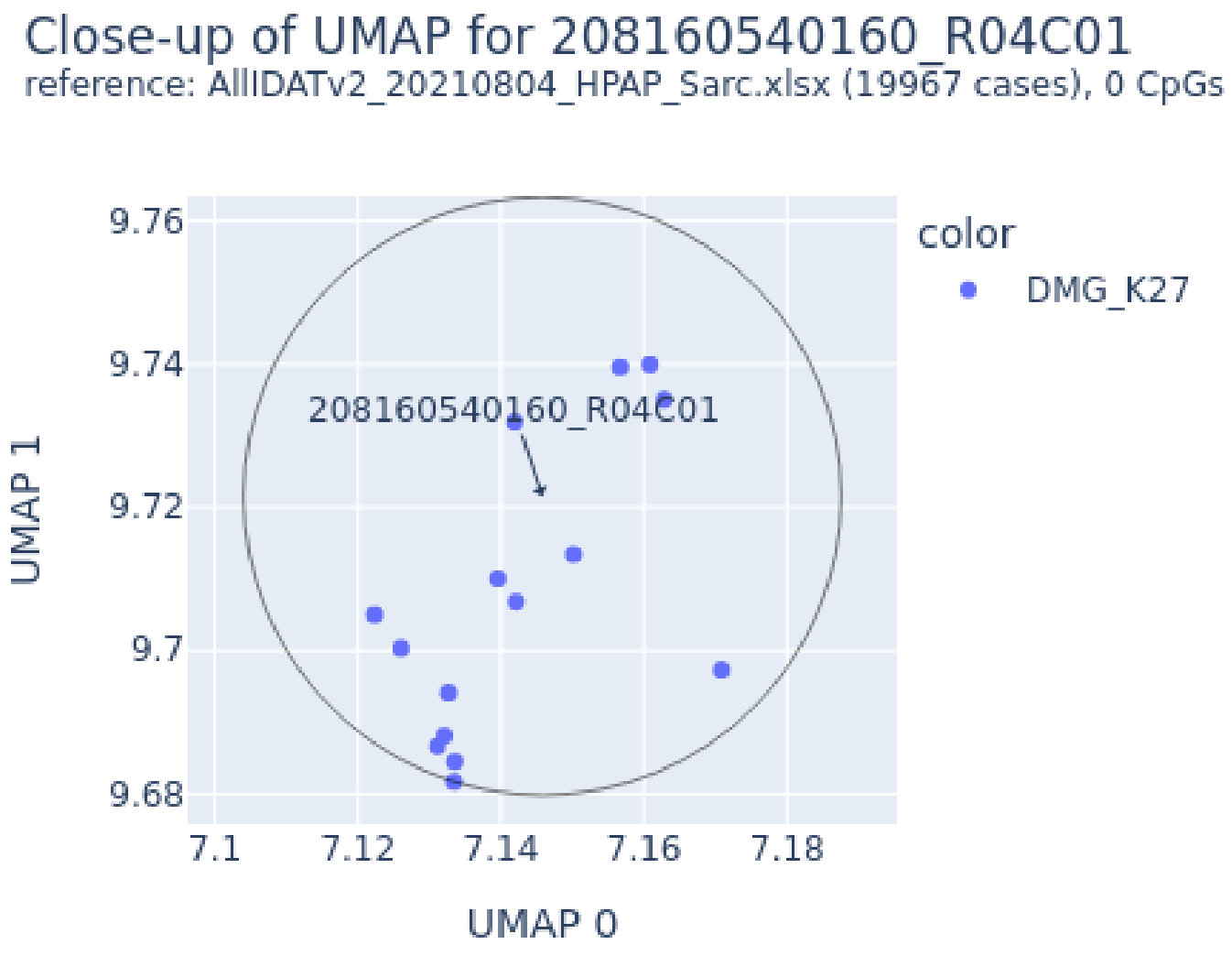

Methylation UMAP Score

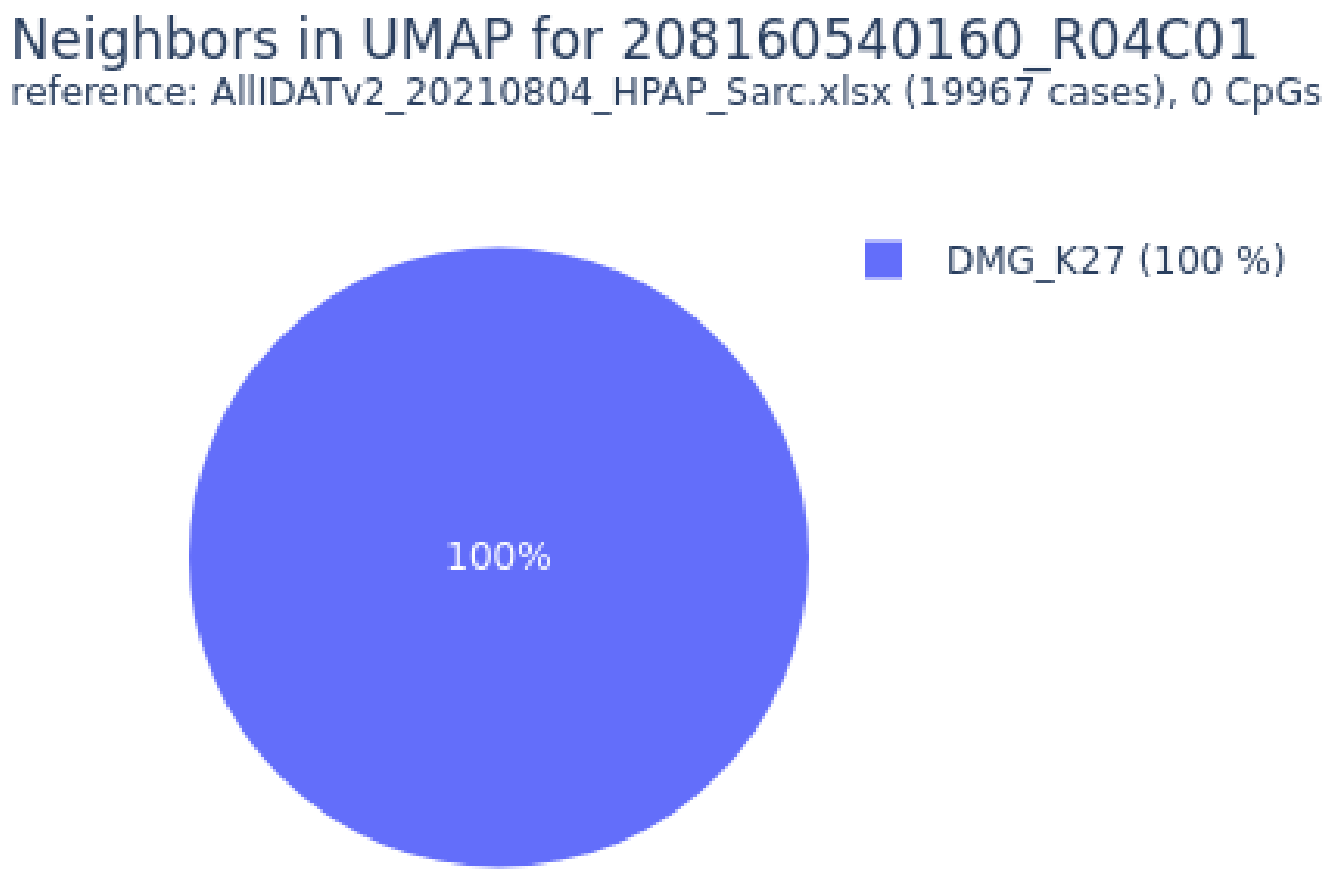

Copy number plot

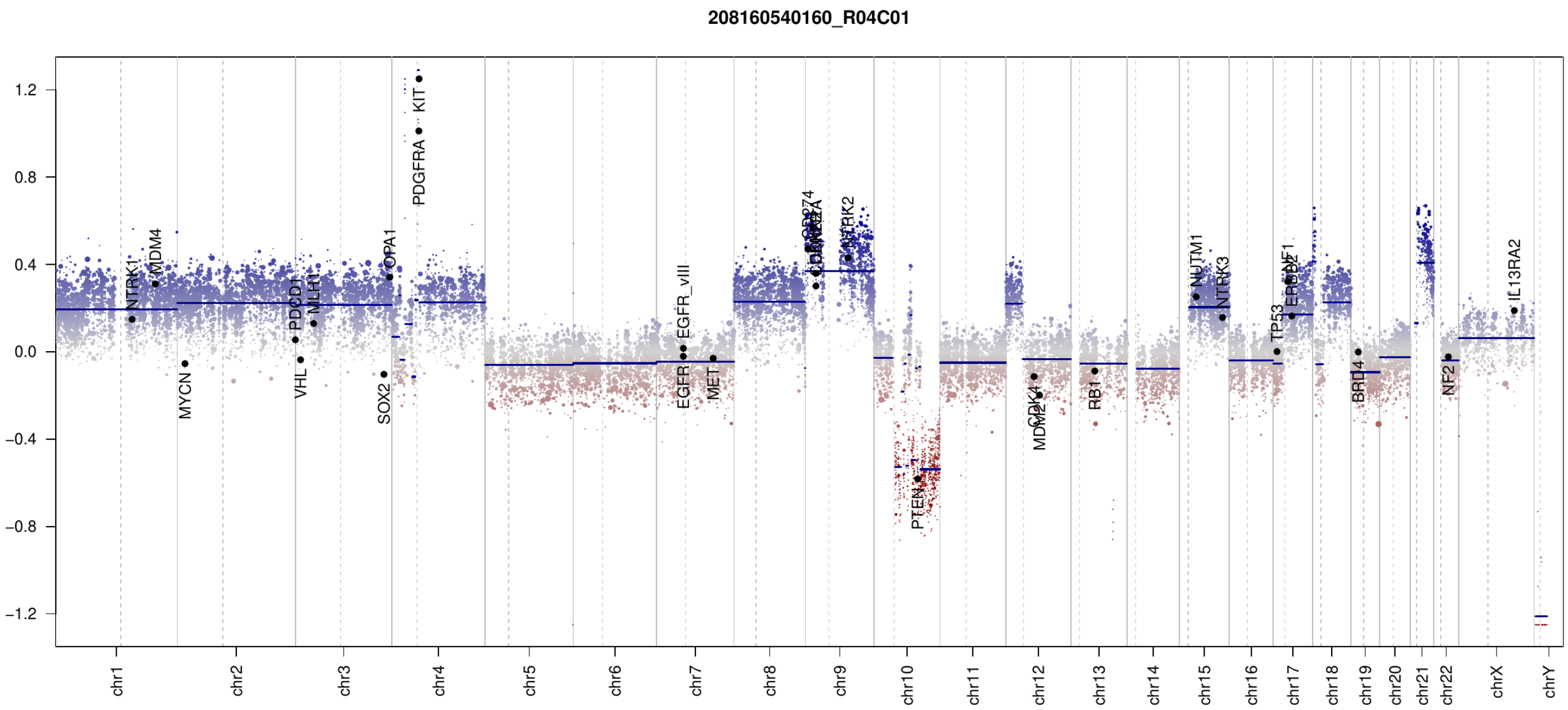

EpiDiP Report for Sample 208285670157_R04C01

Generated on	:	epidip02 / 20260616_171405
Detected barcode	:	undetermined
Valid reads	:	0
450K overlap CpG count	:	0
Reference data	:	AllIDATv2_20210804_HPAP_Sarc

Methylation UMAP plot

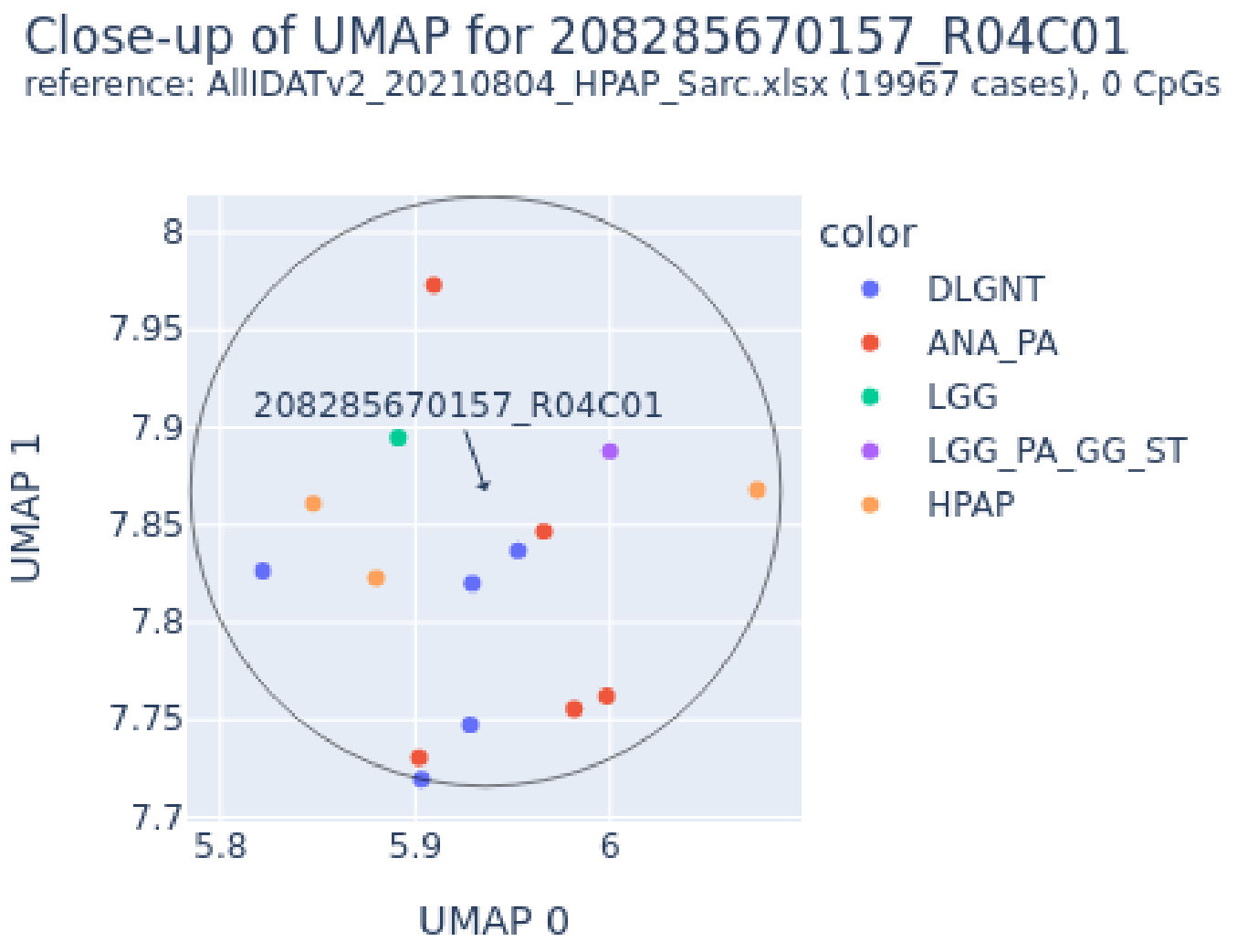

Methylation UMAP Score

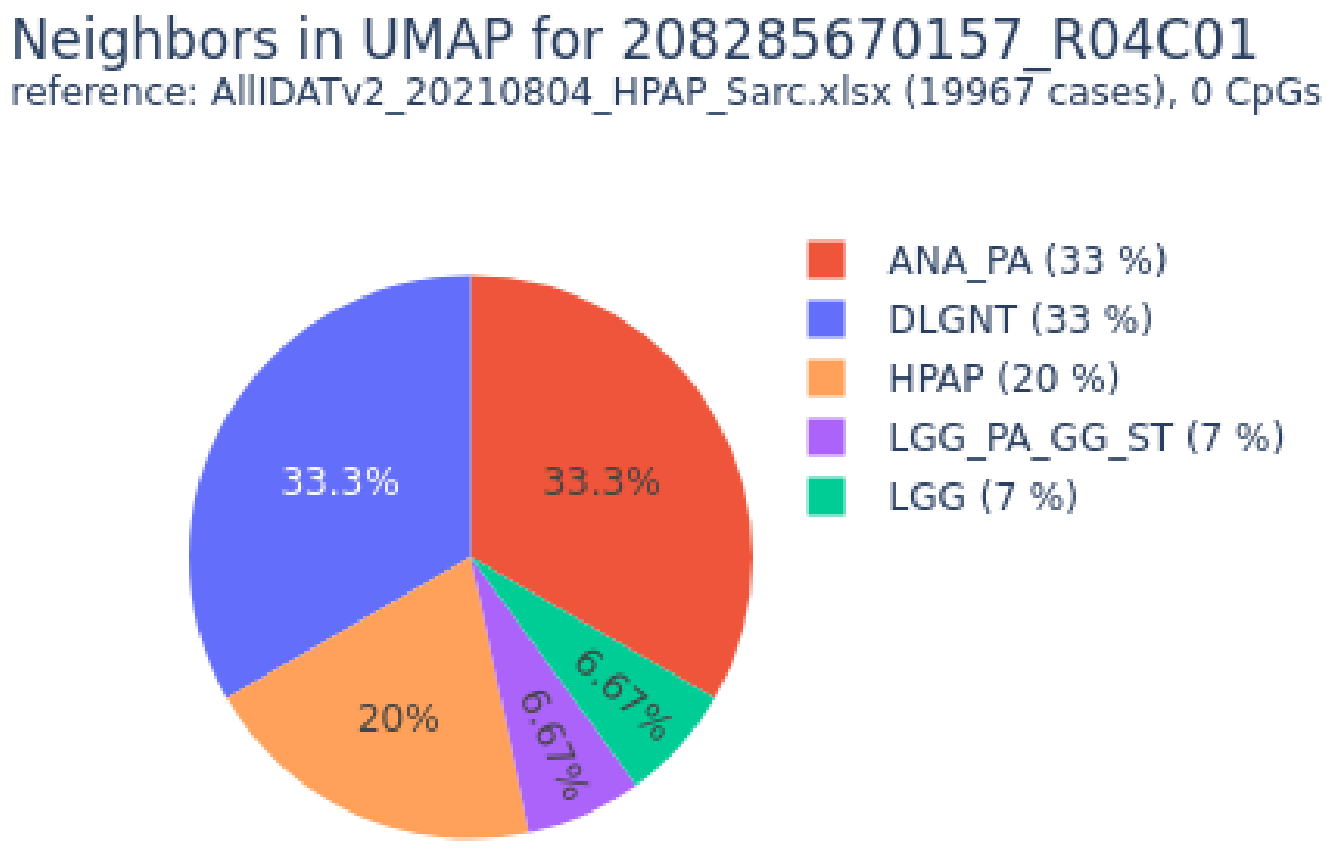

Copy number plot

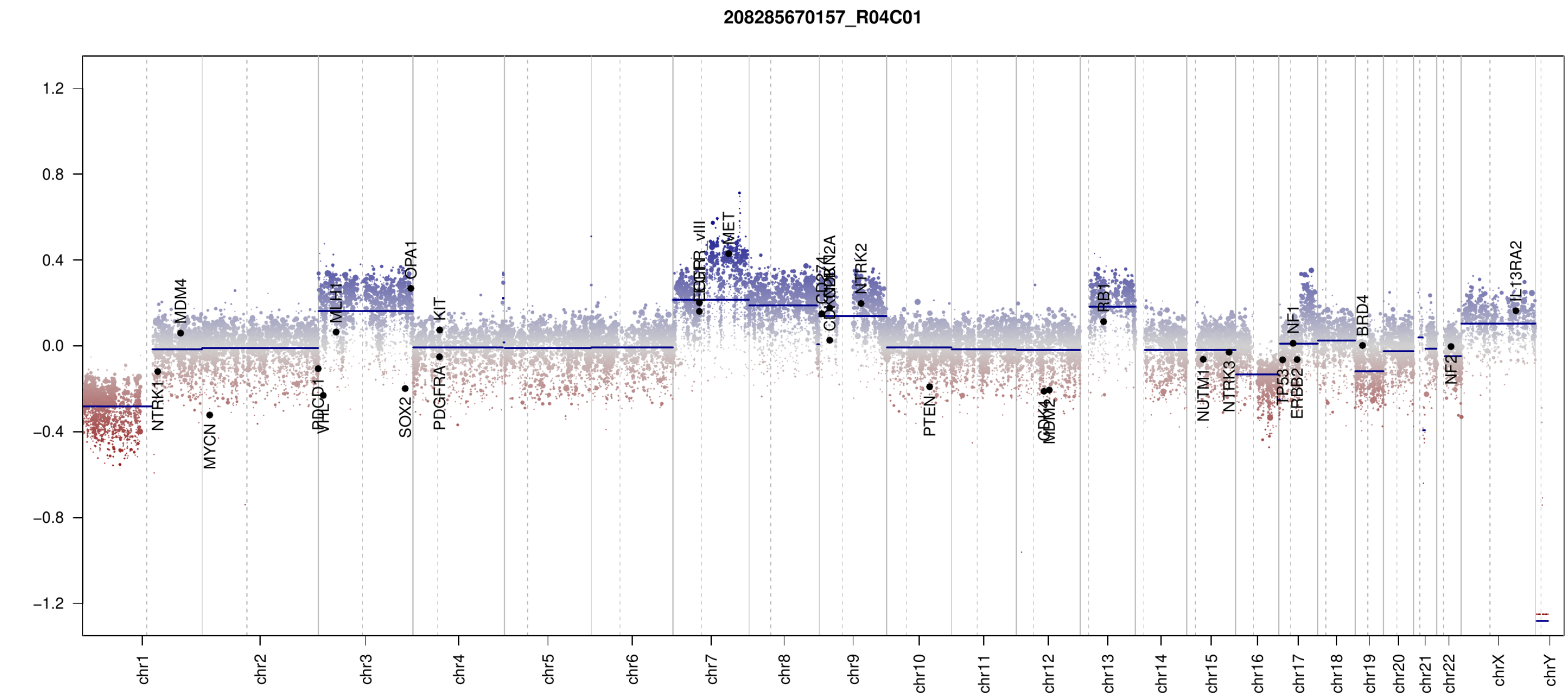

Supplement: Supplementary file 3 [file freeneuropathol-07-21-9648-s4.pdf]
